# Supplementary material for: Concurrence of FGFR1 mutations modulates oncogenesis in glioneuronal tumors
Source: EMBO J. 2025 Oct 31;44(24):7513–40. doi: 10.1038/s44318-025-00600-3 (PMC12705663; doi:10.1038/s44318-025-00600-3)
Supplement: Supplementary file 1 — Appendix [file 44318_2025_600_MOESM1_ESM.pdf]

Title: Concurrence of *FGFR1* mutations modulates oncogenesis in glioneuronal tumors.

Appendix – Table of Contents

Appendix Figures and Tables 2
Appendix Figure S1. 2
Appendix Figure S2. 2
Appendix Figure S3. 3
Appendix Figure S4. 4
Appendix Table S1. 5
Appendix Table S2. Primer sequences for PCR. 5
Appendix Table S3. Guide RNA sequences targeting FGFR1. 5
Appendix Table S4. Donor template sequences. 6
Appendix Table S5. Primer sequences for qPCR. 6

Appendix Figures and Tables

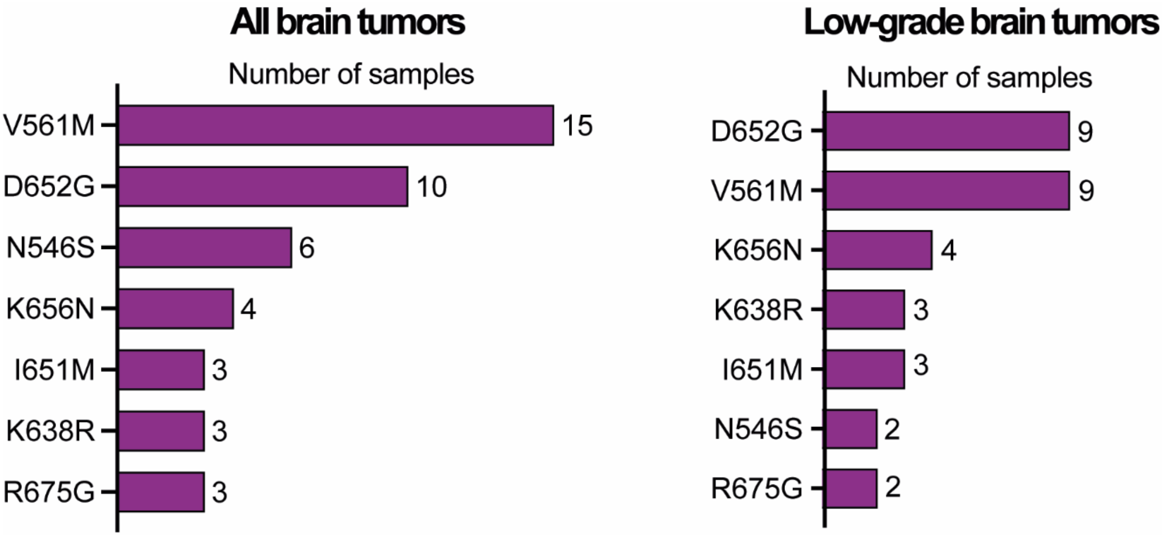

Appendix Figure S1. Most recurrent secondary missense variants in *FGFR1* gene identified in tumor samples from the GENIE database (brain tumor types, see methods section), co-occurring with one of the hotspots N546K/K656E. The majority was identified in low-grade tumors (right panel). Number of cases for each mutation (only mutations appearing in at least n = 2 cases have been plotted).

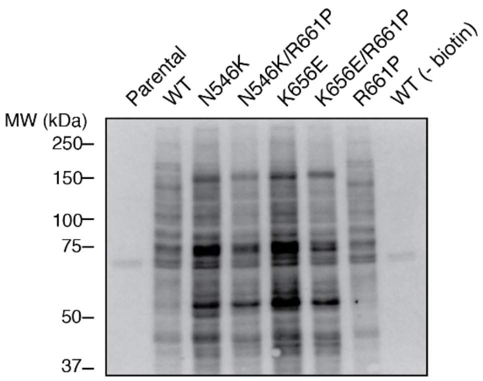

Appendix Figure S2. Western blot showing the profiles of biotinylated proteins for the six baits, 24 hours post Tet-induction and biotin treatment, detected through streptavidin-HRP. Parental cell line and WT FGFR1-BirA*-Flag with no biotin added have been used as controls.

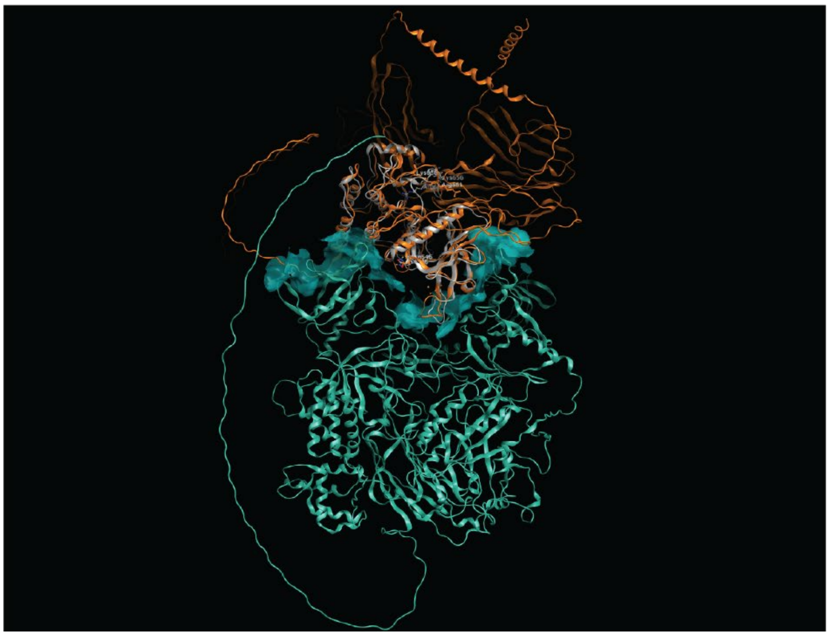

Appendix Figure S3. Overlap of the crystallized human active form of the kinase domain of FGFR1 protein 3GQI and AlfaFold 3 FGFR1-PLCγ complex. In orange; predicted FGFR1 protein by AF3. In light grey: crystallised active FGFR1 kinase domain 3GQI. In light blue: predicted PLCγ protein by AF3.

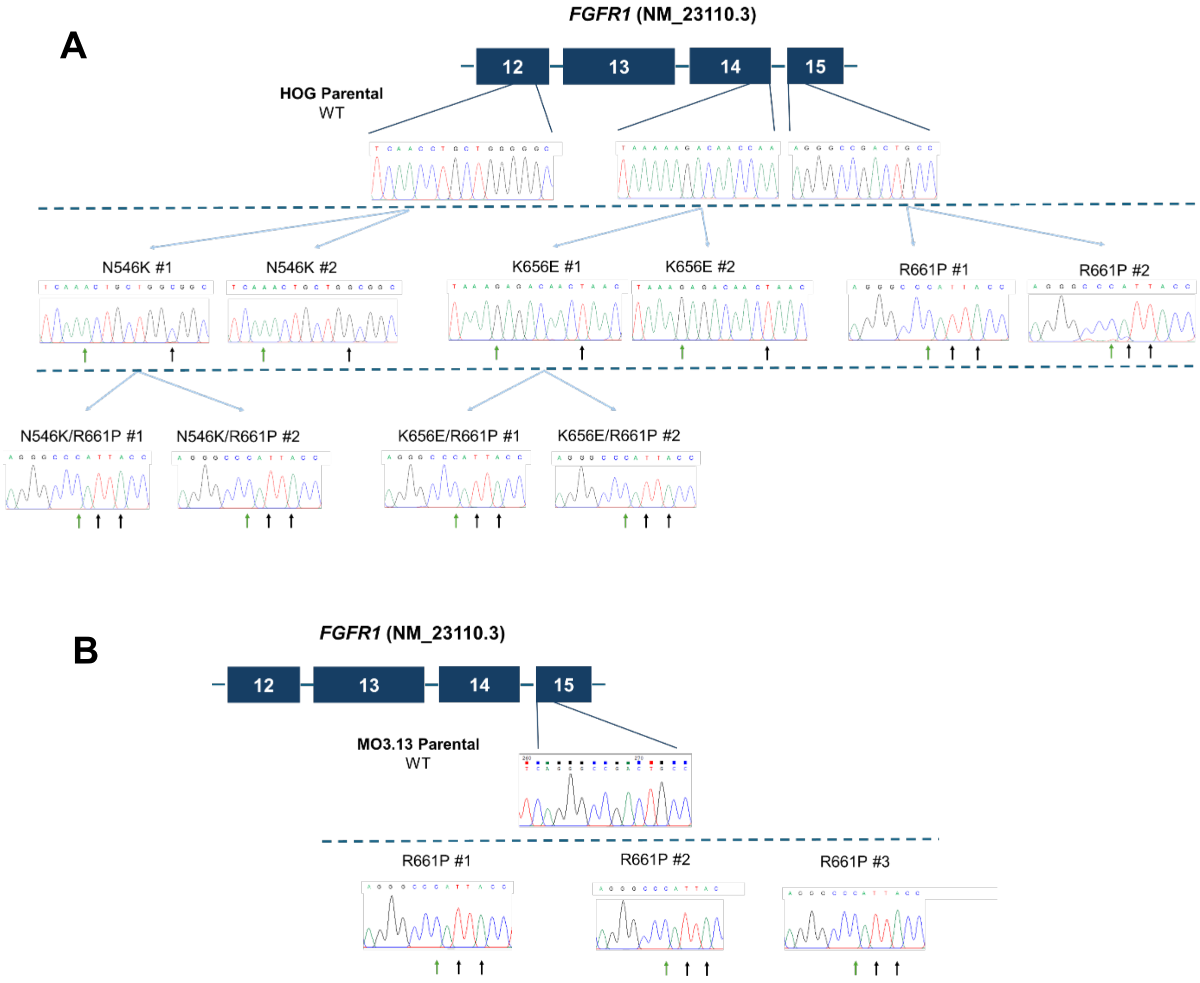

Appendix Figure S4. A) CRISPR/Cas9-editing of HOG cells was performed in multiple steps. Single FGFR1 mutant N546K, K656E and R661P were generated in a first step. R661P editing was performed to generate double mutant clones using N546K #1 and K656E #1 clones as parental cell lines. Correct genome editing was assessed through PCR and Sanger sequencing. **B)** CRISPR/Cas9 editing of MO3.13 cells (R661P mutation). Sequences of the WT locus (Parental cell line) and R661P mutated clones are shown. Blue arrows indicate SNV causing the desired missense mutations, while black arrows indicate additional silent mutations (PAM and/or restriction sites).

N546K	
**Amino acid change**	**Number of patients**
N546K	213
N546D	24
N546S	6
N546H	1
Total	244

K656E	
**Amino acid change**	**Number of patients**
K656E	121
K656N	11
K656M	4
K656Q	1
K656D*	3
Total*	137

Appendix Table S1. Distribution of each missense variants identified at FGFR1 codon N546 (N546K, N546D, N546S and N546H) and K656 (K656E, K656N, K656M, K656Q and K656D) in patients from the GENIE cohort. *K656D is most likely a combination of the missense variants K656N and K656E and therefore K656D cases have been counted also in the K656E and K656N categories.

Primer	Forward	Reverse
***FGFR1* Exon 14-15**	CGCTTGCTGTGATGAGAAGCCTG	GCCTTTCAACATCTGGAGCAGAG
***FGFR1* Exon 12**	CCCACTCCCTTAGCCTTTATCC	CTCTTAACCCCCTTCCCTAGC
***FGFR1* Exon 4-5**	TGTCCGTGTTCATCTGGAACTG	TGAAAAGCATGTAATCAGGACTTC

Appendix Table S2. Primer sequences for PCR.

CRISPR	Forward	Reverse
**N546K**	CACCGAAGAATATCATCAACCTGCT	AAACAGCAGGTTGATGATATTCTTC
**K656E**	CACCGGCCTTGTCGGCACTCACGT	AAACACGTGAGTGCCGACAAGGCC
**R661P**	CACCGCCATCCACTTCACAGGCAGT	AAACACTGCCTGTGAAGTGGATGGC
**KO**	CACCGAGATGCTCTCCCCTCCTCGG	AAACCCGAGGAGGGGAGAGCATCTC

Appendix Table S3. Guide RNA sequences targeting FGFR1.

CRISPR	Sequence
**N546K**	AGATGATGAAGATGATCGGGAAGCATAAGAATATCATCAAACT GCTGGCGGCCTGCACGCAGGATGGTGGGTGCCGGCCAGAC
**K656E**	AGATGAAACCACCAGCACAGGGCGGCCTTGTCGGCACTCACGTT AGTTGTCTC TTATAGTAGTCGATGTGGTGAATGTCCCGTGCGAGGCCA
**R661P**	CCGGTCAAATAATGCCTCGGGTGCCATCCACTTCACAGGTAAT GGGCCCTGAAAGCAGCACAGGGGAGGTTGGAGTGGCCCCAG

Appendix Table S4. Donor template sequences.

Primer	Forward	Reverse
SOX9	GGCAAGCTCTGGAGACTTCTG	CCCGTTCTTCACCGACTTCC
GLI2	TTTGTCTCTCTCGGATTGCCA	AGGAGAGGCCTTTTTACCCG
HIF1A	AAGCCTTGGATGGTTTTGTTATGG	CCCTTTTTCACAAGGCCATTTCT
WNT5A	GCTTTGCCAAGGAGTTCGTG	CCAGGTTGTACACCGTCCTG
NRP1	TGTGAAGTGGAAGCCCCTAC	TGGTGCTGTCTATGACCGTG
SEMA3E	CCGGTTACGCCTGTCACATA	ACTCGGCCAGTGTATCTCTT
THBS1	CAGGAGCAACCTCTACTCCG	CAGCAGGGATCCTGTGTGT
FN1	CACCTGTACCCACACGGTC	TCCAGGAACCCTGAACTGTAAG

Appendix Table S5. Primer sequences for qPCR.
