# Supplementary material for: Concurrence of FGFR1 mutations modulates oncogenesis in glioneuronal tumors
Source: EMBO J. 2025 Oct 31;44(24):7513–40. doi: 10.1038/s44318-025-00600-3 (PMC12705663; doi:10.1038/s44318-025-00600-3)
Supplement: Supplementary file 5 — Source data Fig. 2 [file 44318_2025_600_MOESM5_ESM.zip › Figure 2/2C/WB 2C.pptx]

## Slide 1
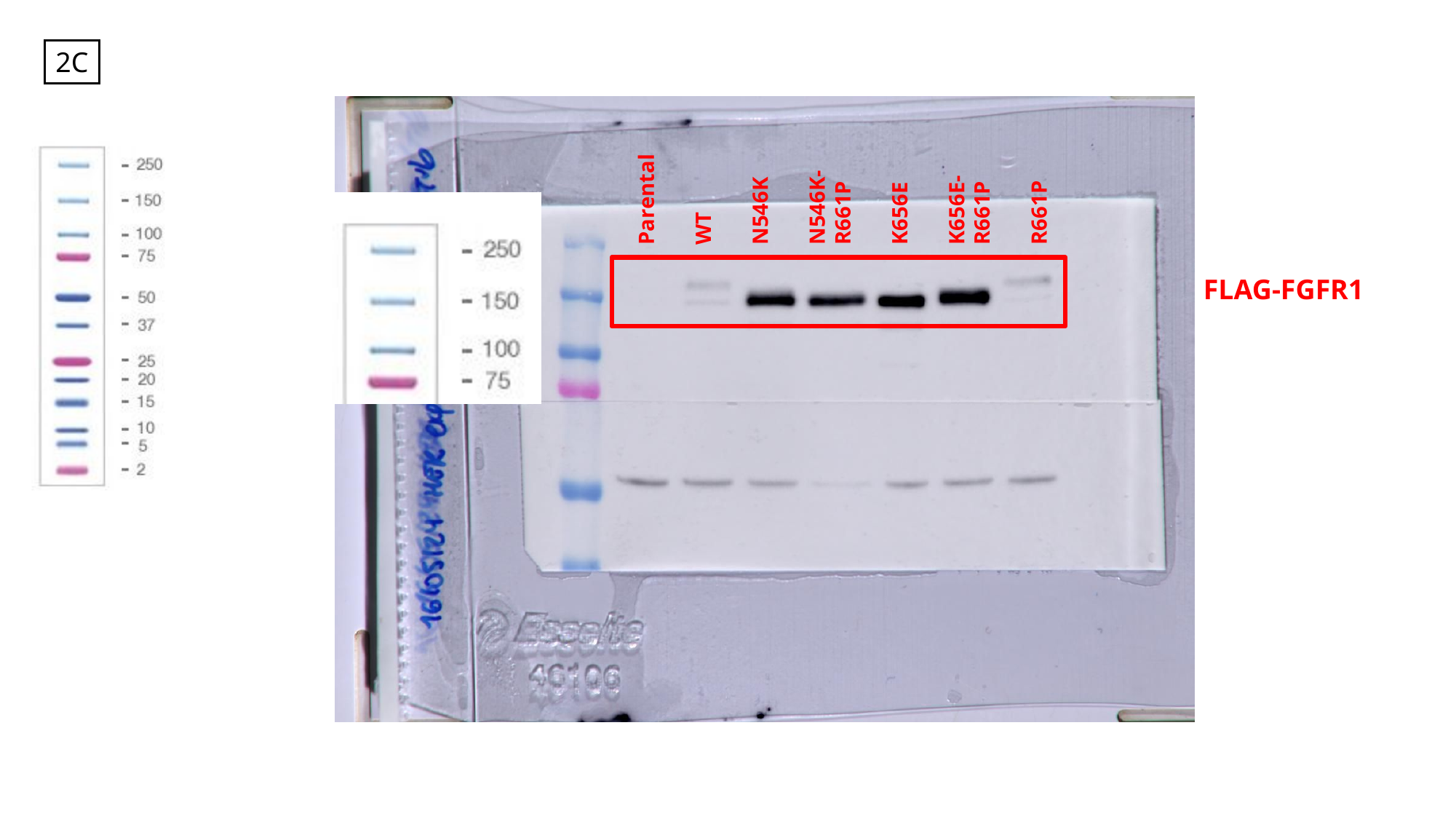

Parental
WT
N546K
N546K-R661P
K656E
K656E-R661P
R661P
2C
FLAG-FGFR1

## Slide 2
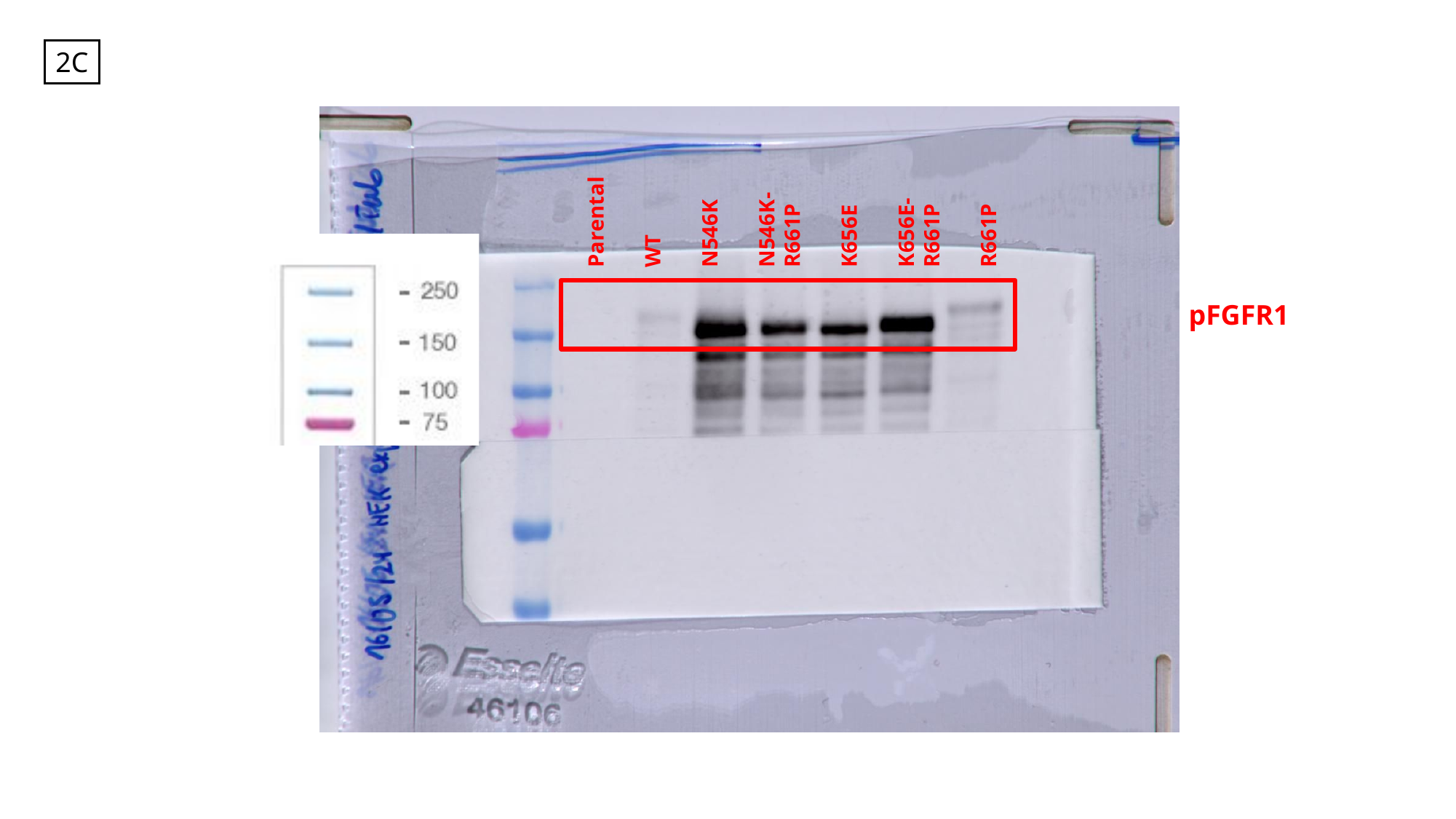

Parental
WT
N546K
N546K-R661P
K656E
K656E-R661P
R661P
2C
pFGFR1

## Slide 3
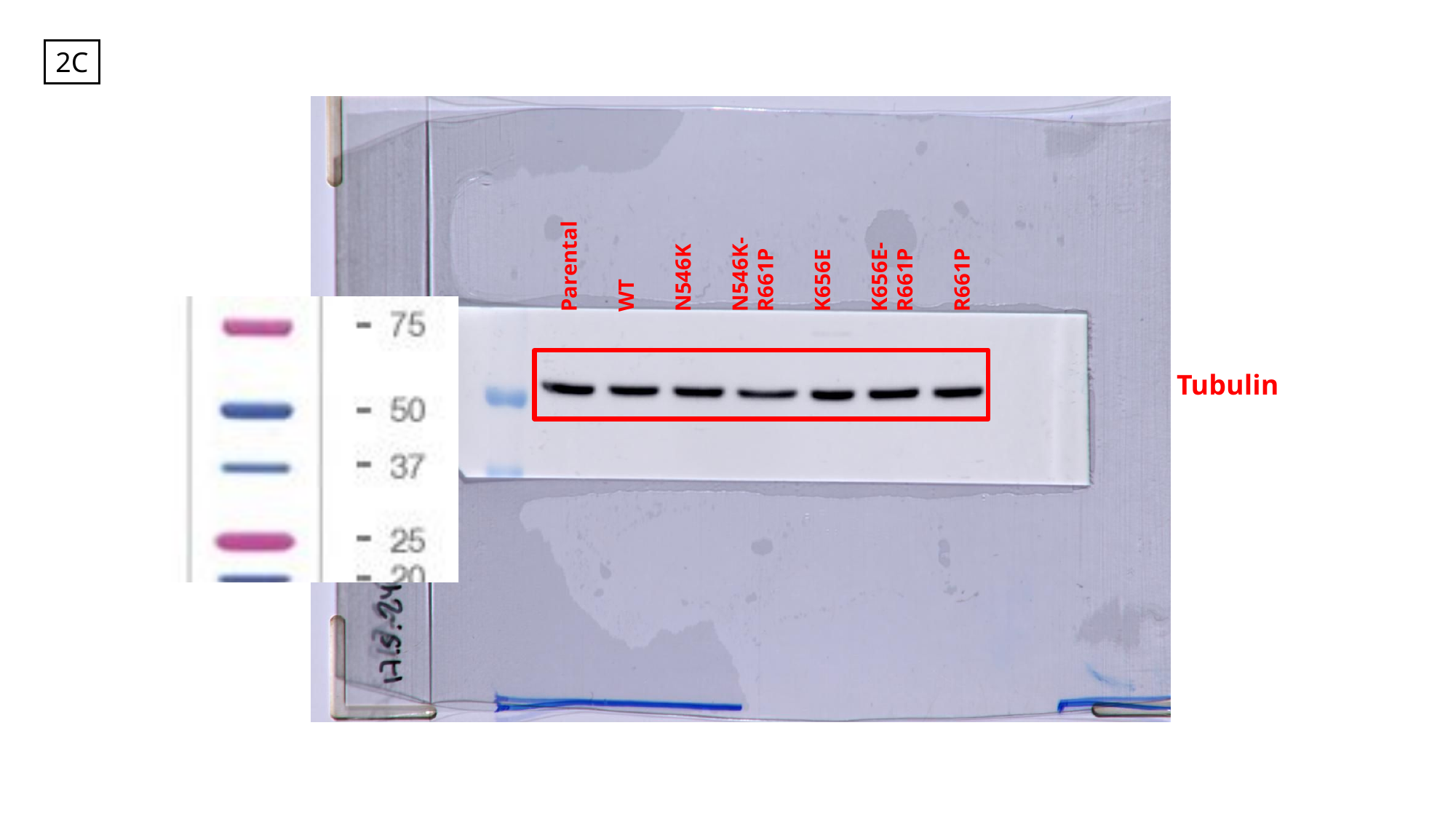

2C
Parental
WT
N546K
N546K-R661P
K656E
K656E-R661P
R661P
Tubulin
