# Supplementary figures and images for: Concurrence of FGFR1 mutations modulates oncogenesis in glioneuronal tumors (part 1 of 2)
Source: EMBO J. 2025 Oct 31;44(24):7513–40. doi: 10.1038/s44318-025-00600-3 (PMC12705663; doi:10.1038/s44318-025-00600-3)

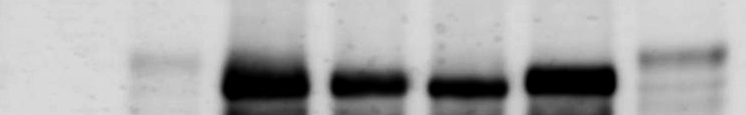

Supplement: Supplementary file 5 — Source data Fig. 2 [file 44318_2025_600_MOESM5_ESM.zip › Figure 2/2C/Fig 2C_pFGFR1.tif]

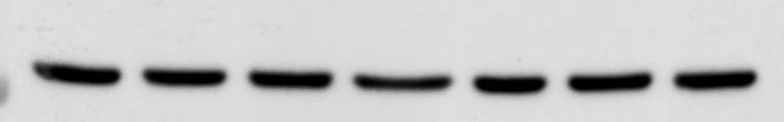

Supplement: Supplementary file 5 — Source data Fig. 2 [file 44318_2025_600_MOESM5_ESM.zip › Figure 2/2C/Fig 2C_TUB.tif]

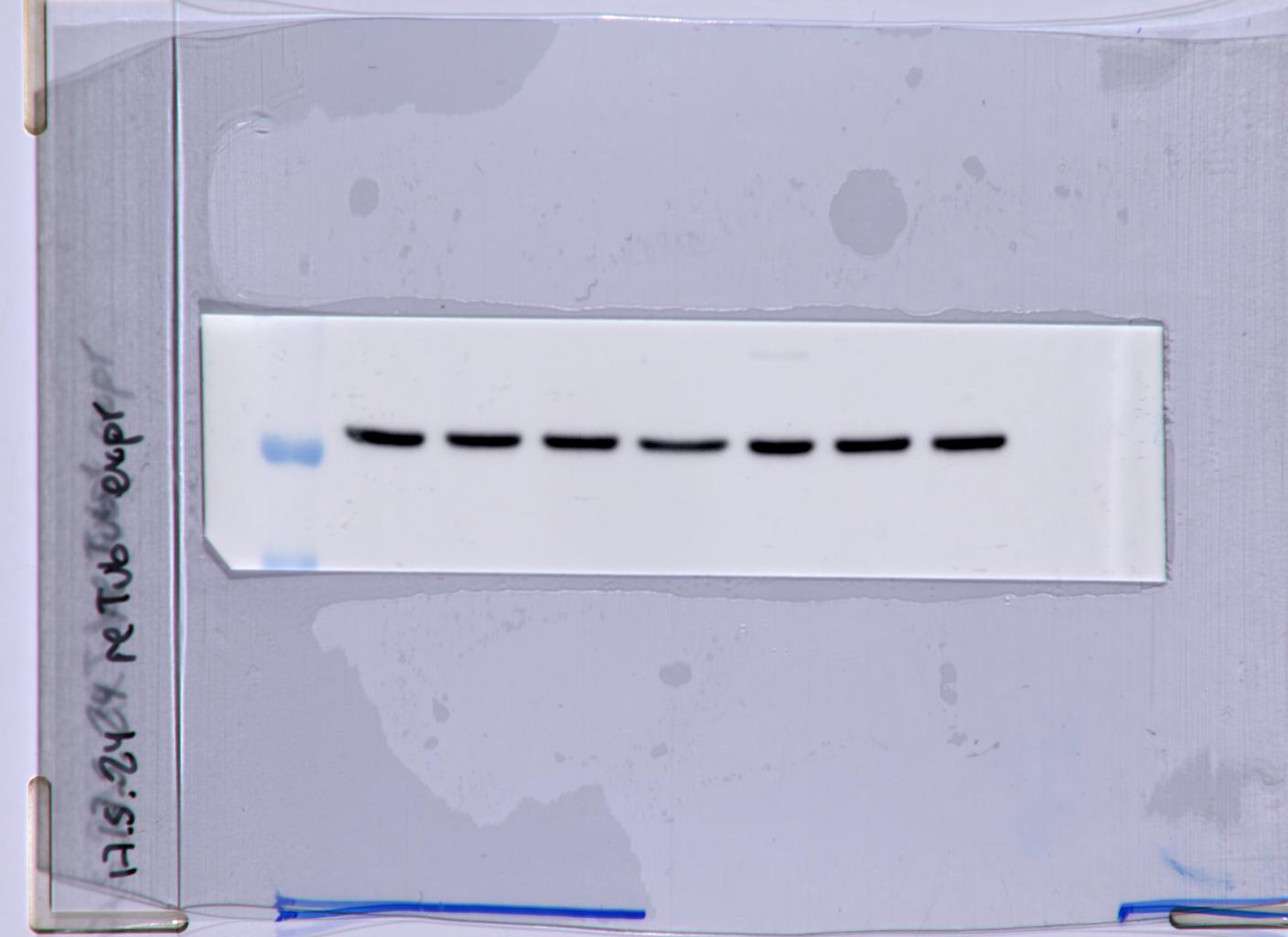

Supplement: Supplementary file 5 — Source data Fig. 2 [file 44318_2025_600_MOESM5_ESM.zip › Figure 2/2C/tubulin original.jpg]

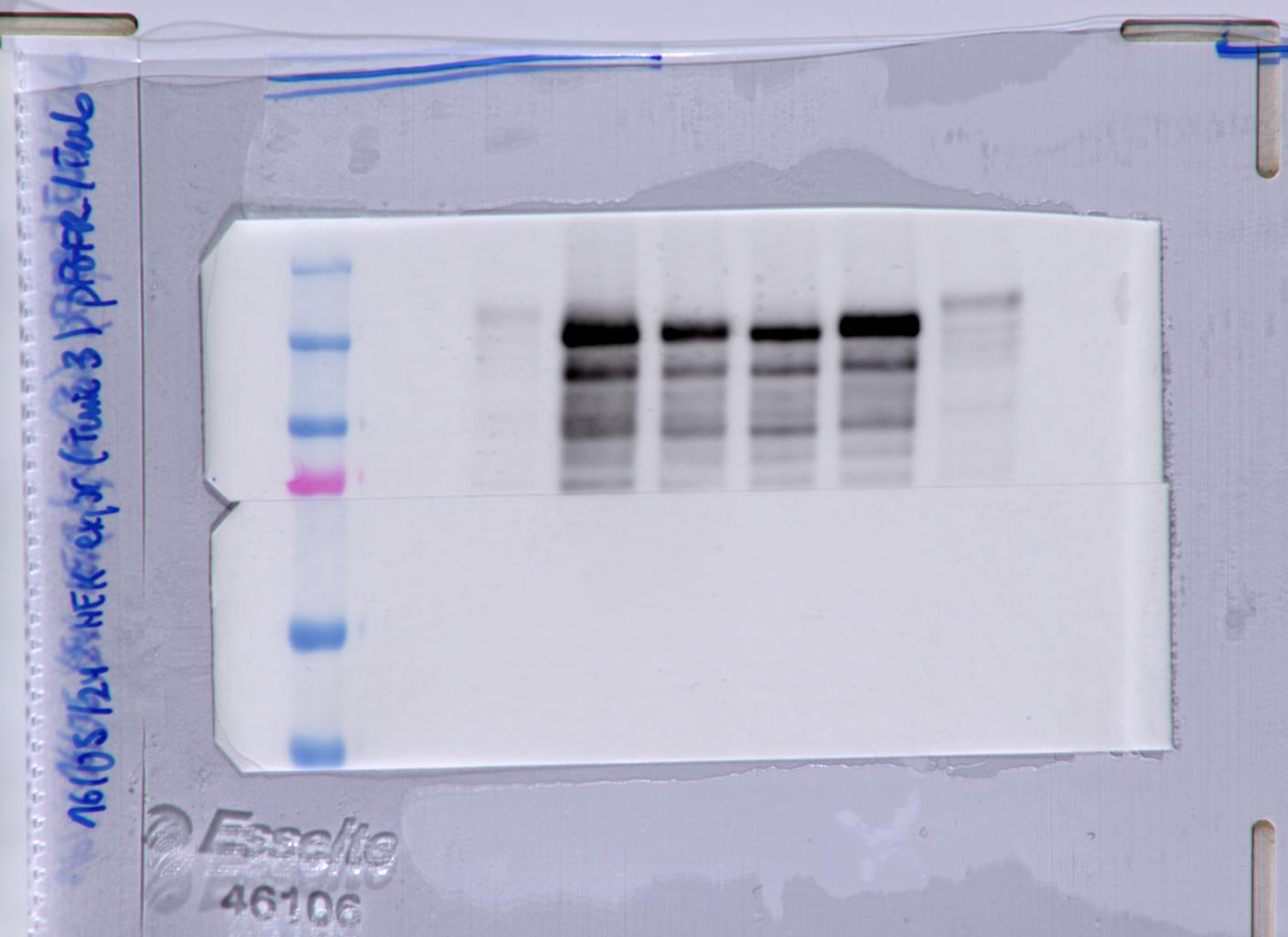

Supplement: Supplementary file 5 — Source data Fig. 2 [file 44318_2025_600_MOESM5_ESM.zip › Figure 2/2C/pFGFR1 original.jpg]

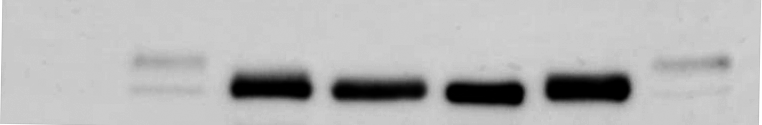

Supplement: Supplementary file 5 — Source data Fig. 2 [file 44318_2025_600_MOESM5_ESM.zip › Figure 2/2C/Fig 2C_FLAG.tif]

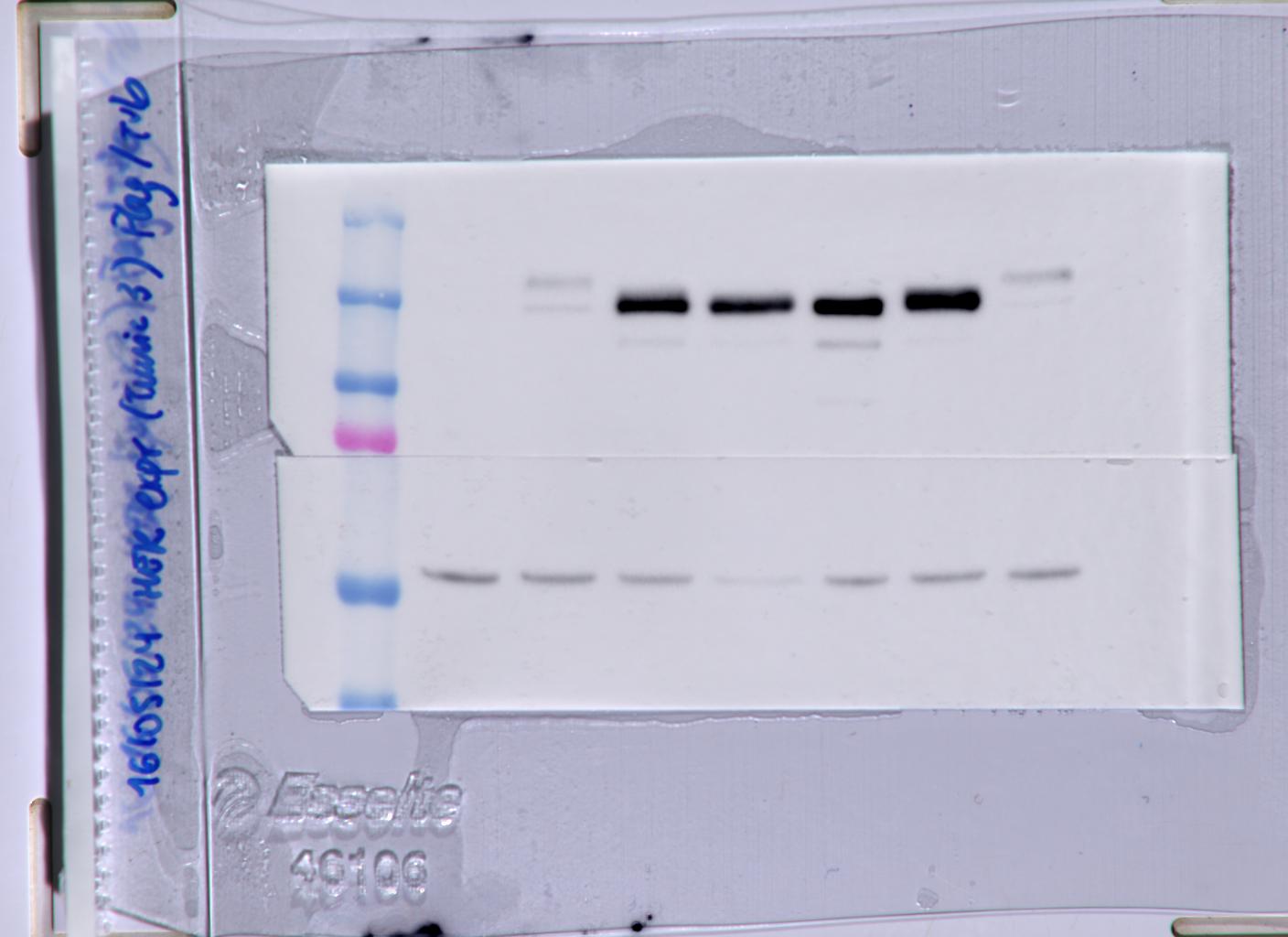

Supplement: Supplementary file 5 — Source data Fig. 2 [file 44318_2025_600_MOESM5_ESM.zip › Figure 2/2C/flag original.jpg]

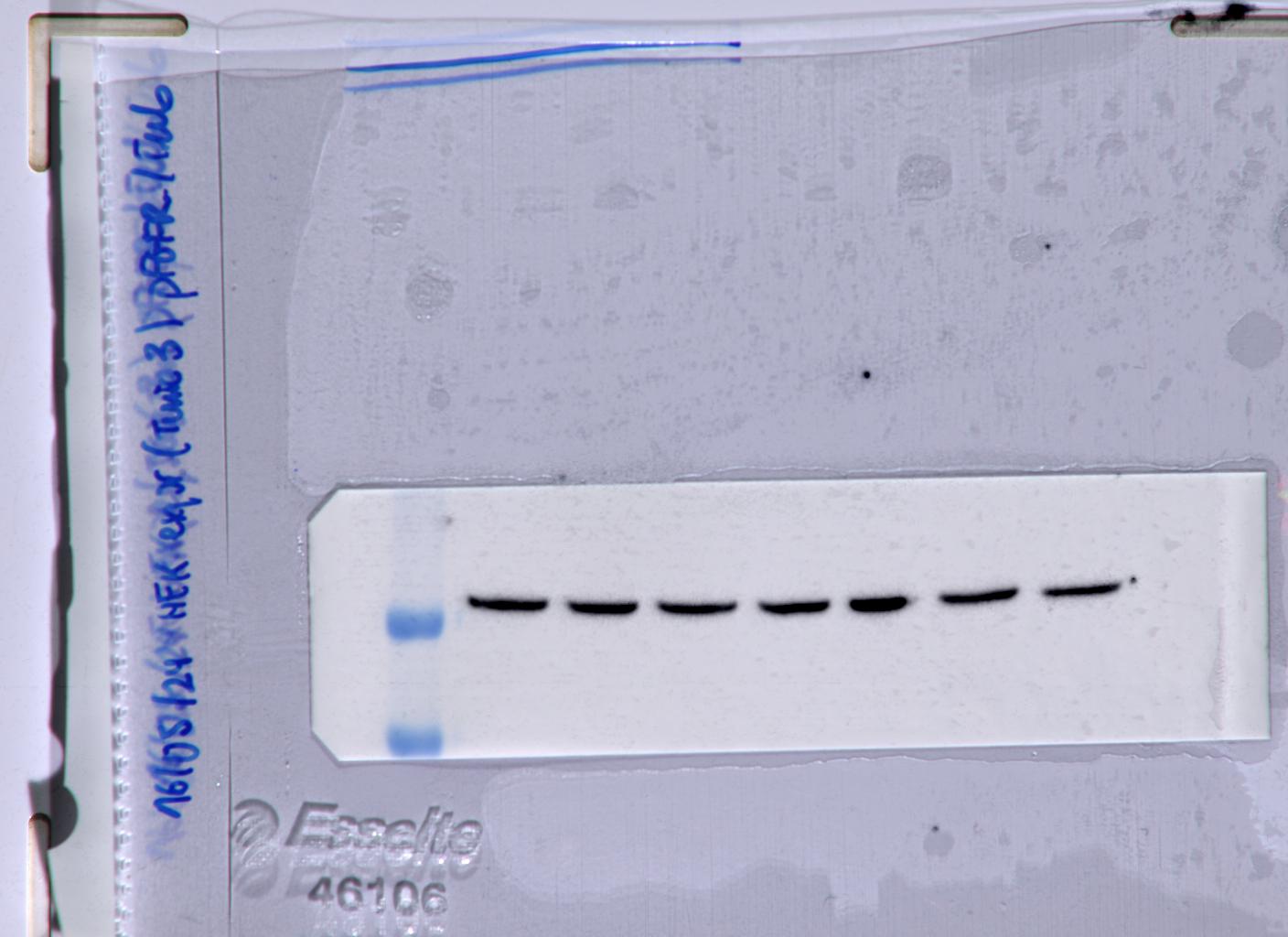

Supplement: Supplementary file 5 — Source data Fig. 2 [file 44318_2025_600_MOESM5_ESM.zip › Figure 2/2C/2C replicates/hek expr pfgftub 12s 2024.05.16_11.22.40_Ch+Marker.jpg]

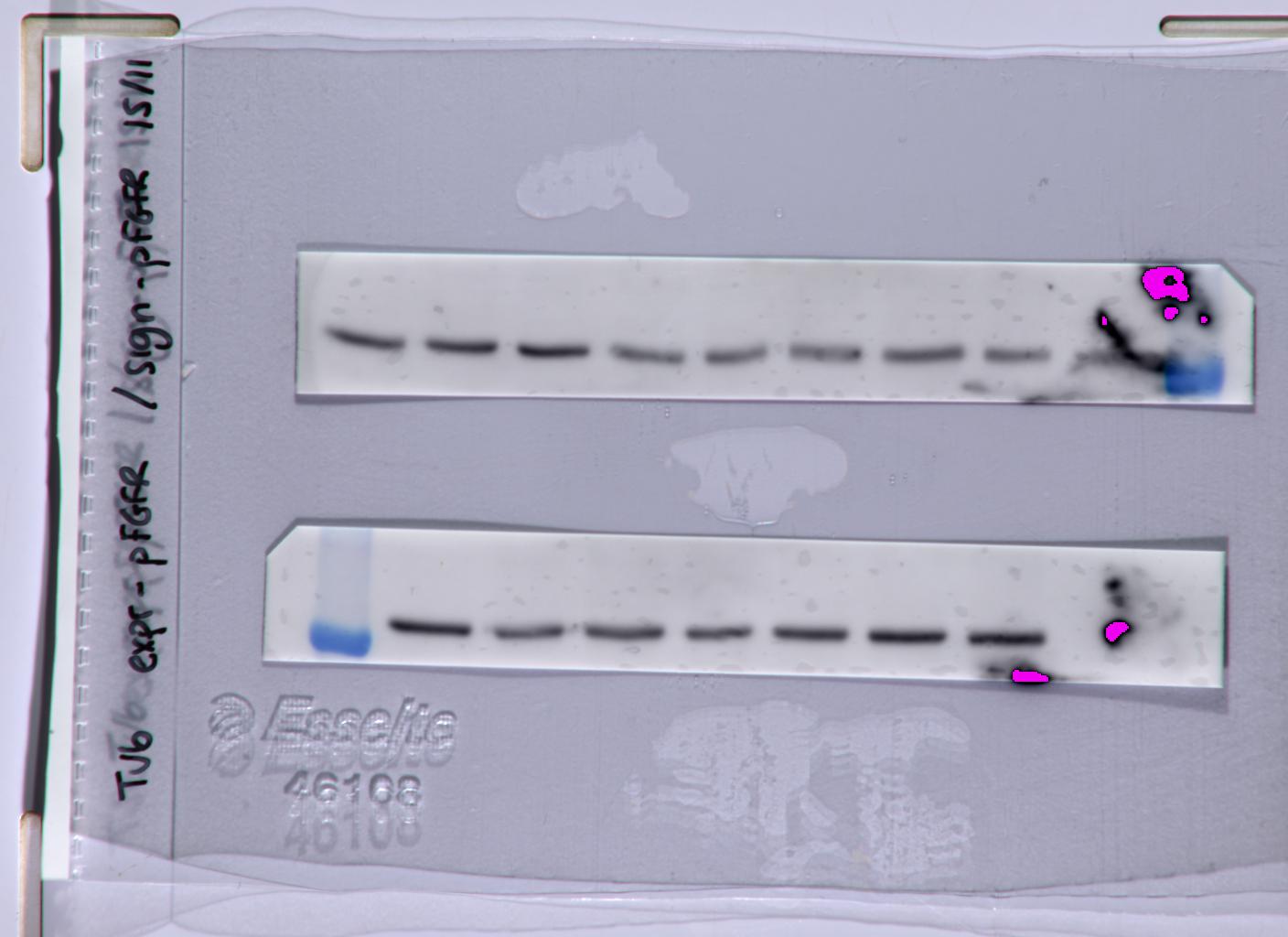

Supplement: Supplementary file 5 — Source data Fig. 2 [file 44318_2025_600_MOESM5_ESM.zip › Figure 2/2C/2C replicates/hek tubs expsign 1m 2023.11.15_12.44.51_Ch+Marker.jpg]

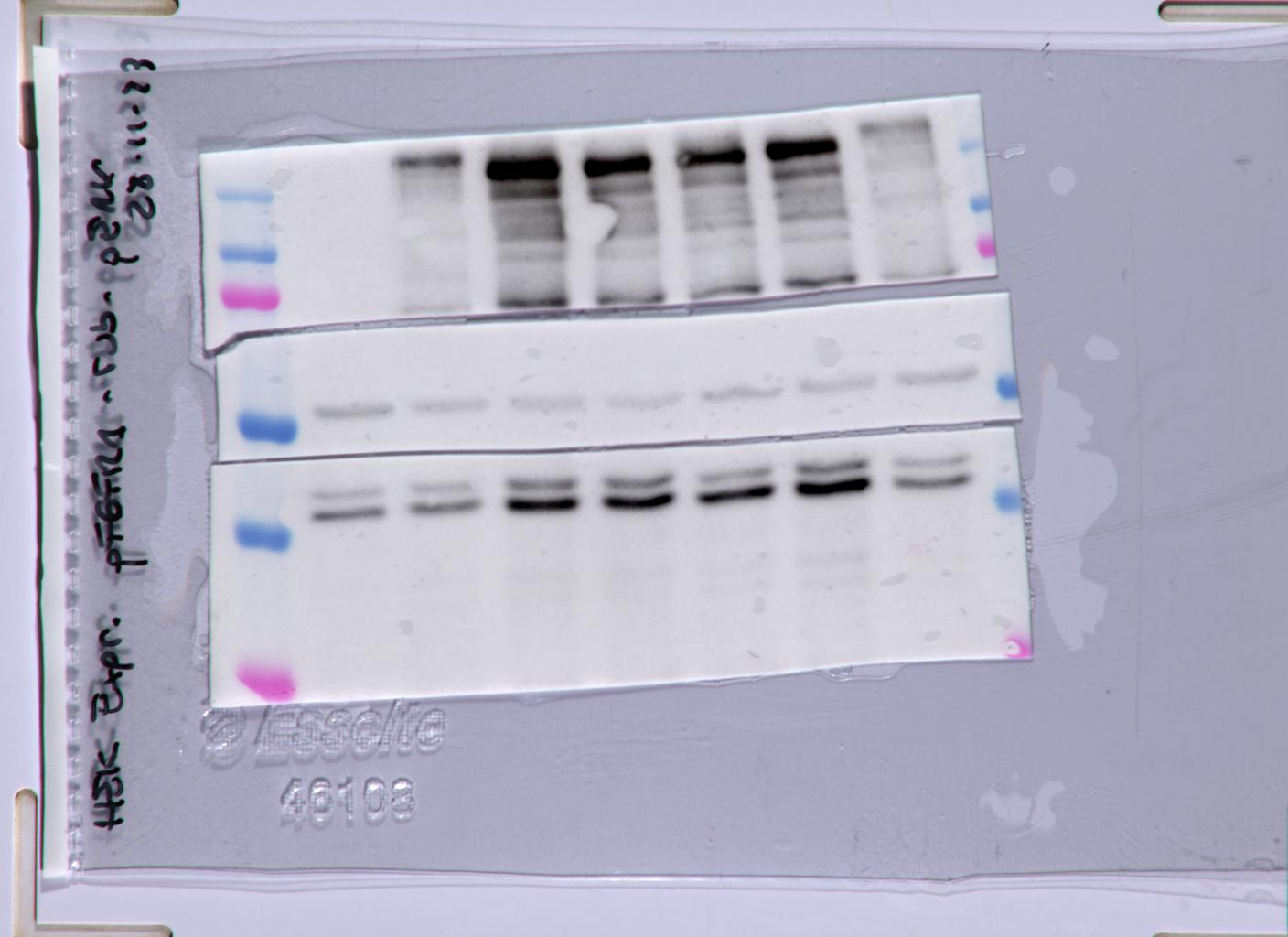

Supplement: Supplementary file 5 — Source data Fig. 2 [file 44318_2025_600_MOESM5_ESM.zip › Figure 2/2C/2C replicates/hekExp.pfgfrperk 0s 2023.11.28_16.11.01_Ch+Marker.jpg]

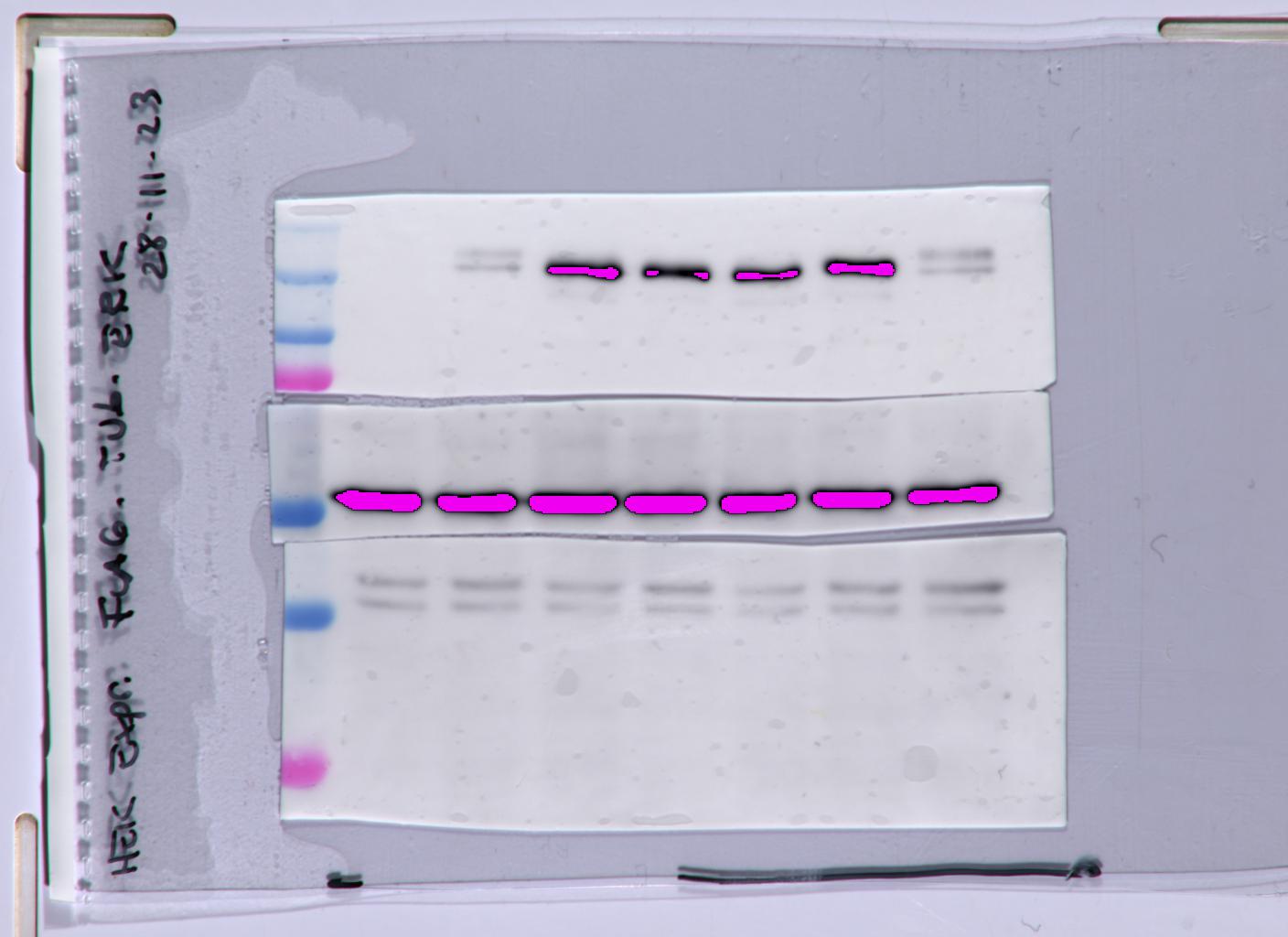

Supplement: Supplementary file 5 — Source data Fig. 2 [file 44318_2025_600_MOESM5_ESM.zip › Figure 2/2C/2C replicates/hekExp.flagErk 1m 2023.11.28_16.23.10_Ch+Marker.jpg]

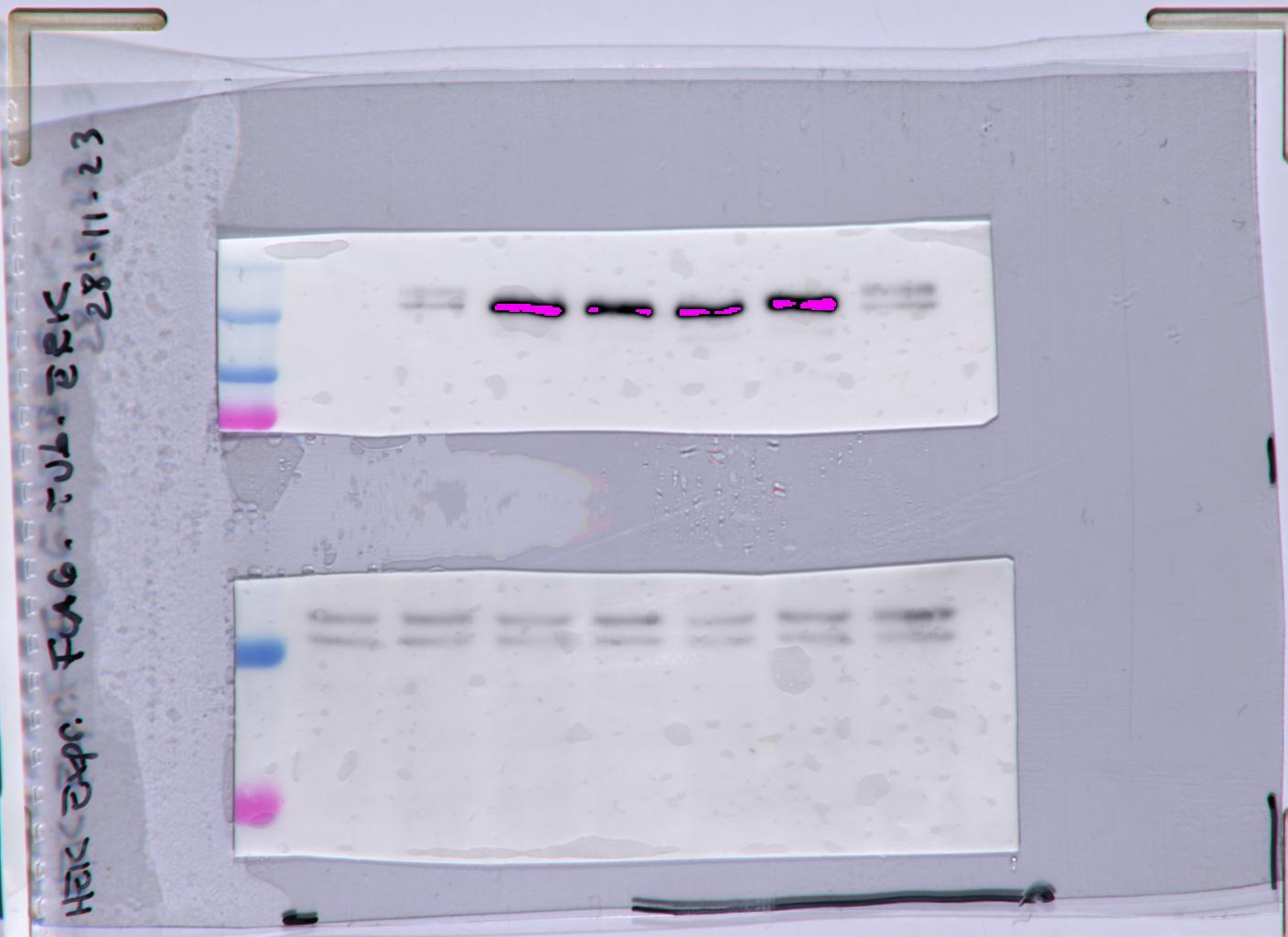

Supplement: Supplementary file 5 — Source data Fig. 2 [file 44318_2025_600_MOESM5_ESM.zip › Figure 2/2C/2C replicates/hekExp.flagErk 1mm 2023.11.28_16.25.52_Ch+Marker.jpg]

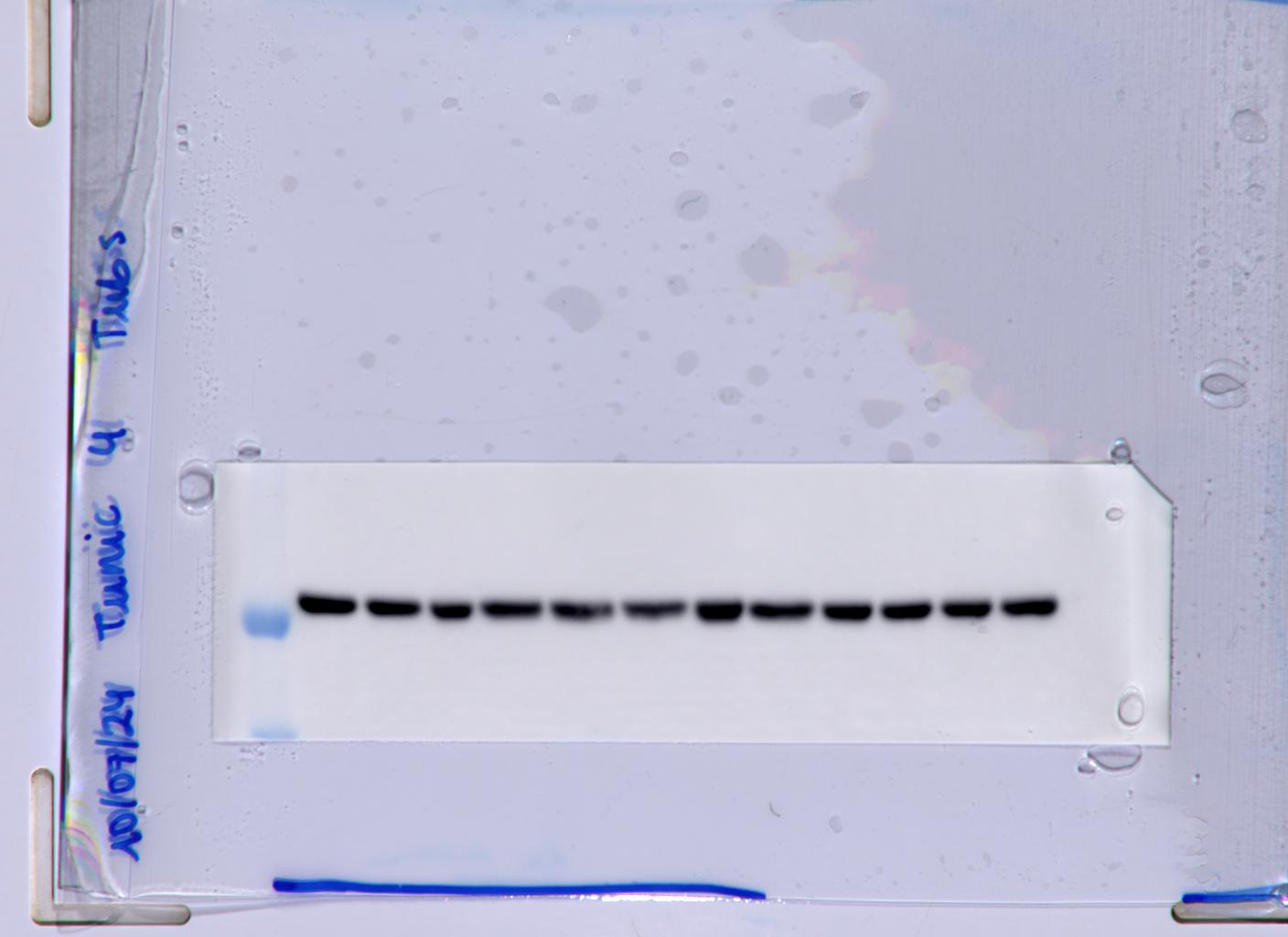

Supplement: Supplementary file 5 — Source data Fig. 2 [file 44318_2025_600_MOESM5_ESM.zip › Figure 2/2C/2C replicates/tunic 4 tub 11s 2024.07.10_11.01.20_Ch+Marker.jpg]

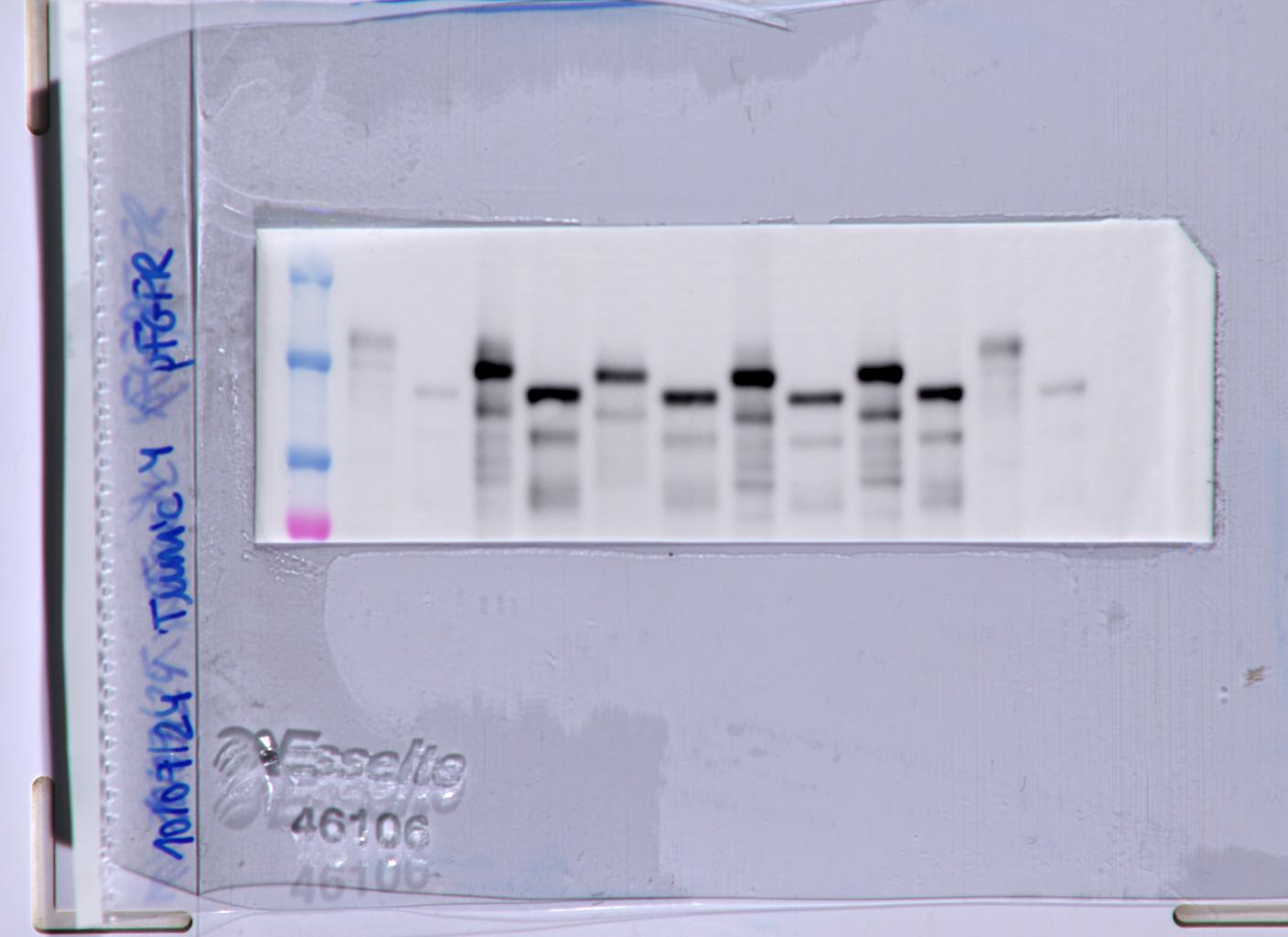

Supplement: Supplementary file 5 — Source data Fig. 2 [file 44318_2025_600_MOESM5_ESM.zip › Figure 2/2C/2C replicates/tunic 4 pfgfr 2024.07.10_10.45.15_Ch+Marker.jpg]

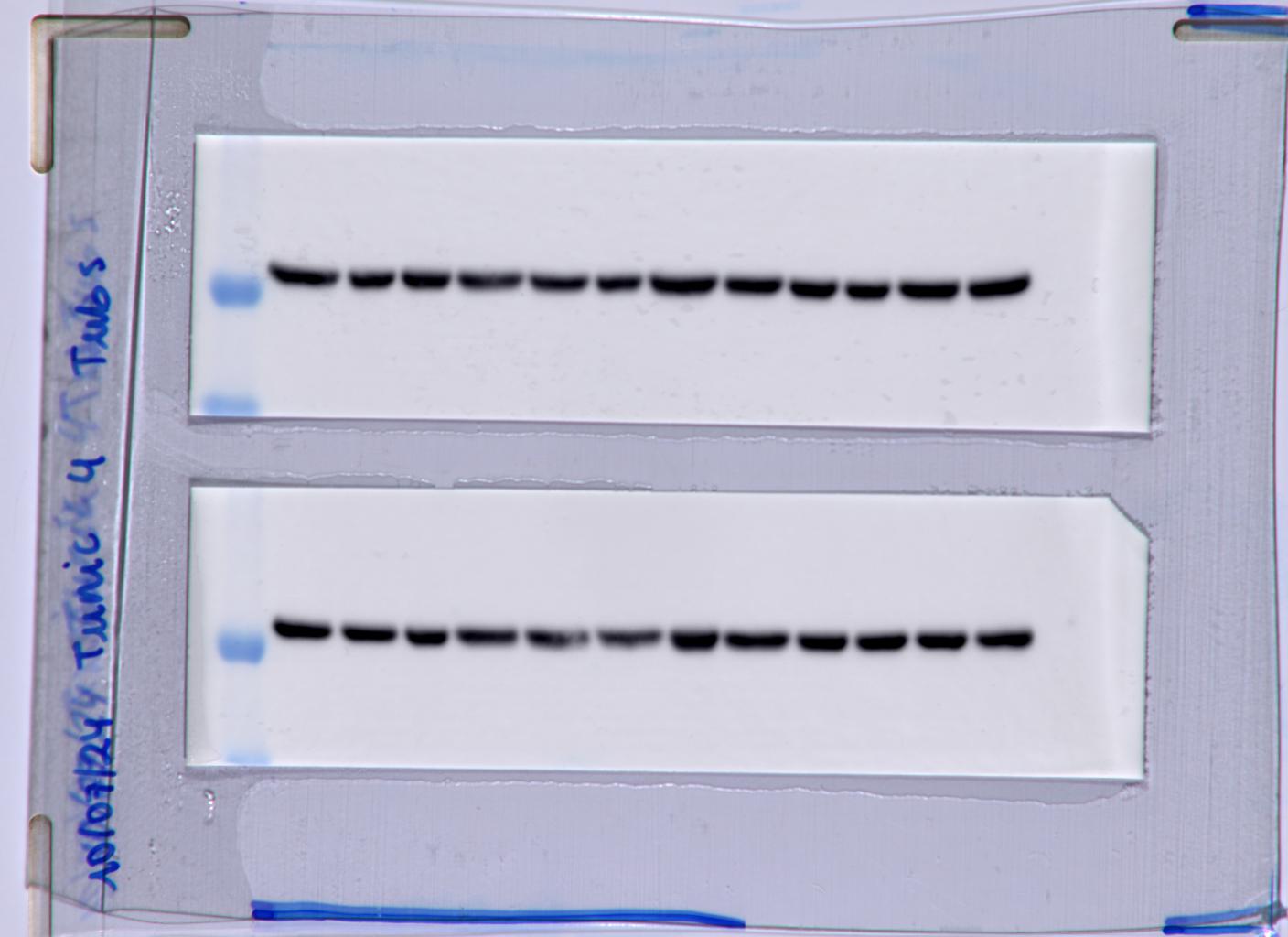

Supplement: Supplementary file 5 — Source data Fig. 2 [file 44318_2025_600_MOESM5_ESM.zip › Figure 2/2C/2C replicates/tunic 4 tub 5s 2024.07.10_10.54.17_Ch+Marker.jpg]

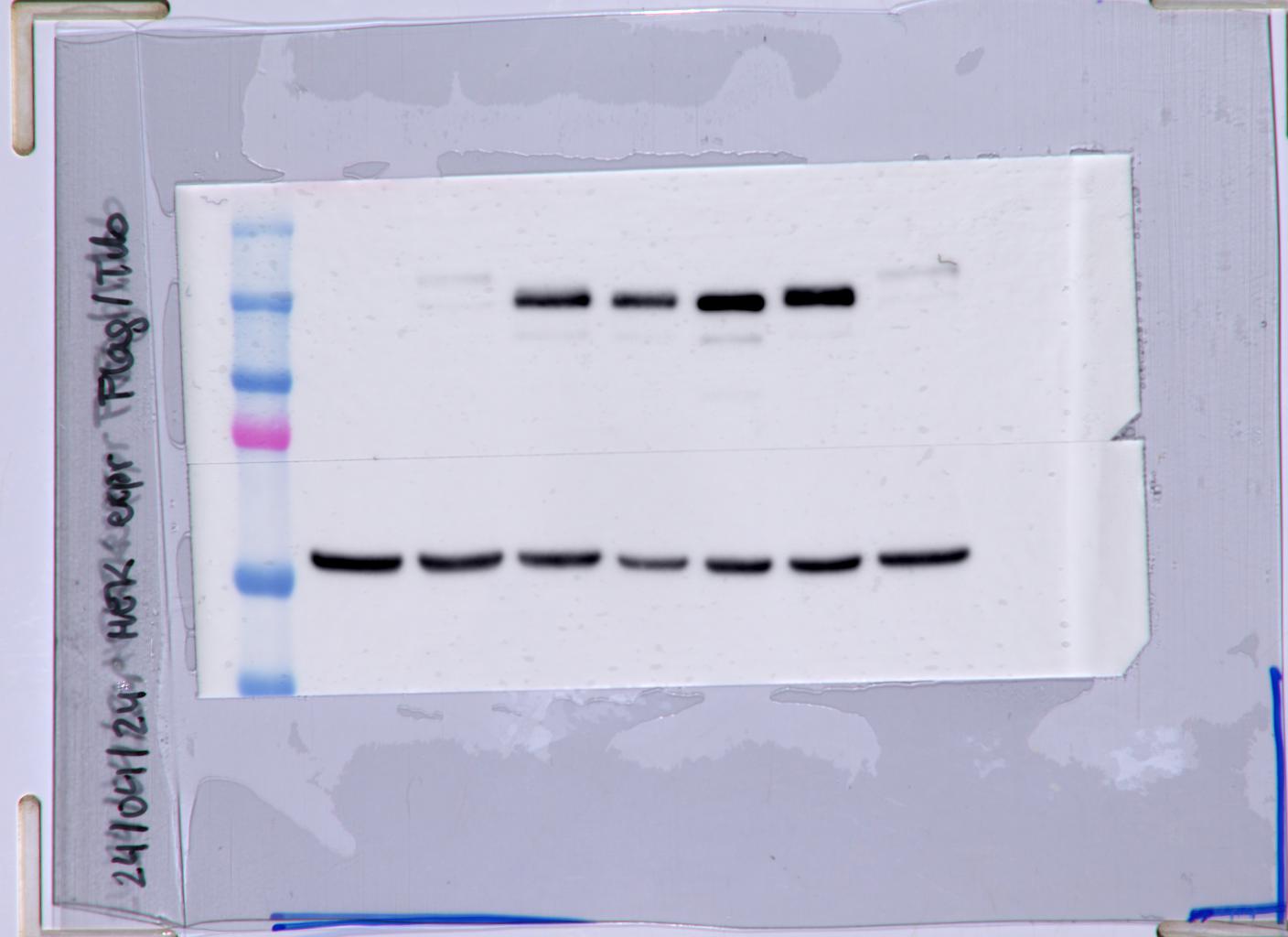

Supplement: Supplementary file 5 — Source data Fig. 2 [file 44318_2025_600_MOESM5_ESM.zip › Figure 2/2C/2C replicates/expr flagtub 8s 2024.04.24_11.15.34_Ch+Marker.jpg]

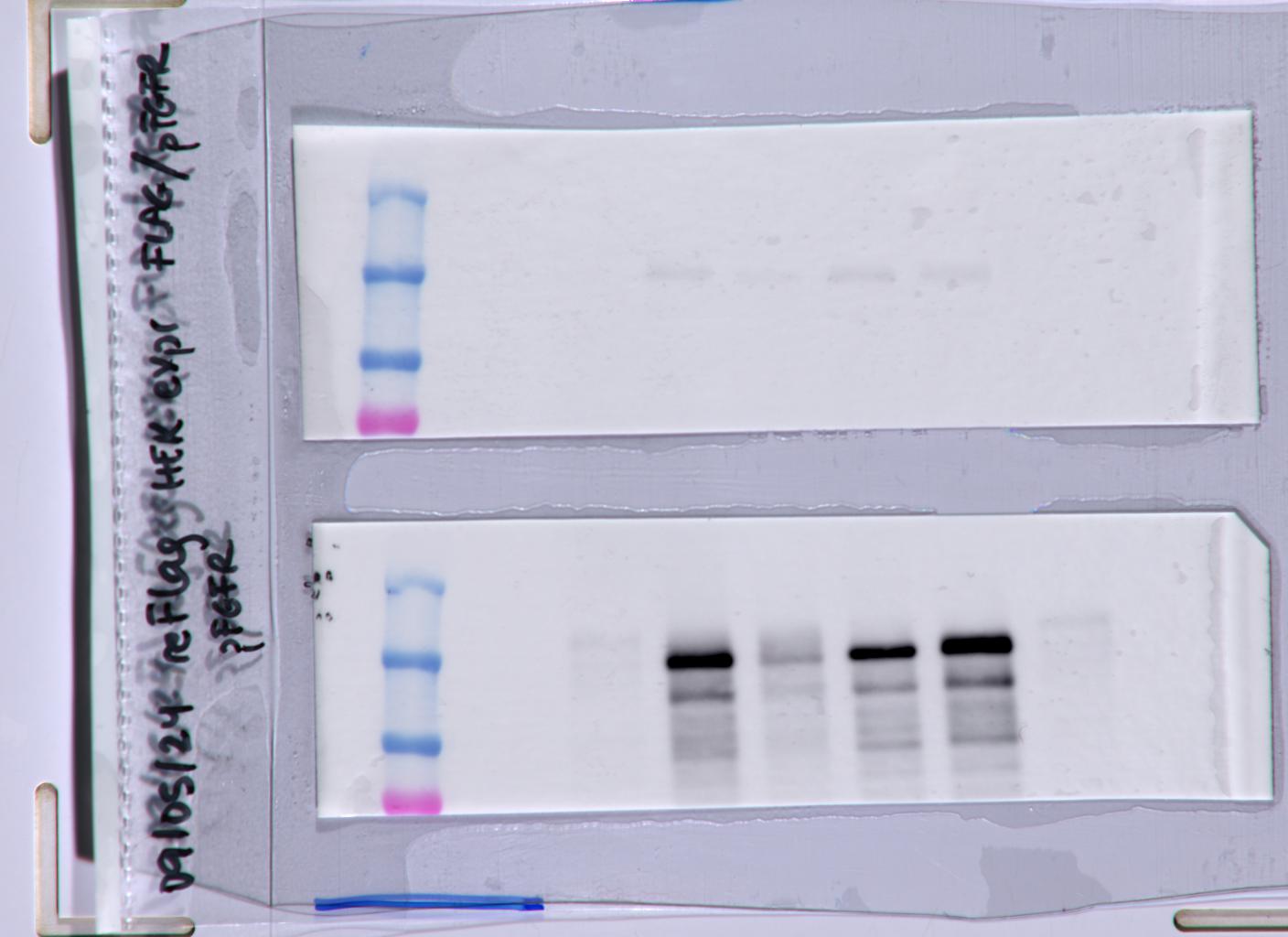

Supplement: Supplementary file 5 — Source data Fig. 2 [file 44318_2025_600_MOESM5_ESM.zip › Figure 2/2C/2C replicates/re expr flagfosf 0.3 2024.05.09_14.08.32_Ch+Marker.jpg]

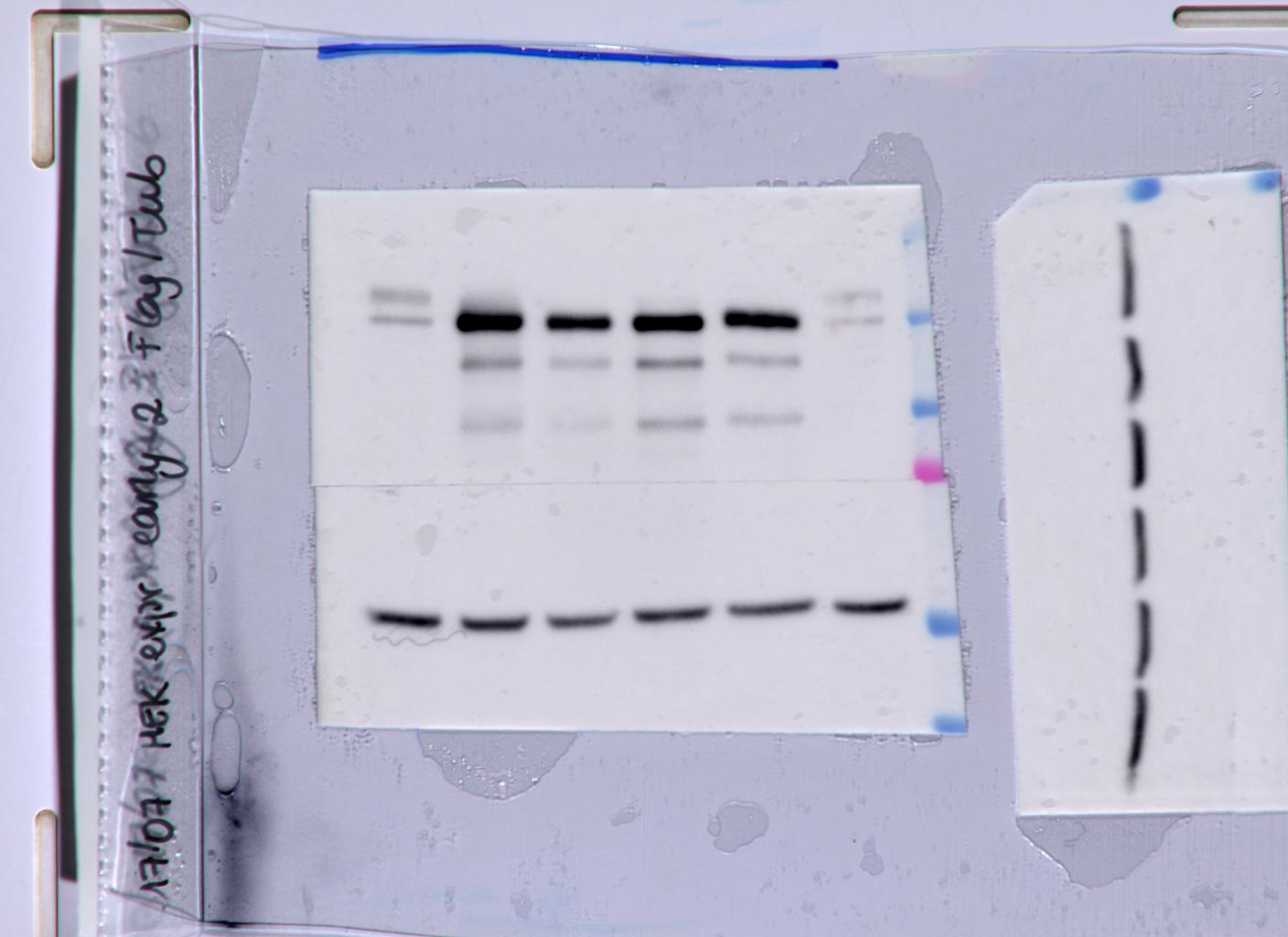

Supplement: Supplementary file 5 — Source data Fig. 2 [file 44318_2025_600_MOESM5_ESM.zip › Figure 2/2C/2C replicates/early2exp flagtub 3s 2024.07.17_14.46.43_Ch+Marker.jpg]

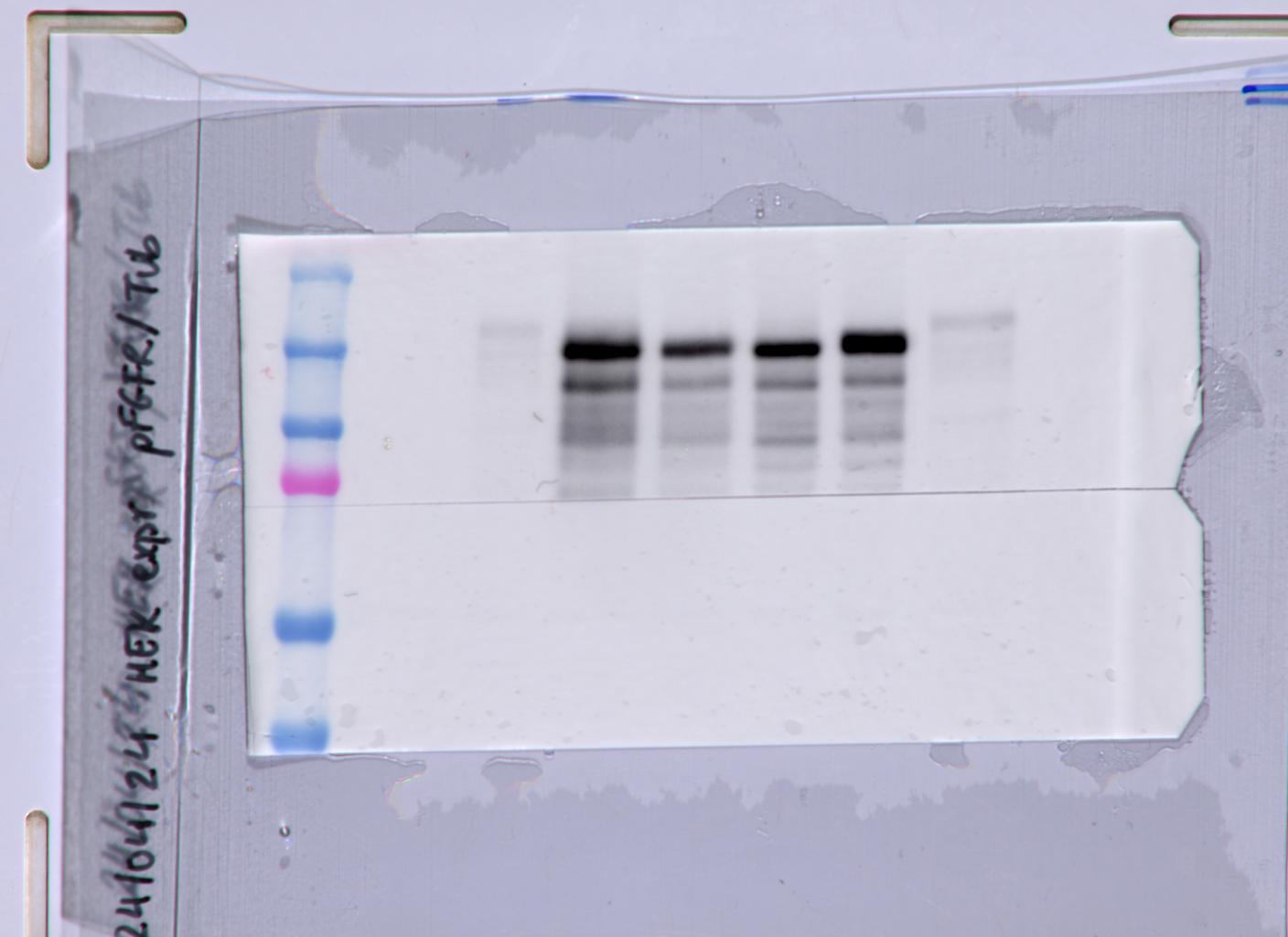

Supplement: Supplementary file 5 — Source data Fig. 2 [file 44318_2025_600_MOESM5_ESM.zip › Figure 2/2C/2C replicates/expr pfgfrtub 1s 2024.04.24_11.01.06_Ch+Marker.jpg]

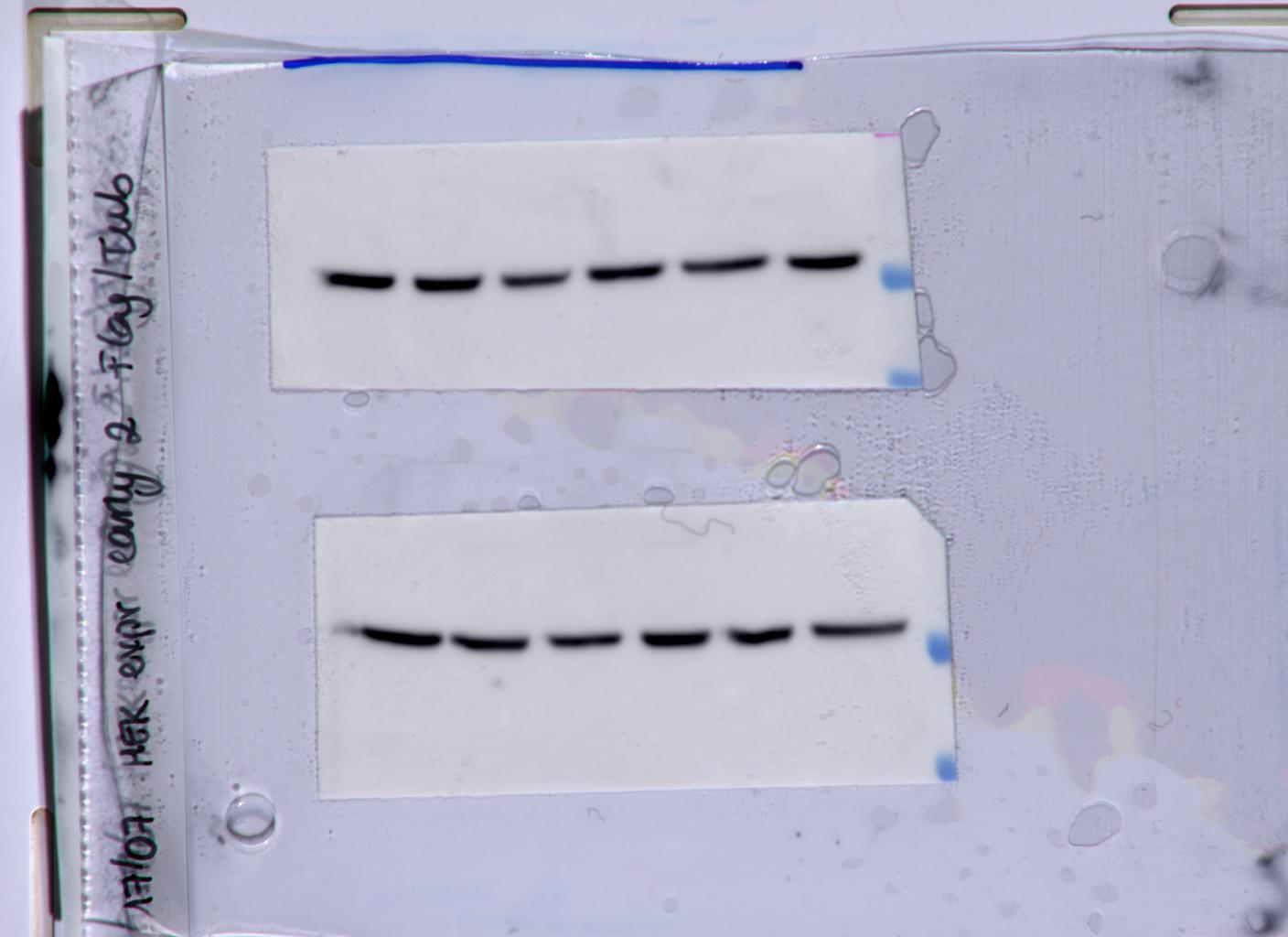

Supplement: Supplementary file 5 — Source data Fig. 2 [file 44318_2025_600_MOESM5_ESM.zip › Figure 2/2C/2C replicates/hekexpr early tub 31 2024.07.17_14.56.14_Ch+Marker.jpg]

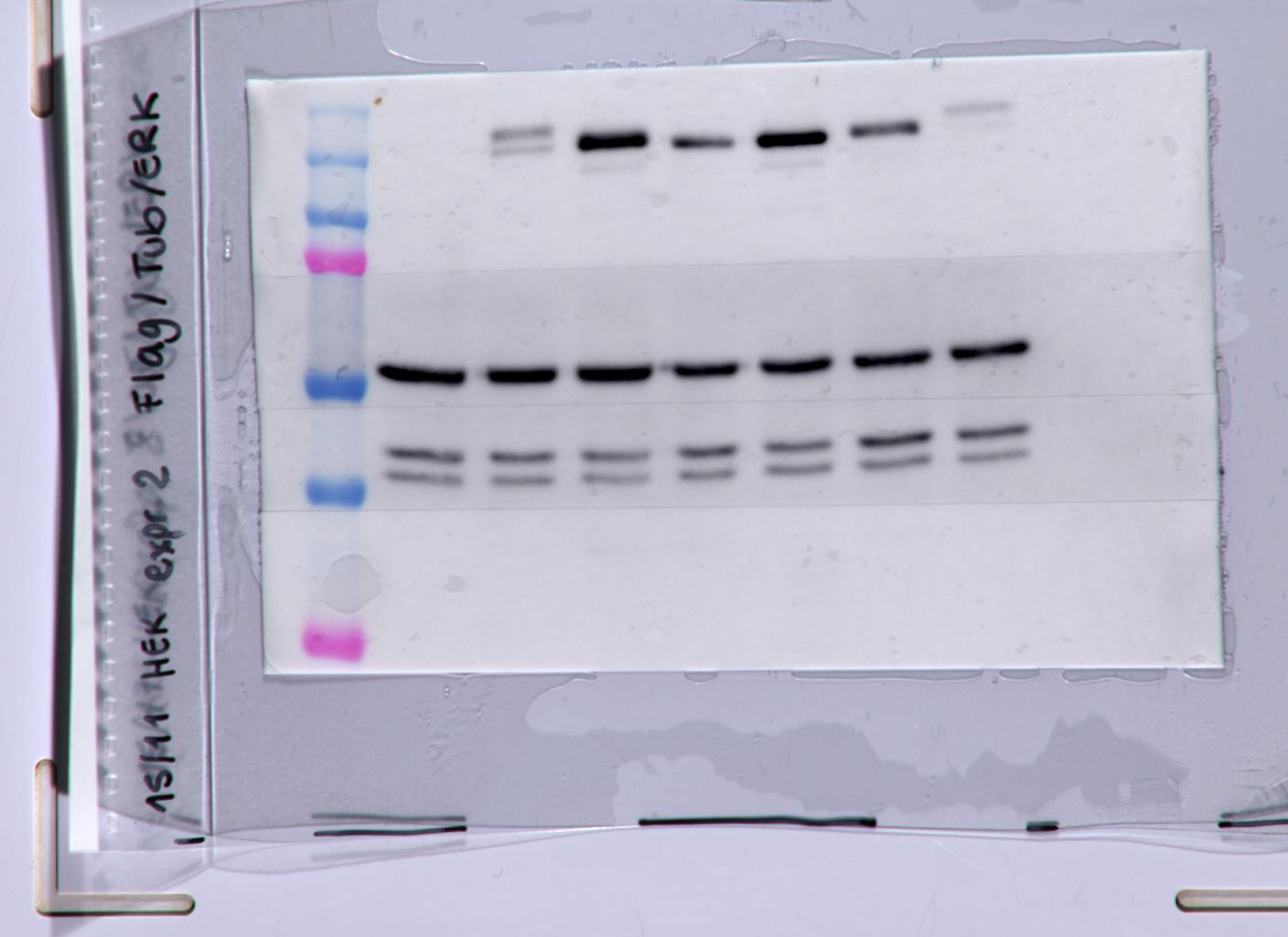

Supplement: Supplementary file 5 — Source data Fig. 2 [file 44318_2025_600_MOESM5_ESM.zip › Figure 2/2C/2C replicates/hek expr flag 5s 2023.11.15_12.29.37_Ch+Marker.jpg]

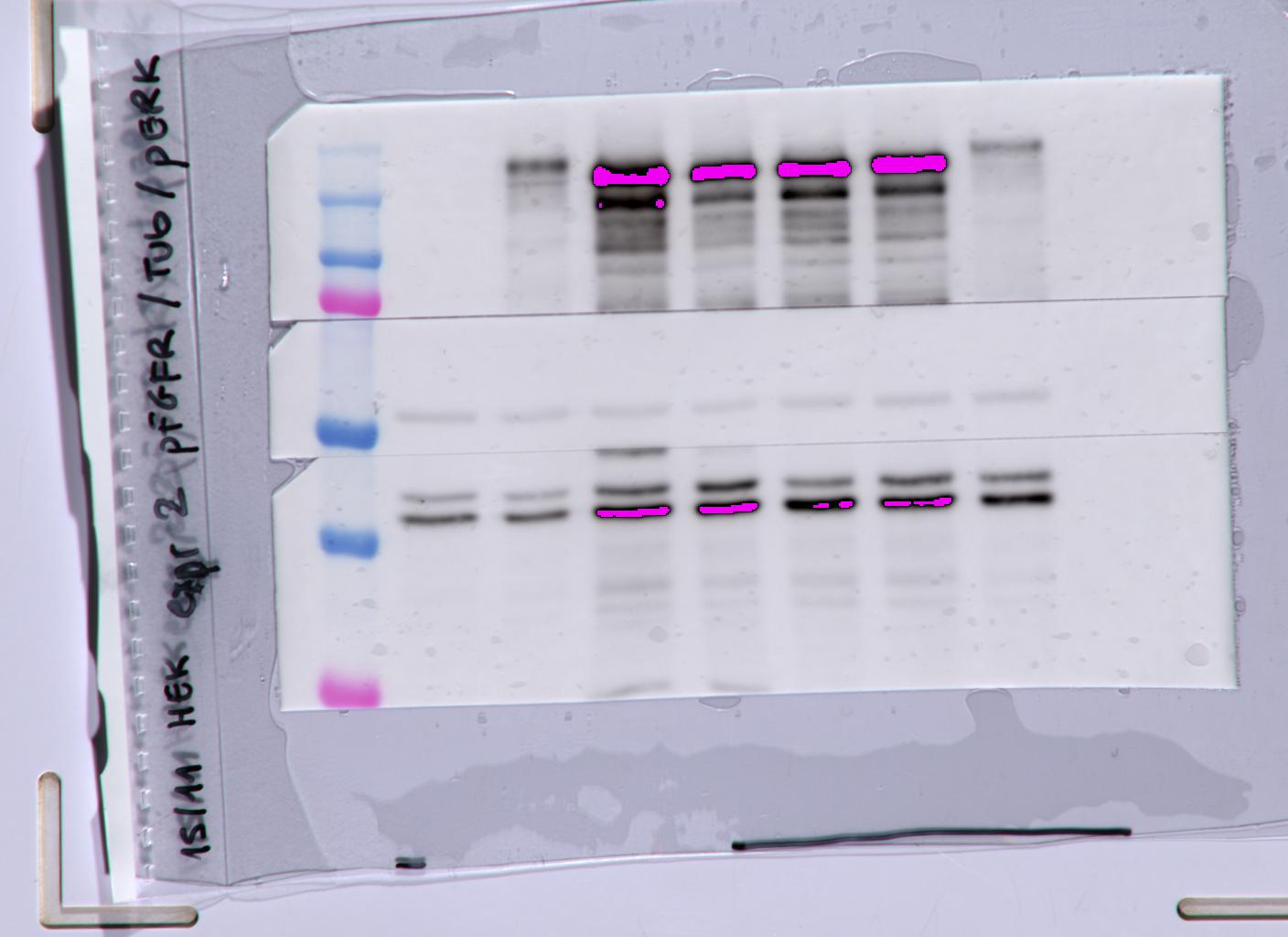

Supplement: Supplementary file 5 — Source data Fig. 2 [file 44318_2025_600_MOESM5_ESM.zip › Figure 2/2C/2C replicates/hek expr2 pfgfr 5s 2023.11.15_12.19.51_Ch+Marker.jpg]

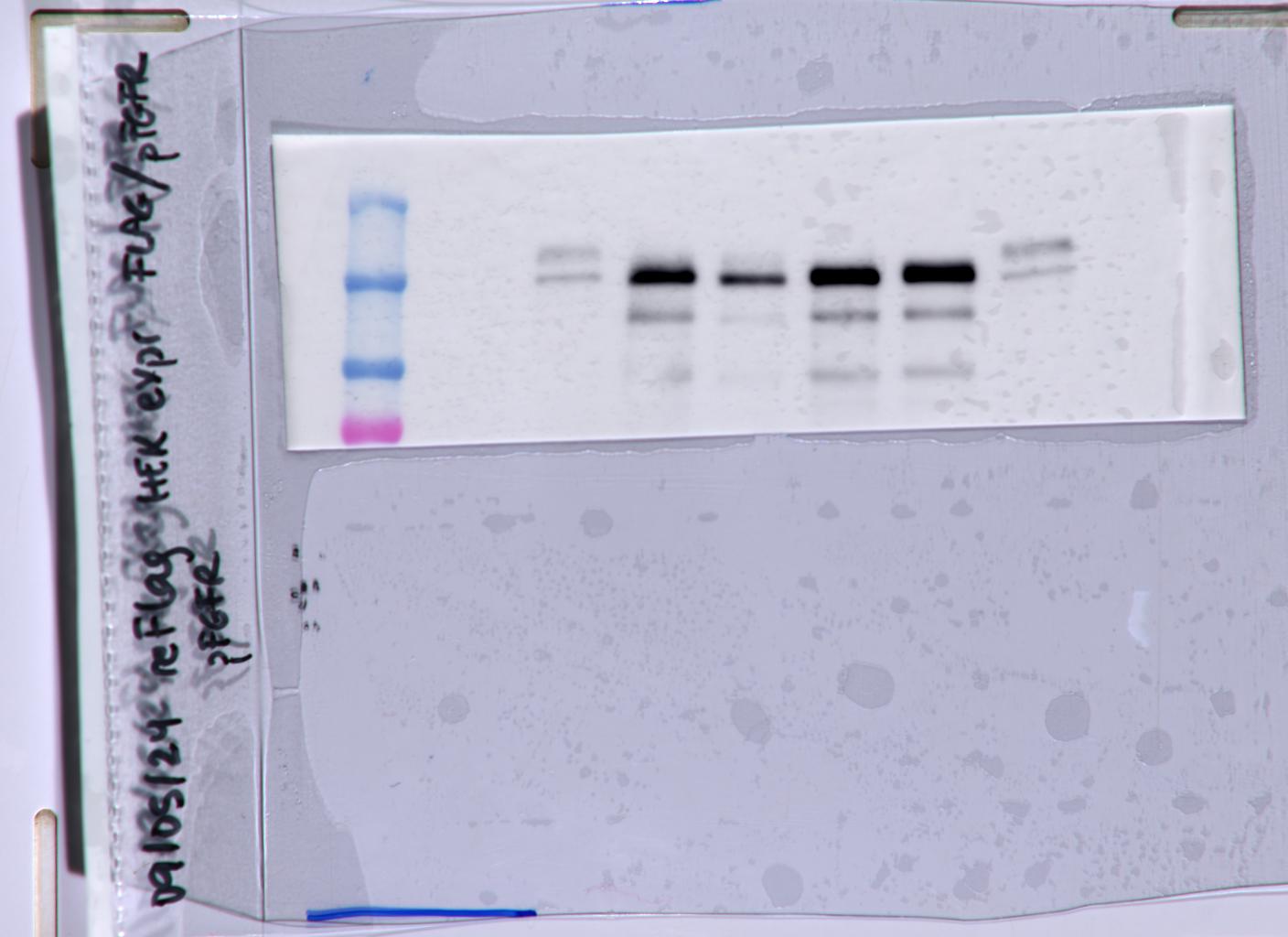

Supplement: Supplementary file 5 — Source data Fig. 2 [file 44318_2025_600_MOESM5_ESM.zip › Figure 2/2C/2C replicates/re expr flag 10s 2024.05.09_14.15.05_Ch+Marker.jpg]

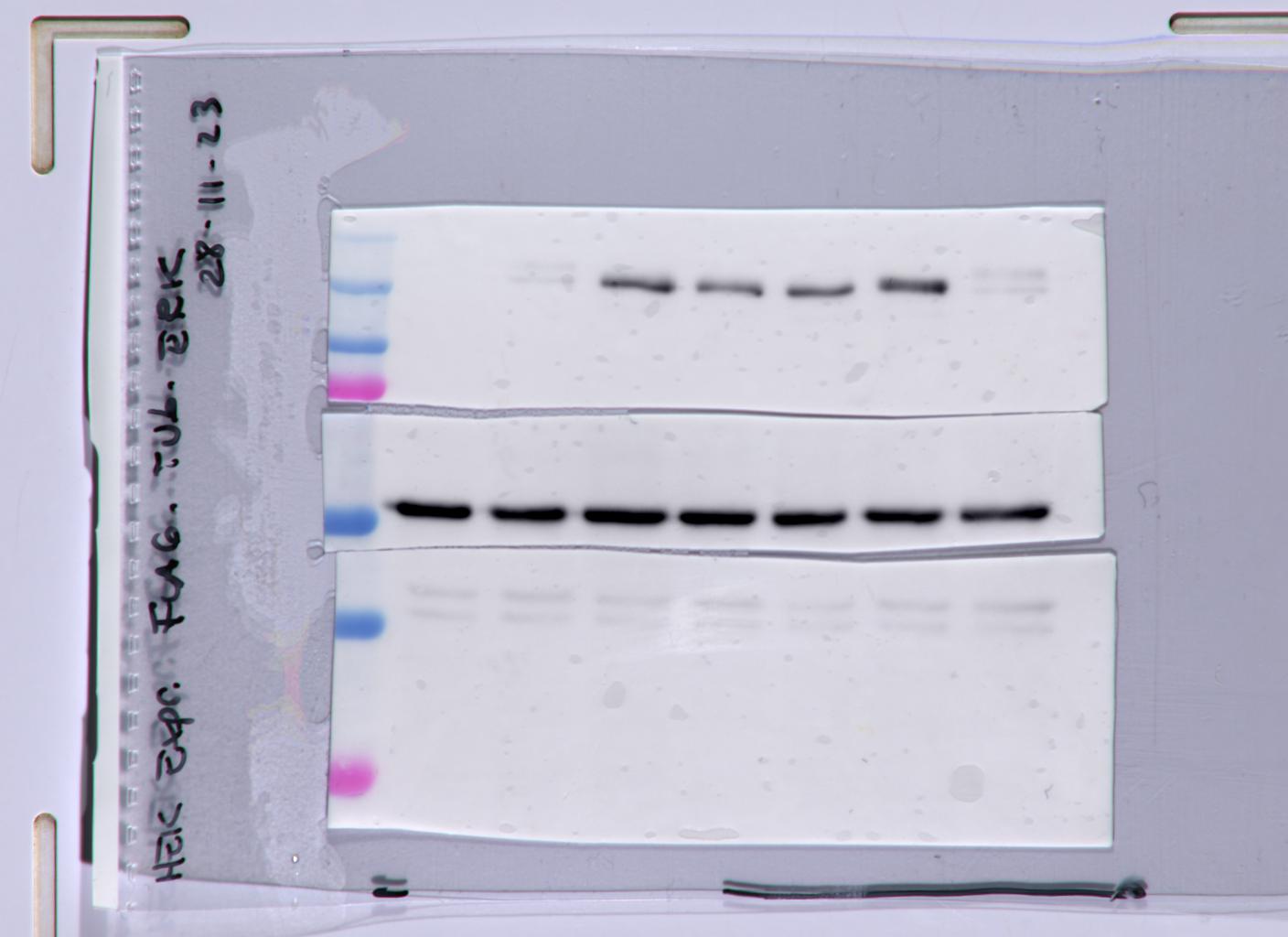

Supplement: Supplementary file 5 — Source data Fig. 2 [file 44318_2025_600_MOESM5_ESM.zip › Figure 2/2C/2C replicates/hekExp.flagtubErk 5s 2023.11.28_16.12.27_Ch+Marker.jpg]

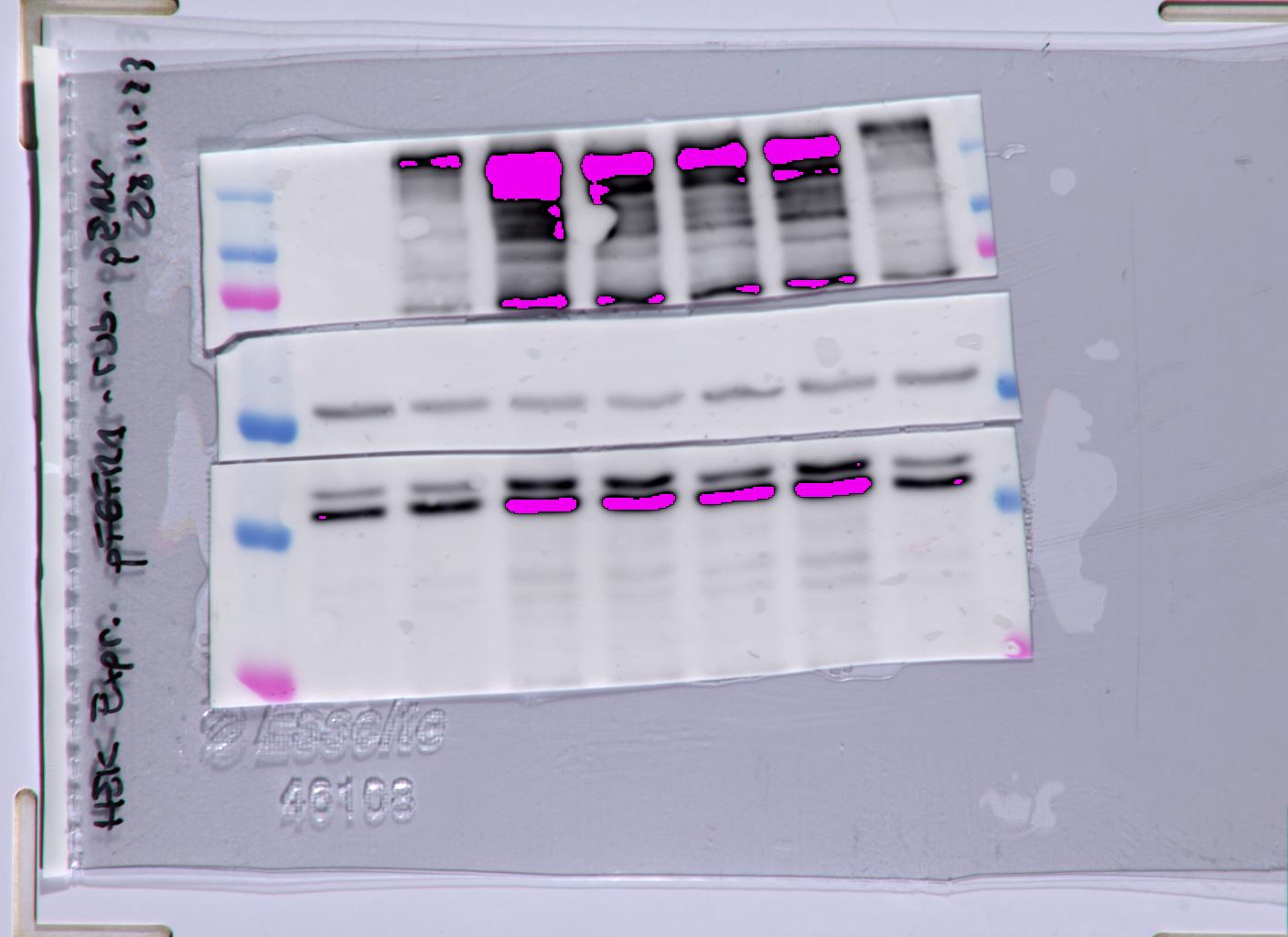

Supplement: Supplementary file 5 — Source data Fig. 2 [file 44318_2025_600_MOESM5_ESM.zip › Figure 2/2C/2C replicates/hekExp.pfgfrperk 5s 2023.11.28_16.09.30_Ch+Marker.jpg]

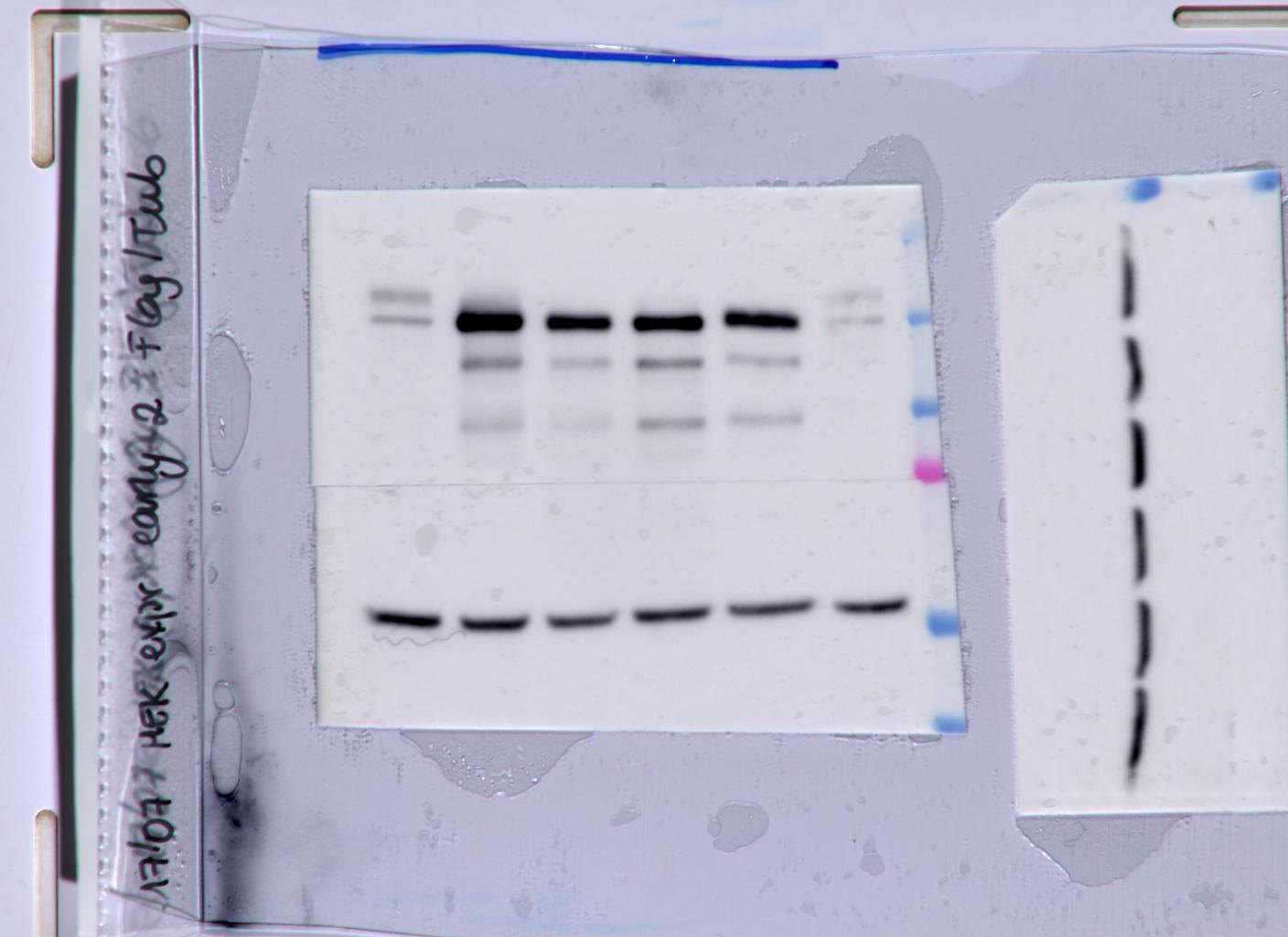

Supplement: Supplementary file 5 — Source data Fig. 2 [file 44318_2025_600_MOESM5_ESM.zip › Figure 2/2C/2C replicates/early2exp flagtub 5s 2024.07.17_14.47.55_Ch+Marker.jpg]

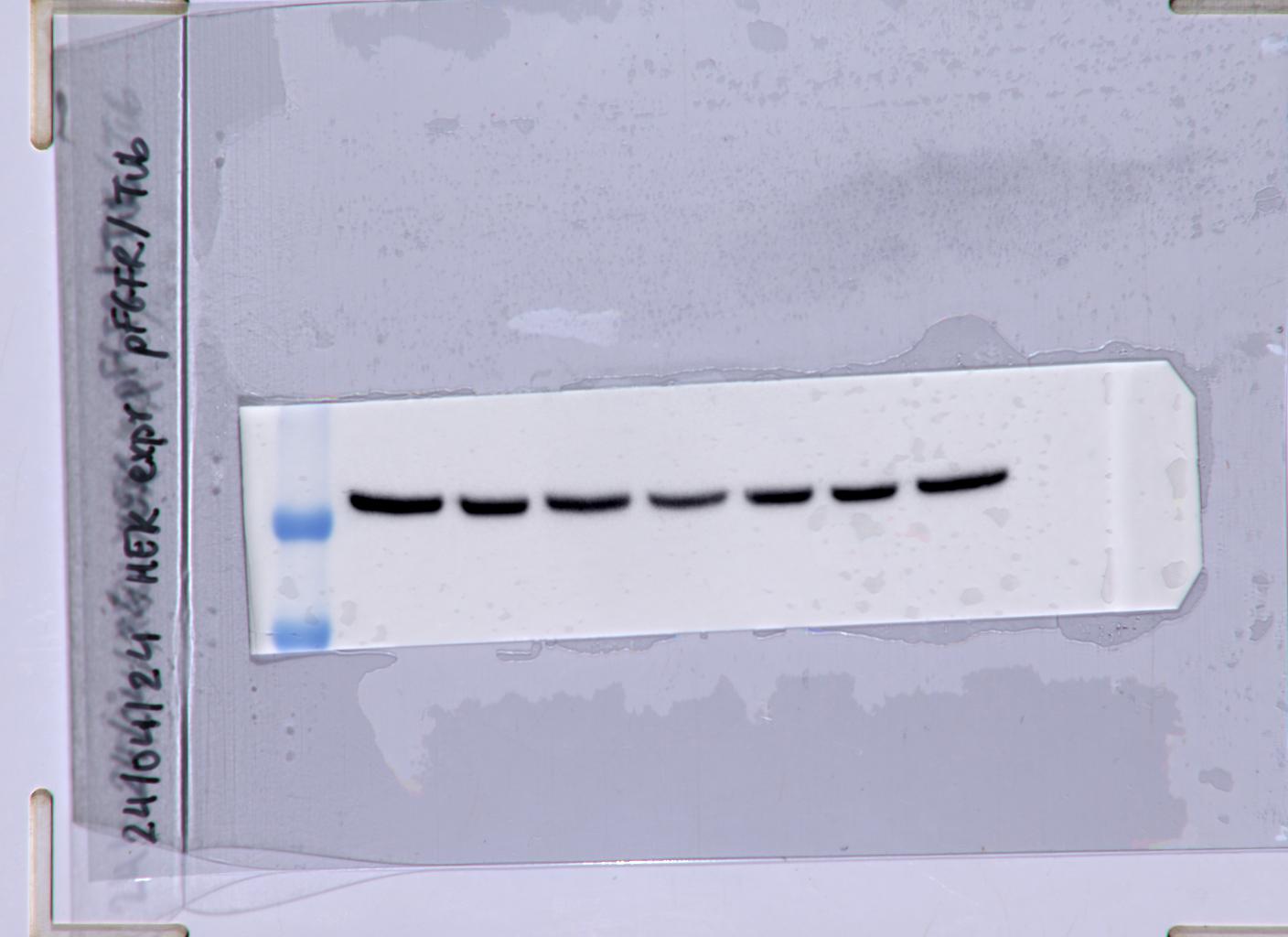

Supplement: Supplementary file 5 — Source data Fig. 2 [file 44318_2025_600_MOESM5_ESM.zip › Figure 2/2C/2C replicates/expr pfgfrtub 2.5s 2024.04.24_11.05.17_Ch+Marker.jpg]

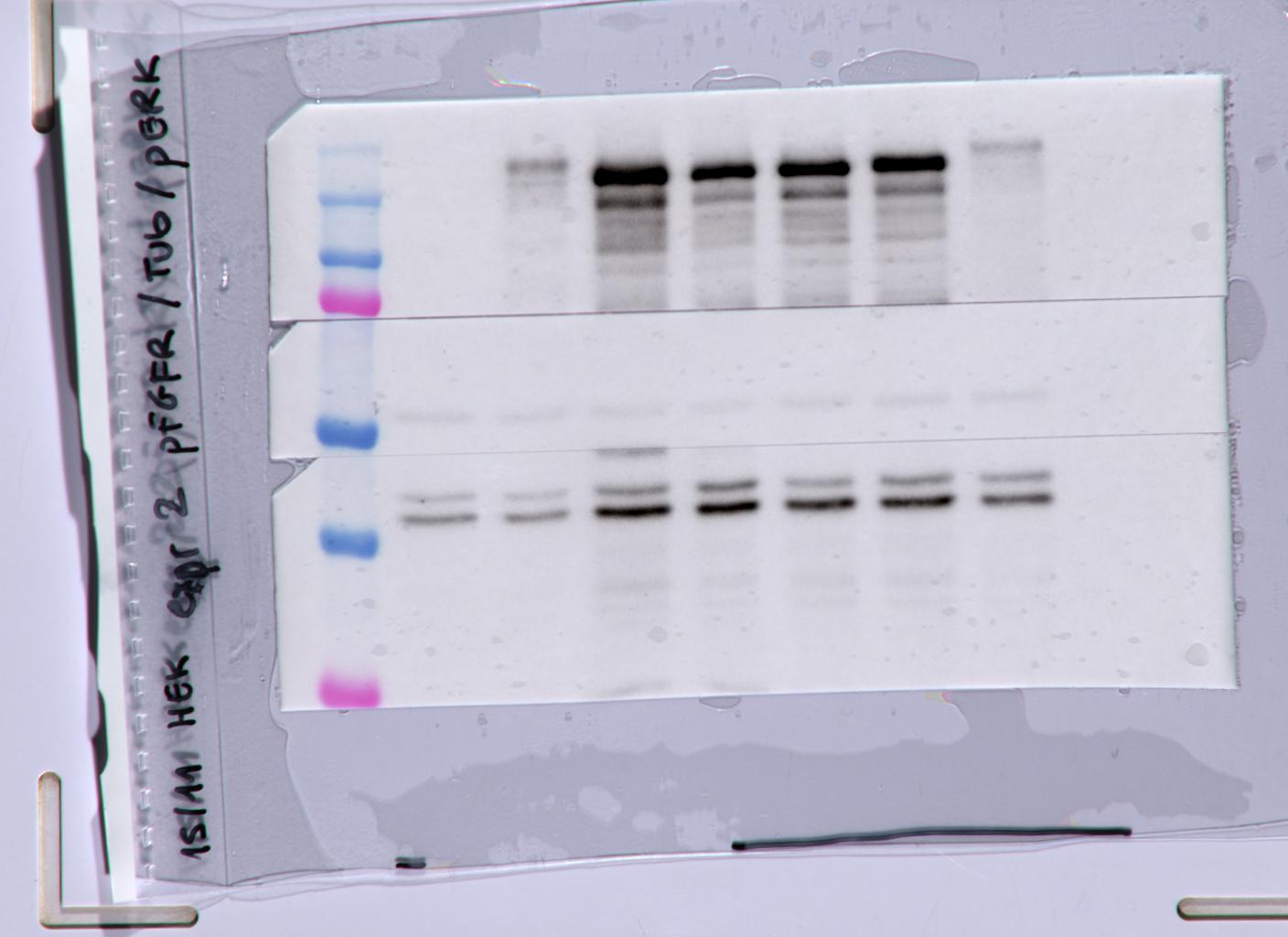

Supplement: Supplementary file 5 — Source data Fig. 2 [file 44318_2025_600_MOESM5_ESM.zip › Figure 2/2C/2C replicates/hek expr2 pfgfr 0.1s 2023.11.15_12.15.45_Ch+Marker.jpg]

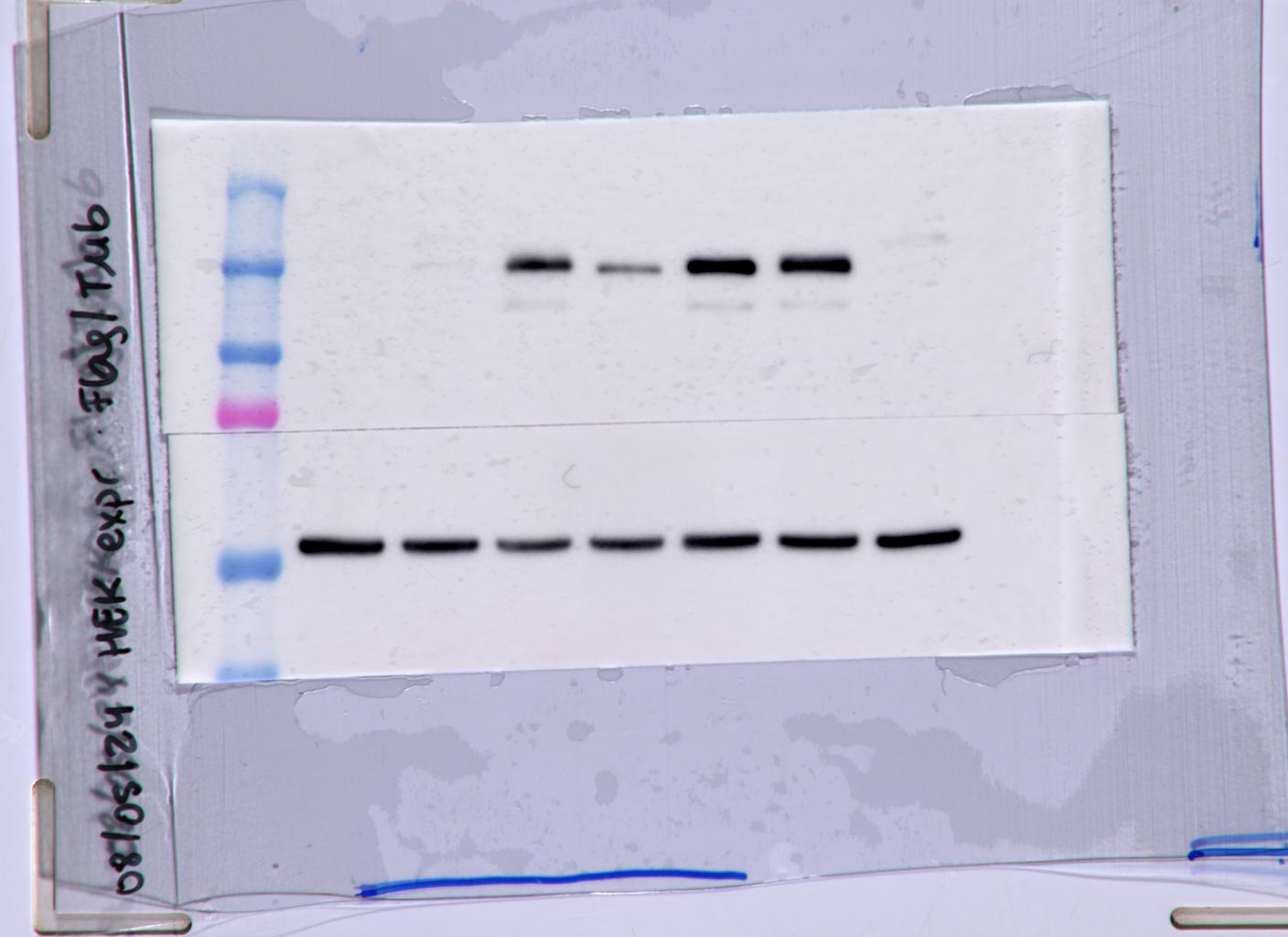

Supplement: Supplementary file 5 — Source data Fig. 2 [file 44318_2025_600_MOESM5_ESM.zip › Figure 2/2C/2C replicates/hekexpr flag 5s 2024.05.08_11.23.37_Ch+Marker.jpg]

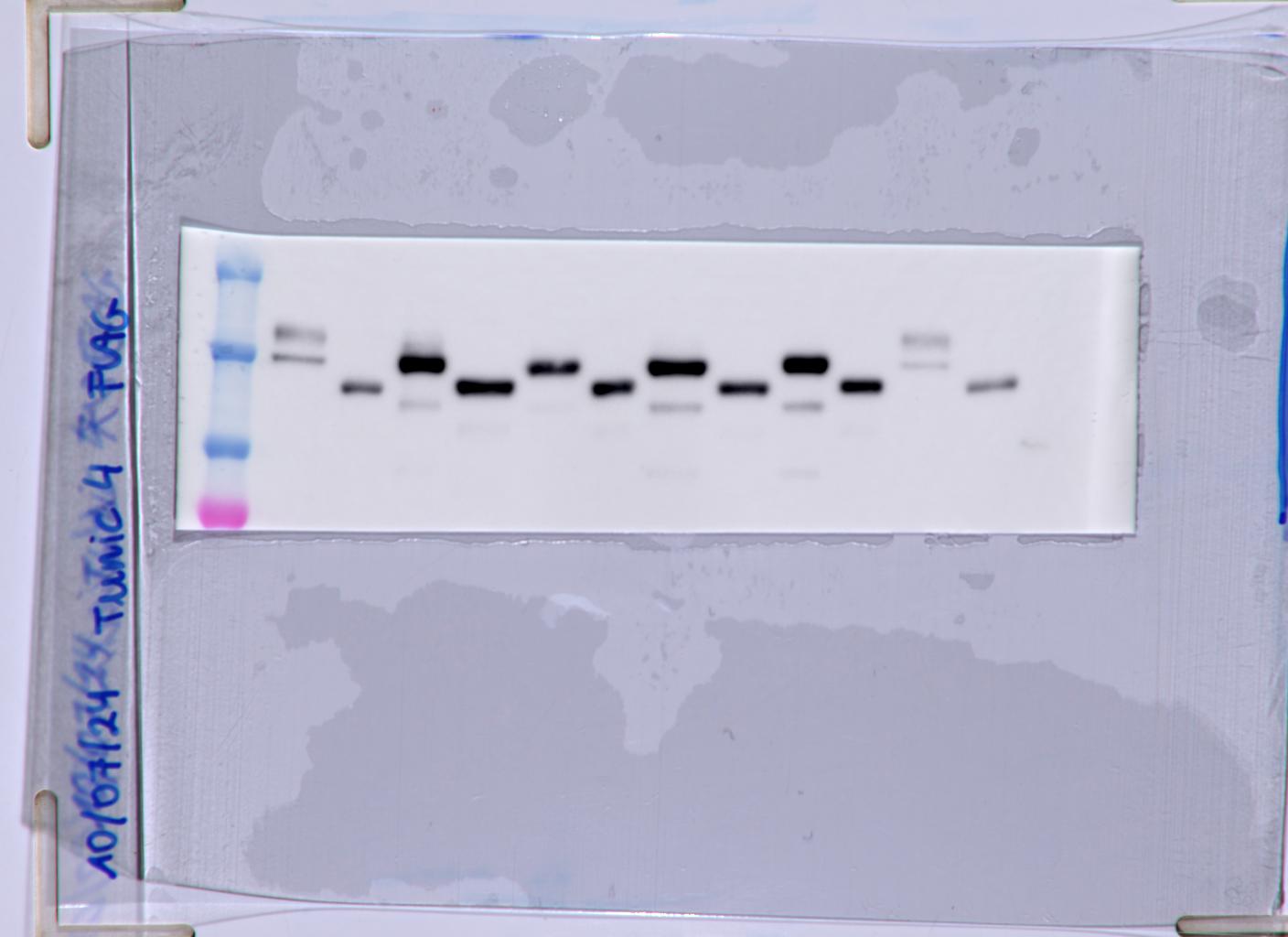

Supplement: Supplementary file 5 — Source data Fig. 2 [file 44318_2025_600_MOESM5_ESM.zip › Figure 2/2C/2C replicates/tunic 4 flag 4s 2024.07.10_10.58.24_Ch+Marker.jpg]

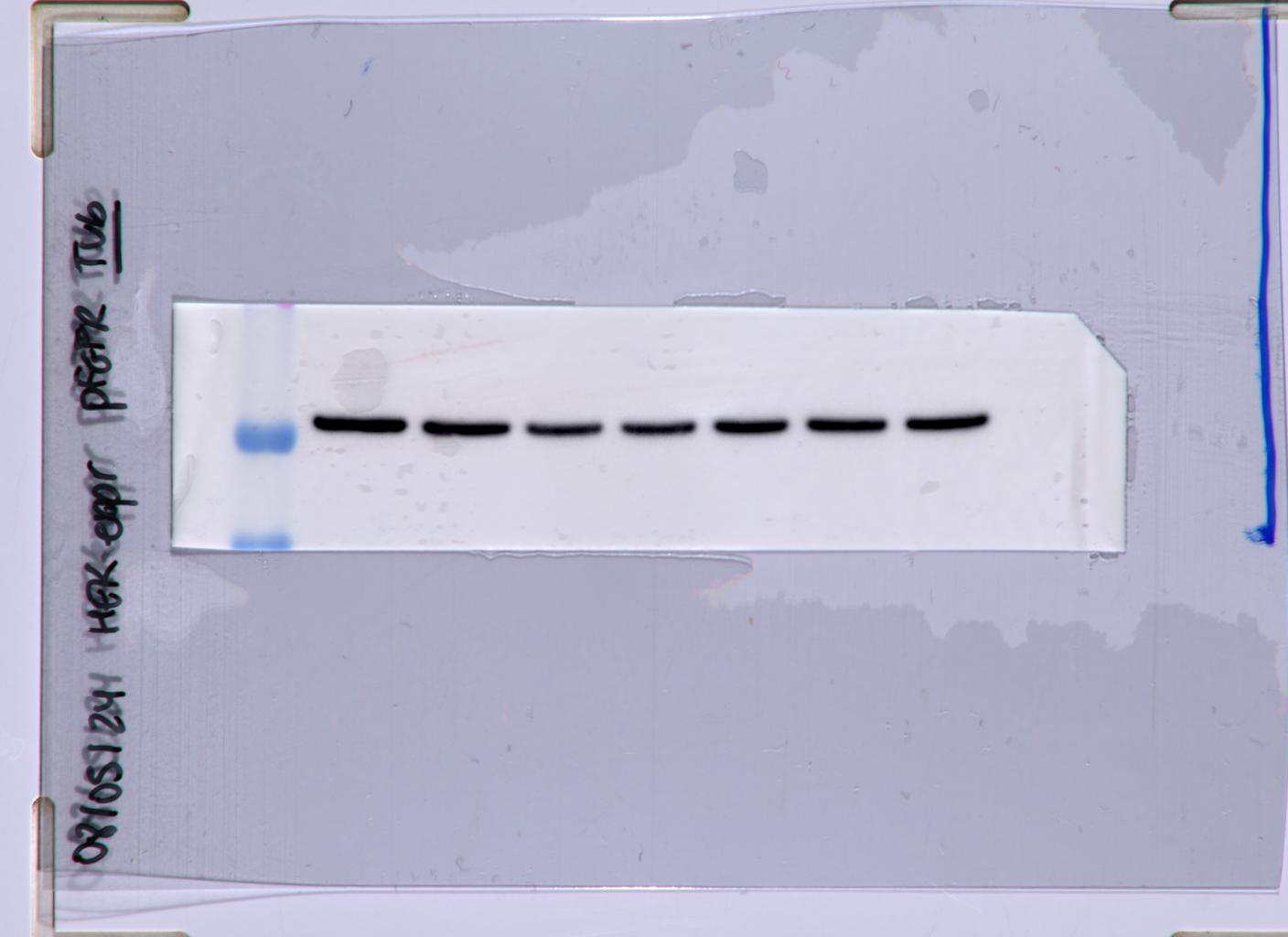

Supplement: Supplementary file 5 — Source data Fig. 2 [file 44318_2025_600_MOESM5_ESM.zip › Figure 2/2C/2C replicates/hekexpr fosfo 3s 2024.05.08_11.51.26_Ch+Marker.jpg]

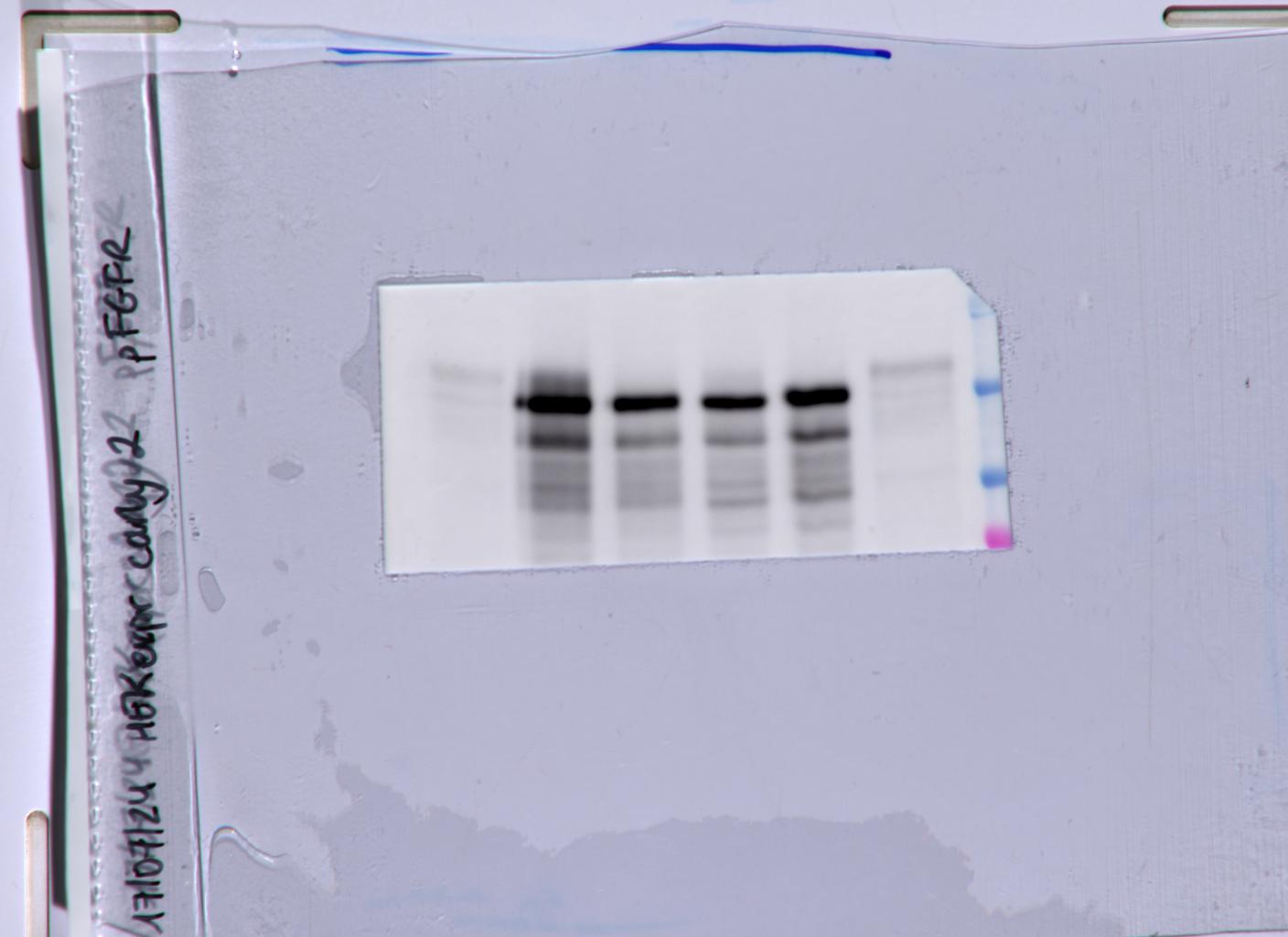

Supplement: Supplementary file 5 — Source data Fig. 2 [file 44318_2025_600_MOESM5_ESM.zip › Figure 2/2C/2C replicates/early2exp pfgfr 0.3s 2024.07.17_14.40.00_Ch+Marker.jpg]

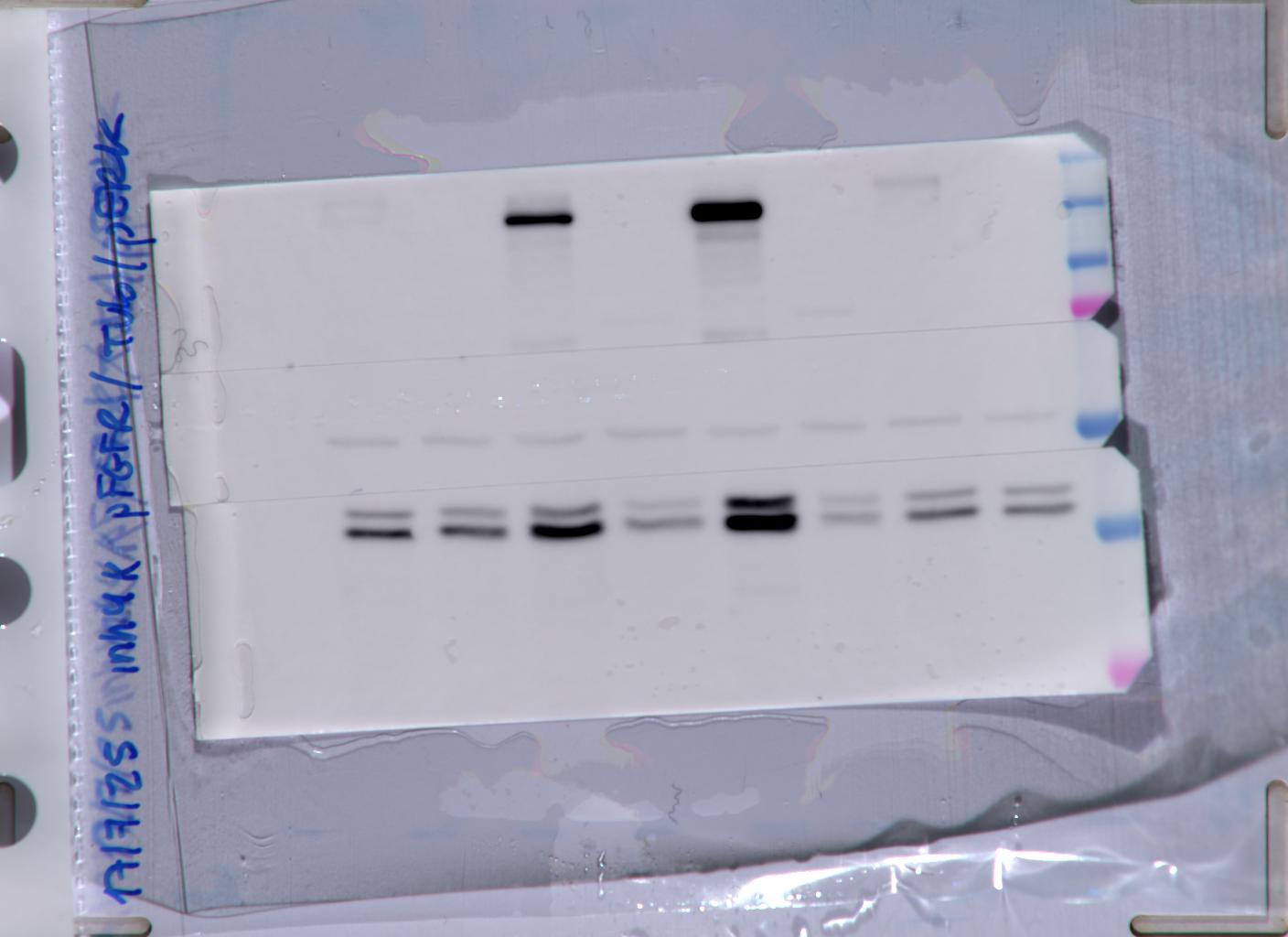

Supplement: Supplementary file 6 — Source data Fig. 3 [file 44318_2025_600_MOESM6_ESM.zip › Figure 3/3E/pFGFR1+pERK K,KR,R original.jpg]

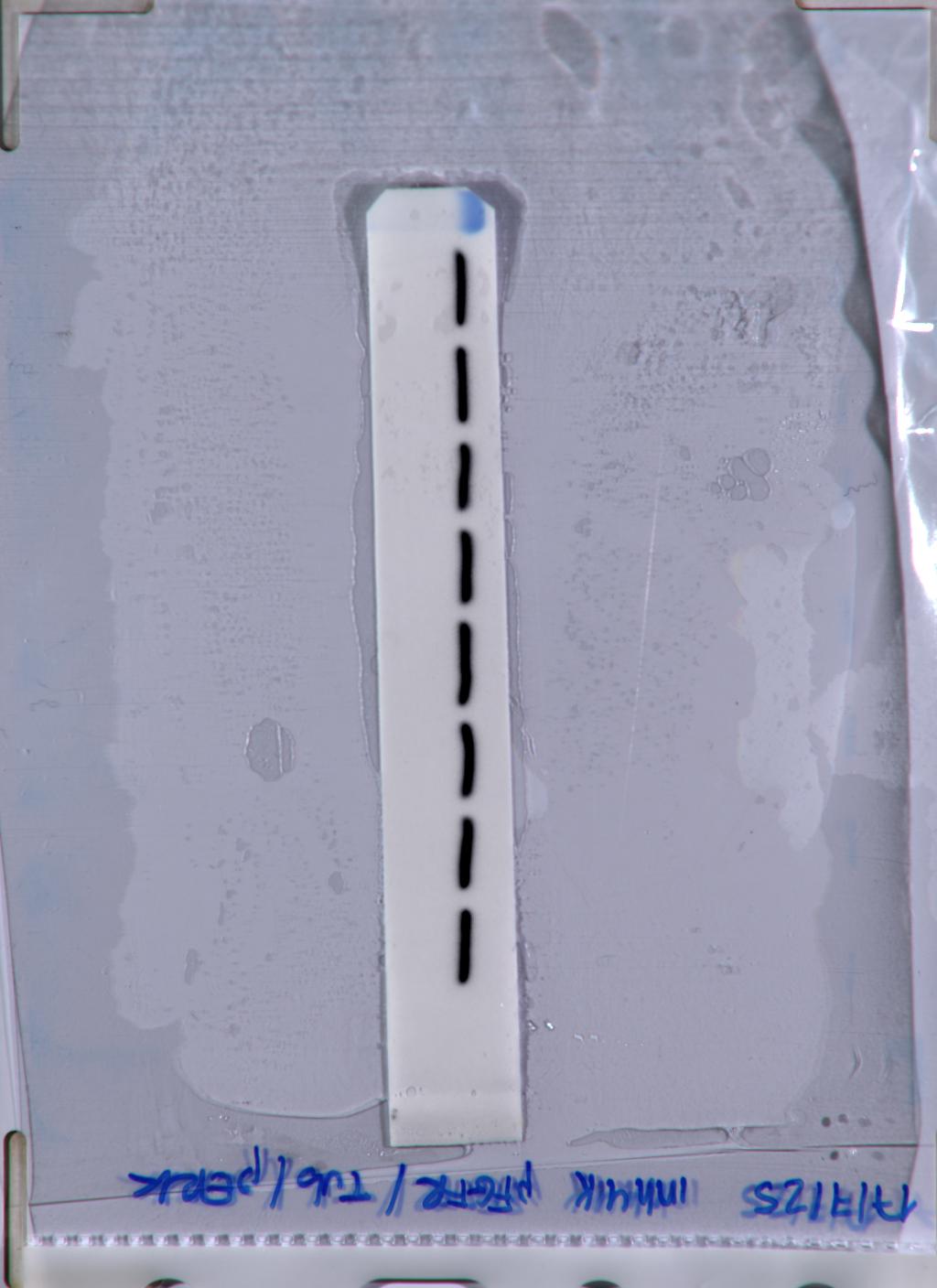

Supplement: Supplementary file 6 — Source data Fig. 3 [file 44318_2025_600_MOESM6_ESM.zip › Figure 3/3E/tub (pFGFR1 membrane) K,KR,R original.jpg]

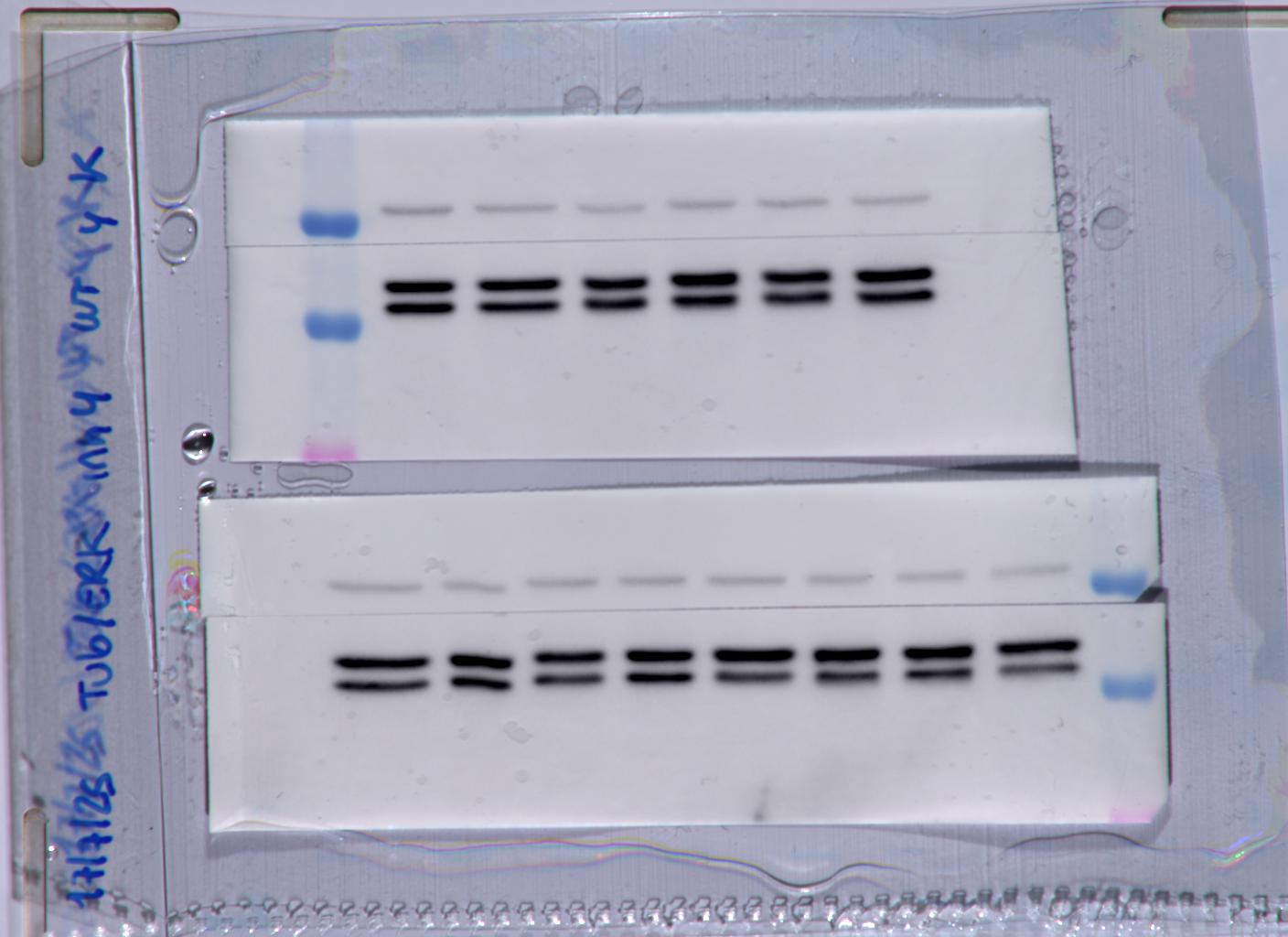

Supplement: Supplementary file 6 — Source data Fig. 3 [file 44318_2025_600_MOESM6_ESM.zip › Figure 3/3E/ERK WT,N,NR + K,KR,R original.jpg]

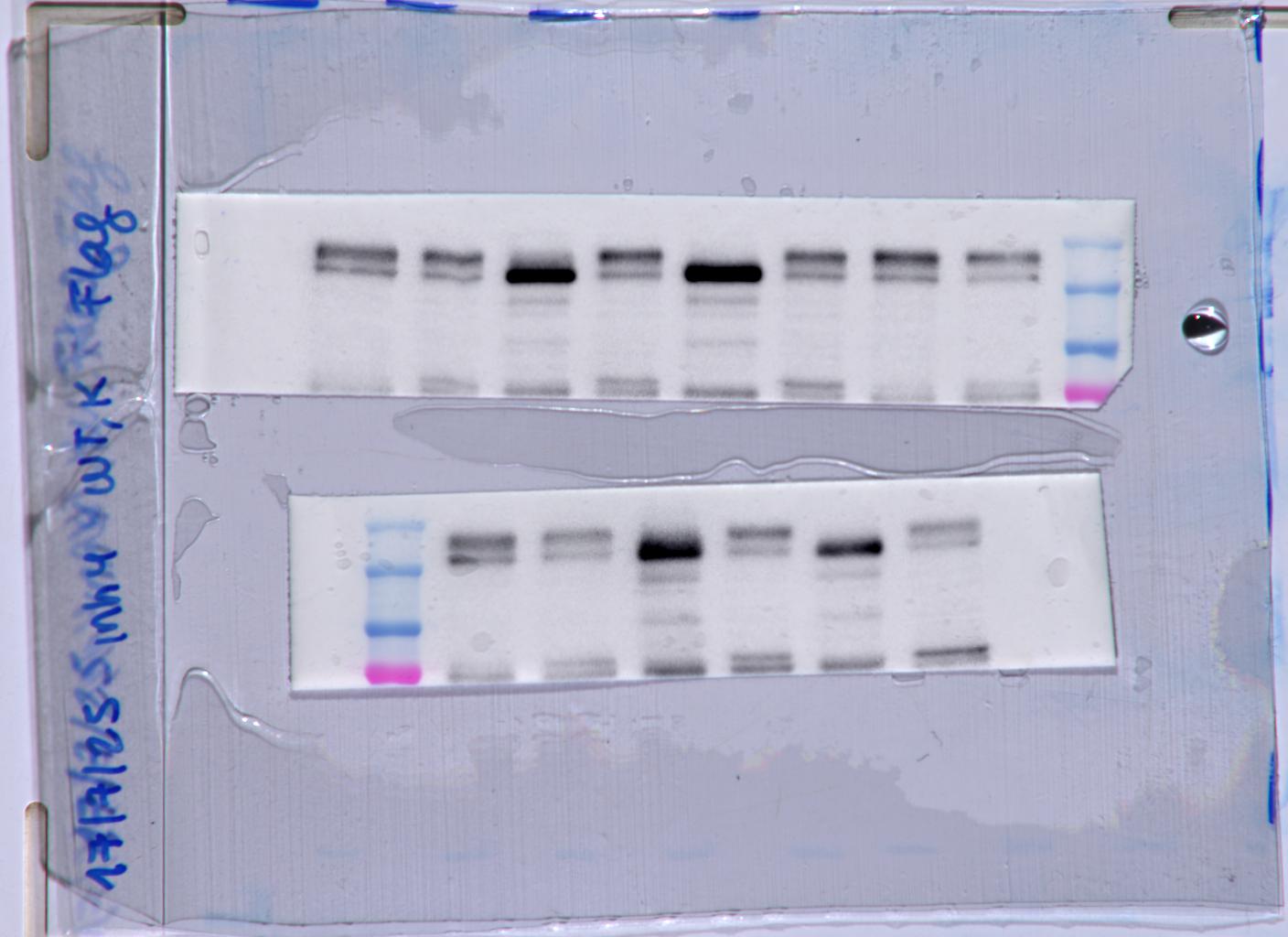

Supplement: Supplementary file 6 — Source data Fig. 3 [file 44318_2025_600_MOESM6_ESM.zip › Figure 3/3E/flag WT, N, NR + K,KR,R original.jpg]

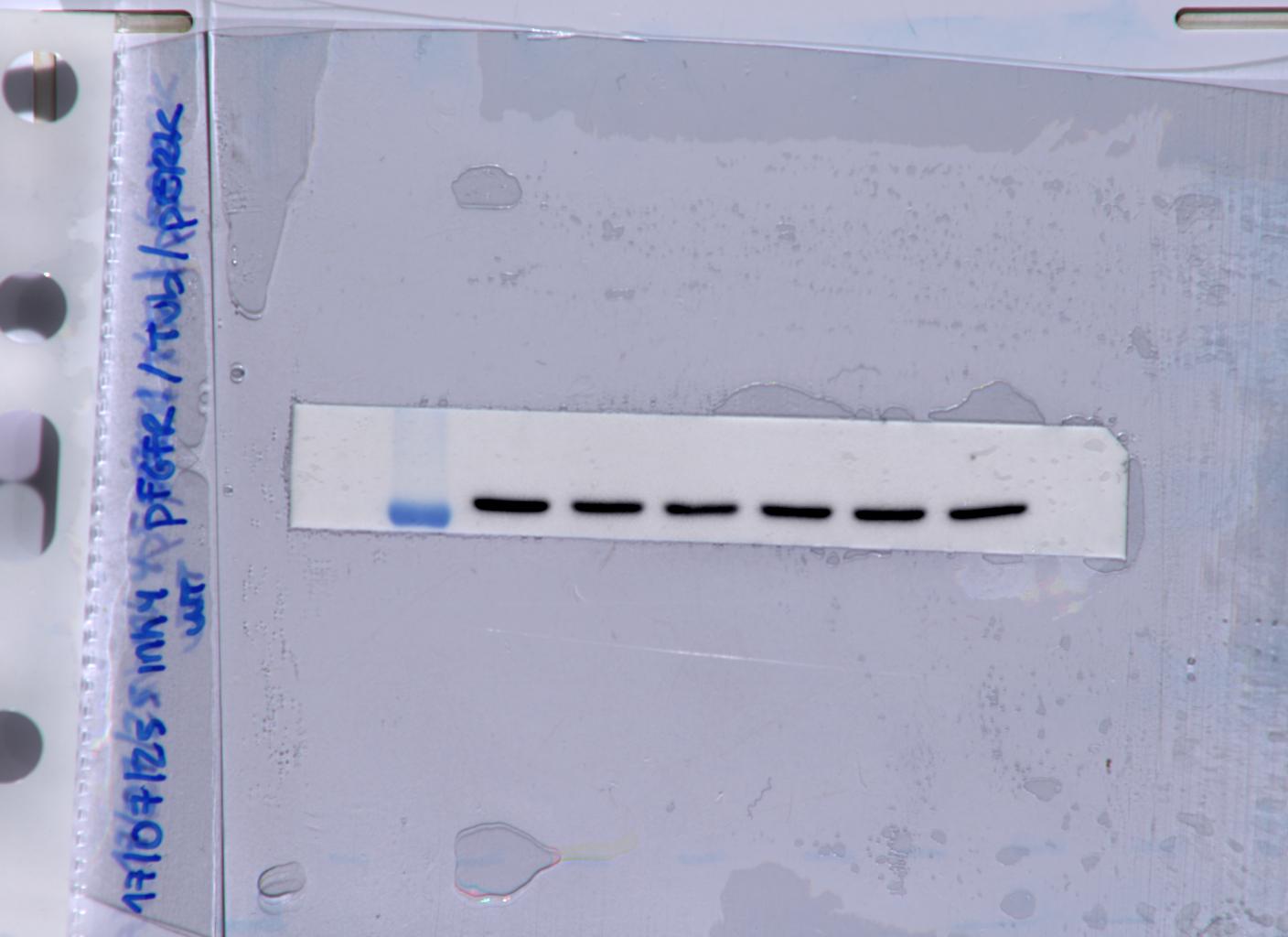

Supplement: Supplementary file 6 — Source data Fig. 3 [file 44318_2025_600_MOESM6_ESM.zip › Figure 3/3E/tub (pFGFR1 membrane) WT,N,NR original.jpg]

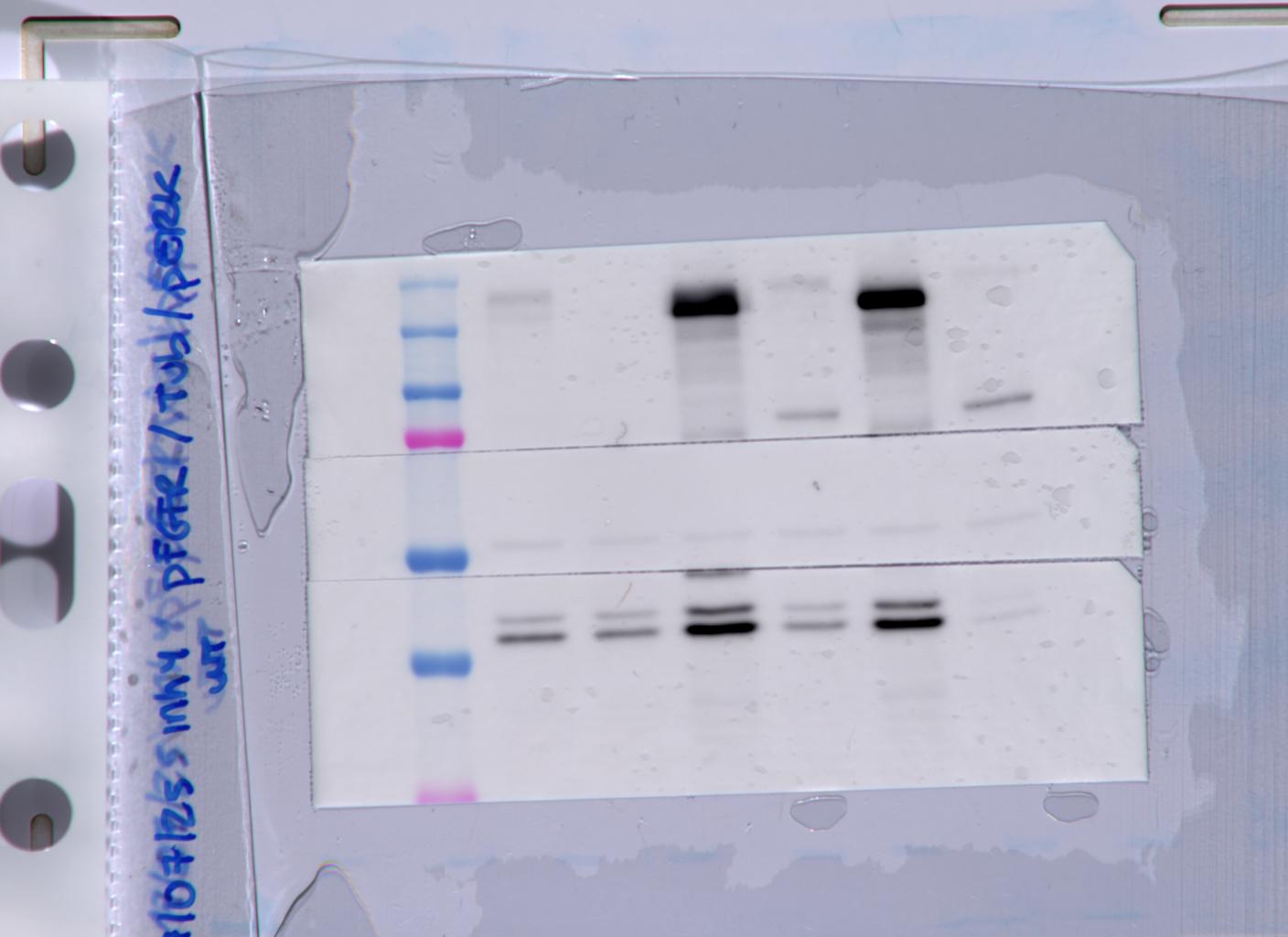

Supplement: Supplementary file 6 — Source data Fig. 3 [file 44318_2025_600_MOESM6_ESM.zip › Figure 3/3E/pFGFR1+pERK WT,N,NR original.jpg]

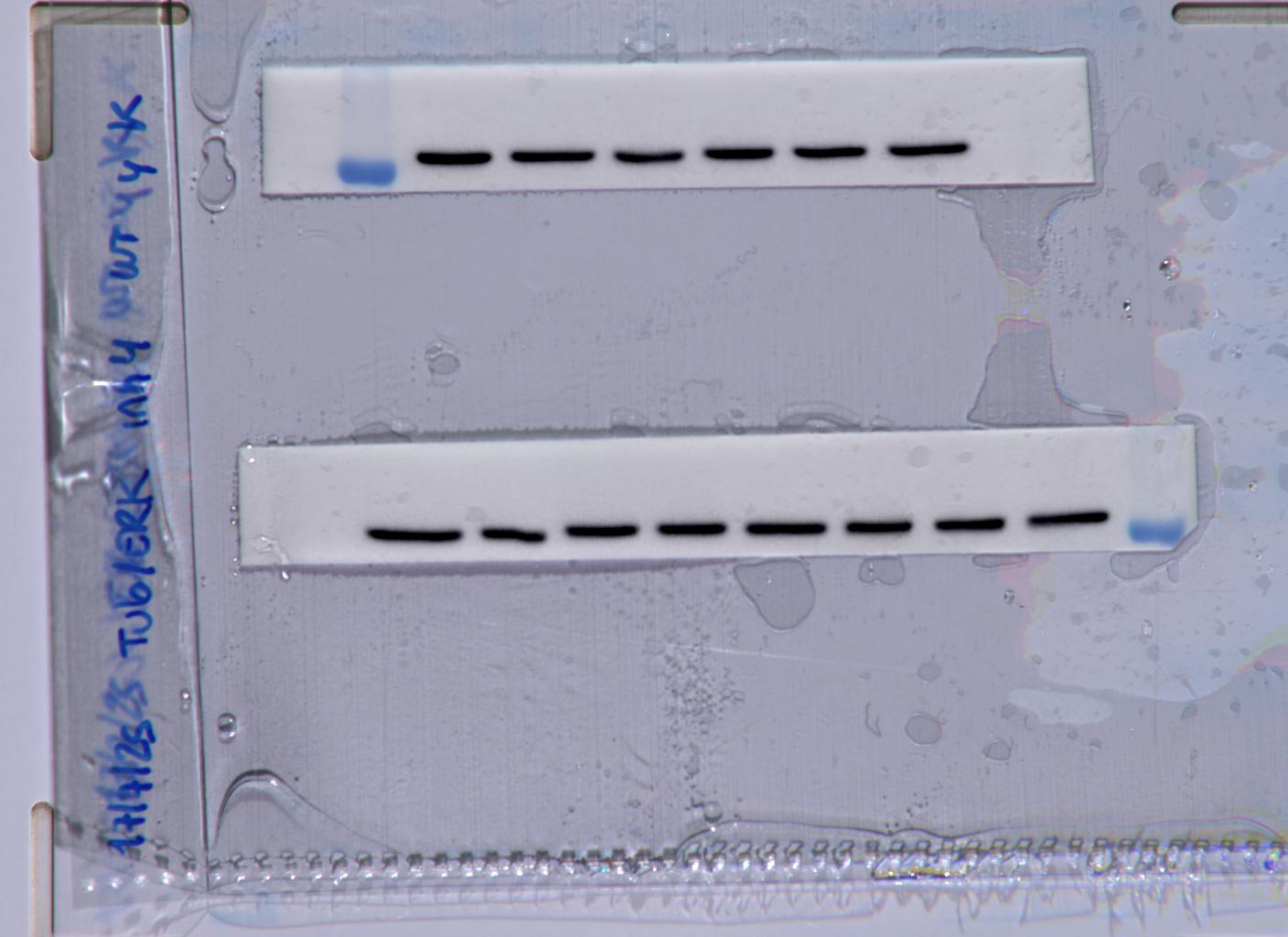

Supplement: Supplementary file 6 — Source data Fig. 3 [file 44318_2025_600_MOESM6_ESM.zip › Figure 3/3E/tub WT,N,NR + K,KR,R original.jpg]

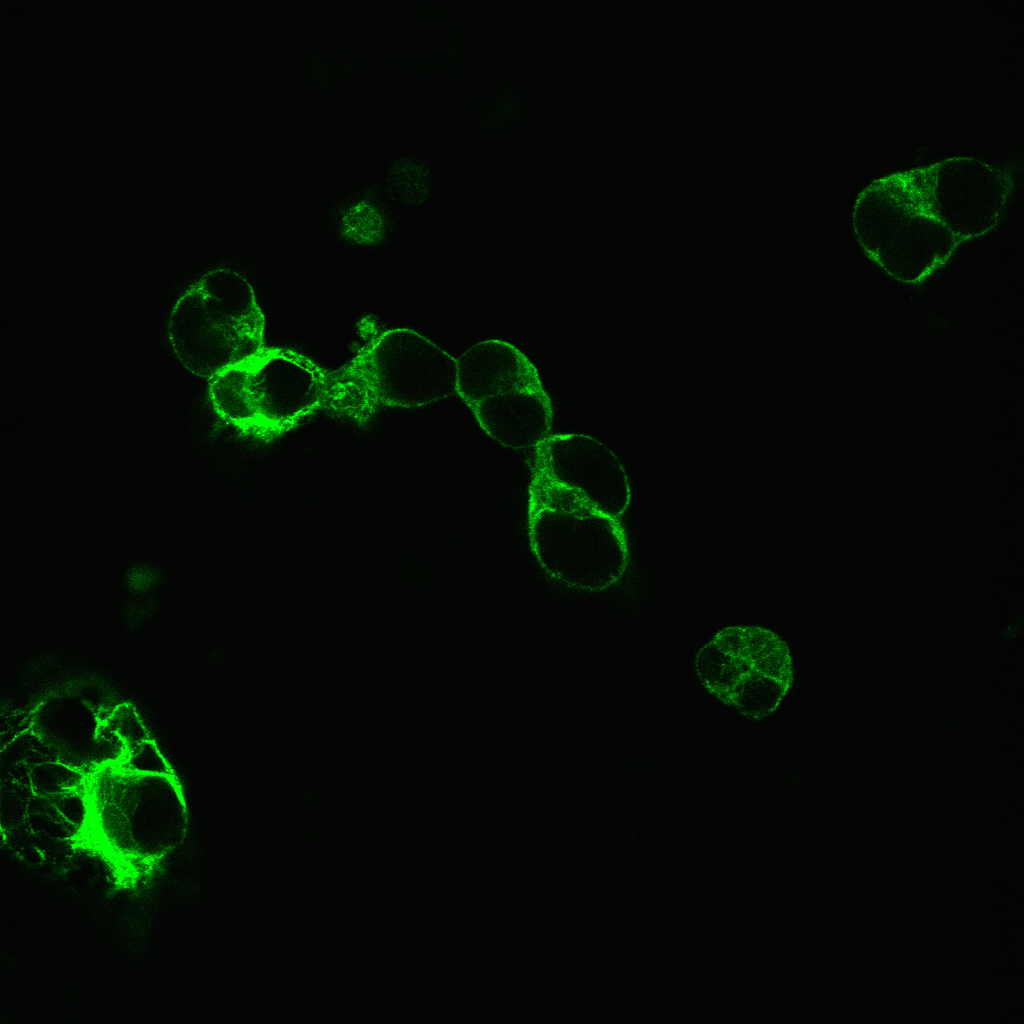

Supplement: Supplementary file 6 — Source data Fig. 3 [file 44318_2025_600_MOESM6_ESM.zip › Figure 3/3C/N546K_R661P, Tubulin.tif]

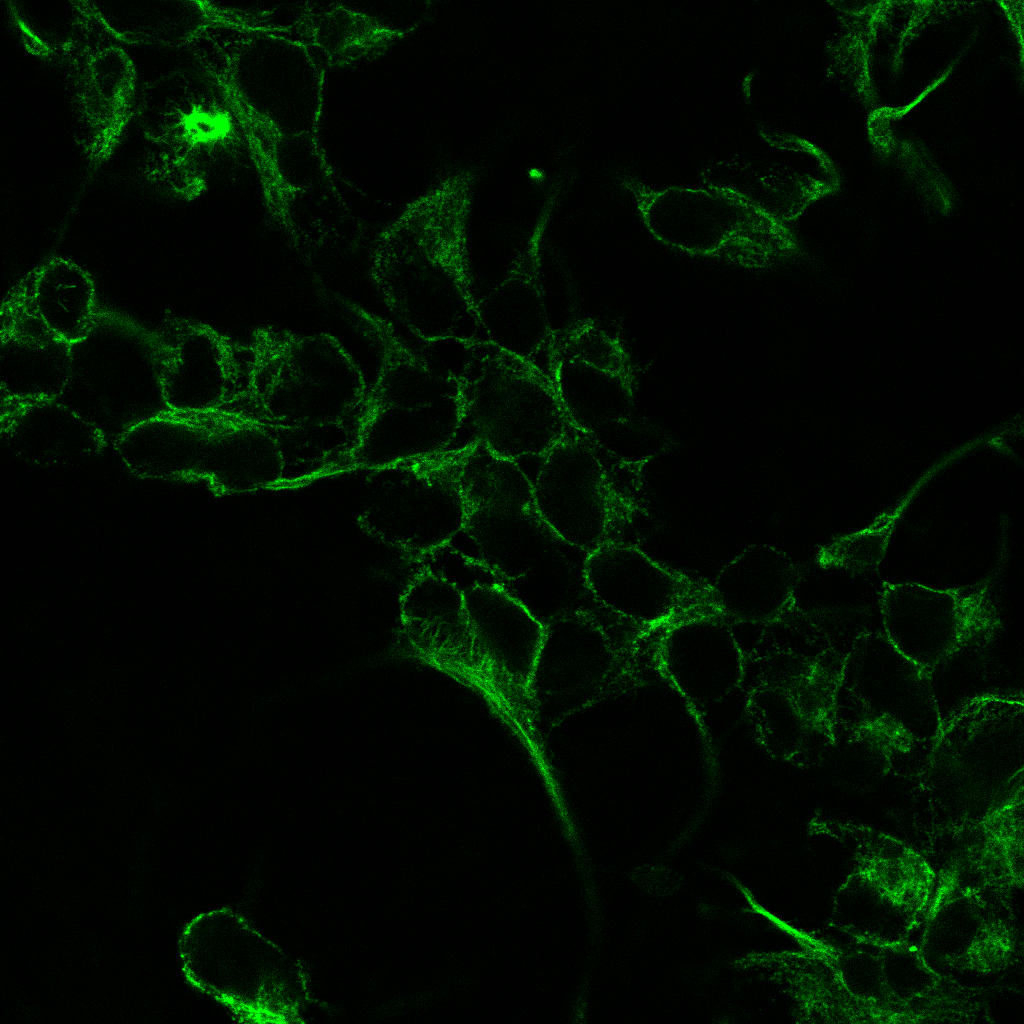

Supplement: Supplementary file 6 — Source data Fig. 3 [file 44318_2025_600_MOESM6_ESM.zip › Figure 3/3C/R661P, Tubulin.tif]

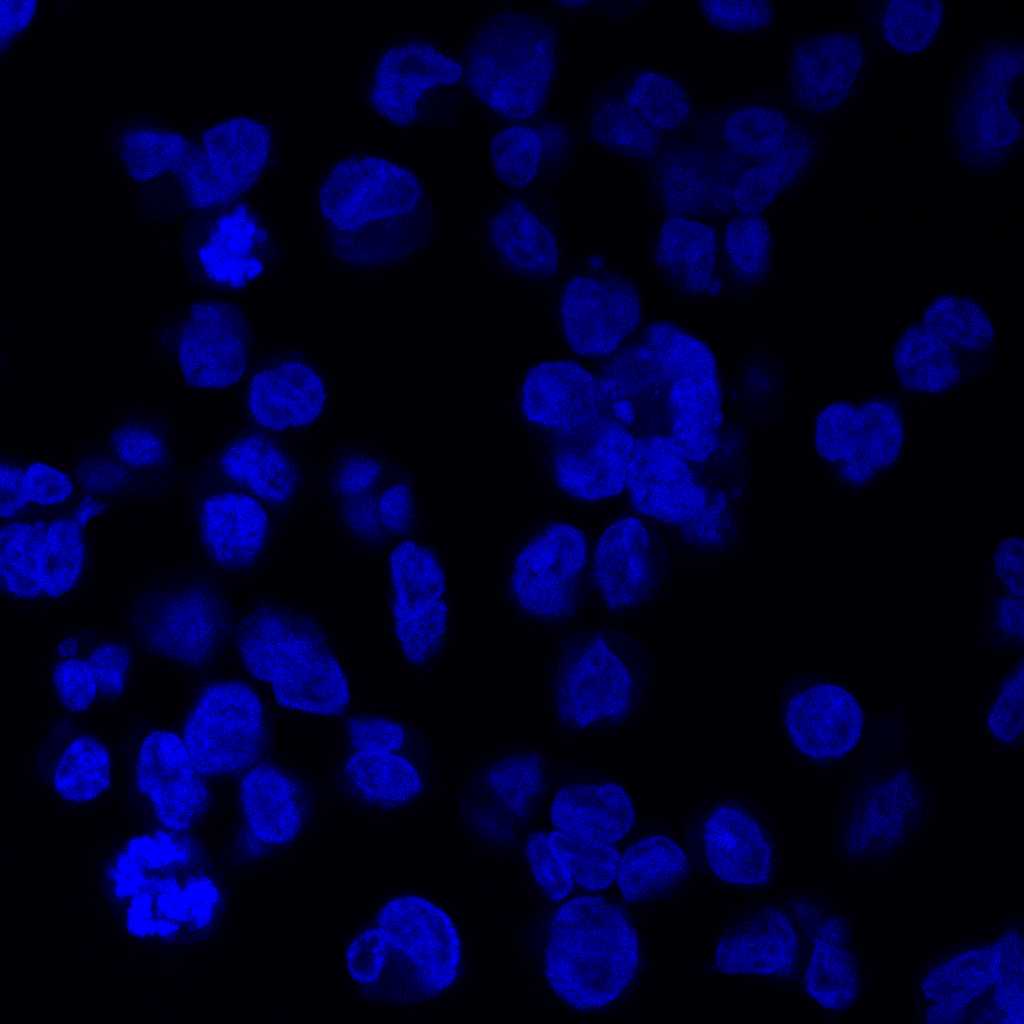

Supplement: Supplementary file 6 — Source data Fig. 3 [file 44318_2025_600_MOESM6_ESM.zip › Figure 3/3C/K656E, DAPI.tif]

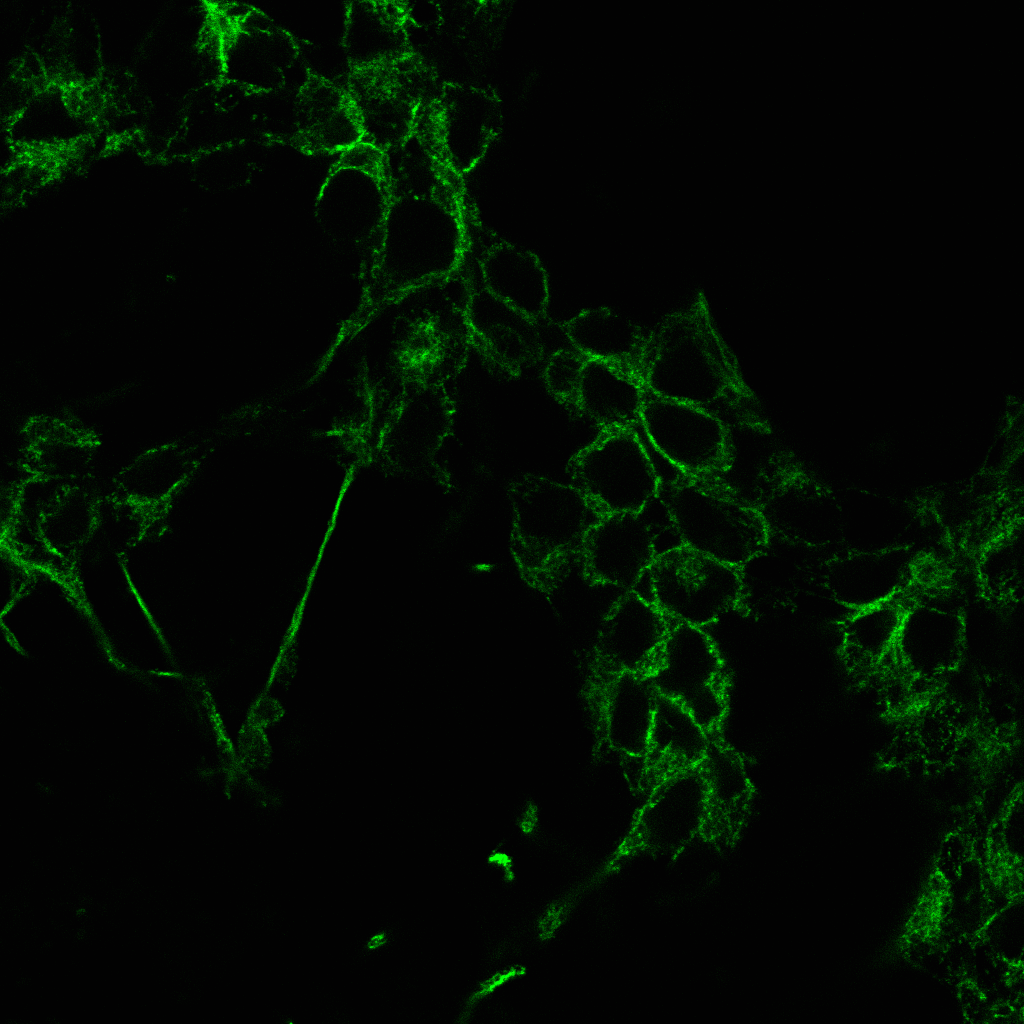

Supplement: Supplementary file 6 — Source data Fig. 3 [file 44318_2025_600_MOESM6_ESM.zip › Figure 3/3C/WT, Tubulin.tif]

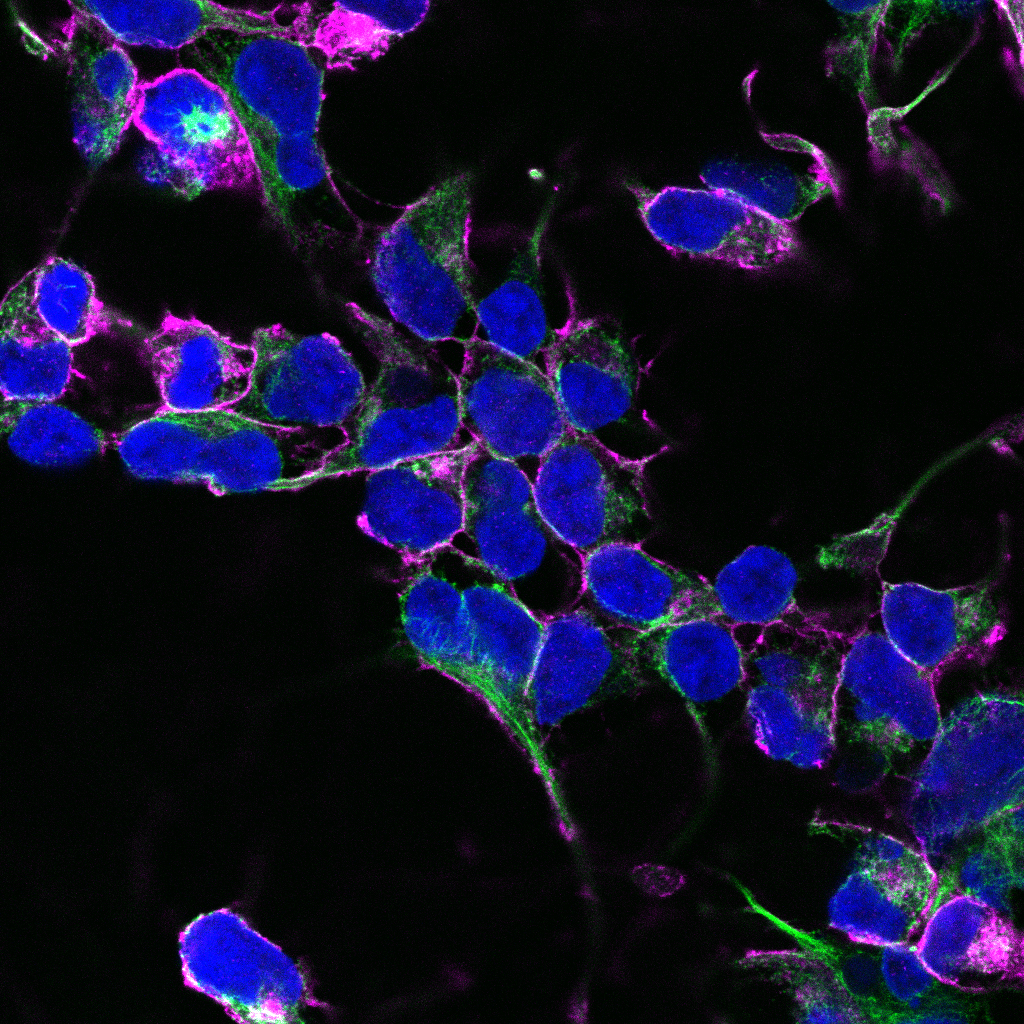

Supplement: Supplementary file 6 — Source data Fig. 3 [file 44318_2025_600_MOESM6_ESM.zip › Figure 3/3C/R661P, Merge.tif]

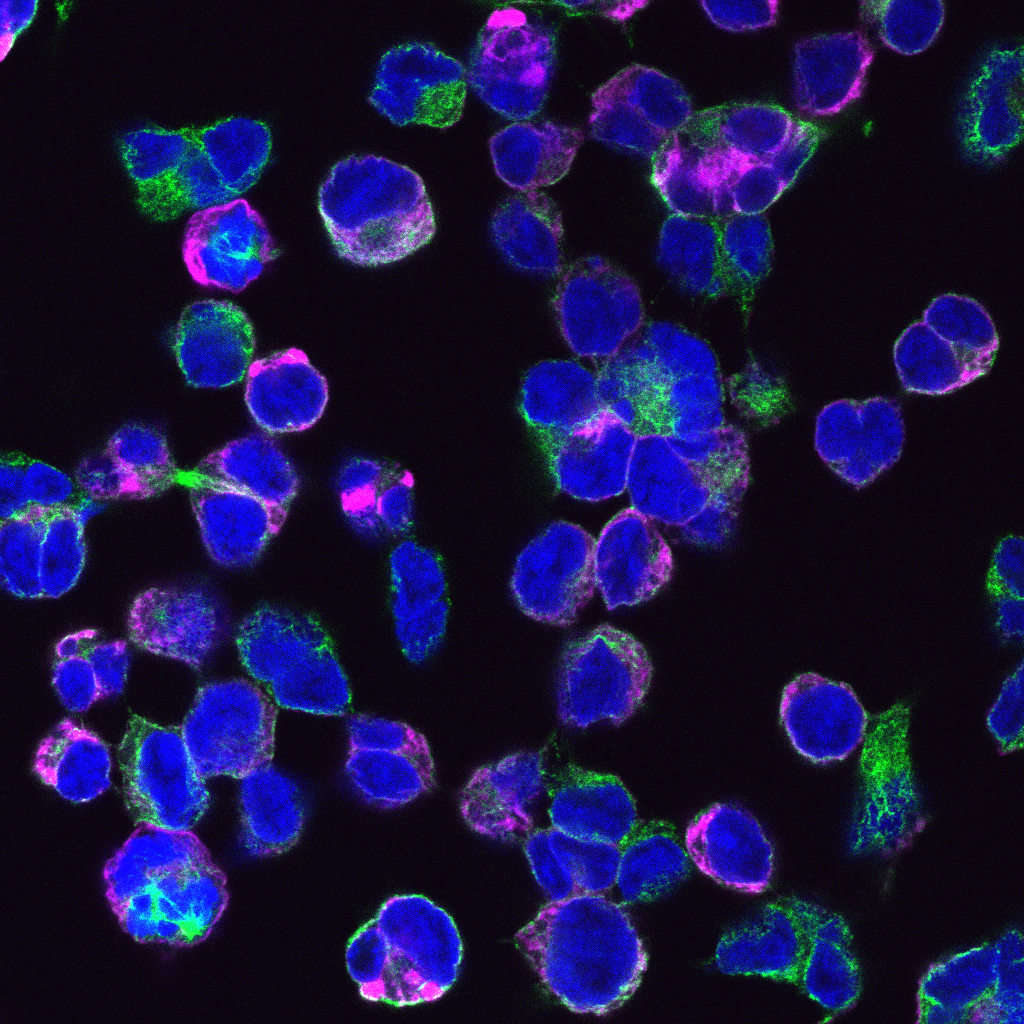

Supplement: Supplementary file 6 — Source data Fig. 3 [file 44318_2025_600_MOESM6_ESM.zip › Figure 3/3C/K656E, Merge.tif]

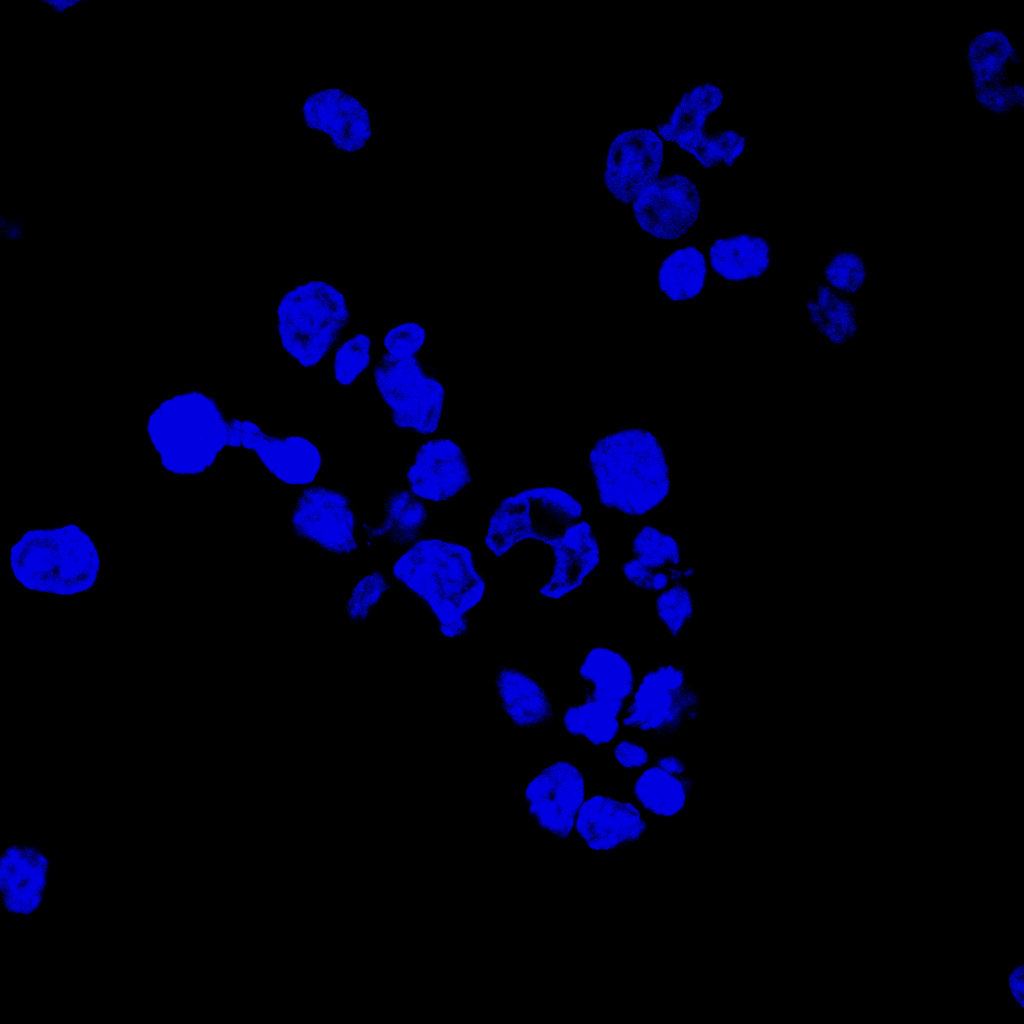

Supplement: Supplementary file 6 — Source data Fig. 3 [file 44318_2025_600_MOESM6_ESM.zip › Figure 3/3C/N546K, DAPI.tif]

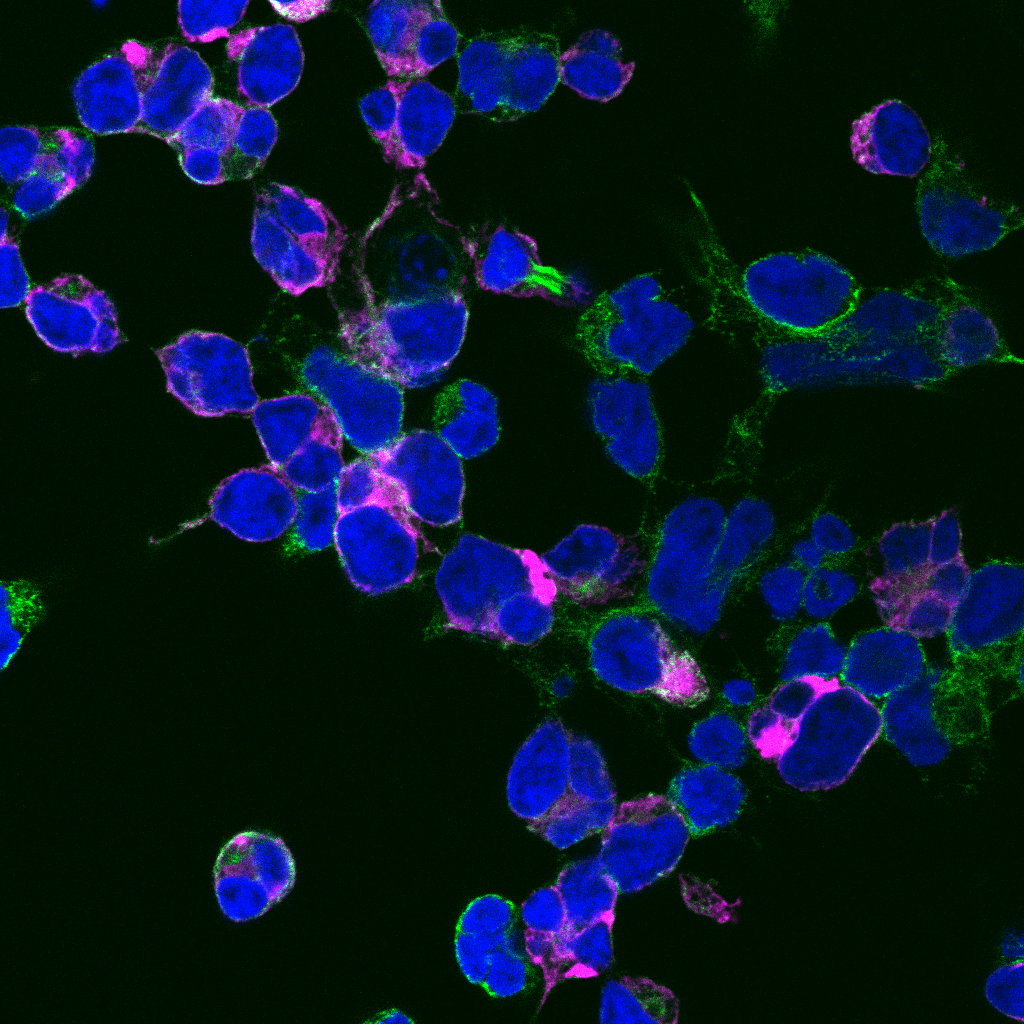

Supplement: Supplementary file 6 — Source data Fig. 3 [file 44318_2025_600_MOESM6_ESM.zip › Figure 3/3C/K656E_R661P, Merge.tif]

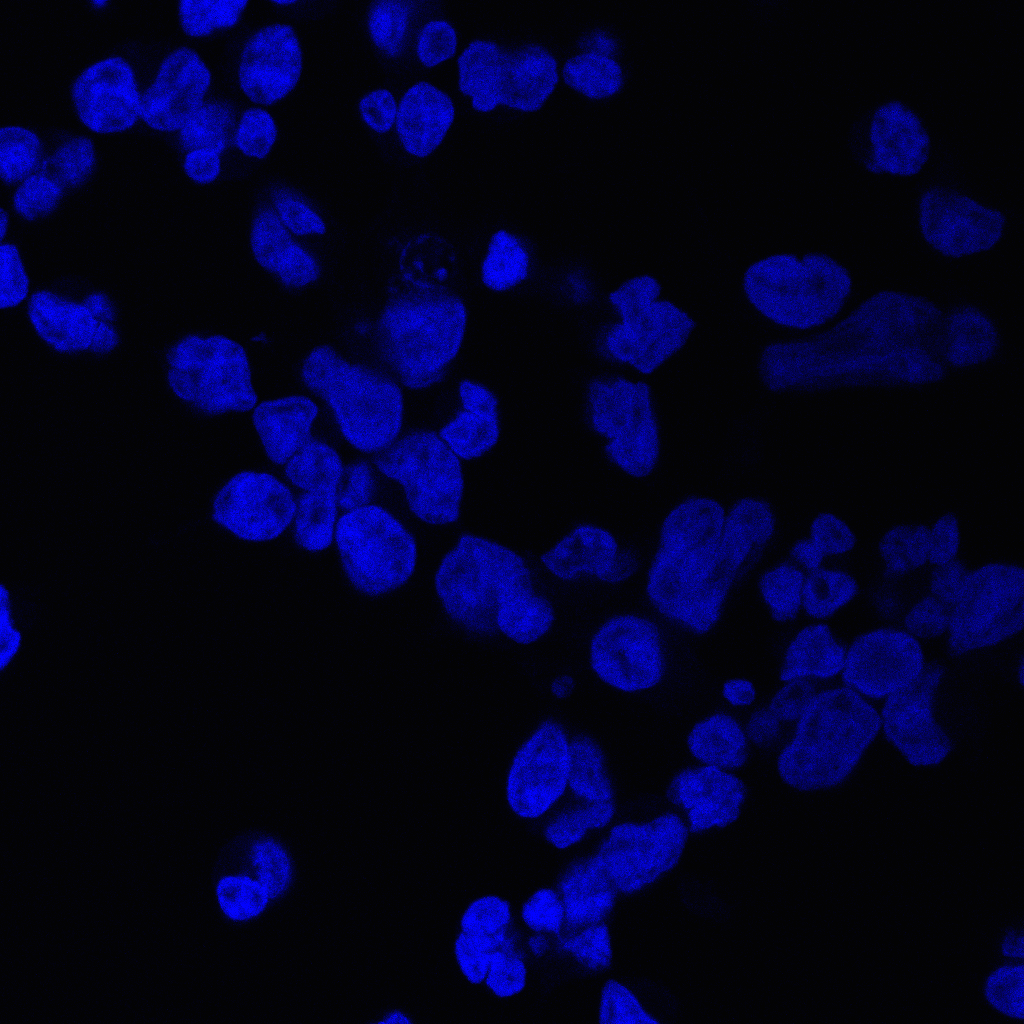

Supplement: Supplementary file 6 — Source data Fig. 3 [file 44318_2025_600_MOESM6_ESM.zip › Figure 3/3C/K656E_R661P, DAPI.tif]

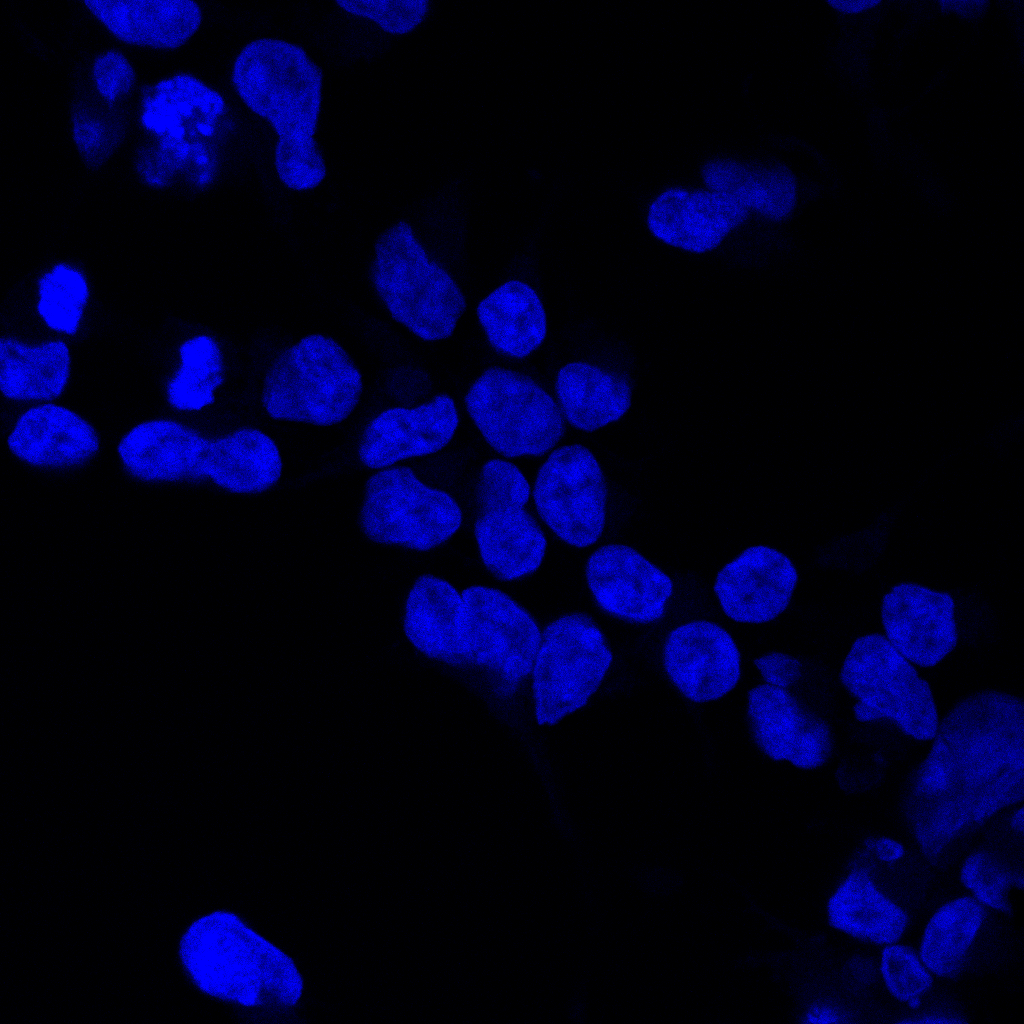

Supplement: Supplementary file 6 — Source data Fig. 3 [file 44318_2025_600_MOESM6_ESM.zip › Figure 3/3C/R661P, DAPI.tif]

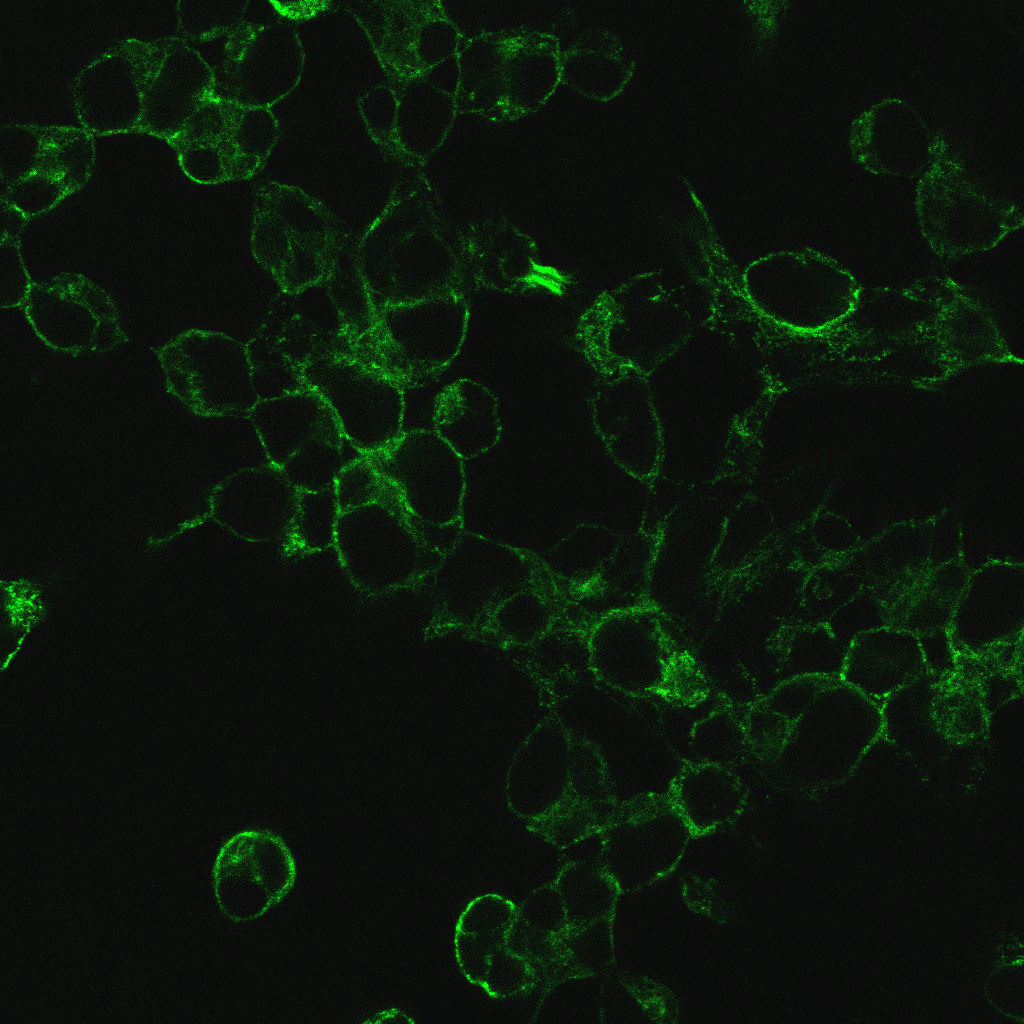

Supplement: Supplementary file 6 — Source data Fig. 3 [file 44318_2025_600_MOESM6_ESM.zip › Figure 3/3C/K656E_R661P, Tubulin.tif]

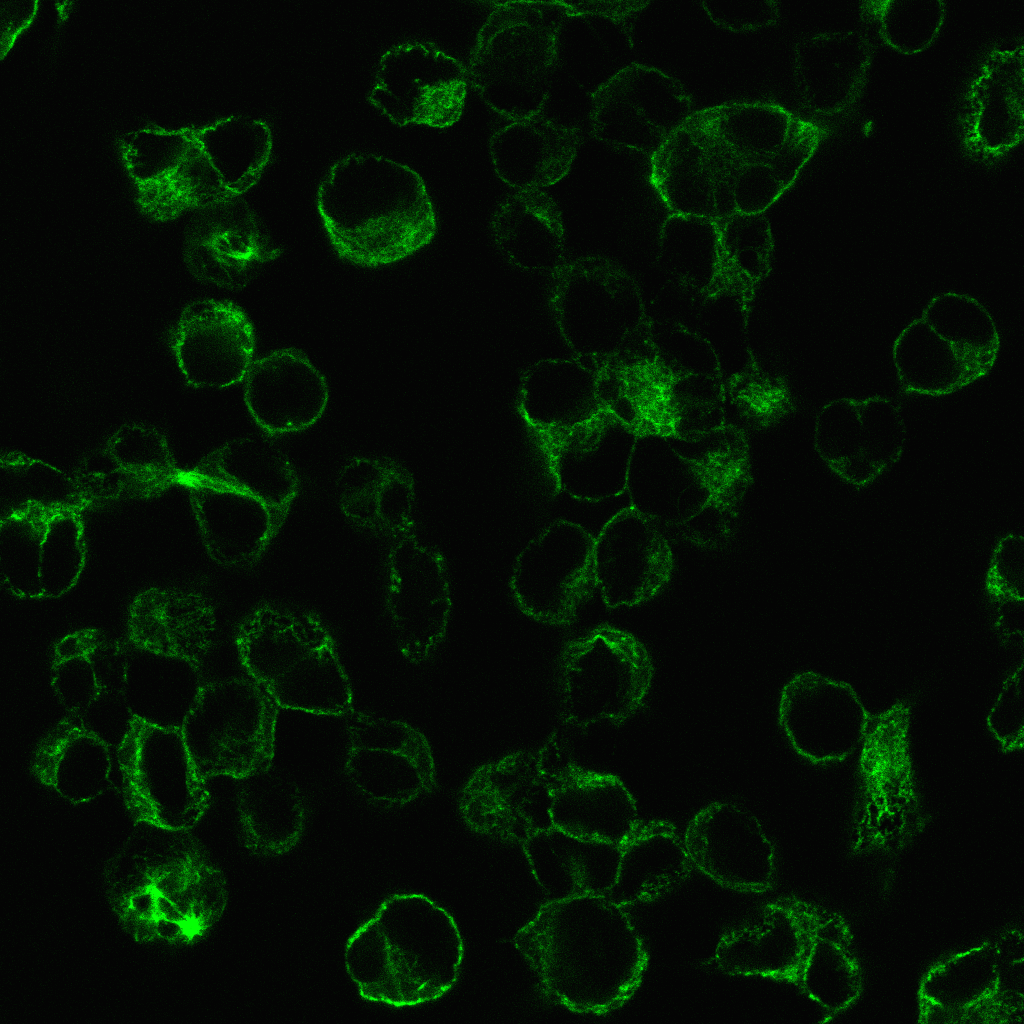

Supplement: Supplementary file 6 — Source data Fig. 3 [file 44318_2025_600_MOESM6_ESM.zip › Figure 3/3C/K656E, Tubulin.tif]

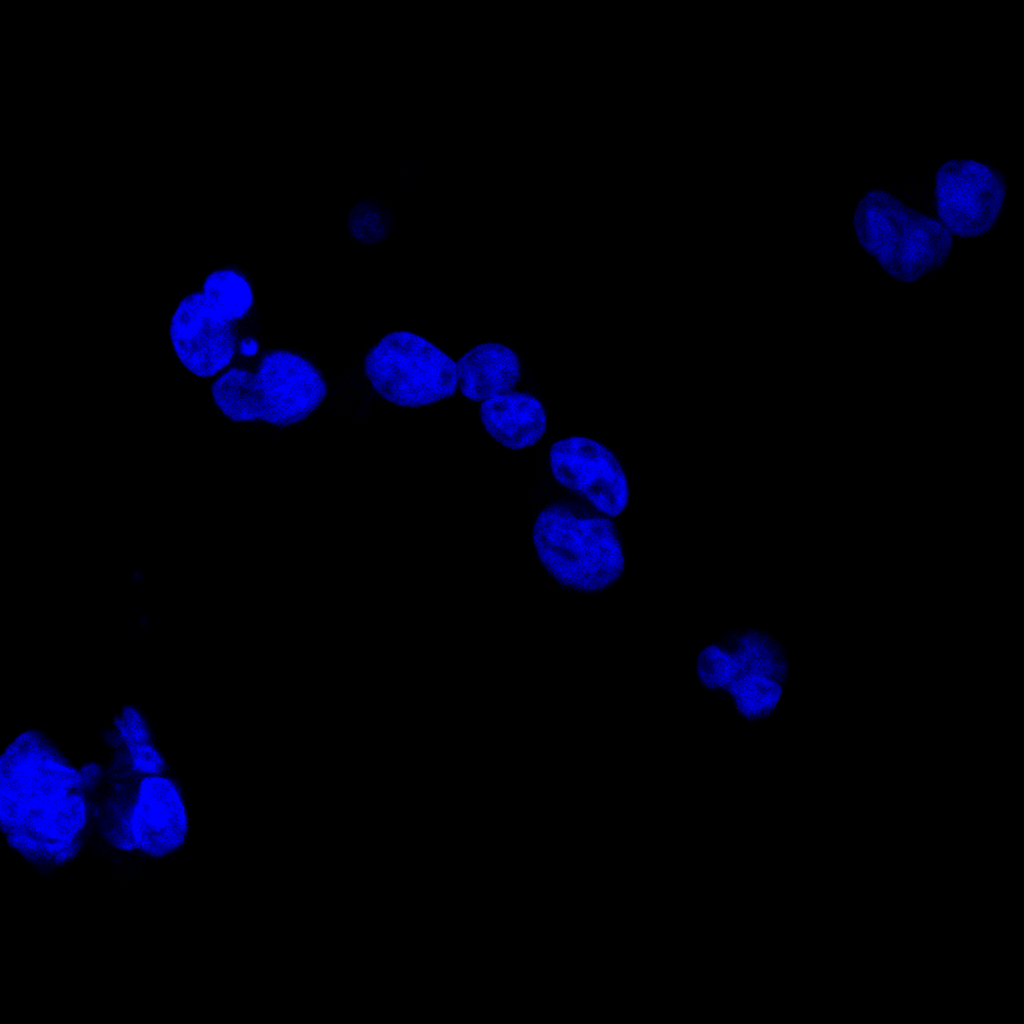

Supplement: Supplementary file 6 — Source data Fig. 3 [file 44318_2025_600_MOESM6_ESM.zip › Figure 3/3C/N546K_R661P, DAPI.tif]

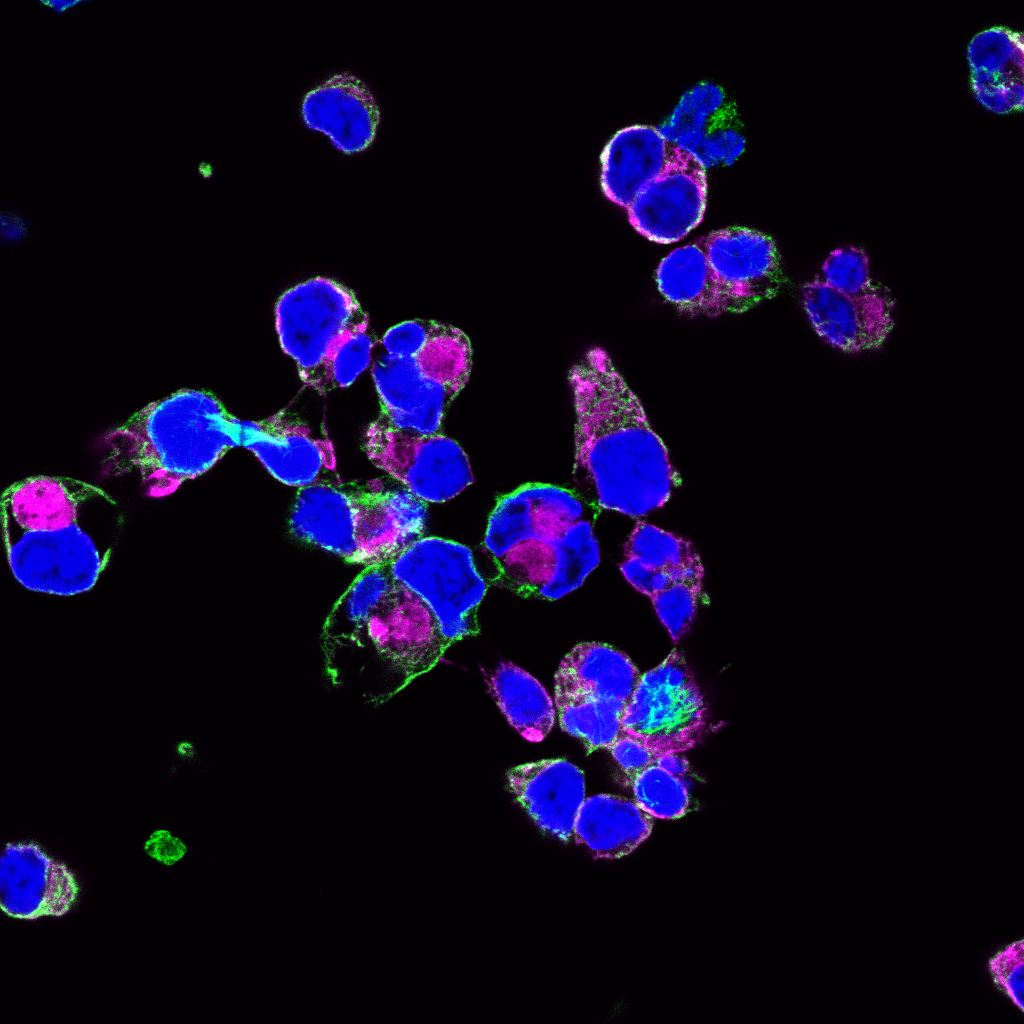

Supplement: Supplementary file 6 — Source data Fig. 3 [file 44318_2025_600_MOESM6_ESM.zip › Figure 3/3C/N546K, Merge.tif]

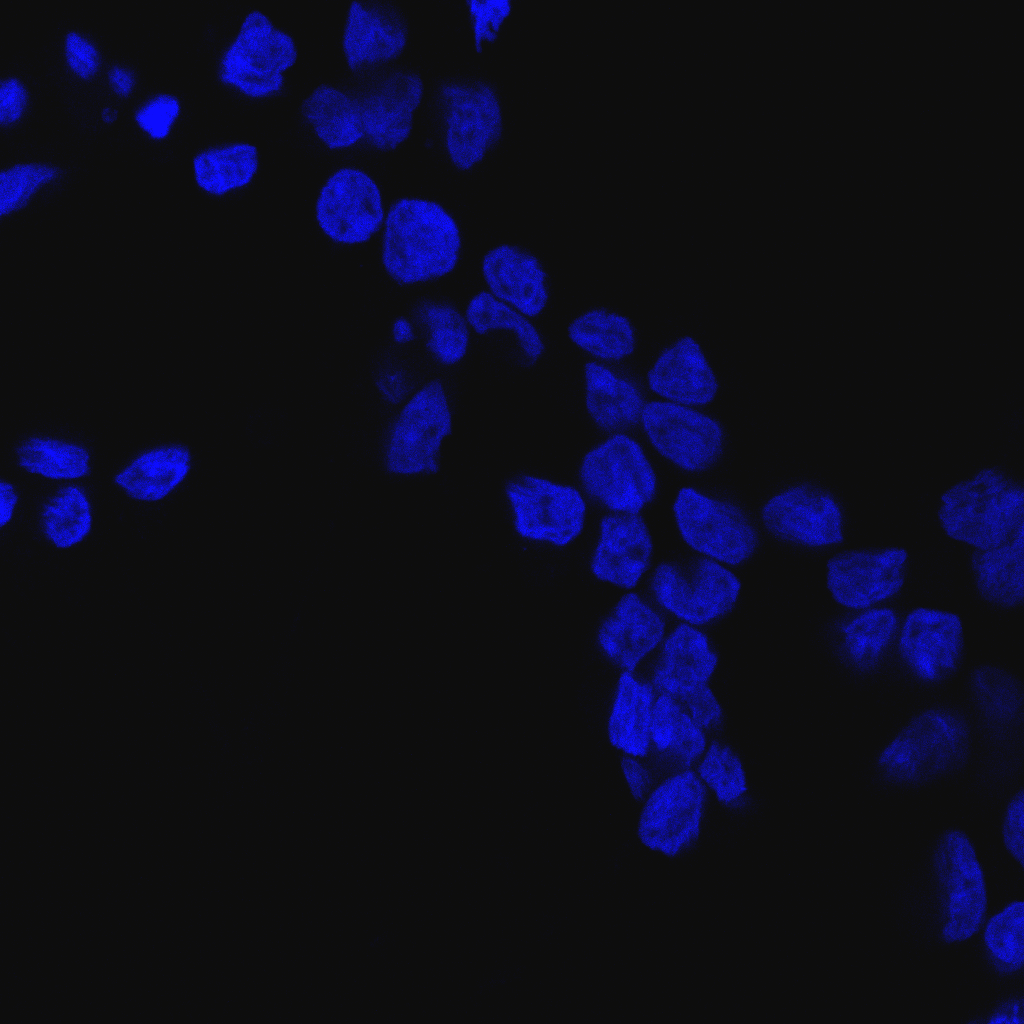

Supplement: Supplementary file 6 — Source data Fig. 3 [file 44318_2025_600_MOESM6_ESM.zip › Figure 3/3C/WT, DAPI.tif]

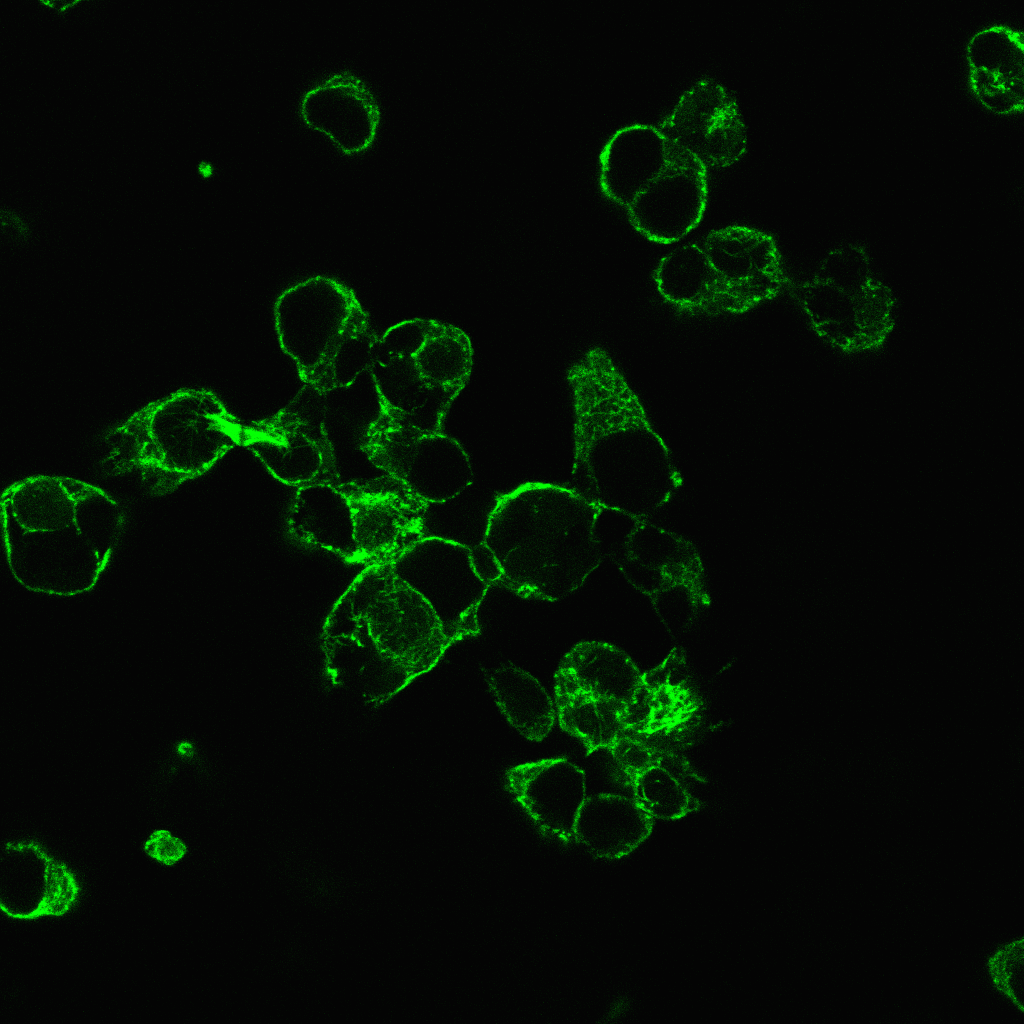

Supplement: Supplementary file 6 — Source data Fig. 3 [file 44318_2025_600_MOESM6_ESM.zip › Figure 3/3C/N546K, Tubulin.tif]

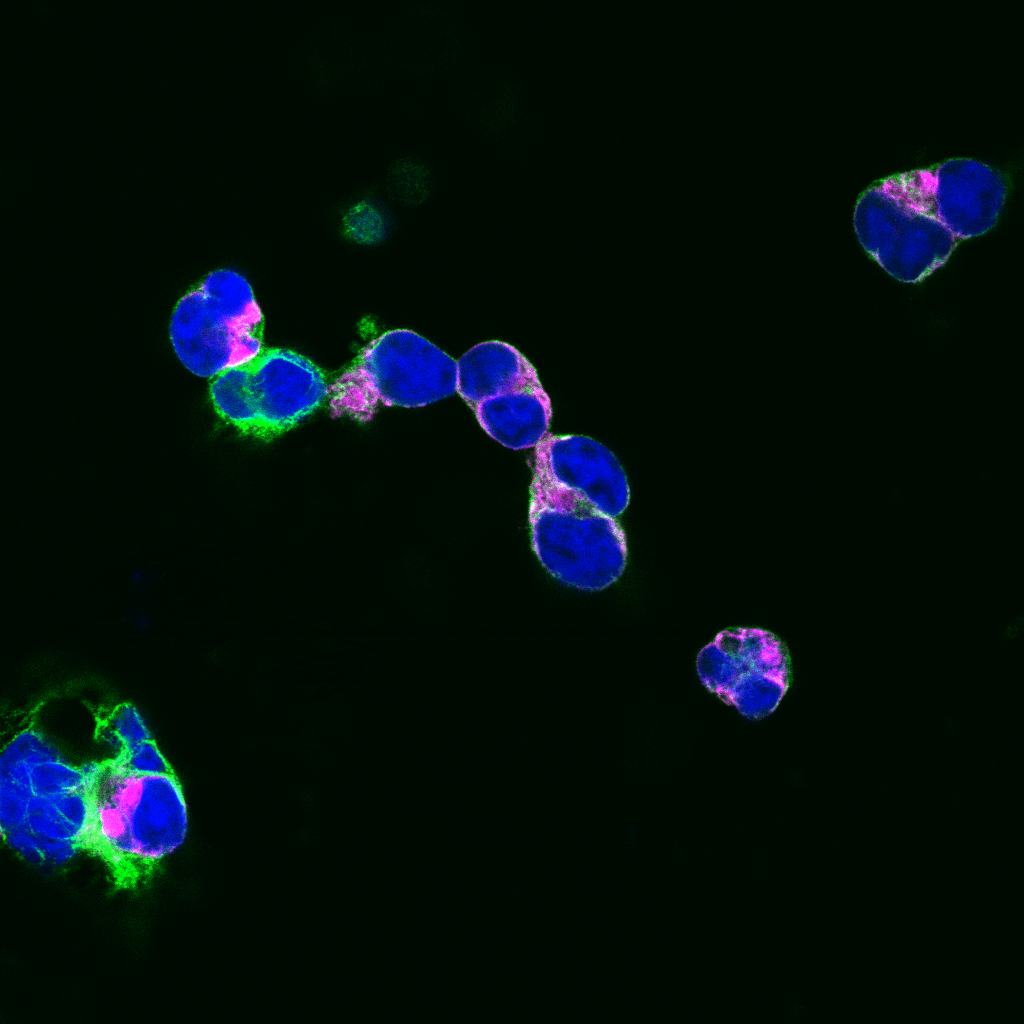

Supplement: Supplementary file 6 — Source data Fig. 3 [file 44318_2025_600_MOESM6_ESM.zip › Figure 3/3C/N546K_R661P, Merge.tif]

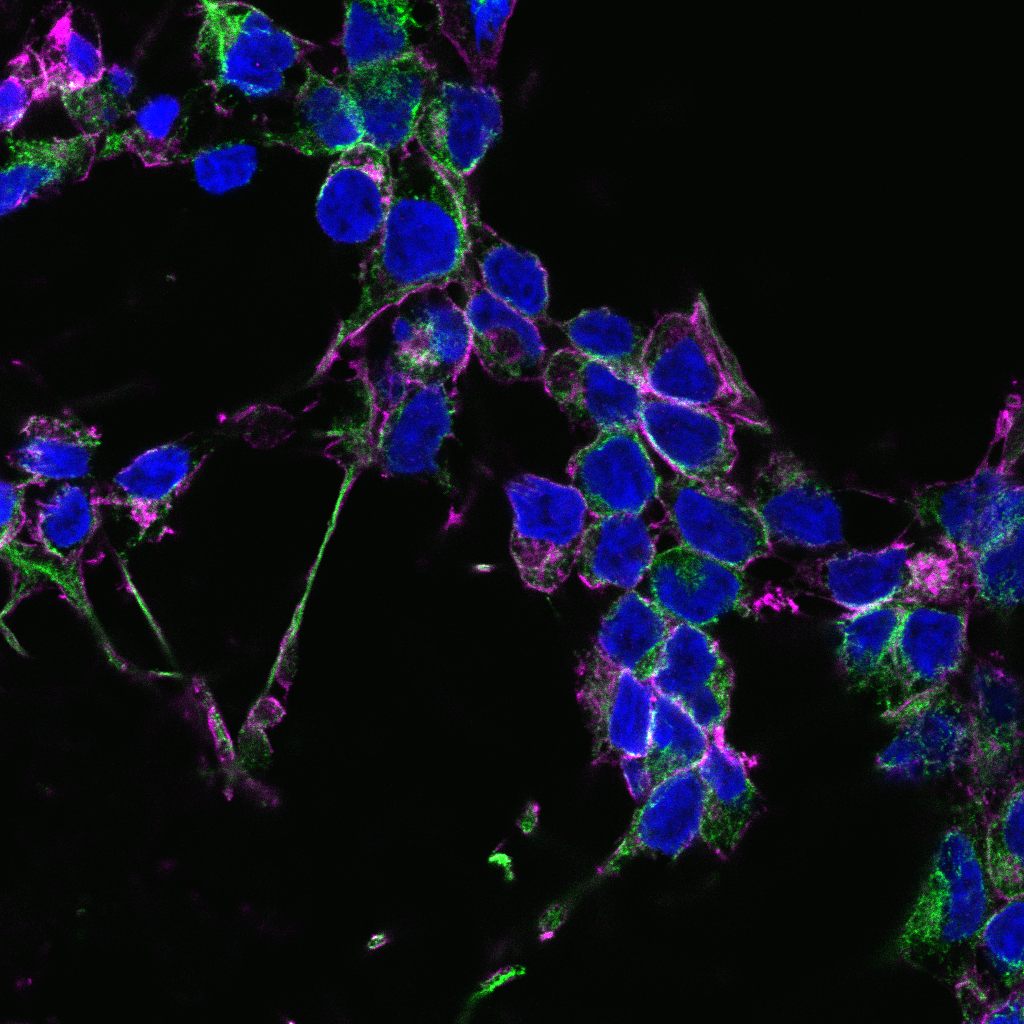

Supplement: Supplementary file 6 — Source data Fig. 3 [file 44318_2025_600_MOESM6_ESM.zip › Figure 3/3C/WT, Merge.tif]

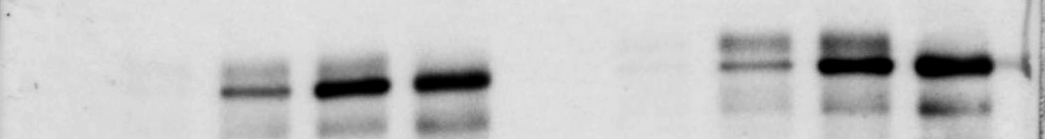

Supplement: Supplementary file 6 — Source data Fig. 3 [file 44318_2025_600_MOESM6_ESM.zip › Figure 3/3D/NR-K_pFGFR.tif]

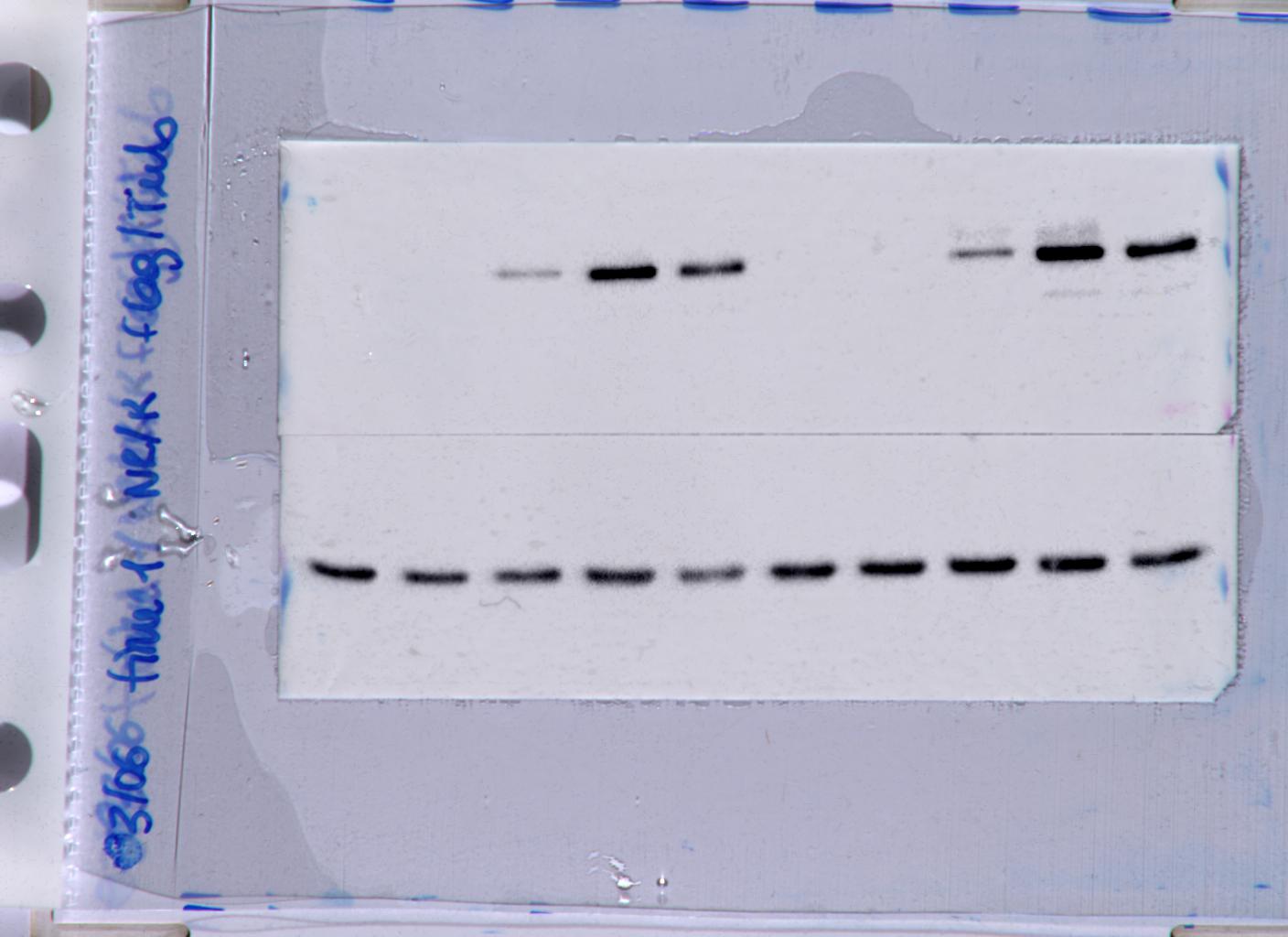

Supplement: Supplementary file 6 — Source data Fig. 3 [file 44318_2025_600_MOESM6_ESM.zip › Figure 3/3D/flag+tub NR, K original.jpg]

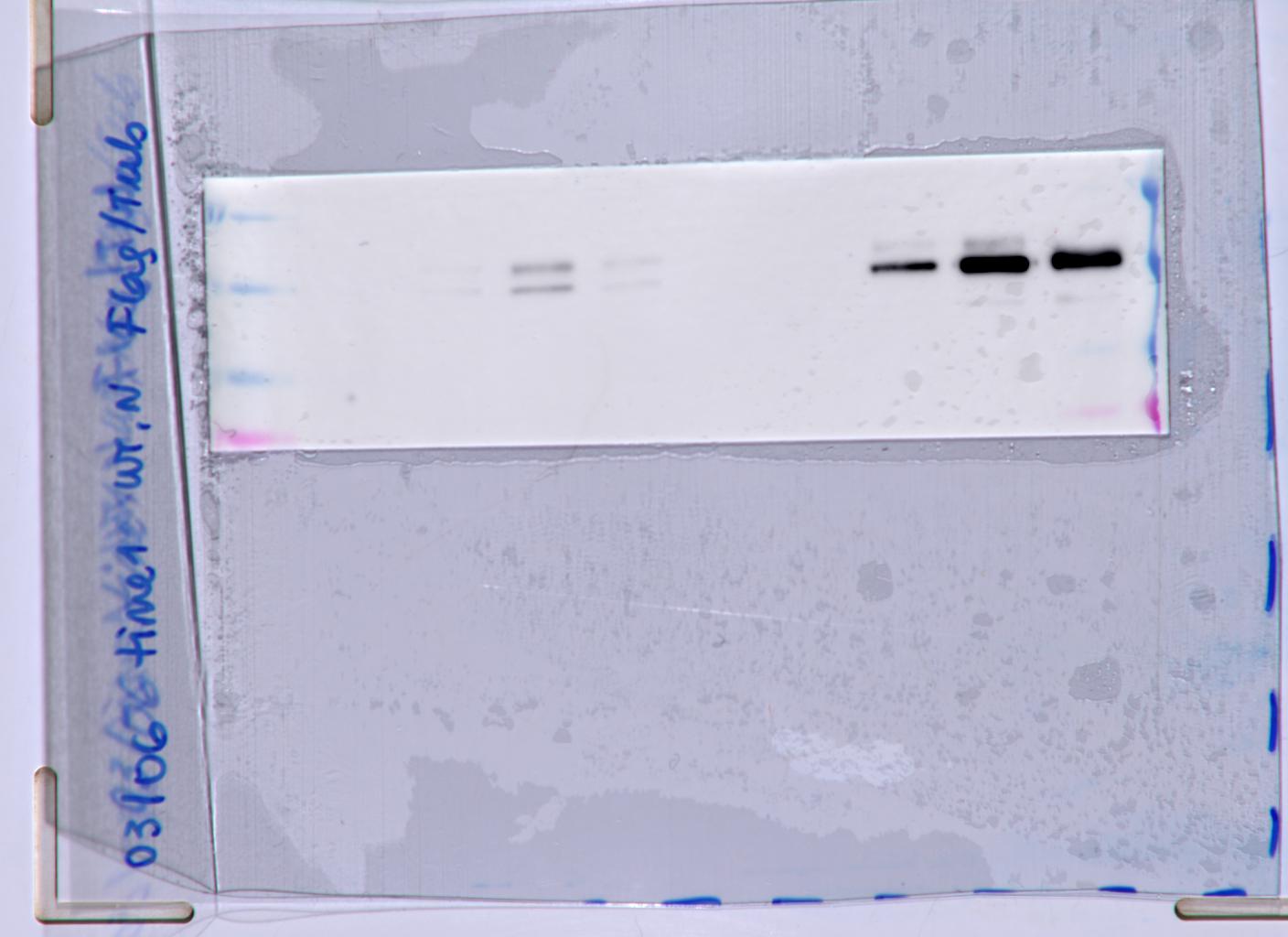

Supplement: Supplementary file 6 — Source data Fig. 3 [file 44318_2025_600_MOESM6_ESM.zip › Figure 3/3D/Flag WT, N original.jpg]

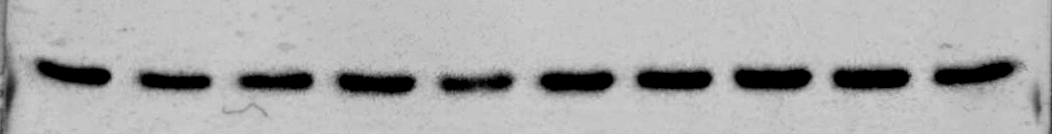

Supplement: Supplementary file 6 — Source data Fig. 3 [file 44318_2025_600_MOESM6_ESM.zip › Figure 3/3D/NR-K_tub.tif]

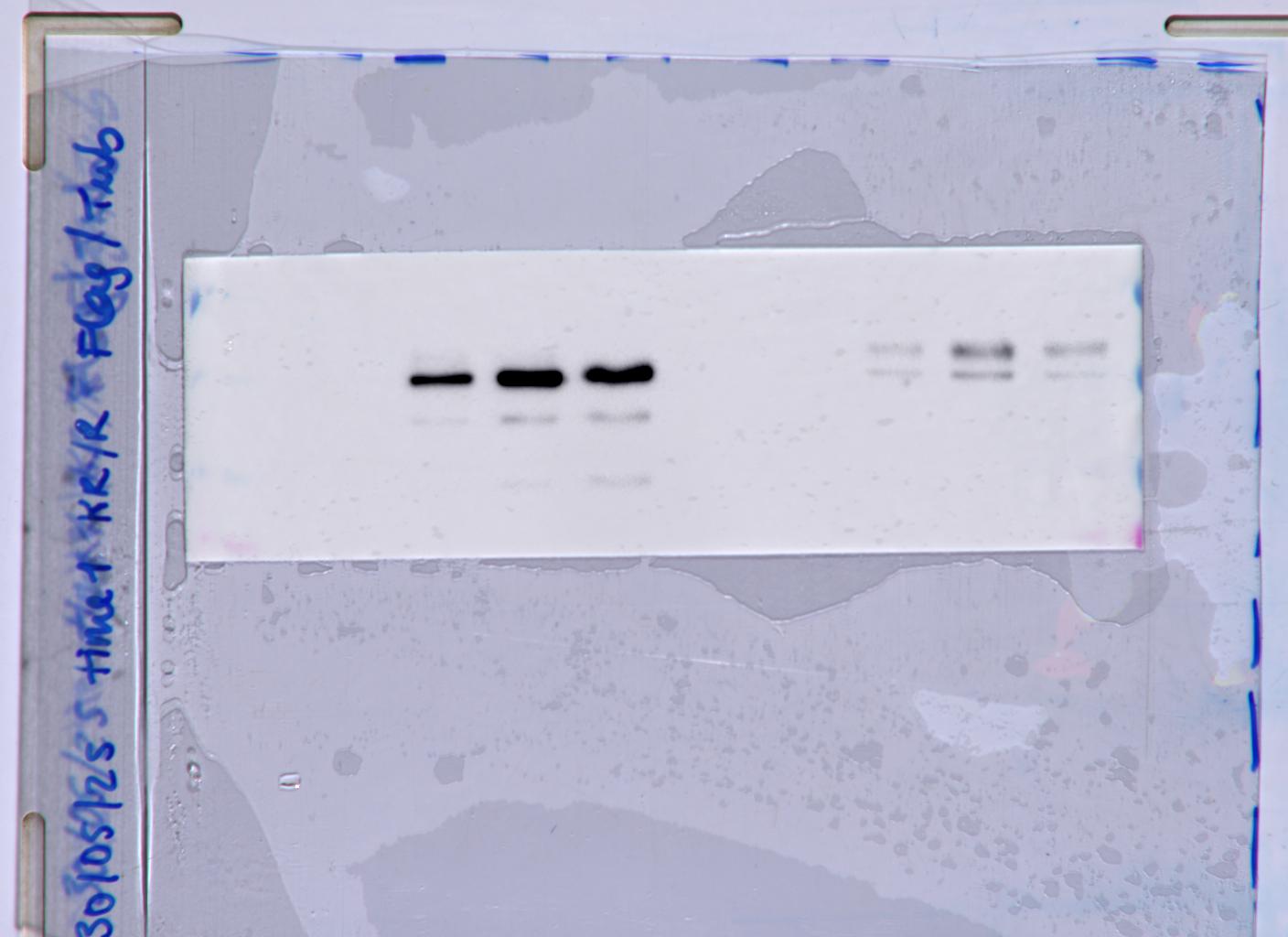

Supplement: Supplementary file 6 — Source data Fig. 3 [file 44318_2025_600_MOESM6_ESM.zip › Figure 3/3D/flag KR, R original.jpg]

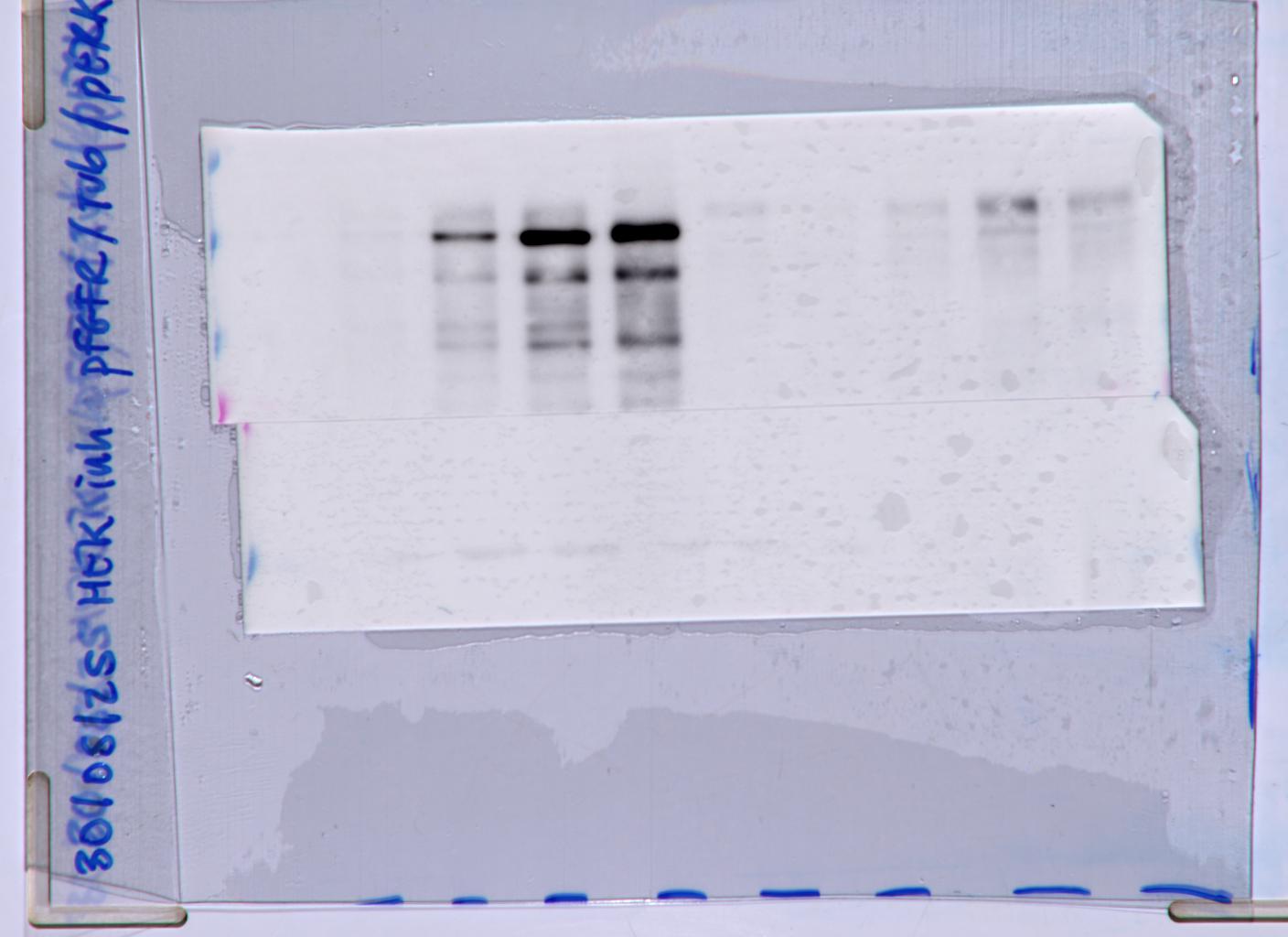

Supplement: Supplementary file 6 — Source data Fig. 3 [file 44318_2025_600_MOESM6_ESM.zip › Figure 3/3D/pFGFR1 KR, R original.jpg]

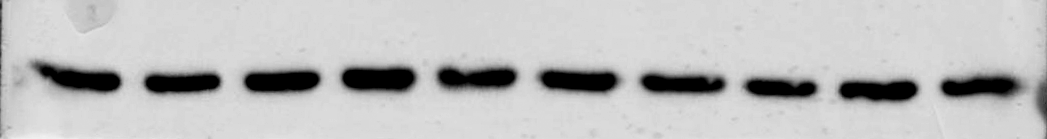

Supplement: Supplementary file 6 — Source data Fig. 3 [file 44318_2025_600_MOESM6_ESM.zip › Figure 3/3D/KR-R_Tub.tif]

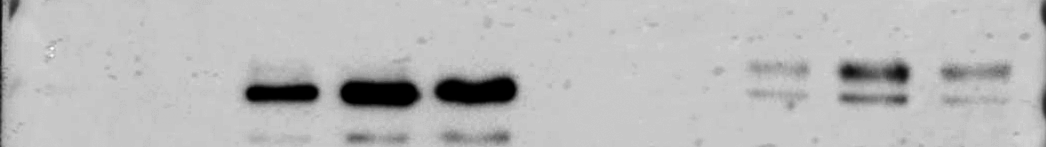

Supplement: Supplementary file 6 — Source data Fig. 3 [file 44318_2025_600_MOESM6_ESM.zip › Figure 3/3D/KR-R_Flag.tif]

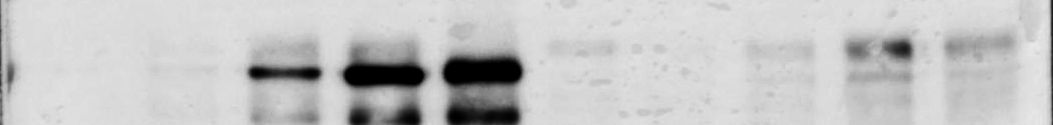

Supplement: Supplementary file 6 — Source data Fig. 3 [file 44318_2025_600_MOESM6_ESM.zip › Figure 3/3D/KR-R_pFGFR.tif]

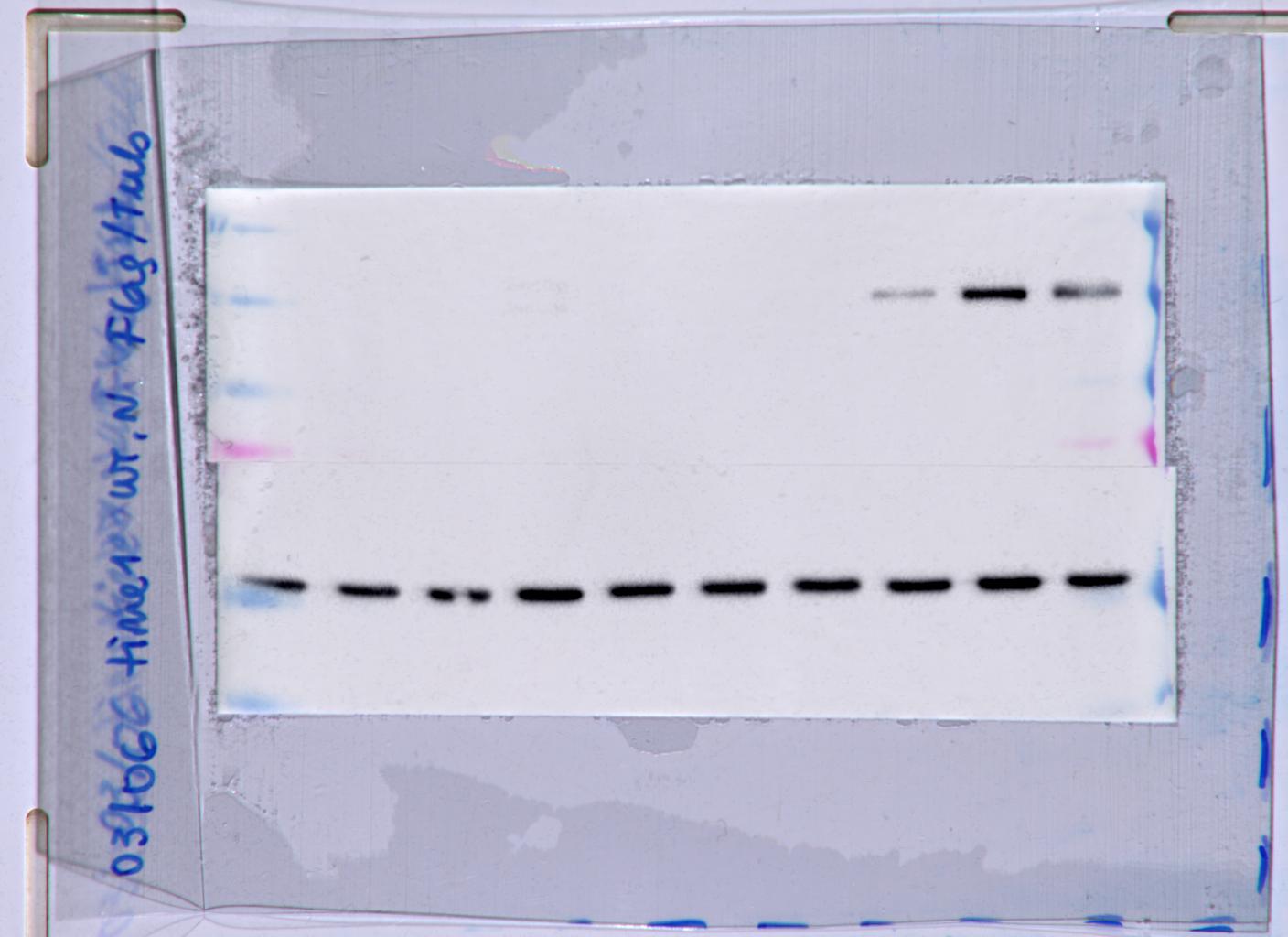

Supplement: Supplementary file 6 — Source data Fig. 3 [file 44318_2025_600_MOESM6_ESM.zip › Figure 3/3D/Tub WT, N original.jpg]

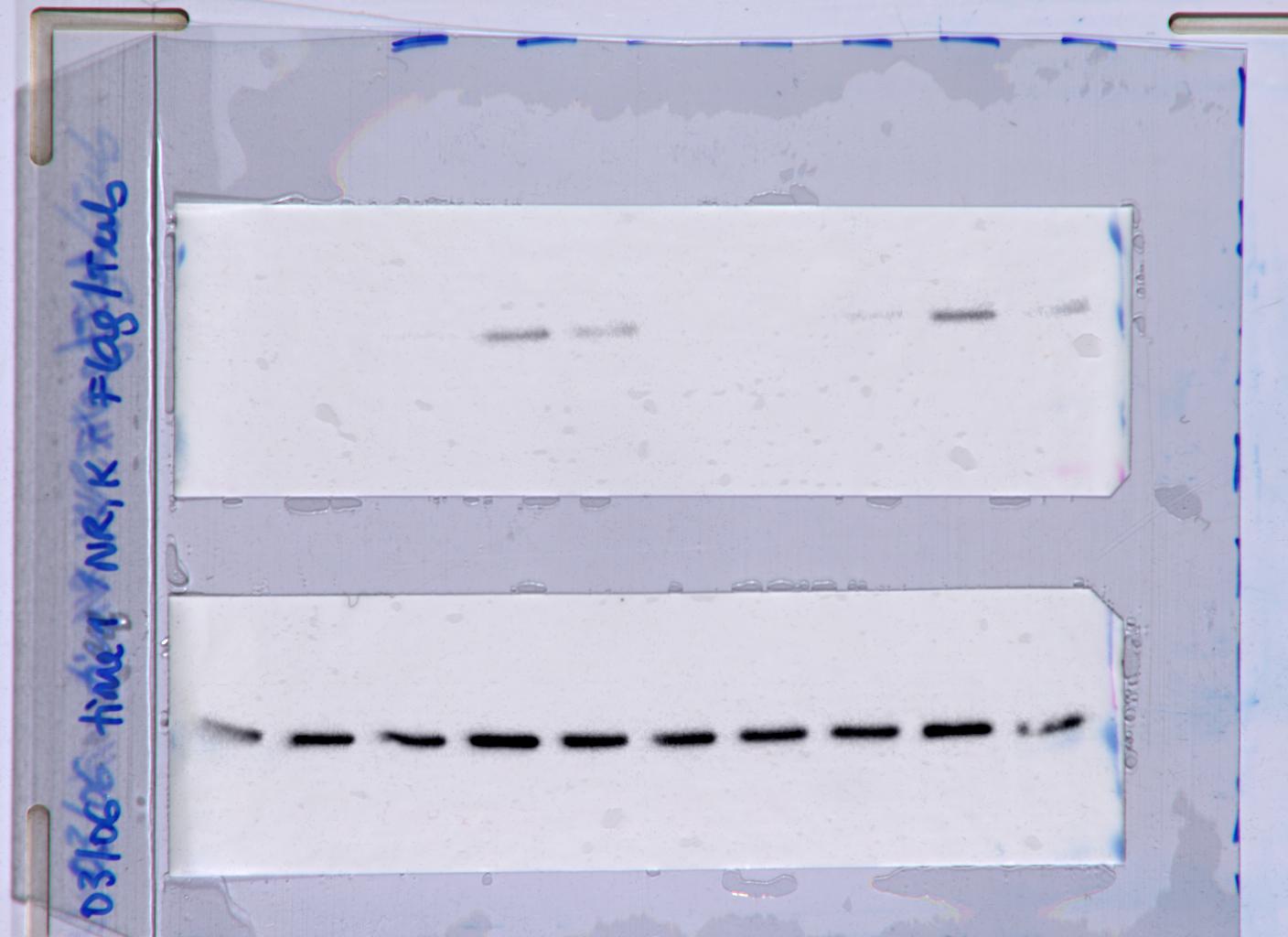

Supplement: Supplementary file 6 — Source data Fig. 3 [file 44318_2025_600_MOESM6_ESM.zip › Figure 3/3D/tub (pFGFR1 membrane) WT, N original.jpg]

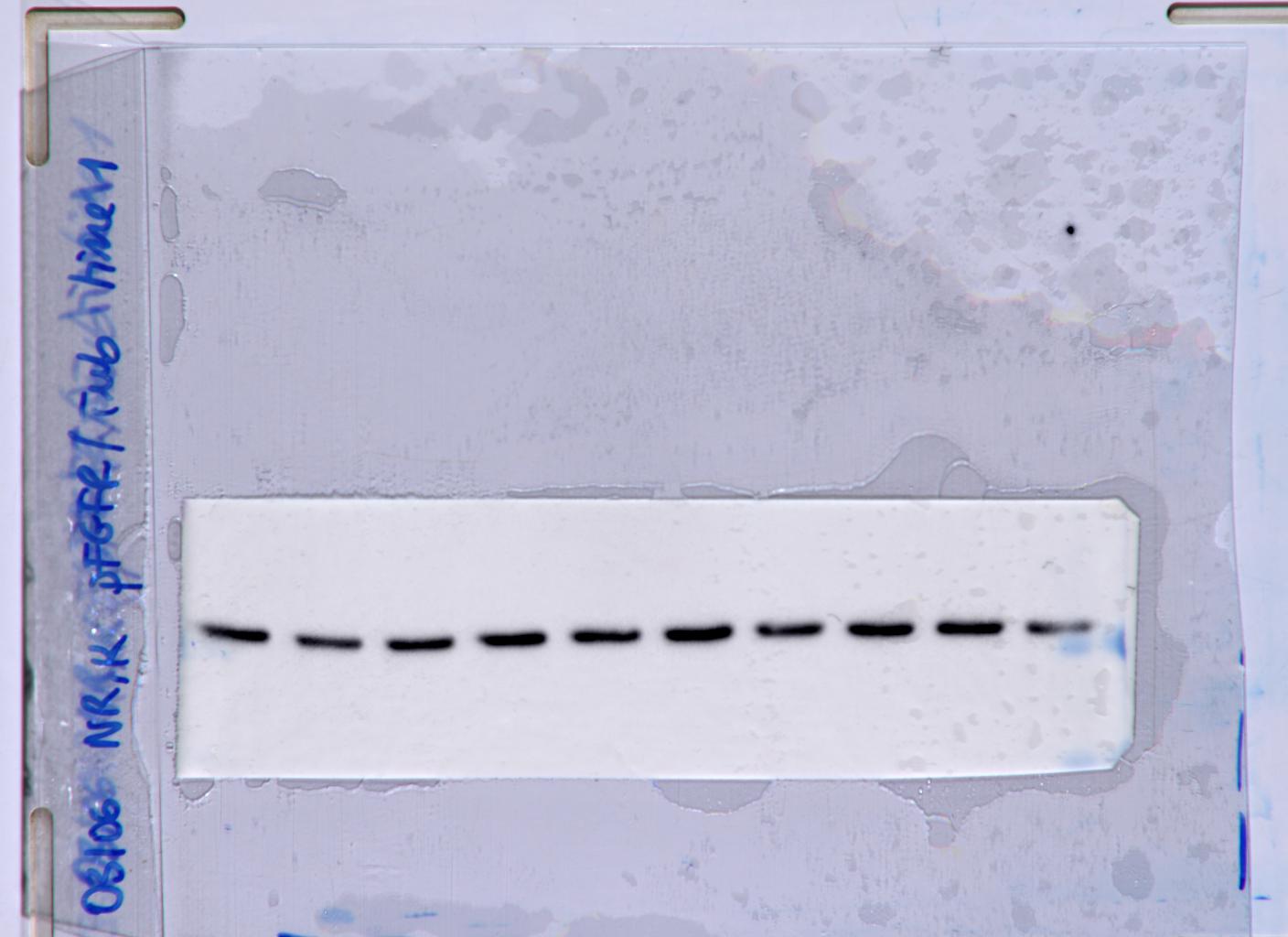

Supplement: Supplementary file 6 — Source data Fig. 3 [file 44318_2025_600_MOESM6_ESM.zip › Figure 3/3D/tub (pFGFR1 membrane) NR, K original.jpg]

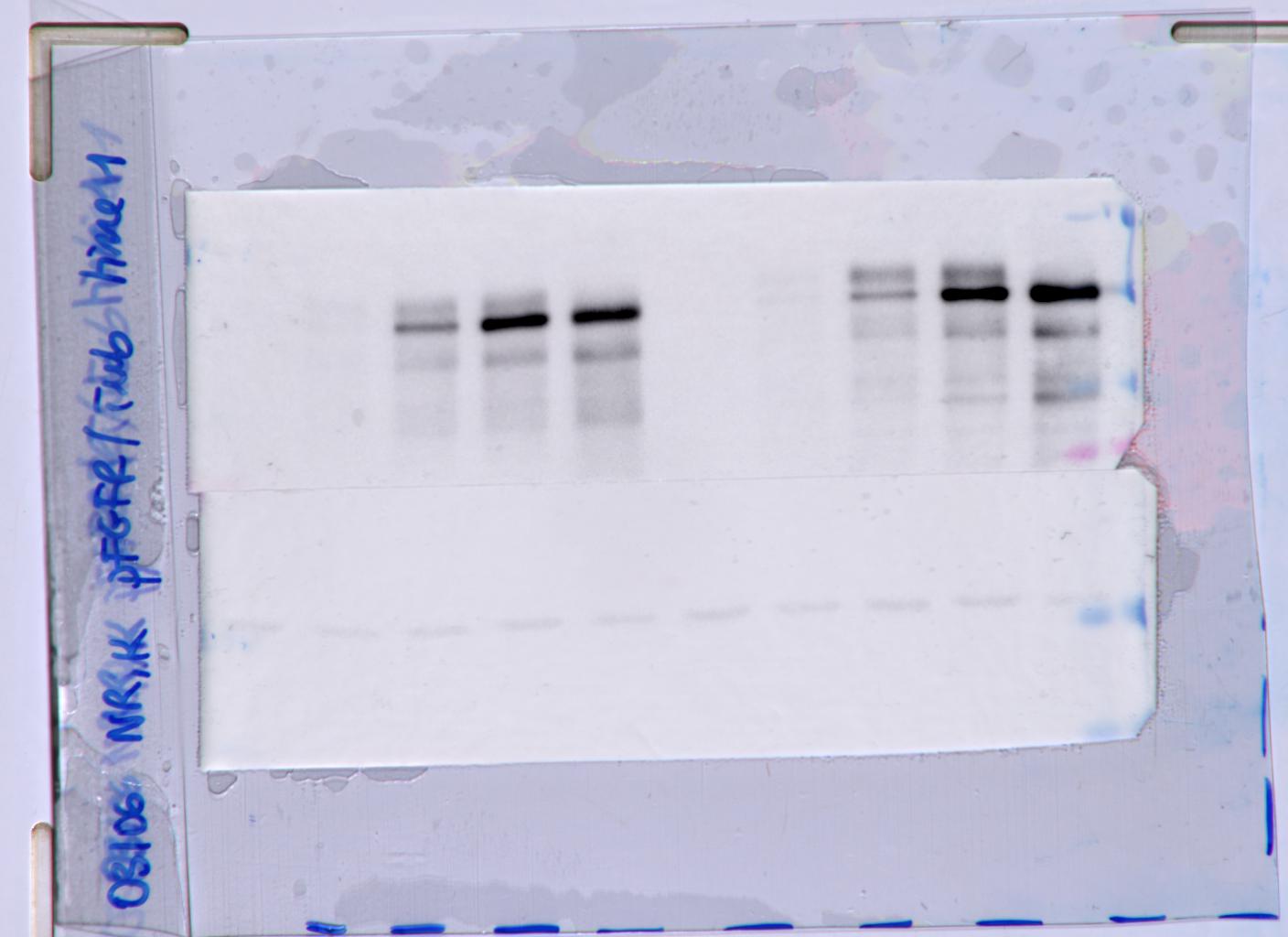

Supplement: Supplementary file 6 — Source data Fig. 3 [file 44318_2025_600_MOESM6_ESM.zip › Figure 3/3D/pFGFR1 NR, K original.jpg]

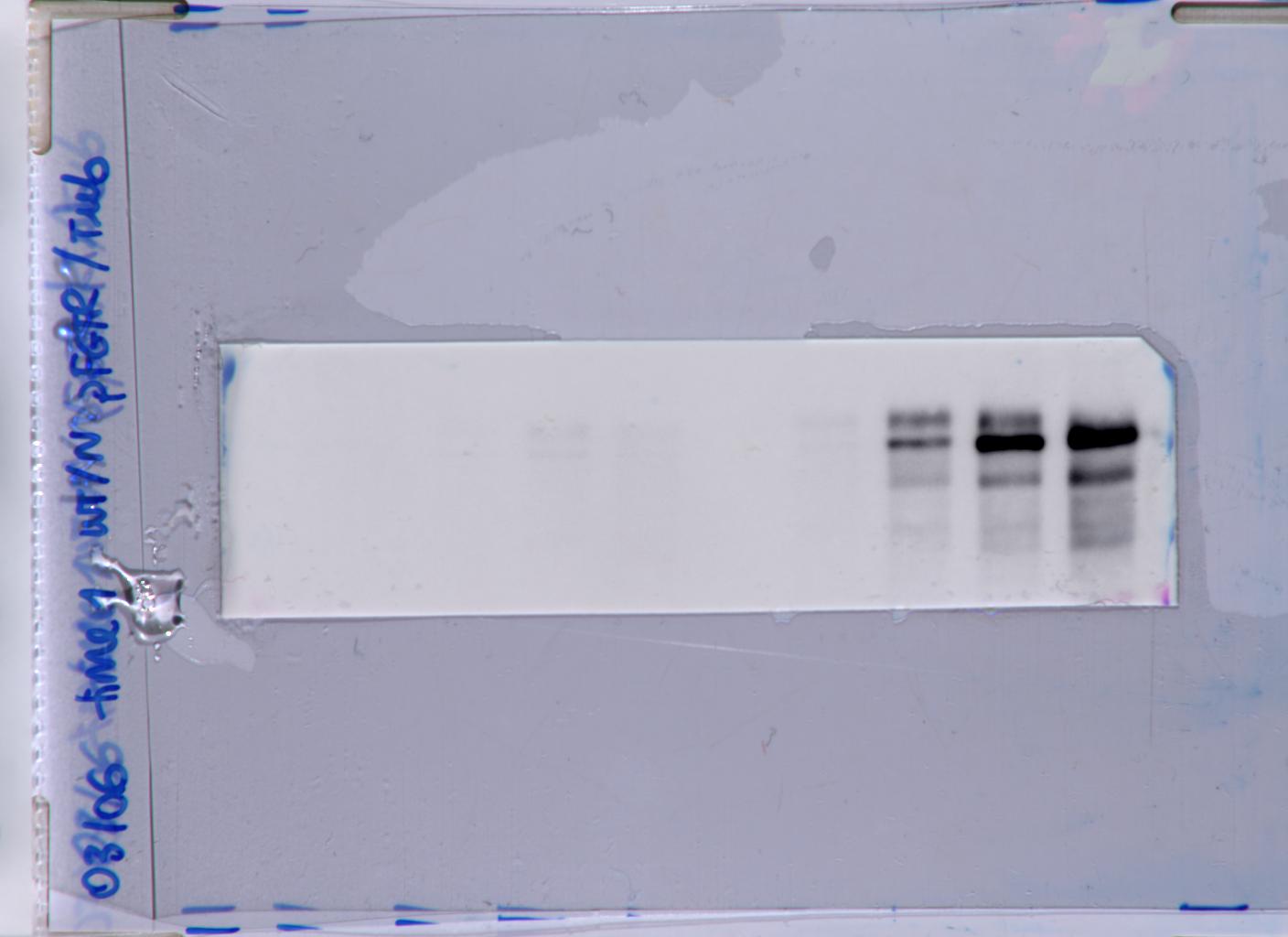

Supplement: Supplementary file 6 — Source data Fig. 3 [file 44318_2025_600_MOESM6_ESM.zip › Figure 3/3D/pFGFR1 WT, N original.jpg]

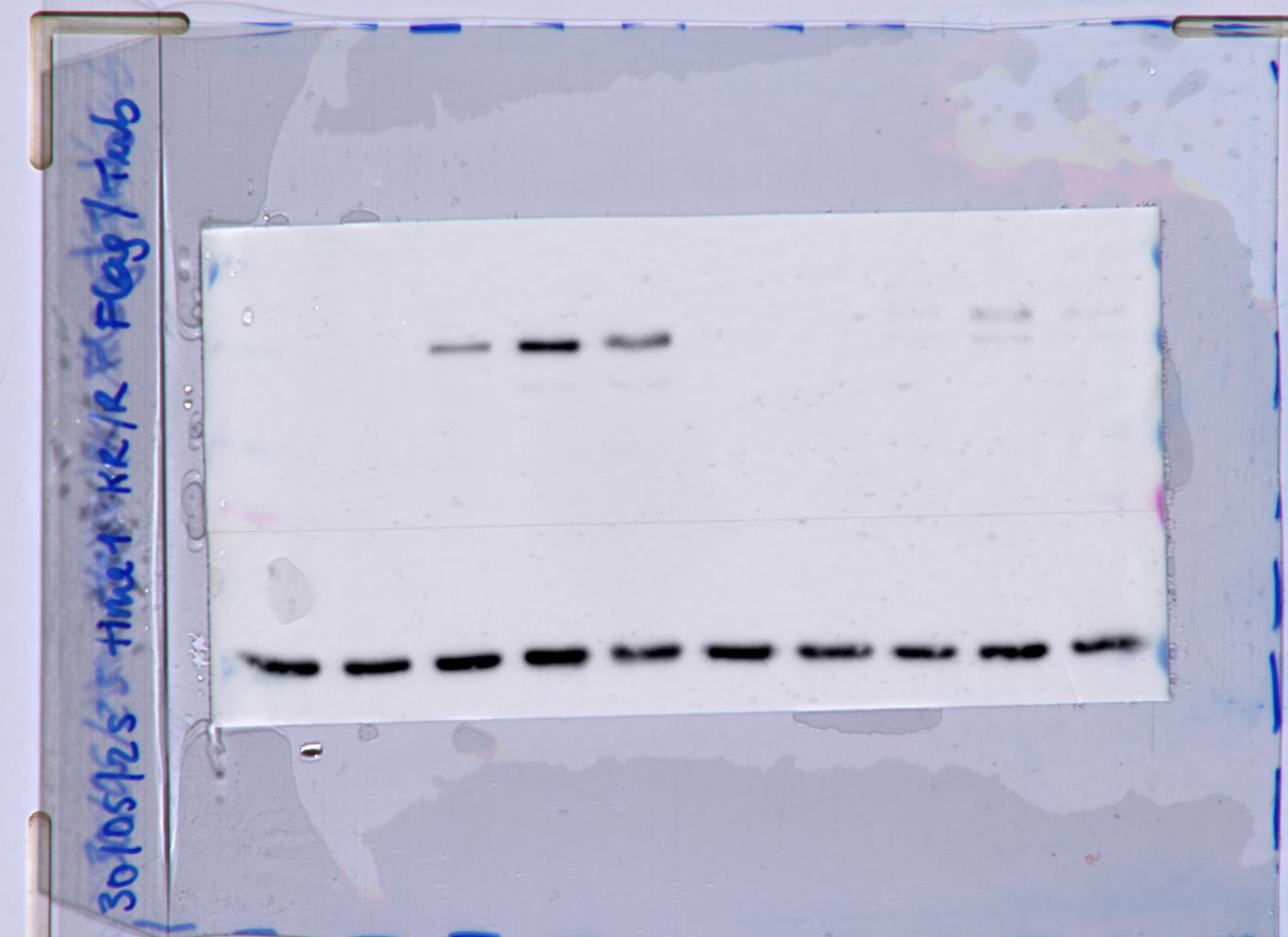

Supplement: Supplementary file 6 — Source data Fig. 3 [file 44318_2025_600_MOESM6_ESM.zip › Figure 3/3D/tub KR, R original.jpg]

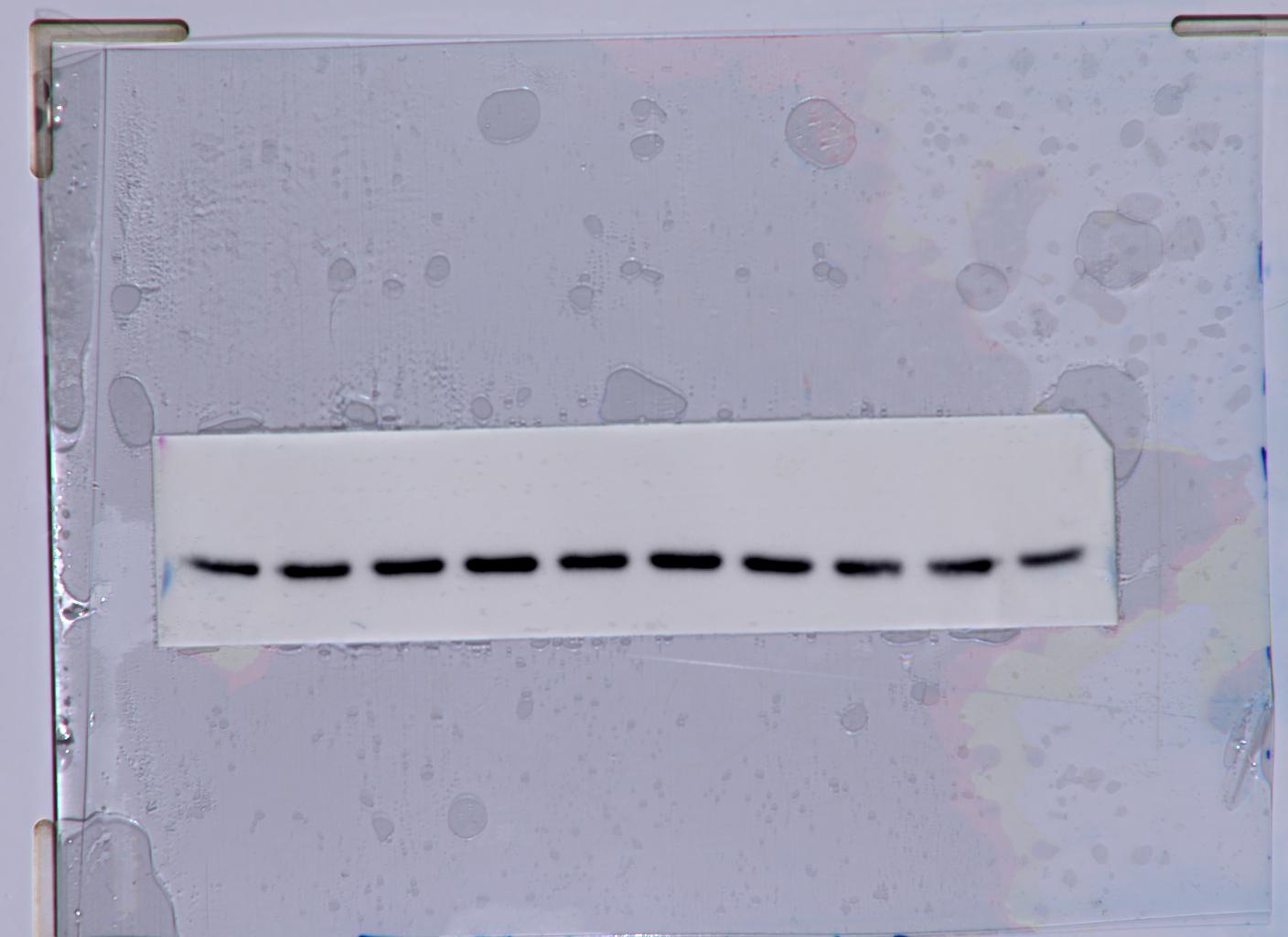

Supplement: Supplementary file 6 — Source data Fig. 3 [file 44318_2025_600_MOESM6_ESM.zip › Figure 3/3D/tub (pFGFR1 membrane) KR, R original.jpg]

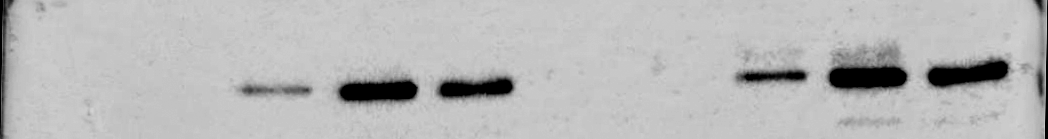

Supplement: Supplementary file 6 — Source data Fig. 3 [file 44318_2025_600_MOESM6_ESM.zip › Figure 3/3D/NR-K_Flag.tif]

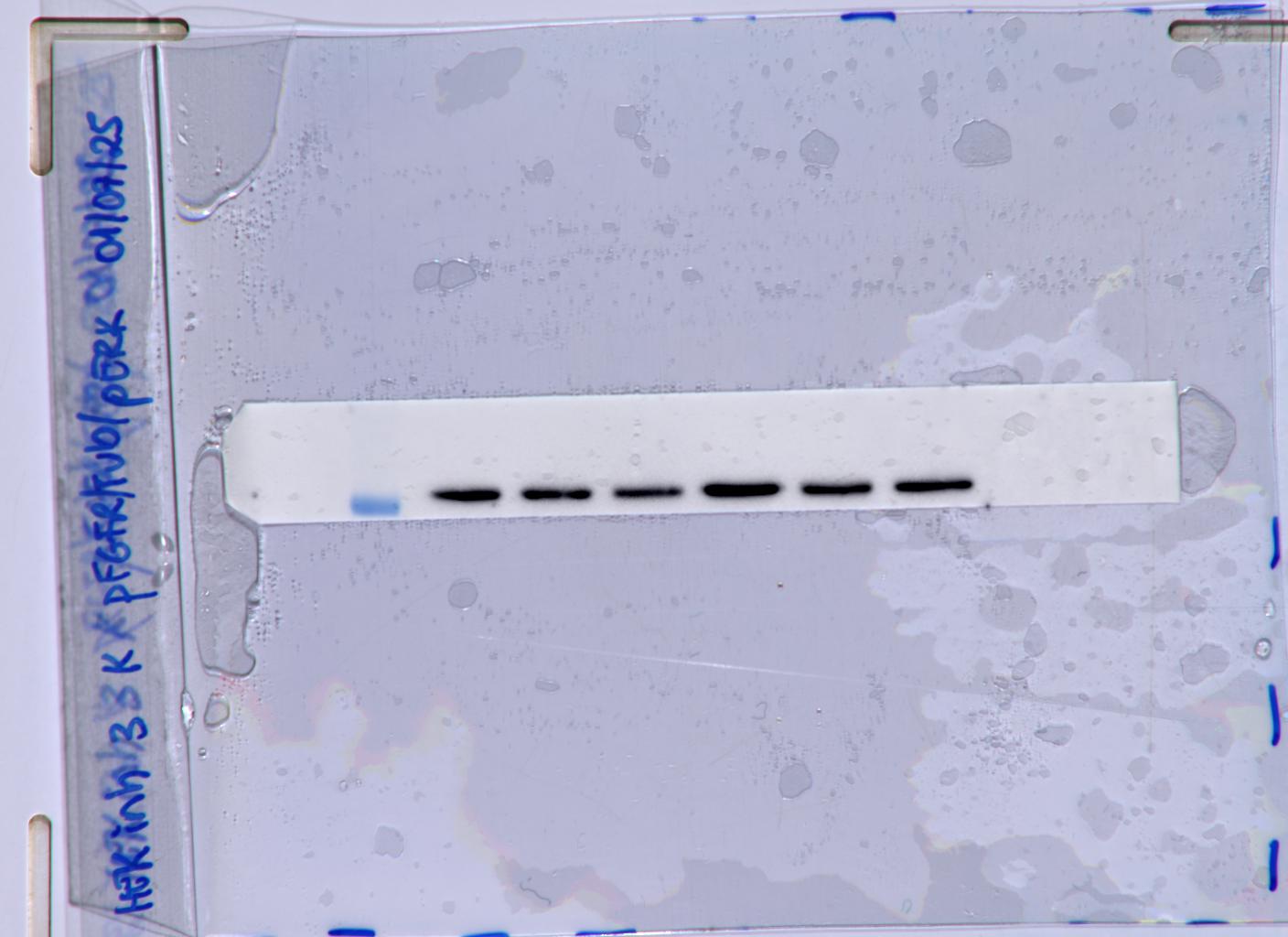

Supplement: Supplementary file 6 — Source data Fig. 3 [file 44318_2025_600_MOESM6_ESM.zip › Figure 3/3E/3E replicates/inh3k ptub 5s 2025.07.01_12.20.38_Ch+Marker.jpg]

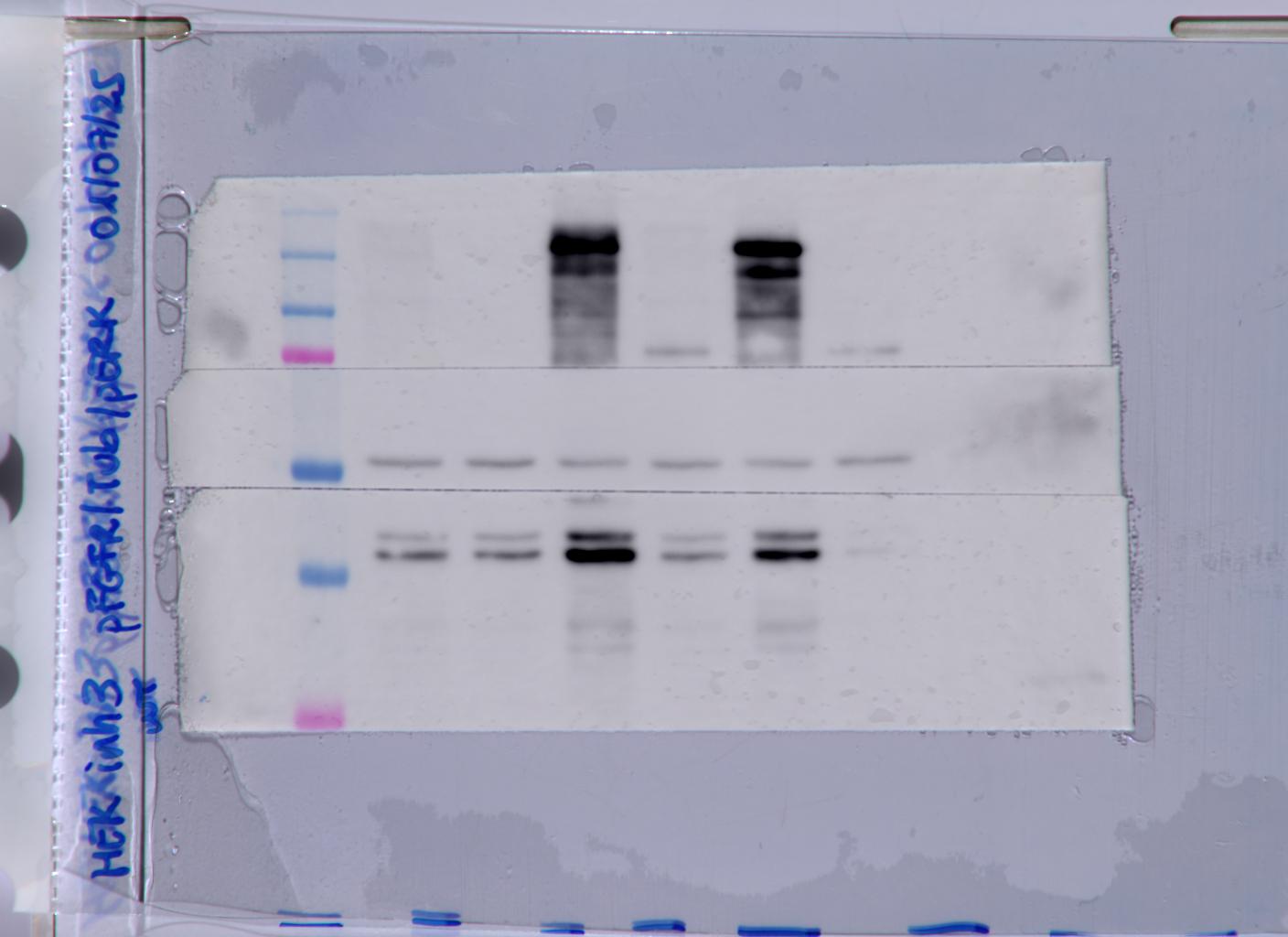

Supplement: Supplementary file 6 — Source data Fig. 3 [file 44318_2025_600_MOESM6_ESM.zip › Figure 3/3E/3E replicates/inh3wt pffrtbpek 1.5 2025.07.01_12.06.02_Ch+Marker.jpg]

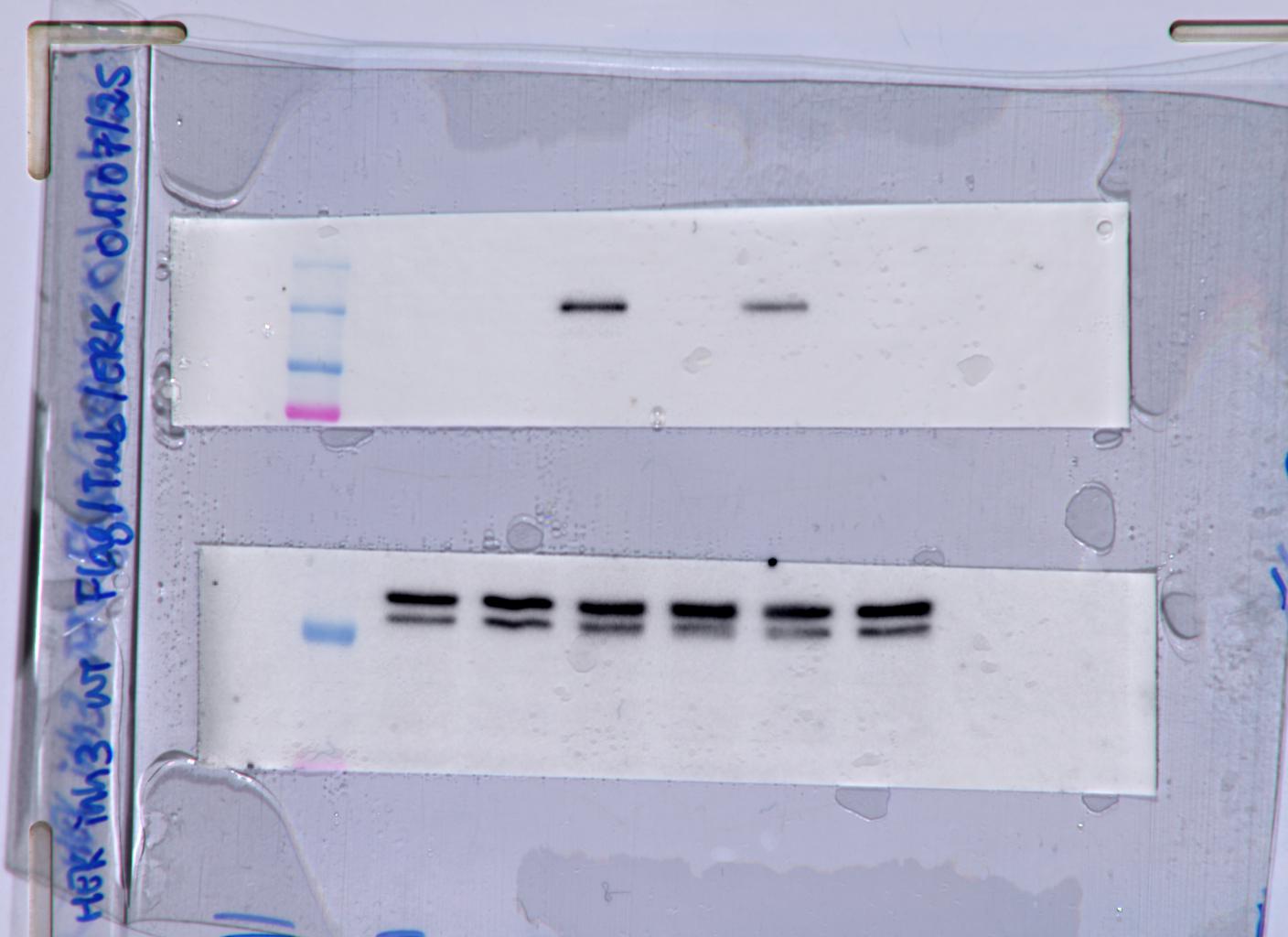

Supplement: Supplementary file 6 — Source data Fig. 3 [file 44318_2025_600_MOESM6_ESM.zip › Figure 3/3E/3E replicates/inh3wt erk 2s 2025.07.01_12.27.06_Ch+Marker.jpg]

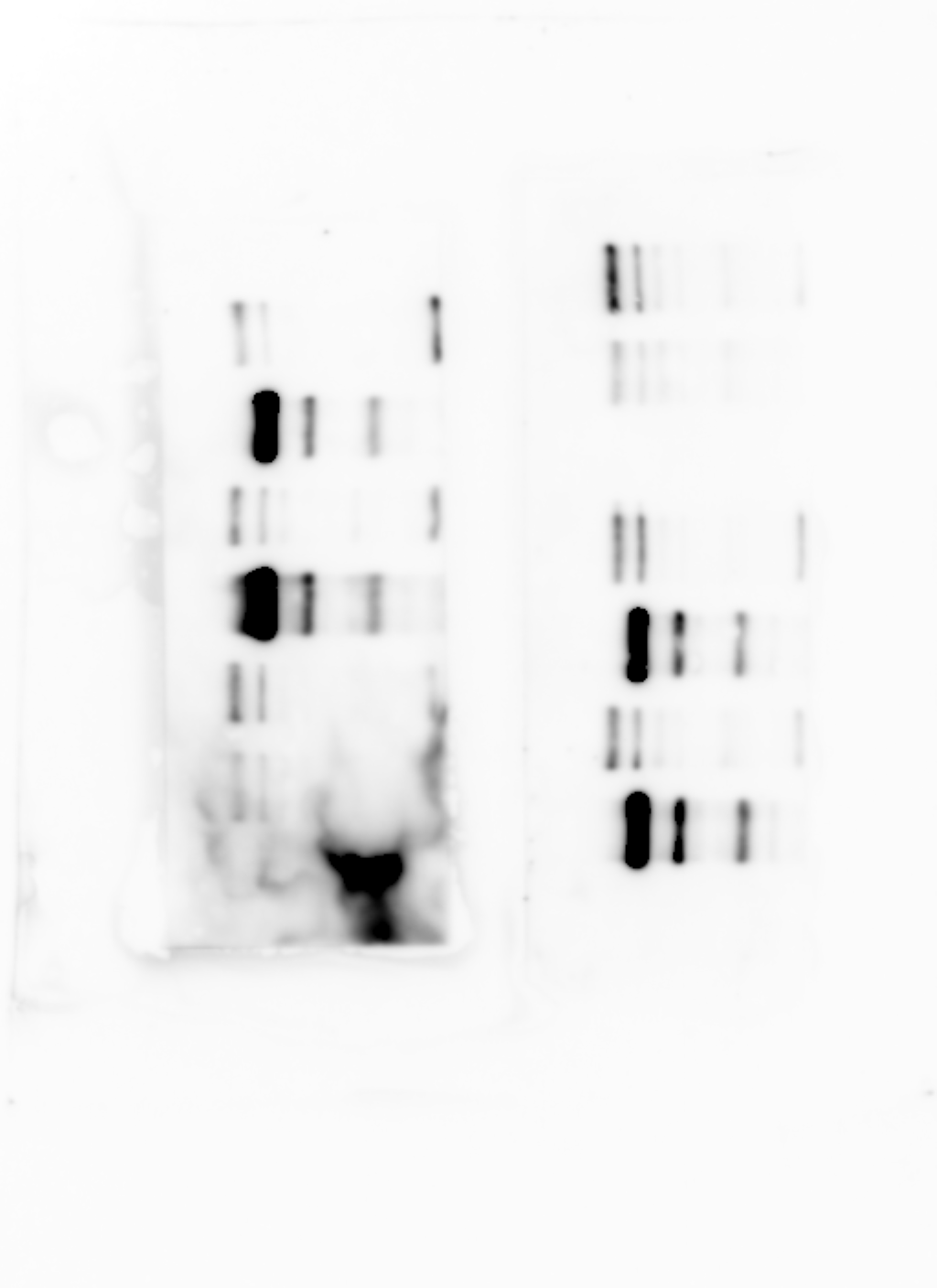

Supplement: Supplementary file 6 — Source data Fig. 3 [file 44318_2025_600_MOESM6_ESM.zip › Figure 3/3E/3E replicates/inh3 flag inc.extrem 2025.07.10_15.22.51-12_Ch.tif]

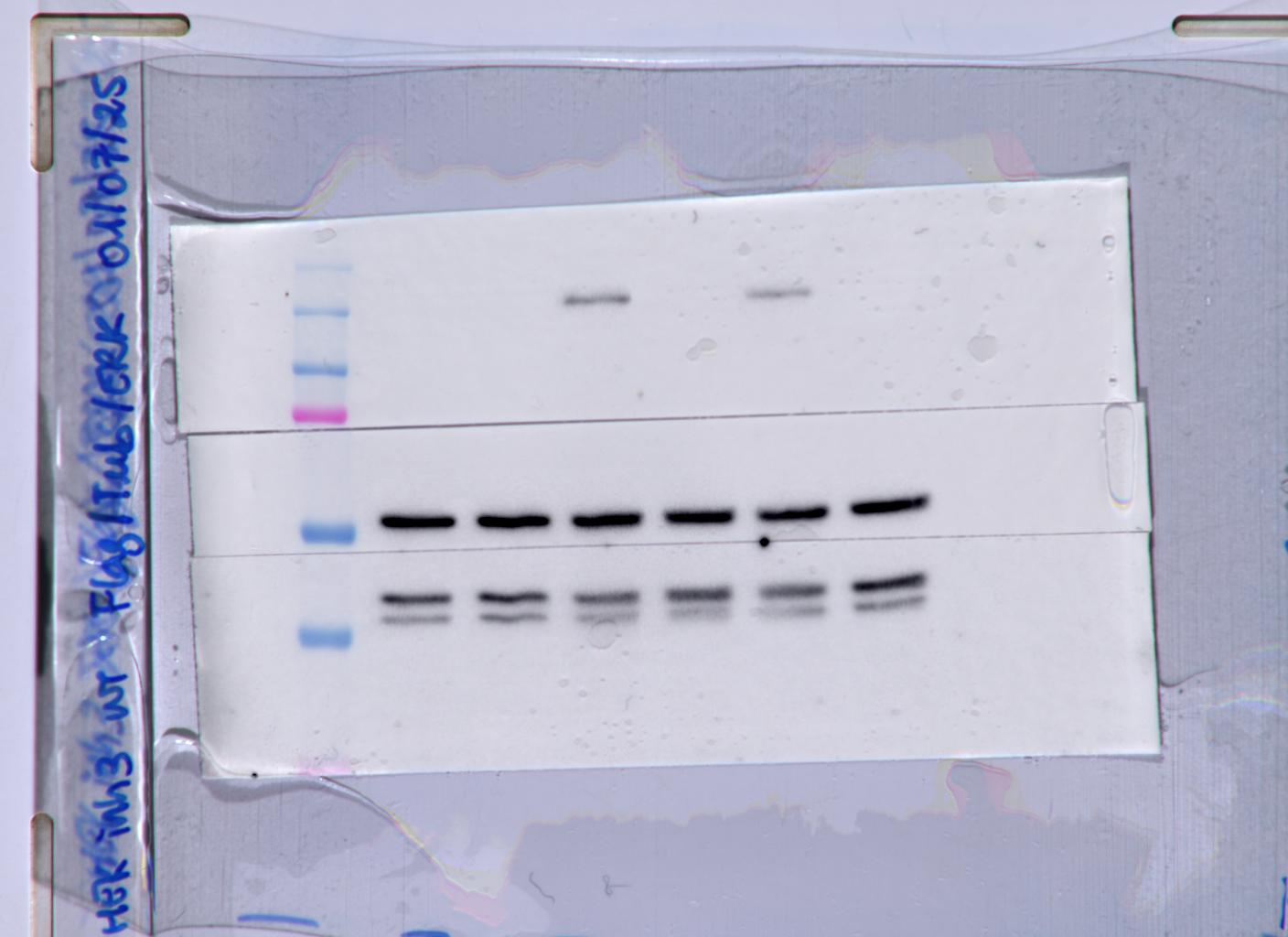

Supplement: Supplementary file 6 — Source data Fig. 3 [file 44318_2025_600_MOESM6_ESM.zip › Figure 3/3E/3E replicates/inh3wt tub 2s 2025.07.01_12.25.28_Ch+Marker.jpg]

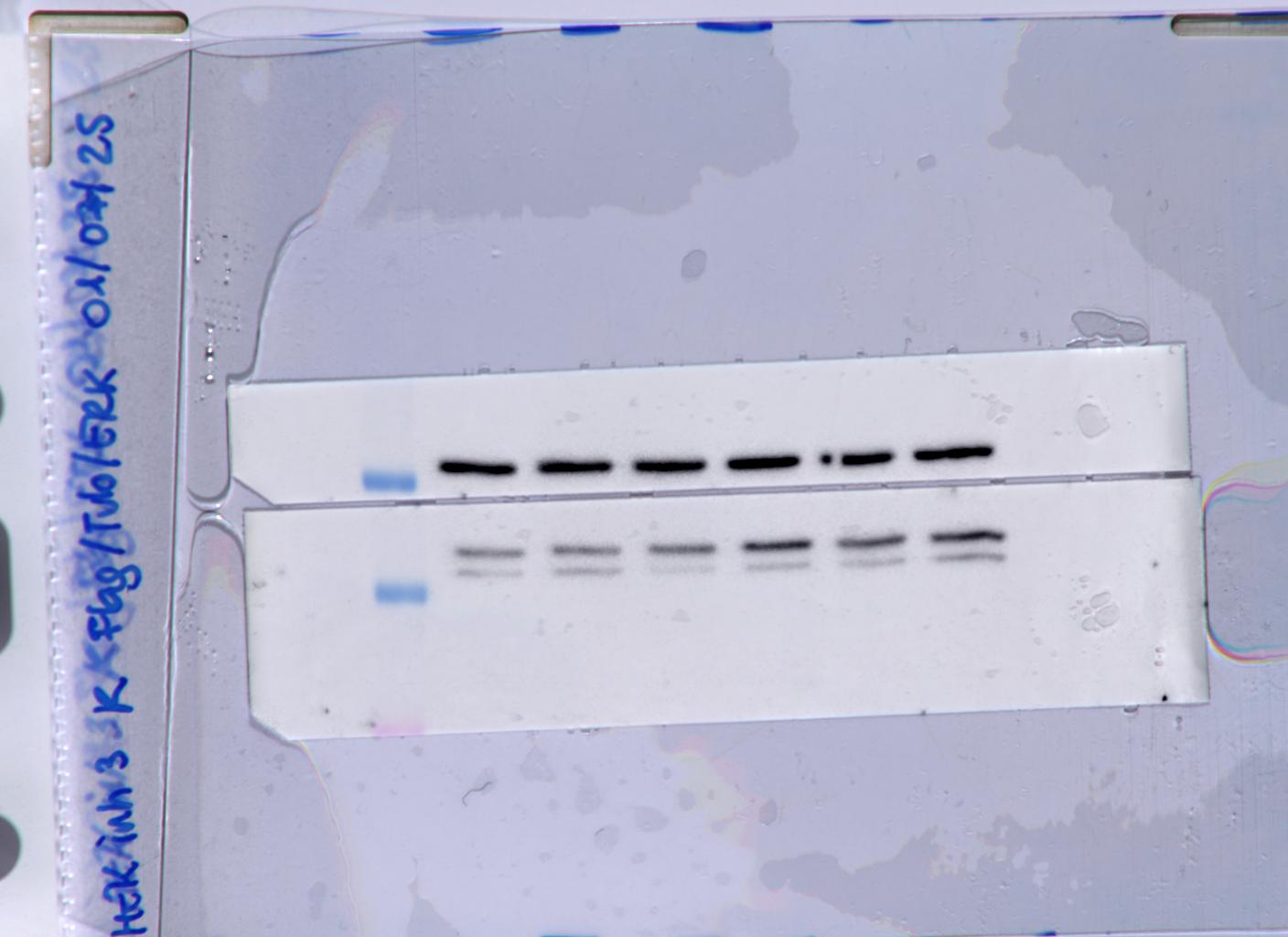

Supplement: Supplementary file 6 — Source data Fig. 3 [file 44318_2025_600_MOESM6_ESM.zip › Figure 3/3E/3E replicates/inh3k tub 2s 2025.07.01_12.31.44_Ch+Marker.jpg]

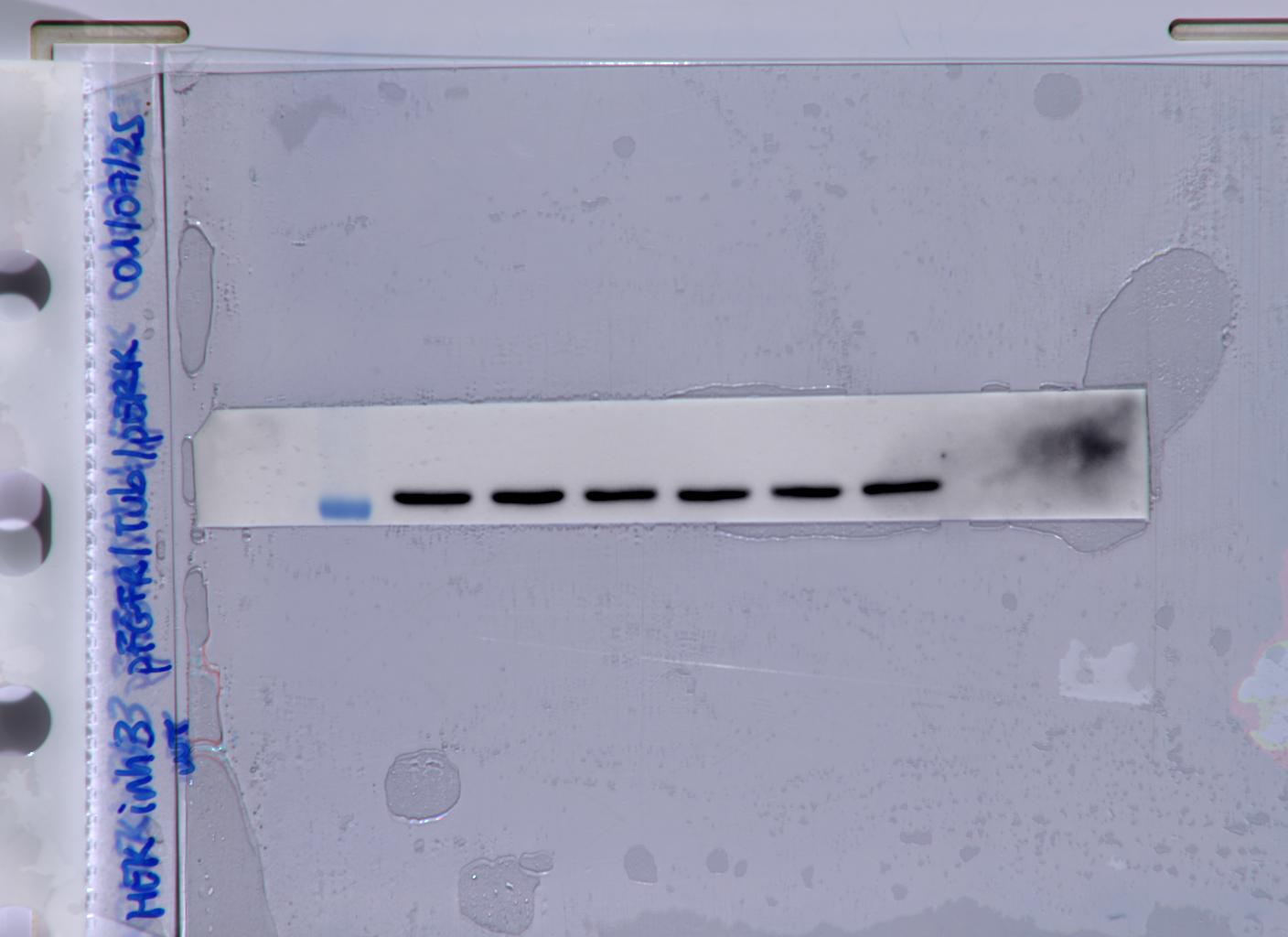

Supplement: Supplementary file 6 — Source data Fig. 3 [file 44318_2025_600_MOESM6_ESM.zip › Figure 3/3E/3E replicates/inh3wt ptub 5s 2025.07.01_12.08.57_Ch+Marker.jpg]

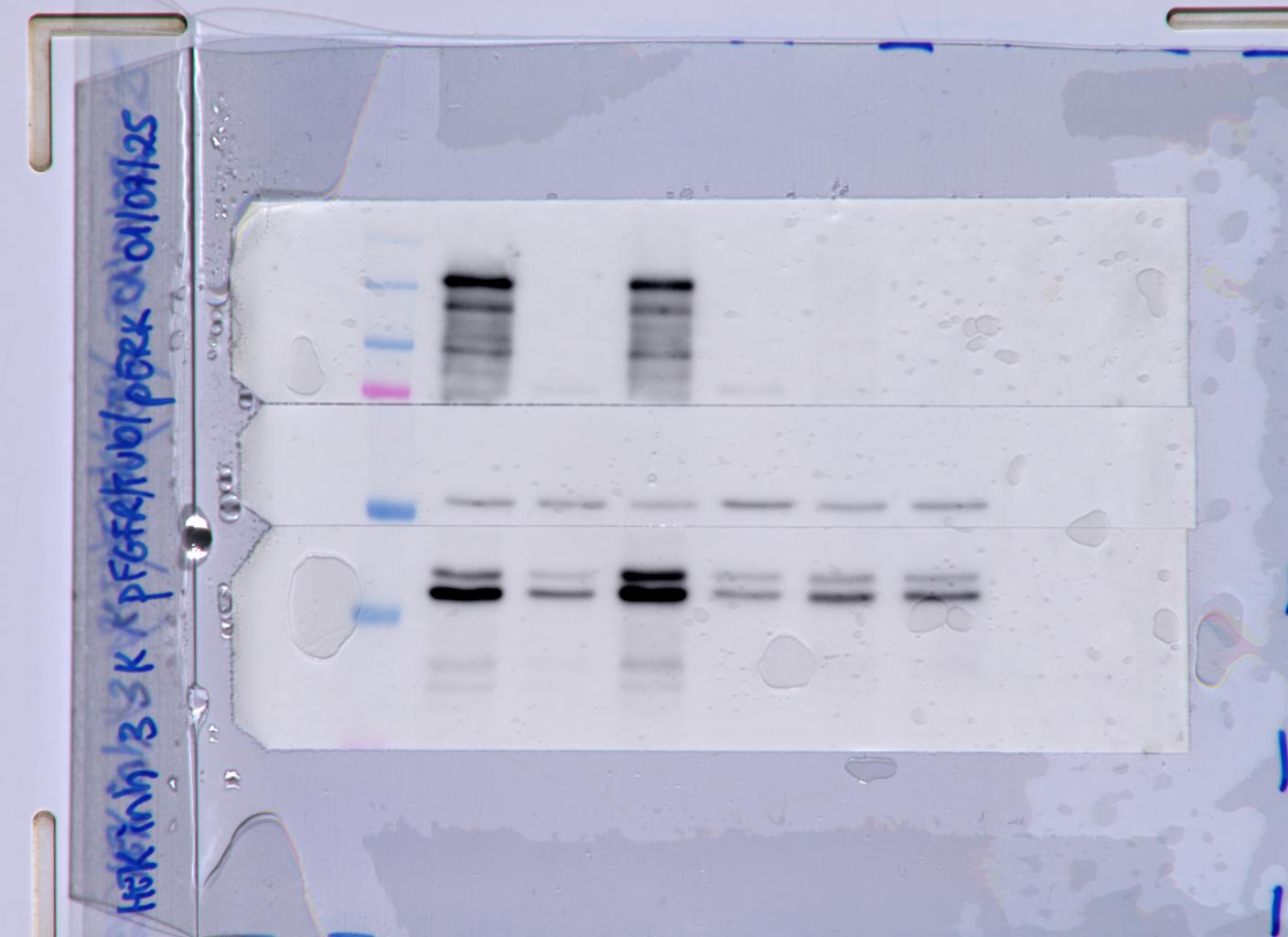

Supplement: Supplementary file 6 — Source data Fig. 3 [file 44318_2025_600_MOESM6_ESM.zip › Figure 3/3E/3E replicates/inh3k pgfrtbprk 1.5 2025.07.01_12.17.57_Ch+Marker.jpg]

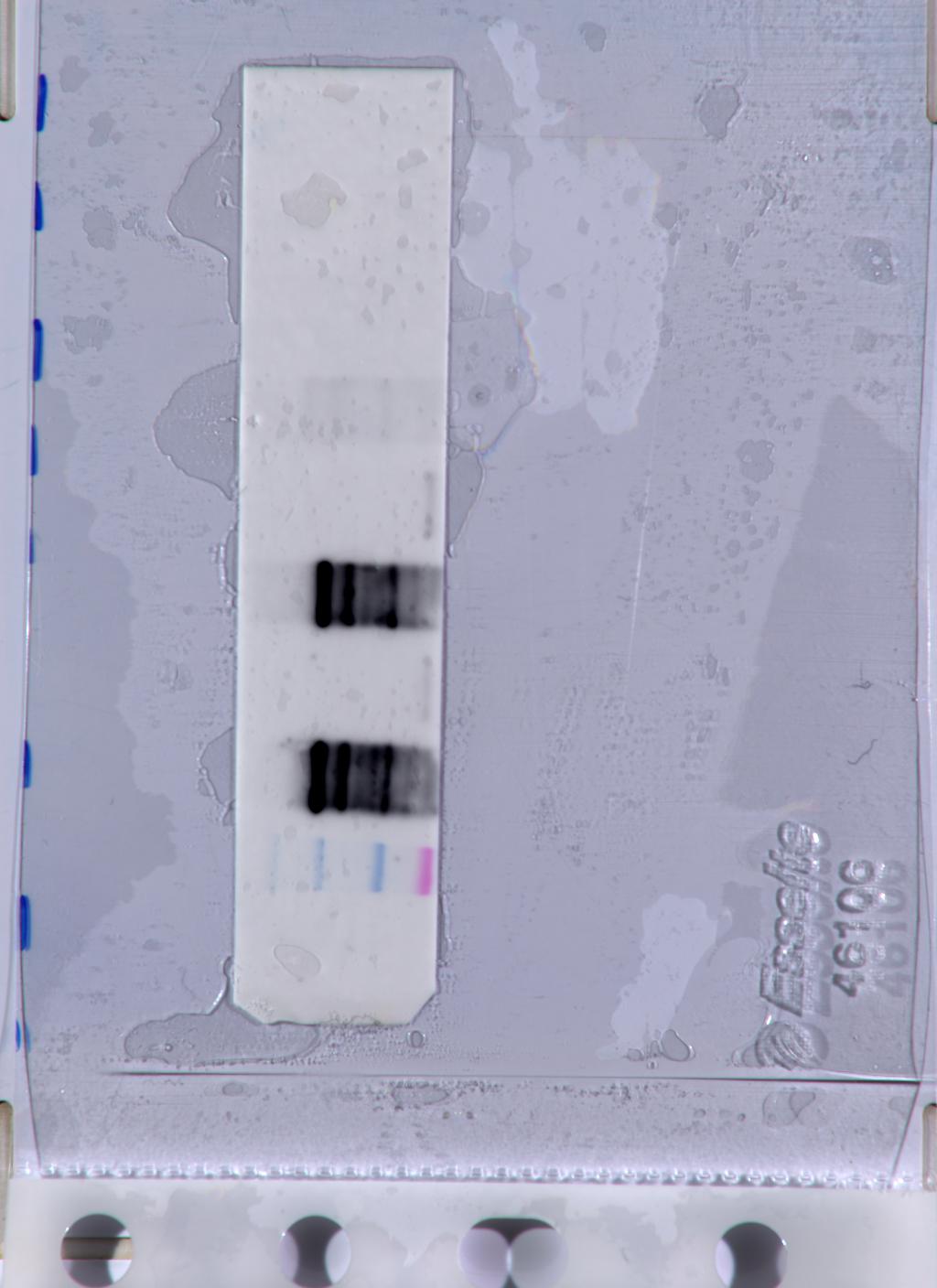

Supplement: Supplementary file 6 — Source data Fig. 3 [file 44318_2025_600_MOESM6_ESM.zip › Figure 3/3E/3E replicates/inh3k pfgfr 15s 2025.07.01_12.22.02_Ch+Marker.jpg]

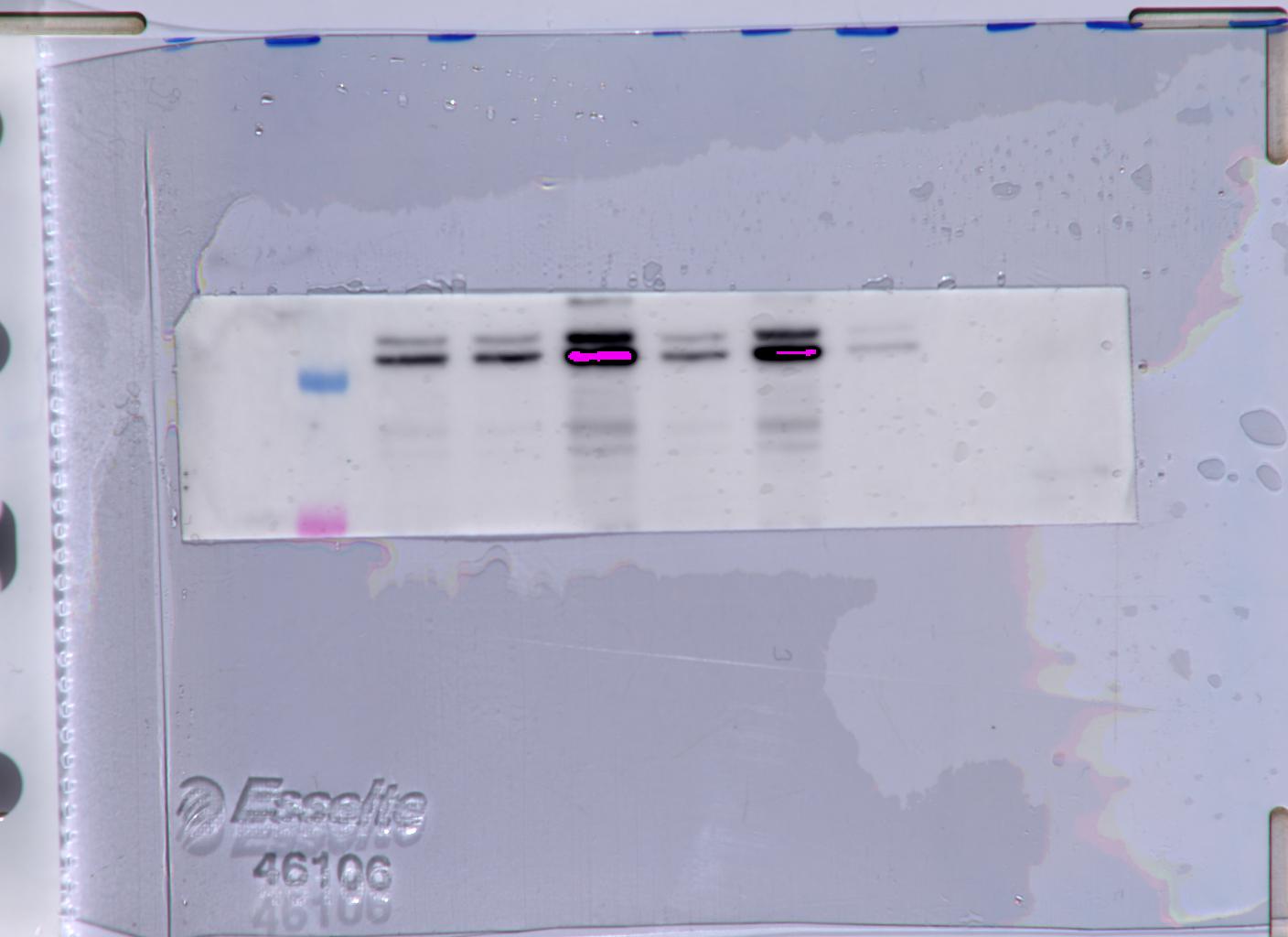

Supplement: Supplementary file 6 — Source data Fig. 3 [file 44318_2025_600_MOESM6_ESM.zip › Figure 3/3E/3E replicates/inh3wt perk 10s 2025.07.01_12.12.47_Ch+Marker.jpg]

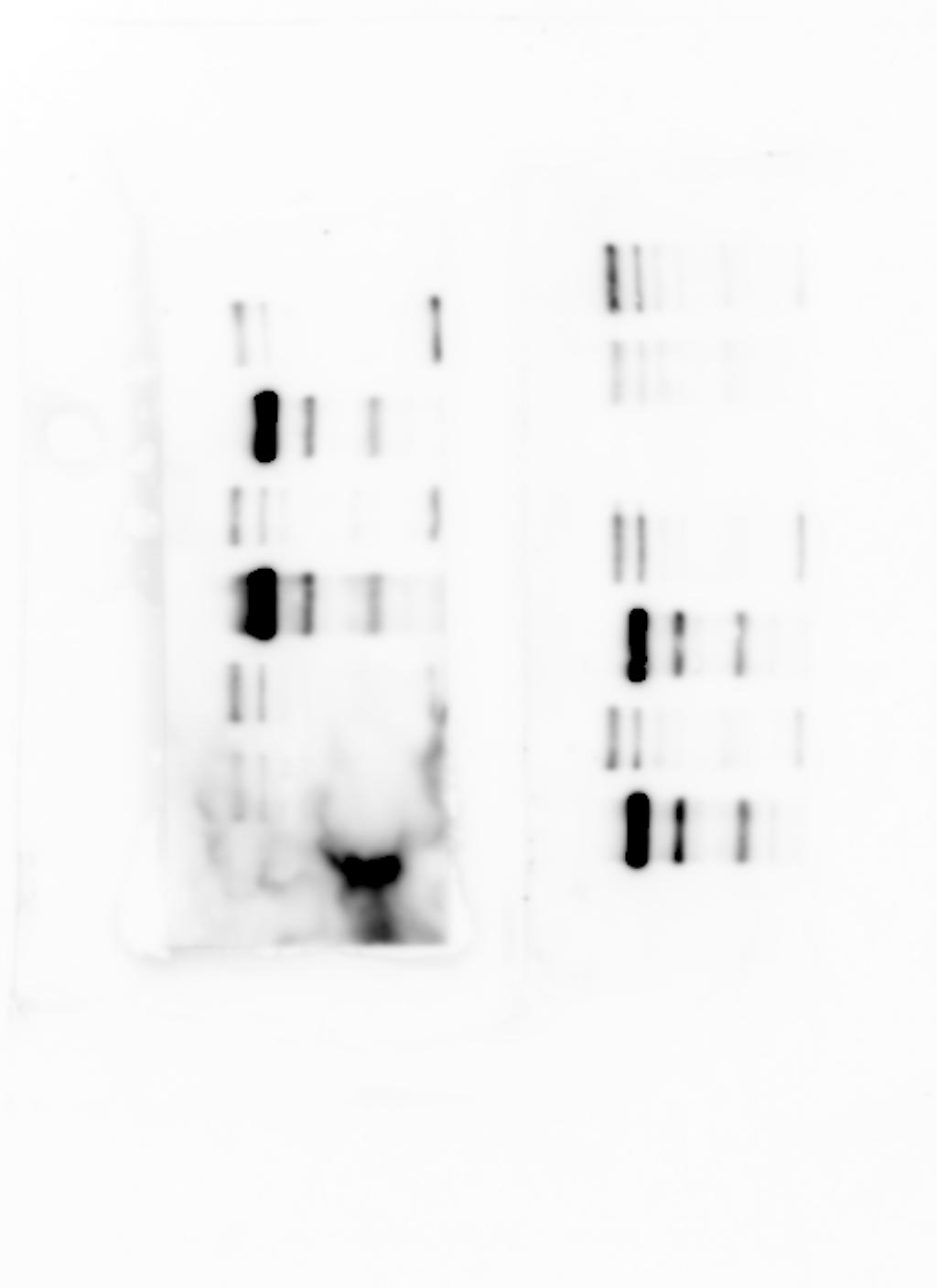

Supplement: Supplementary file 6 — Source data Fig. 3 [file 44318_2025_600_MOESM6_ESM.zip › Figure 3/3E/3E replicates/inh3 flag inc.extrem 2025.07.10_15.22.51-09_Ch.jpg]

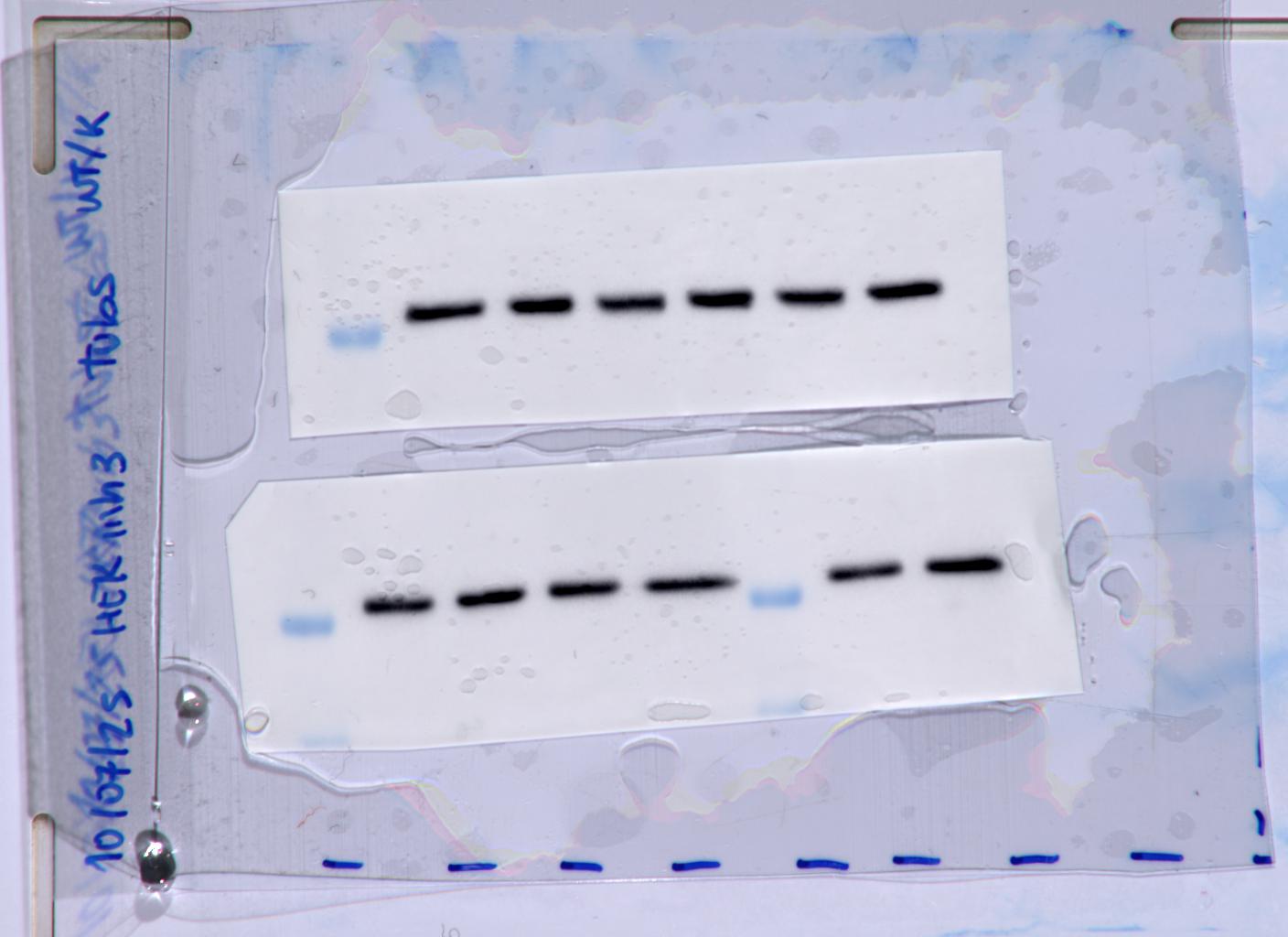

Supplement: Supplementary file 6 — Source data Fig. 3 [file 44318_2025_600_MOESM6_ESM.zip › Figure 3/3E/3E replicates/inh3 tubs 3s 2025.07.10_15.03.18_Ch+Marker.jpg]

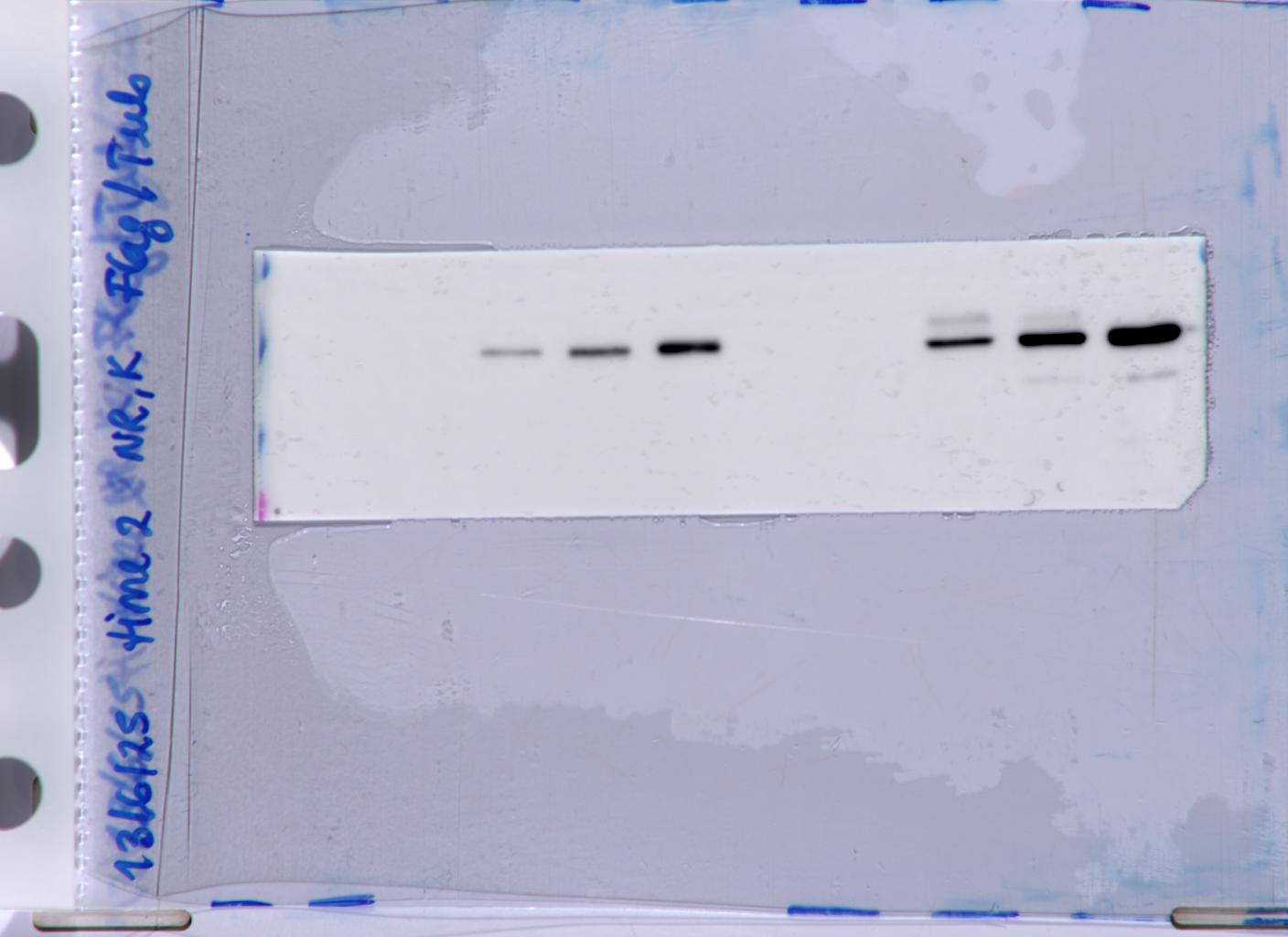

Supplement: Supplementary file 6 — Source data Fig. 3 [file 44318_2025_600_MOESM6_ESM.zip › Figure 3/3D/3D replicates/time 2nr,k flag 30s 2025.06.13_12.50.29_Ch+Marker.jpg]

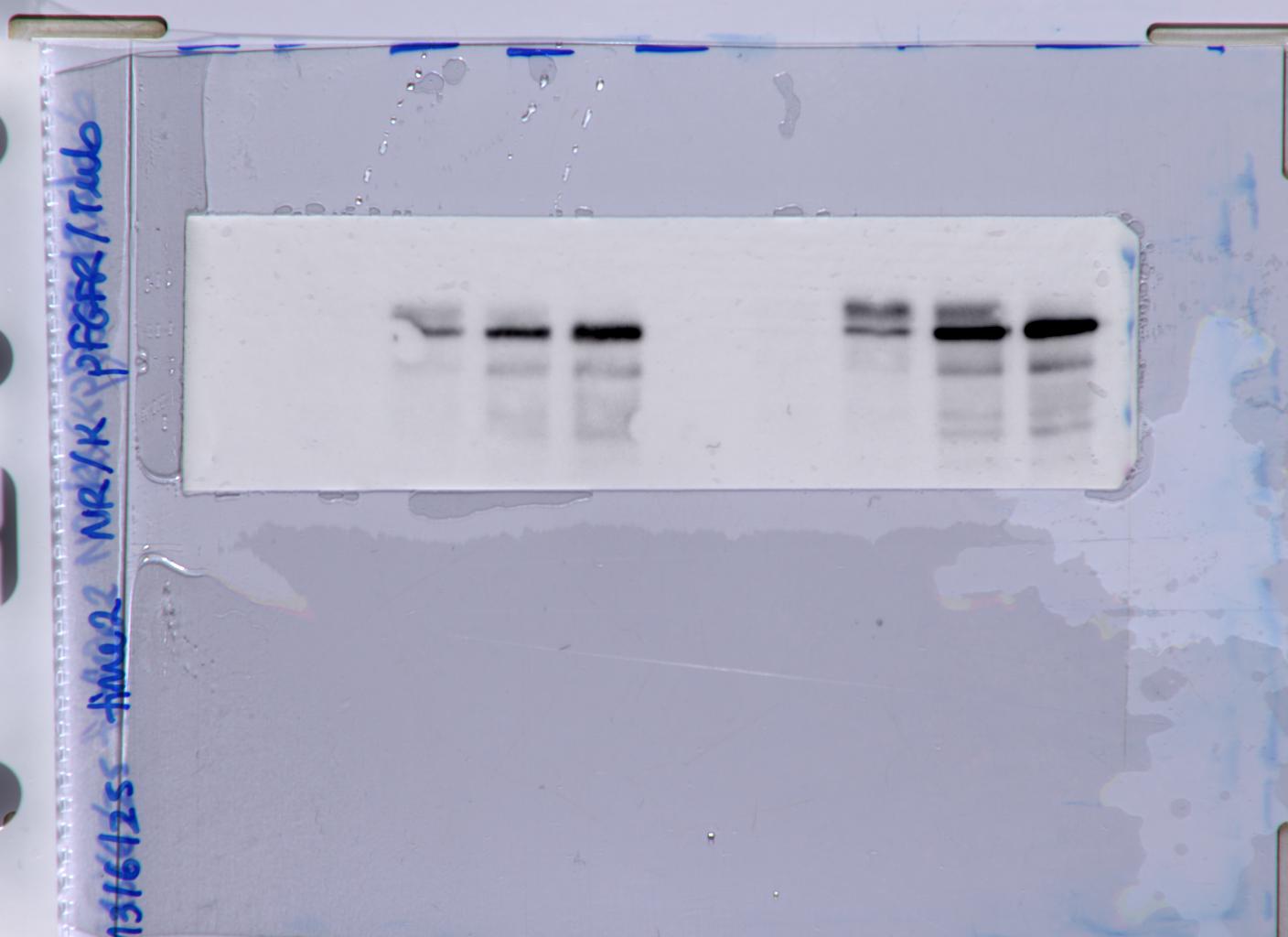

Supplement: Supplementary file 6 — Source data Fig. 3 [file 44318_2025_600_MOESM6_ESM.zip › Figure 3/3D/3D replicates/time2nrk pfgfr 0.1s 2025.06.13_12.08.23_Ch+Marker.jpg]

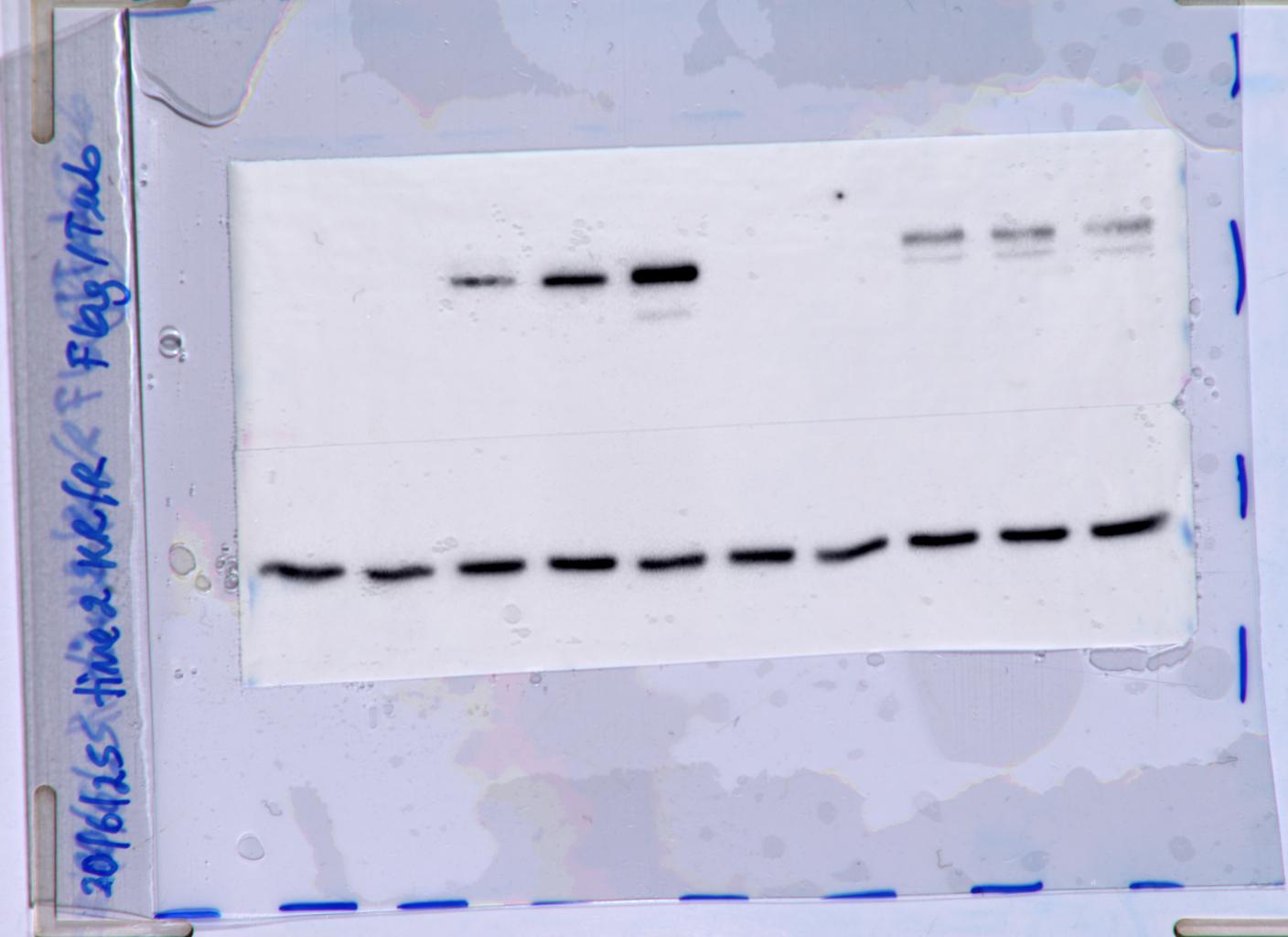

Supplement: Supplementary file 6 — Source data Fig. 3 [file 44318_2025_600_MOESM6_ESM.zip › Figure 3/3D/3D replicates/time2kr,r flagtub 1s 2025.06.20_12.17.24_Ch+Marker.jpg]

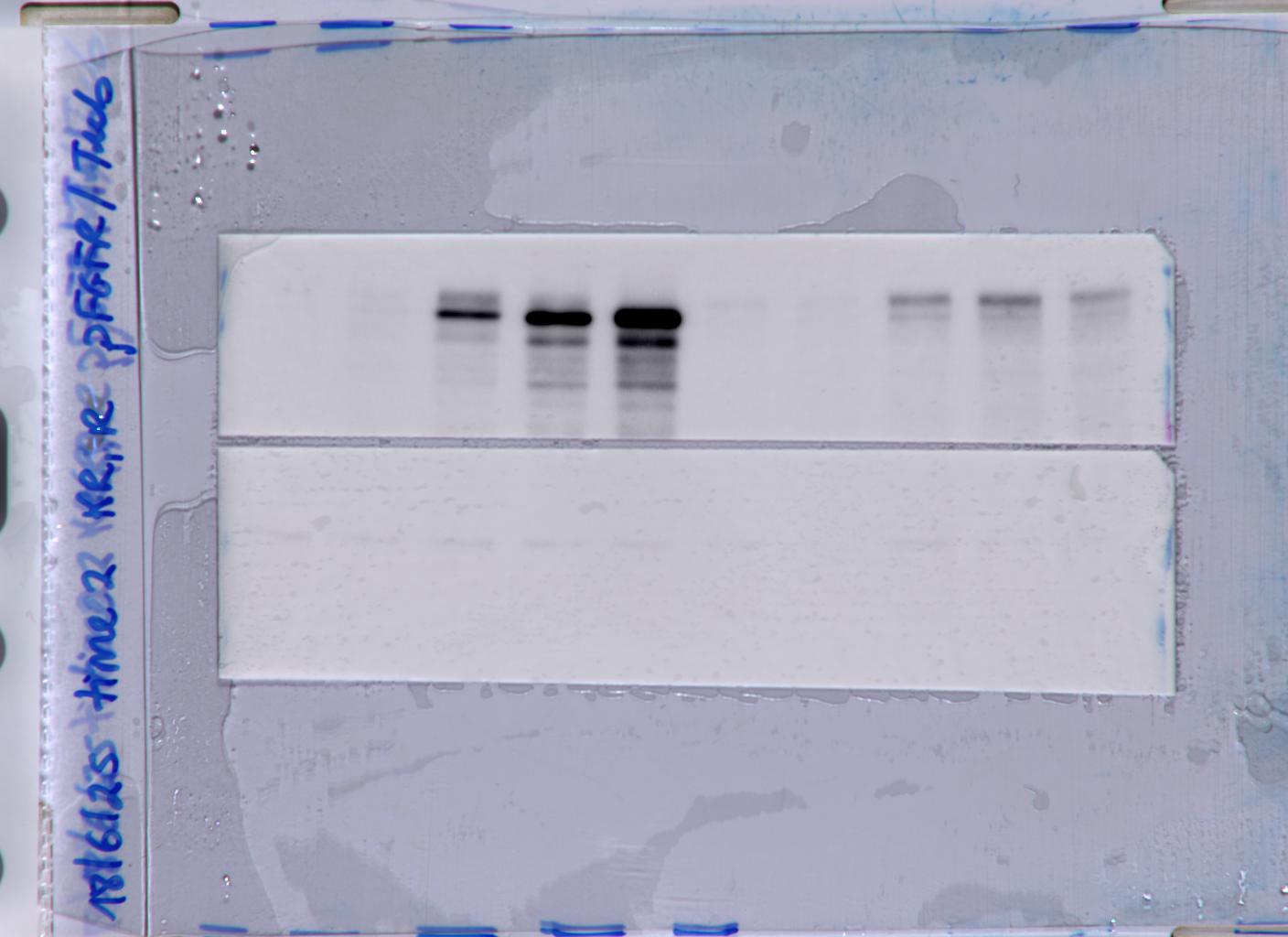

Supplement: Supplementary file 6 — Source data Fig. 3 [file 44318_2025_600_MOESM6_ESM.zip › Figure 3/3D/3D replicates/time2krr pfgrtub 0.5 2025.06.18_12.04.19_Ch+Marker.jpg]

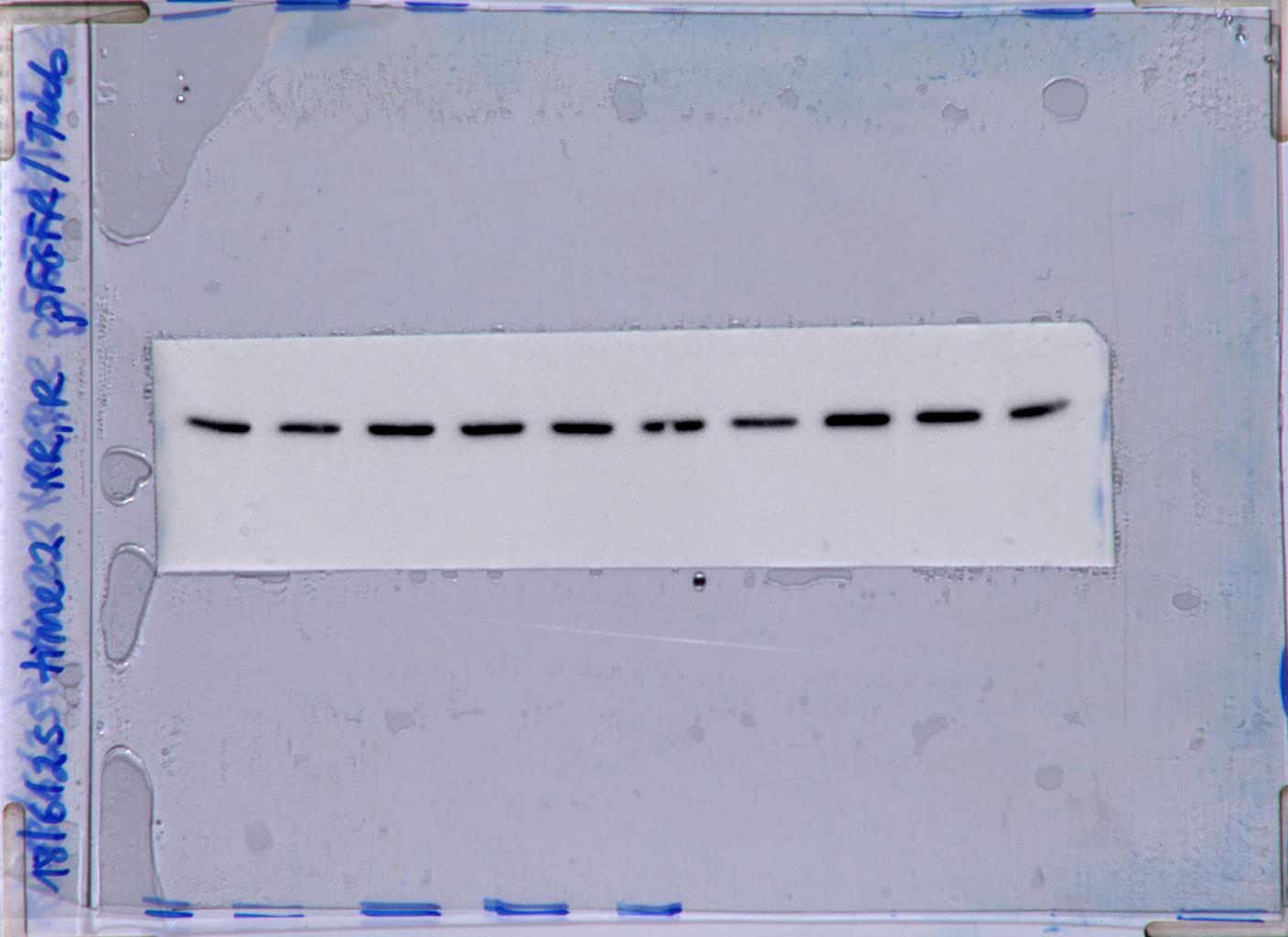

Supplement: Supplementary file 6 — Source data Fig. 3 [file 44318_2025_600_MOESM6_ESM.zip › Figure 3/3D/3D replicates/time2krr ptub 2.5 2025.06.18_12.09.13_Ch+Marker.jpg]

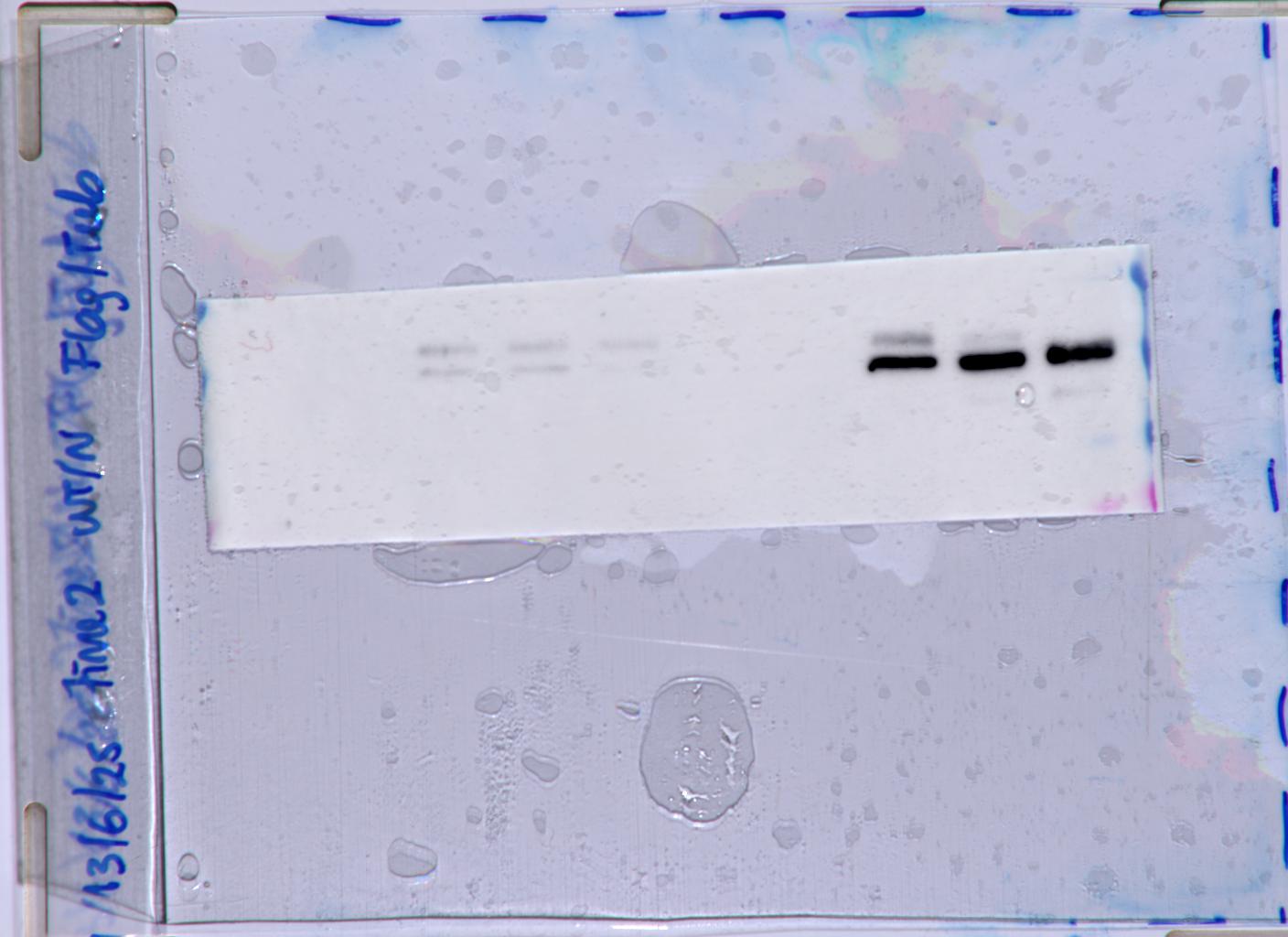

Supplement: Supplementary file 6 — Source data Fig. 3 [file 44318_2025_600_MOESM6_ESM.zip › Figure 3/3D/3D replicates/time 2wt,n flag 10s 2025.06.13_13.14.50_Ch+Marker.jpg]

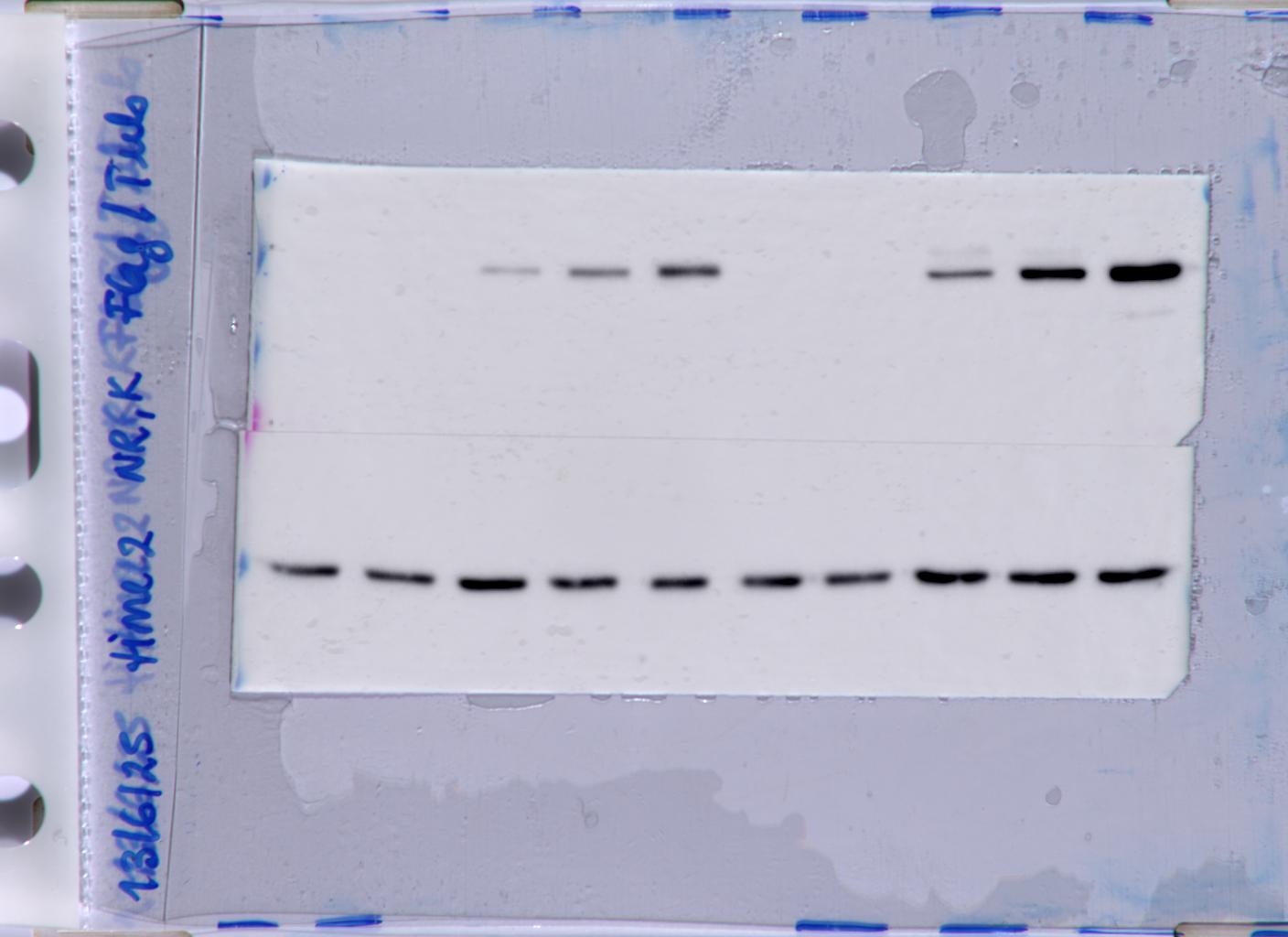

Supplement: Supplementary file 6 — Source data Fig. 3 [file 44318_2025_600_MOESM6_ESM.zip › Figure 3/3D/3D replicates/time2nrk flagtub 5s 2025.06.13_12.30.56_Ch+Marker.jpg]

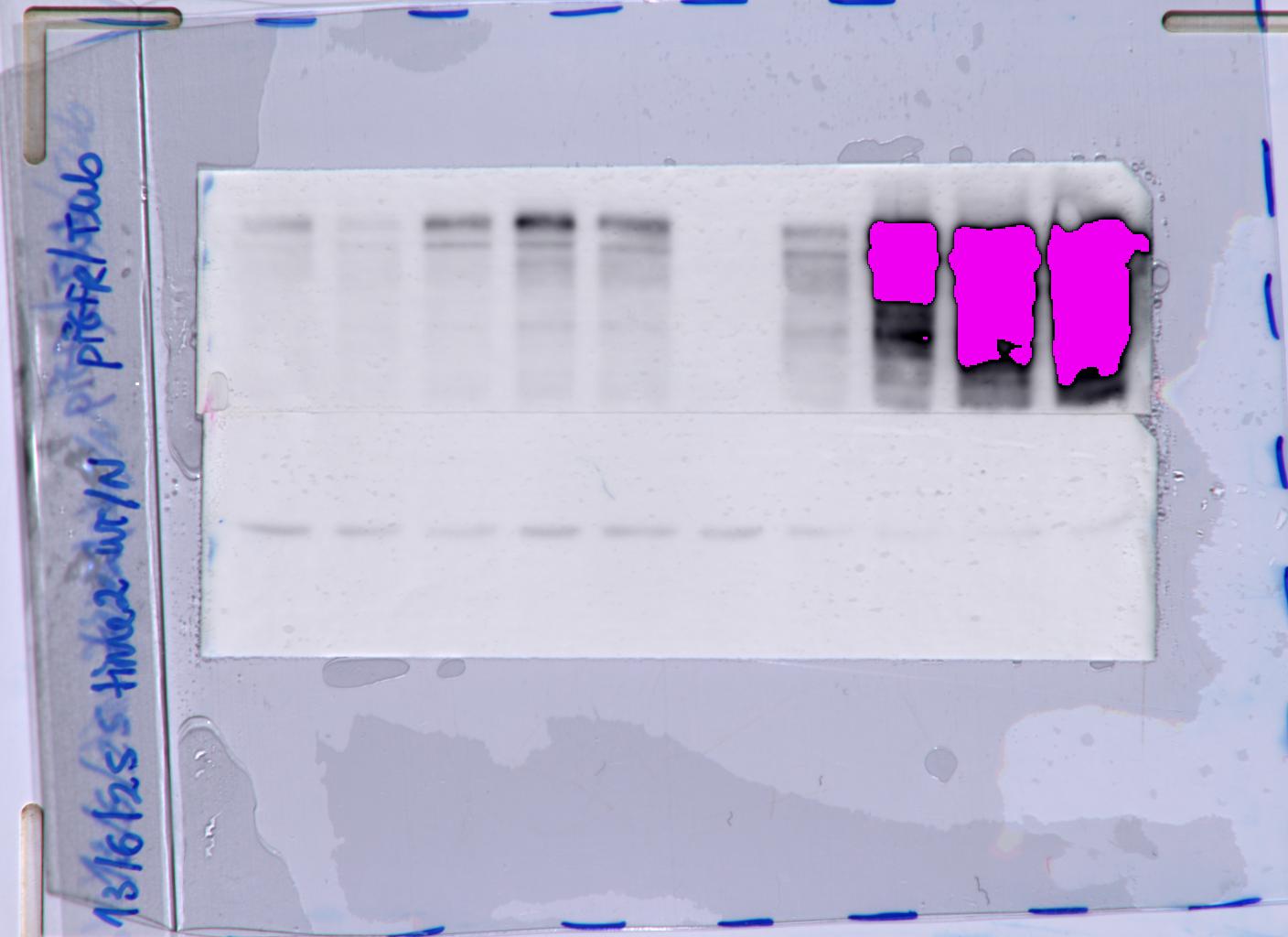

Supplement: Supplementary file 6 — Source data Fig. 3 [file 44318_2025_600_MOESM6_ESM.zip › Figure 3/3D/3D replicates/time2wtn pfgfrtub 10 2025.06.13_11.58.42_Ch+Marker.jpg]

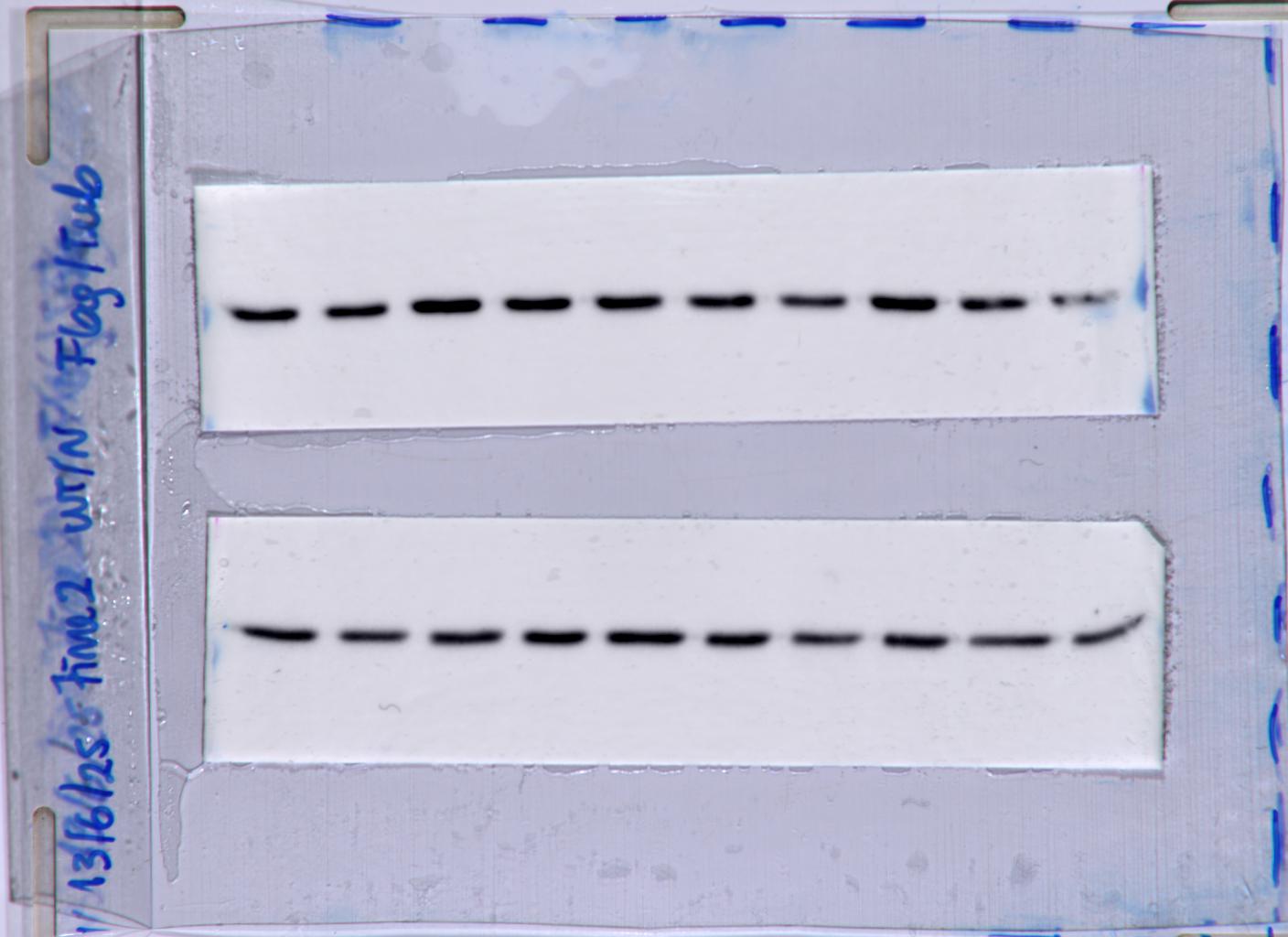

Supplement: Supplementary file 6 — Source data Fig. 3 [file 44318_2025_600_MOESM6_ESM.zip › Figure 3/3D/3D replicates/time 2 tubs 5s 2025.06.13_12.40.29_Ch+Marker.jpg]

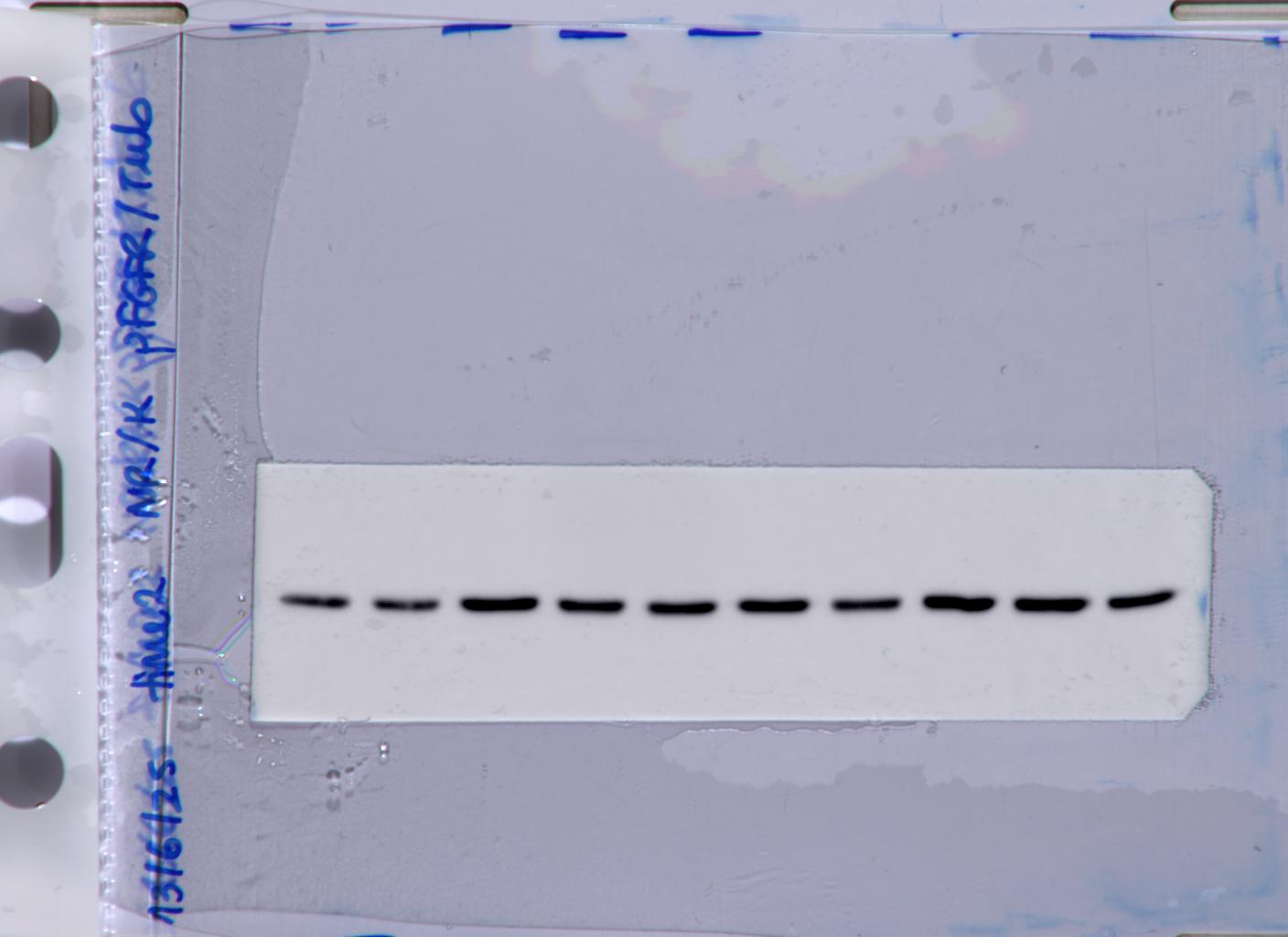

Supplement: Supplementary file 6 — Source data Fig. 3 [file 44318_2025_600_MOESM6_ESM.zip › Figure 3/3D/3D replicates/time2nrk ptub 5s 2025.06.13_12.15.06_Ch+Marker.jpg]

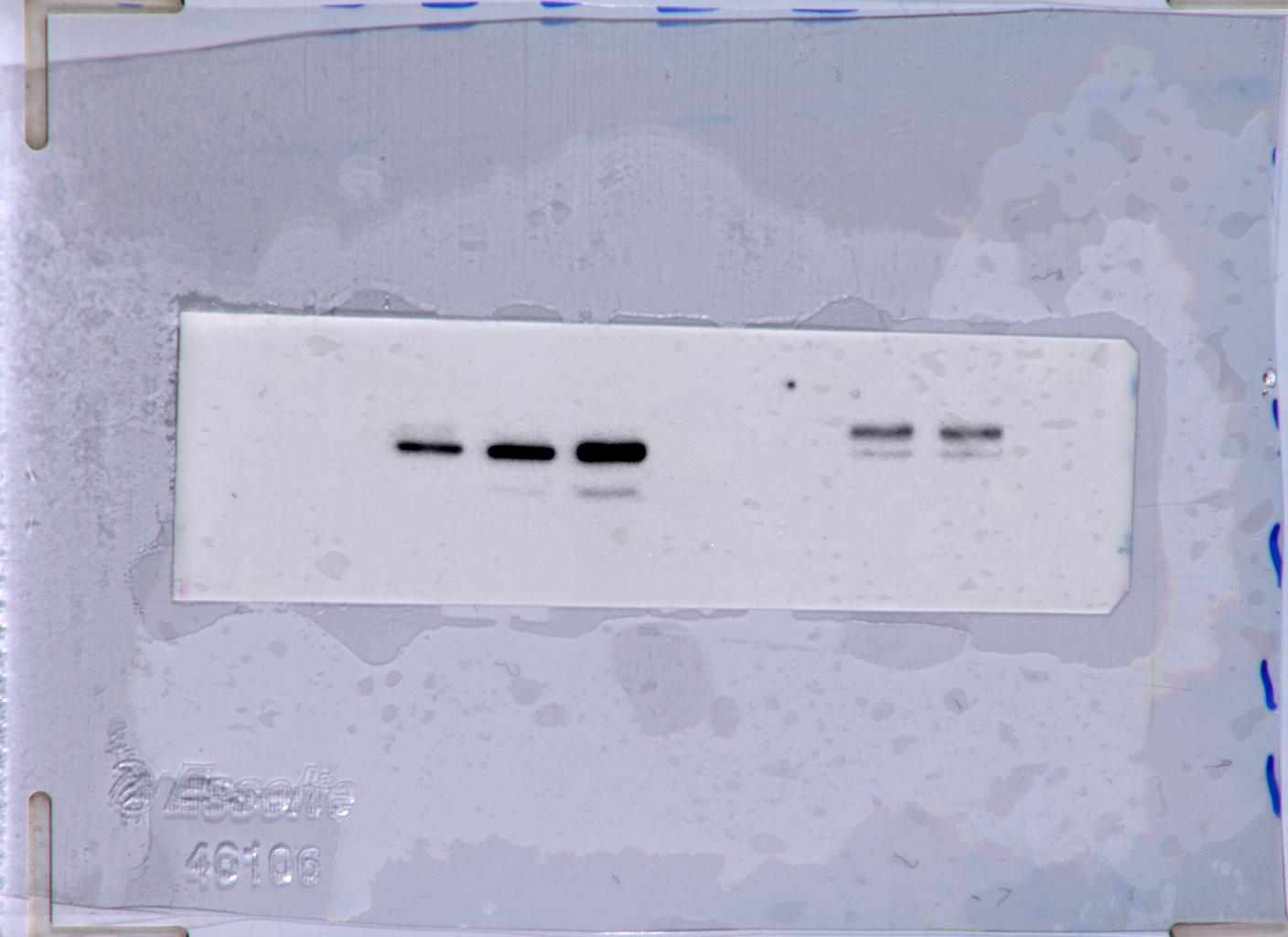

Supplement: Supplementary file 6 — Source data Fig. 3 [file 44318_2025_600_MOESM6_ESM.zip › Figure 3/3D/3D replicates/time2kr,r flag 1s 2025.06.20_12.19.46_Ch+Marker.jpg]

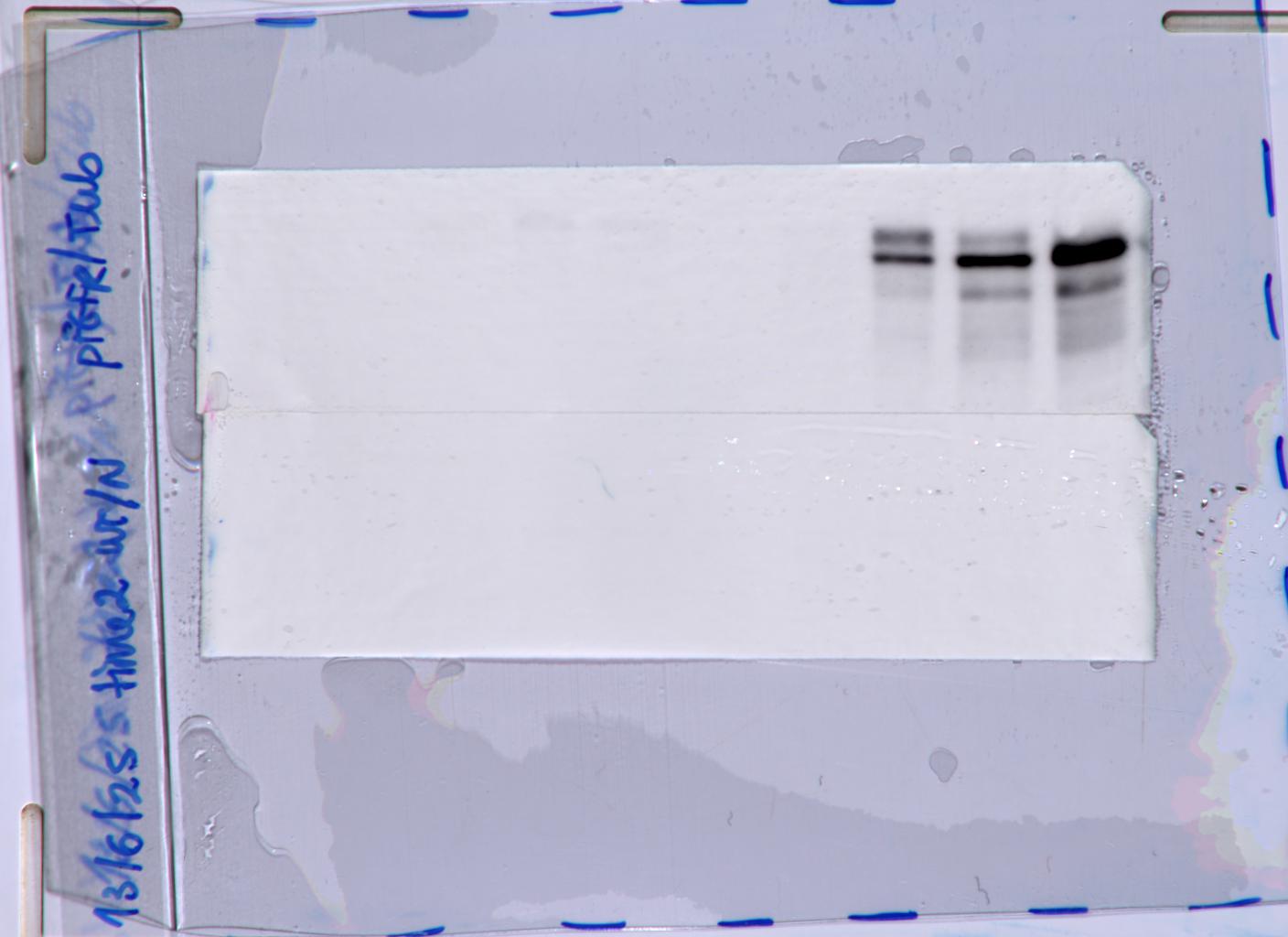

Supplement: Supplementary file 6 — Source data Fig. 3 [file 44318_2025_600_MOESM6_ESM.zip › Figure 3/3D/3D replicates/time2wtn pfgfrtub 0. 2025.06.13_11.56.11_Ch+Marker.jpg]

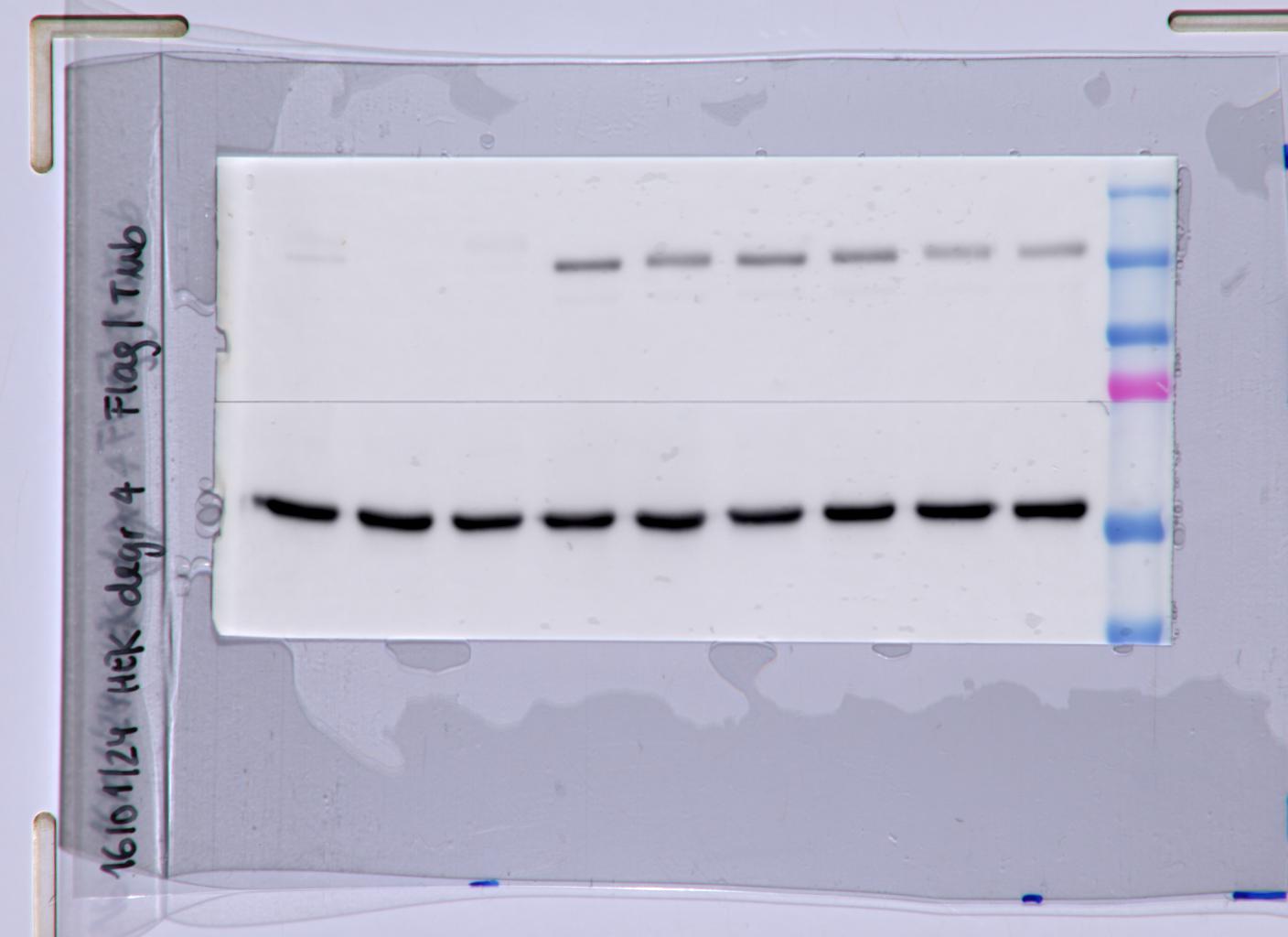

Supplement: Supplementary file 7 — Source data Fig. 4 [file 44318_2025_600_MOESM7_ESM.zip › Figure 4/4E/tubulin original.jpg]

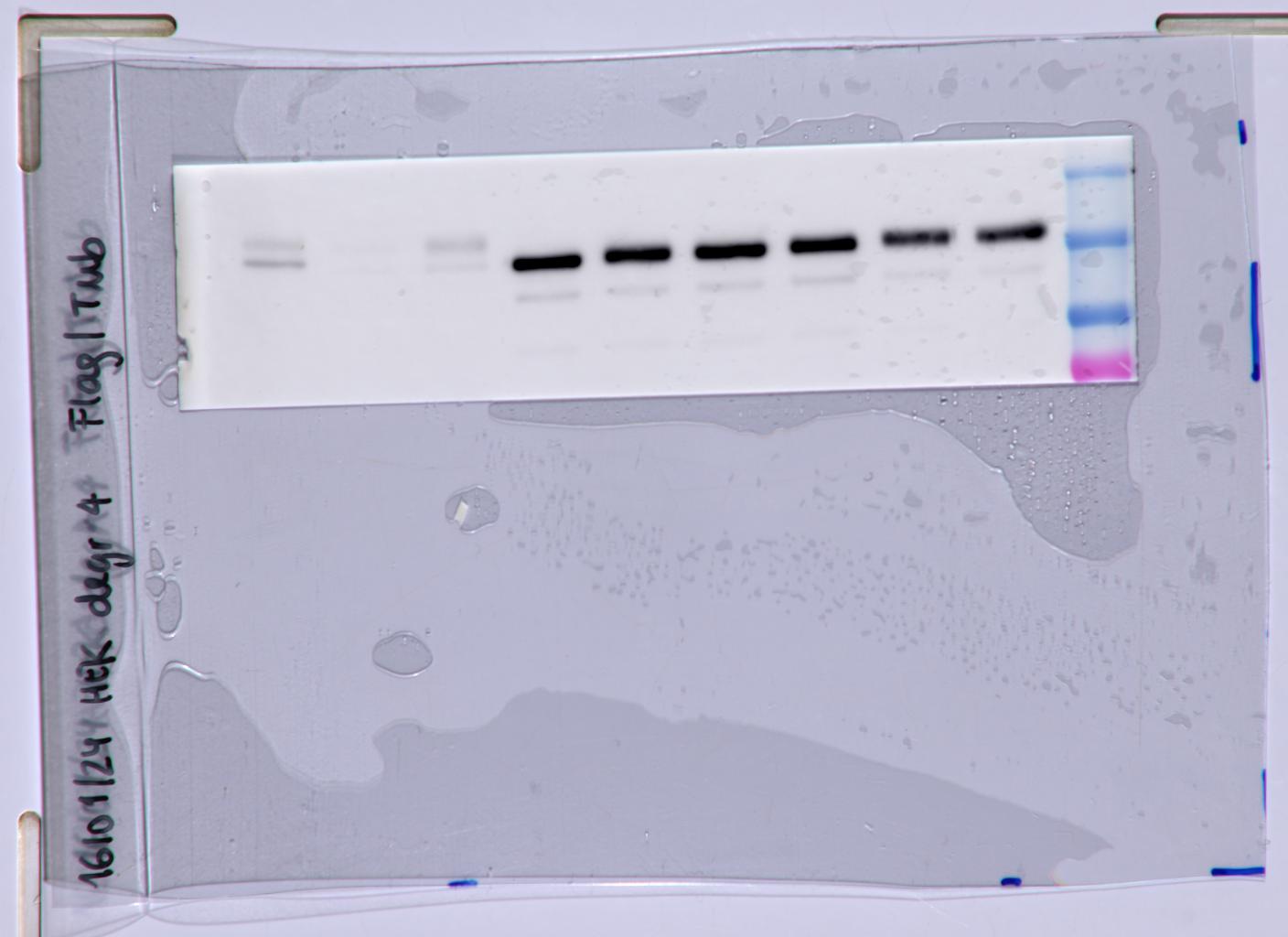

Supplement: Supplementary file 7 — Source data Fig. 4 [file 44318_2025_600_MOESM7_ESM.zip › Figure 4/4E/flag original.jpg]

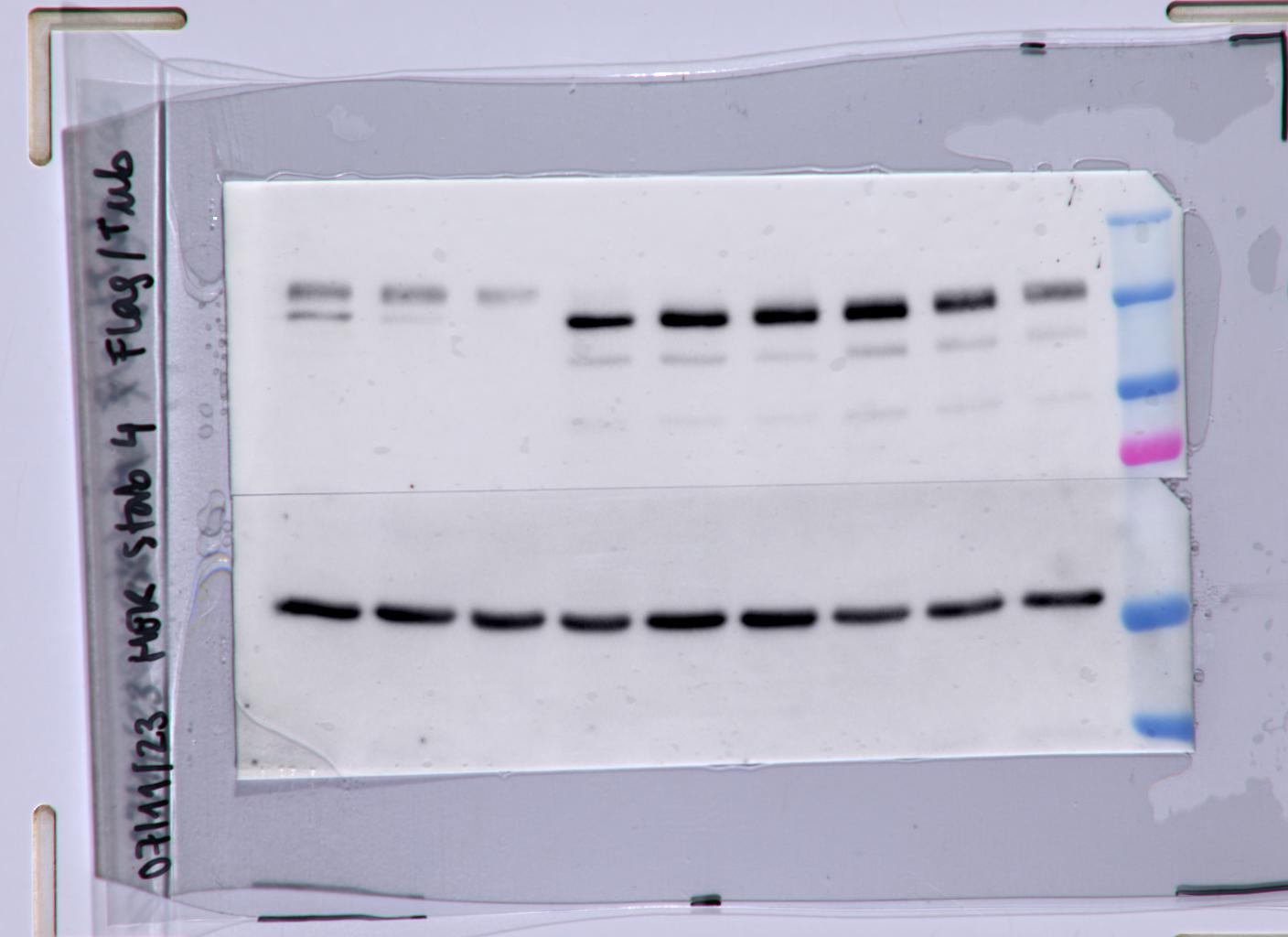

Supplement: Supplementary file 7 — Source data Fig. 4 [file 44318_2025_600_MOESM7_ESM.zip › Figure 4/4C/flag and tubulin original.jpg]
