# Supplementary material for: Concurrence of FGFR1 mutations modulates oncogenesis in glioneuronal tumors
Source: EMBO J. 2025 Oct 31;44(24):7513–40. doi: 10.1038/s44318-025-00600-3 (PMC12705663; doi:10.1038/s44318-025-00600-3)
Supplement: Supplementary file 6 — Source data Fig. 3 [file 44318_2025_600_MOESM6_ESM.zip › Figure 3/3E/WB 3D-E.pptx]

## Slide 1
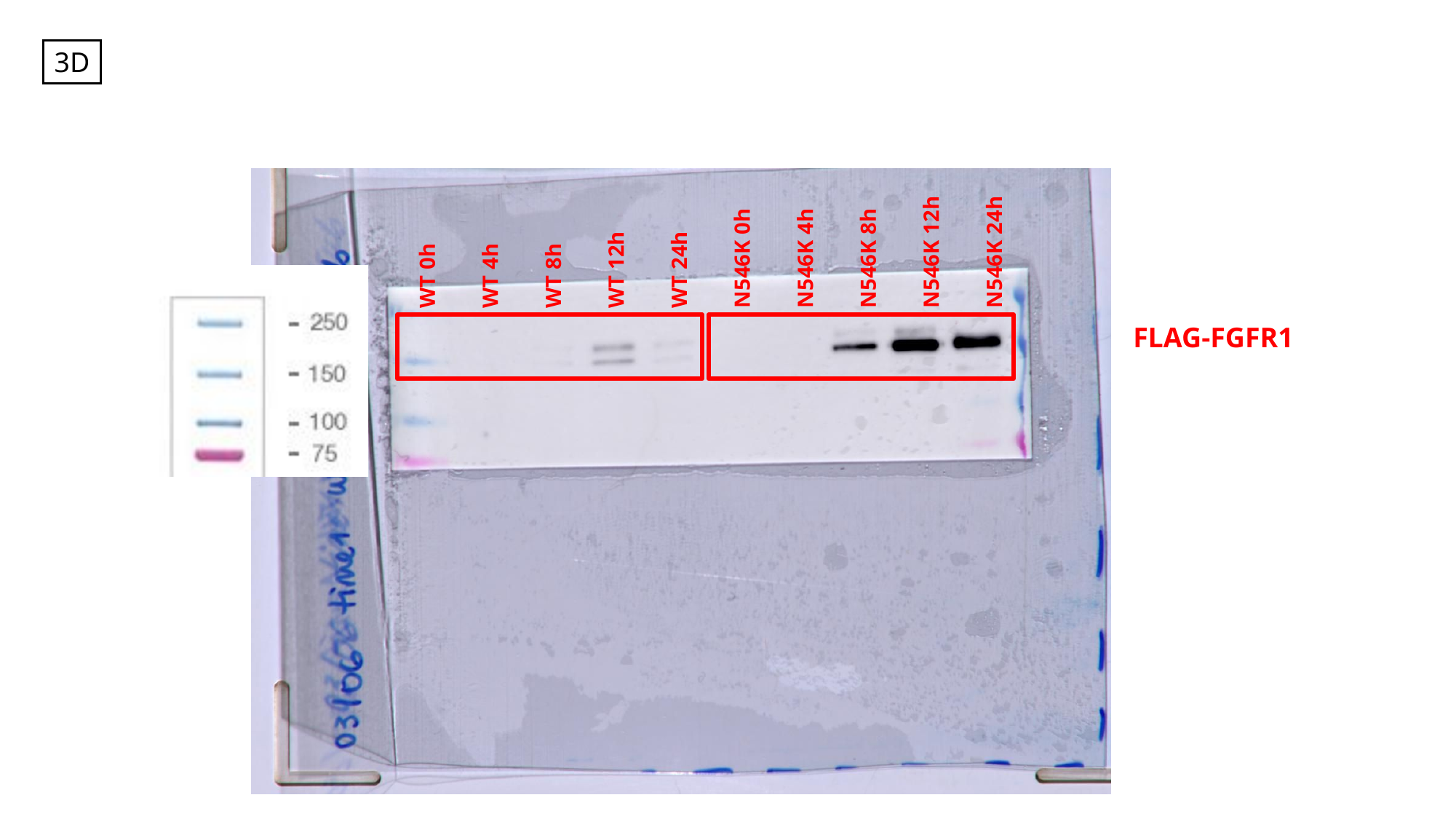

WT 0h
WT 4h
WT 8h
WT 12h
WT 24h
N546K 0h
N546K 4h
N546K 8h
N546K 12h
N546K 24h
3D
FLAG-FGFR1

## Slide 2
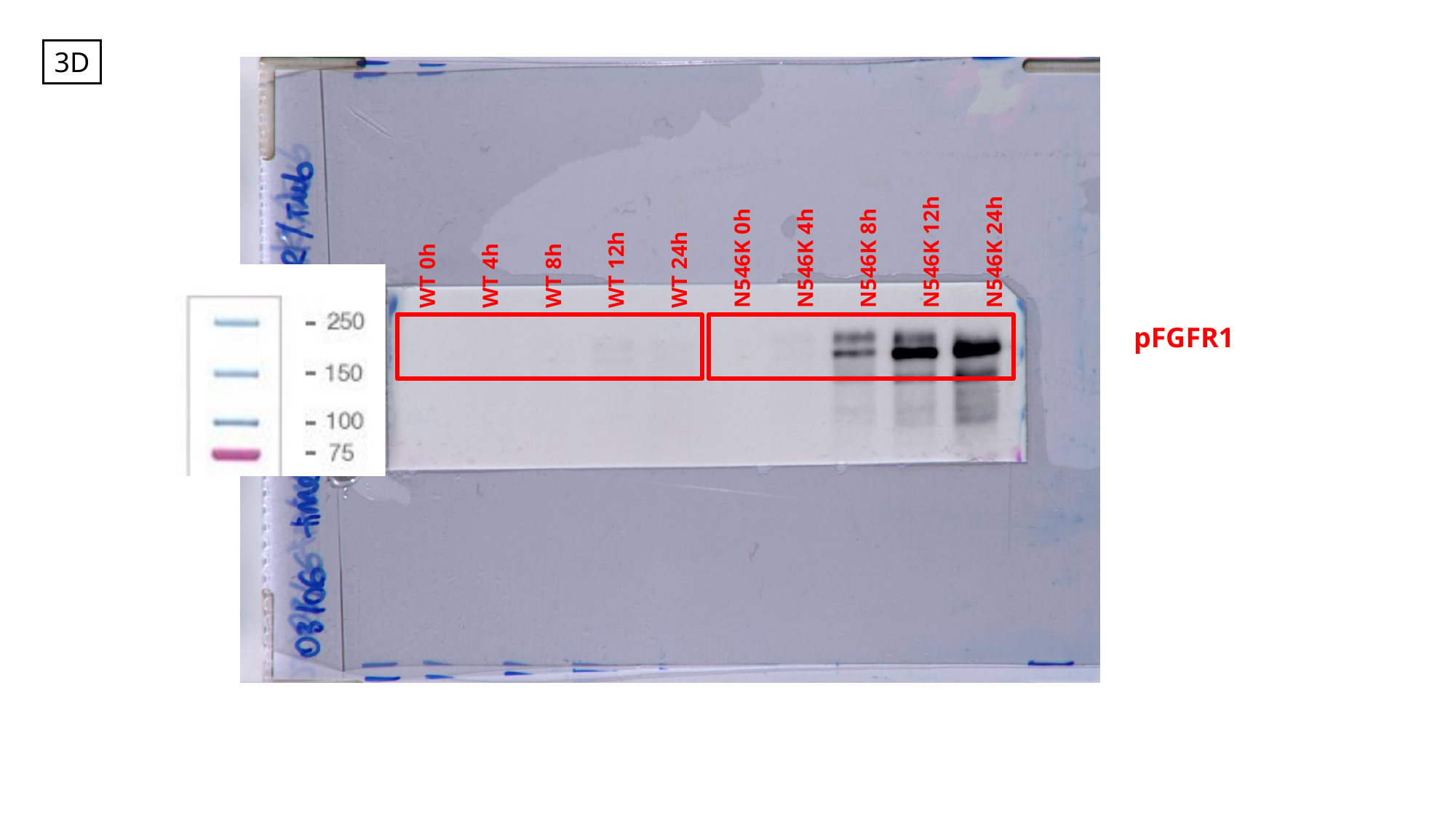

WT 0h
WT 4h
WT 8h
WT 12h
WT 24h
N546K 0h
N546K 4h
N546K 8h
N546K 12h
N546K 24h
3D
pFGFR1

## Slide 3
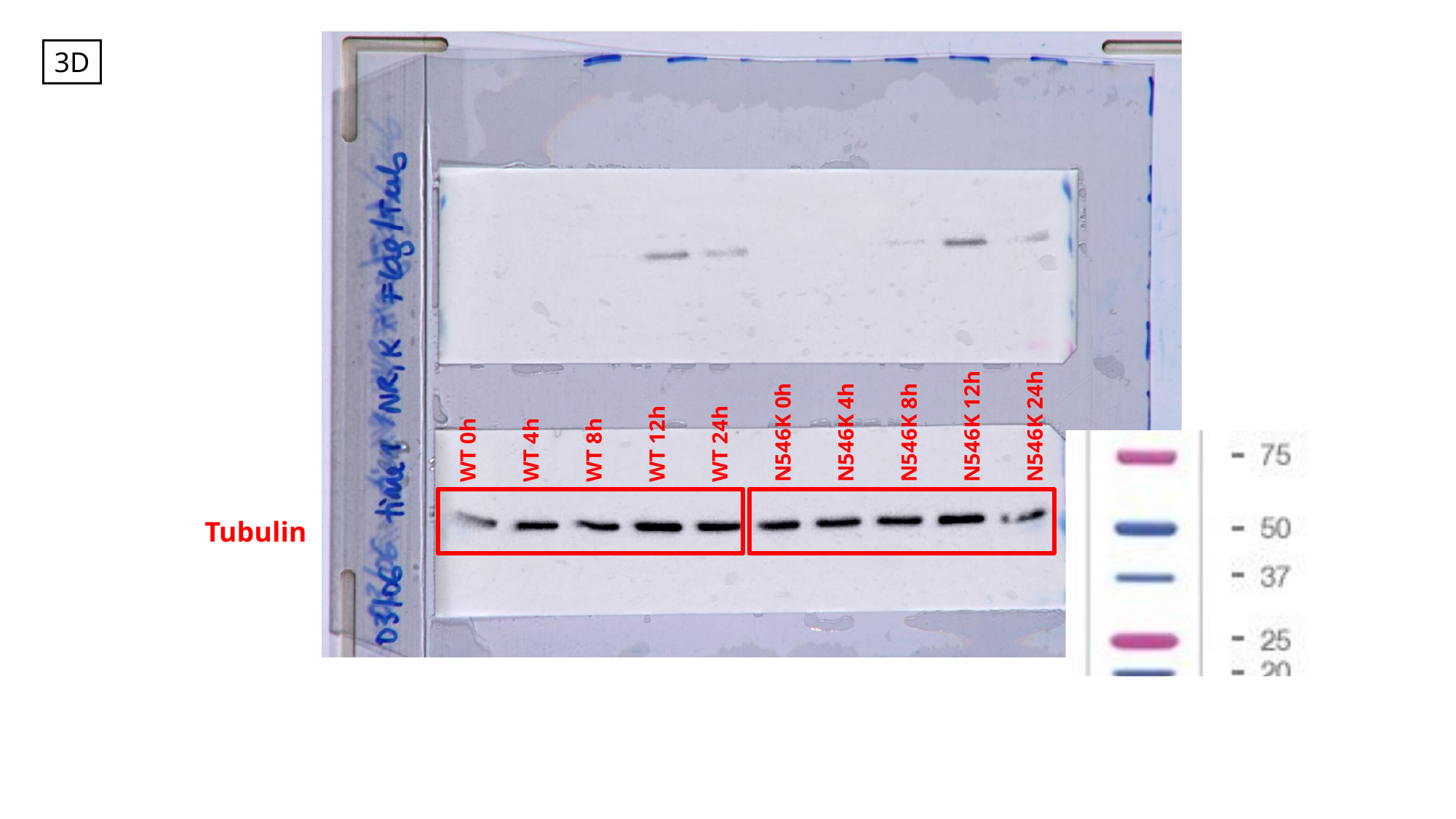

3D
WT 0h
WT 4h
WT 8h
WT 12h
WT 24h
N546K 0h
N546K 4h
N546K 8h
N546K 12h
N546K 24h
Tubulin

## Slide 4
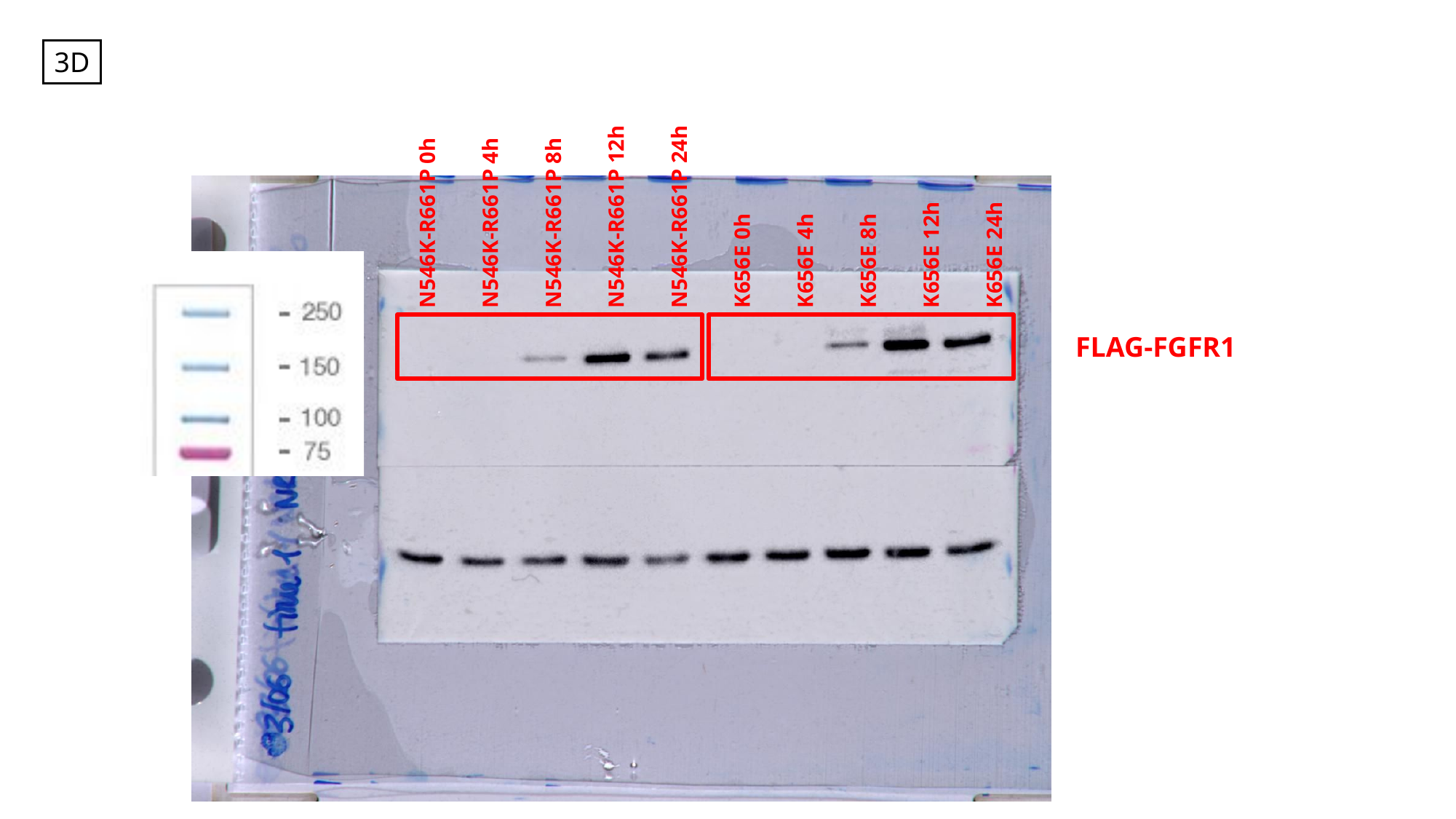

N546K-R661P 0h
N546K-R661P 4h
N546K-R661P 8h
N546K-R661P 12h
N546K-R661P 24h
K656E 0h
K656E 4h
K656E 8h
K656E 12h
K656E 24h
3D
FLAG-FGFR1

## Slide 5
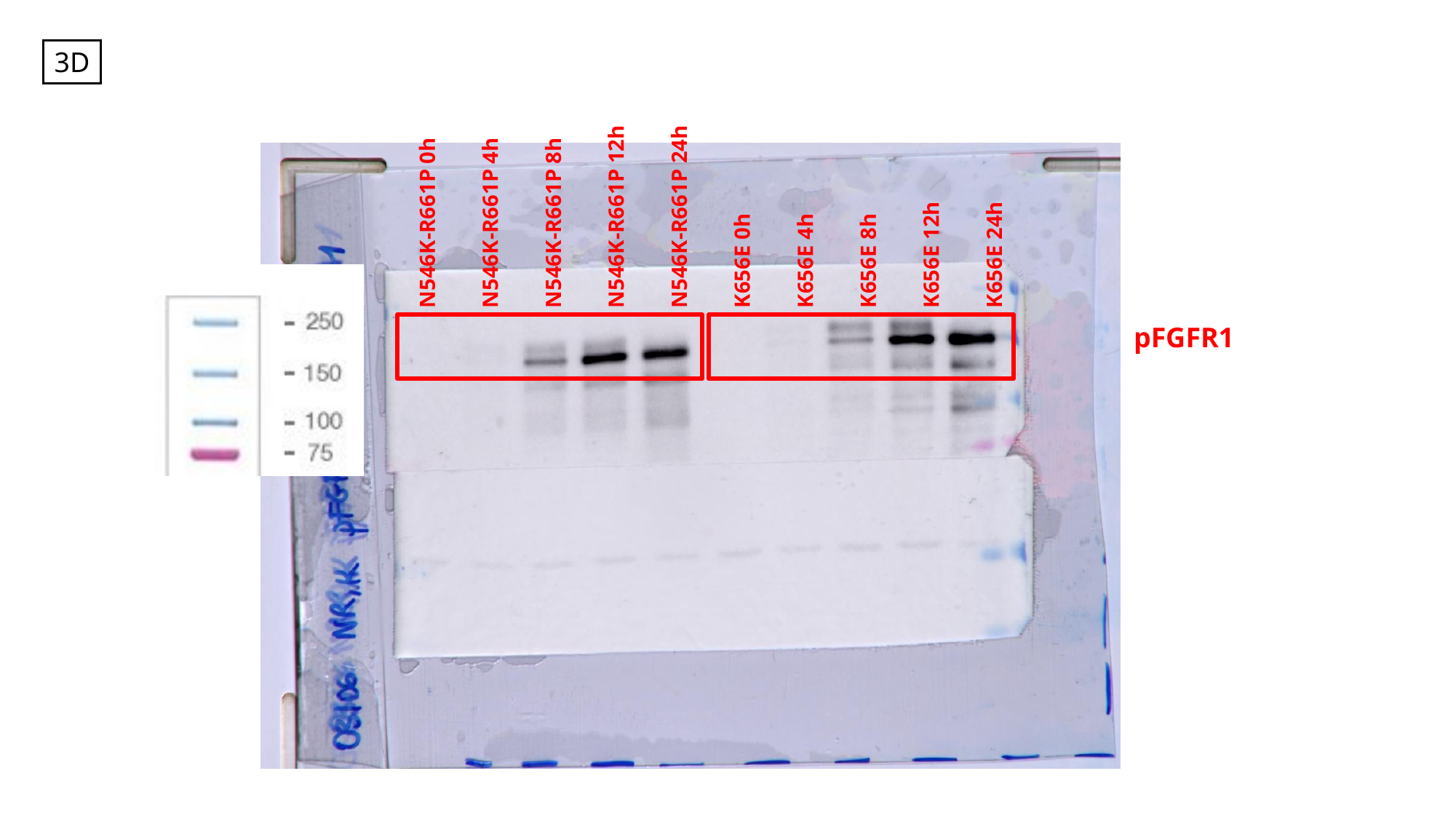

N546K-R661P 0h
N546K-R661P 4h
N546K-R661P 8h
N546K-R661P 12h
N546K-R661P 24h
K656E 0h
K656E 4h
K656E 8h
K656E 12h
K656E 24h
3D
pFGFR1

## Slide 6
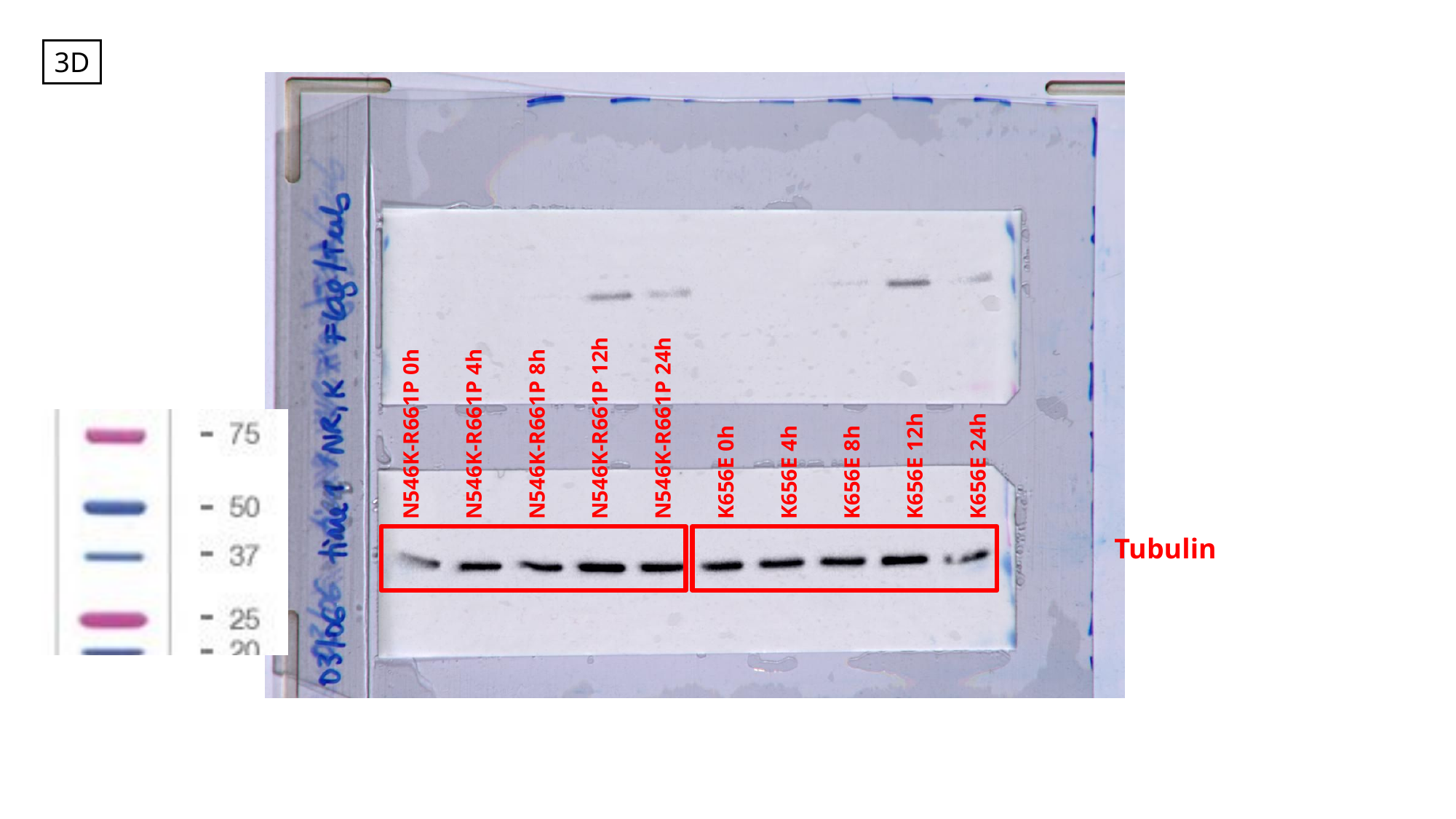

3D
N546K-R661P 0h
N546K-R661P 4h
N546K-R661P 8h
N546K-R661P 12h
N546K-R661P 24h
K656E 0h
K656E 4h
K656E 8h
K656E 12h
K656E 24h
Tubulin

## Slide 7
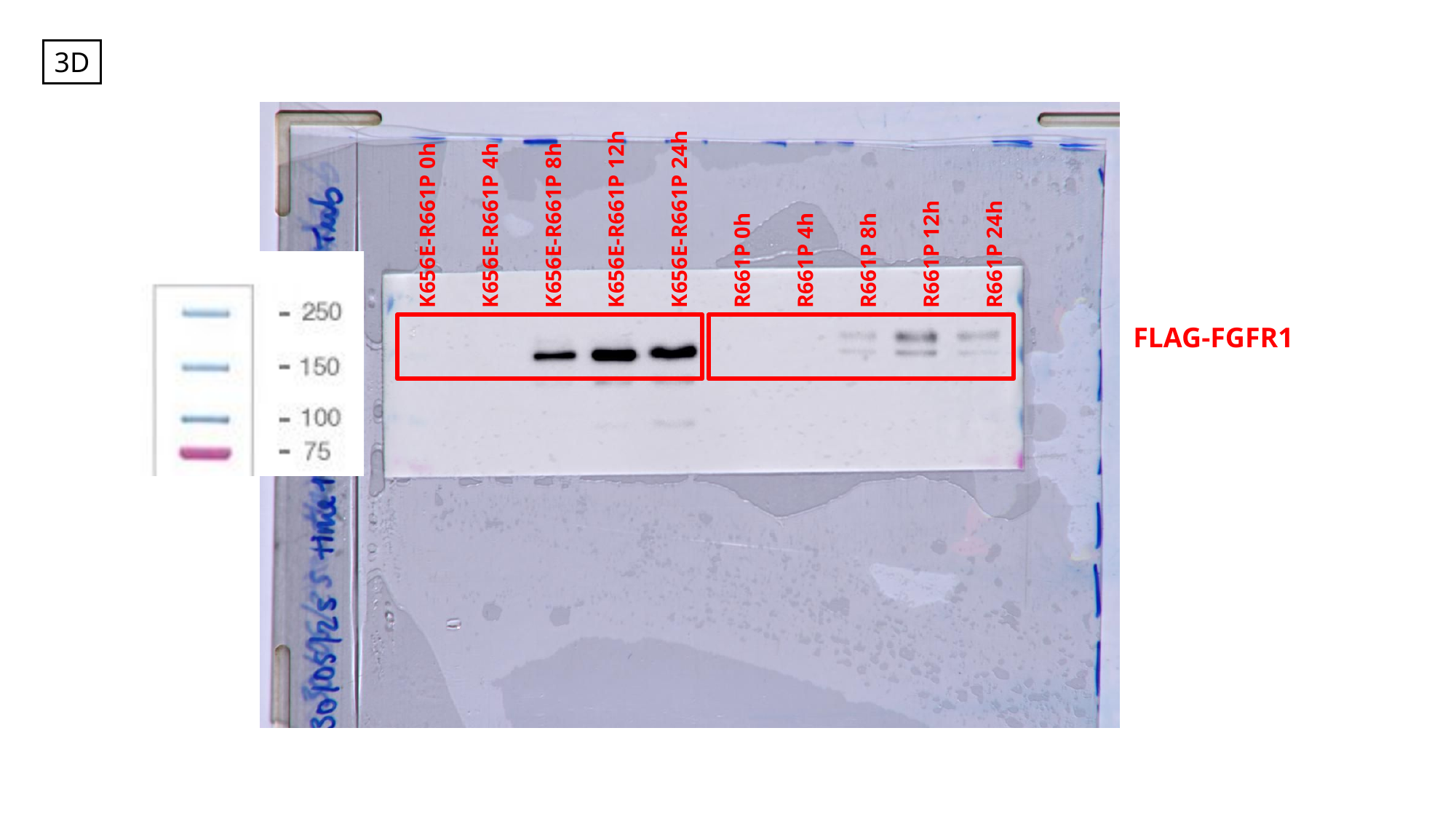

K656E-R661P 0h
K656E-R661P 4h
K656E-R661P 8h
K656E-R661P 12h
K656E-R661P 24h
R661P 0h
R661P 4h
R661P 8h
R661P 12h
R661P 24h
3D
FLAG-FGFR1

## Slide 8
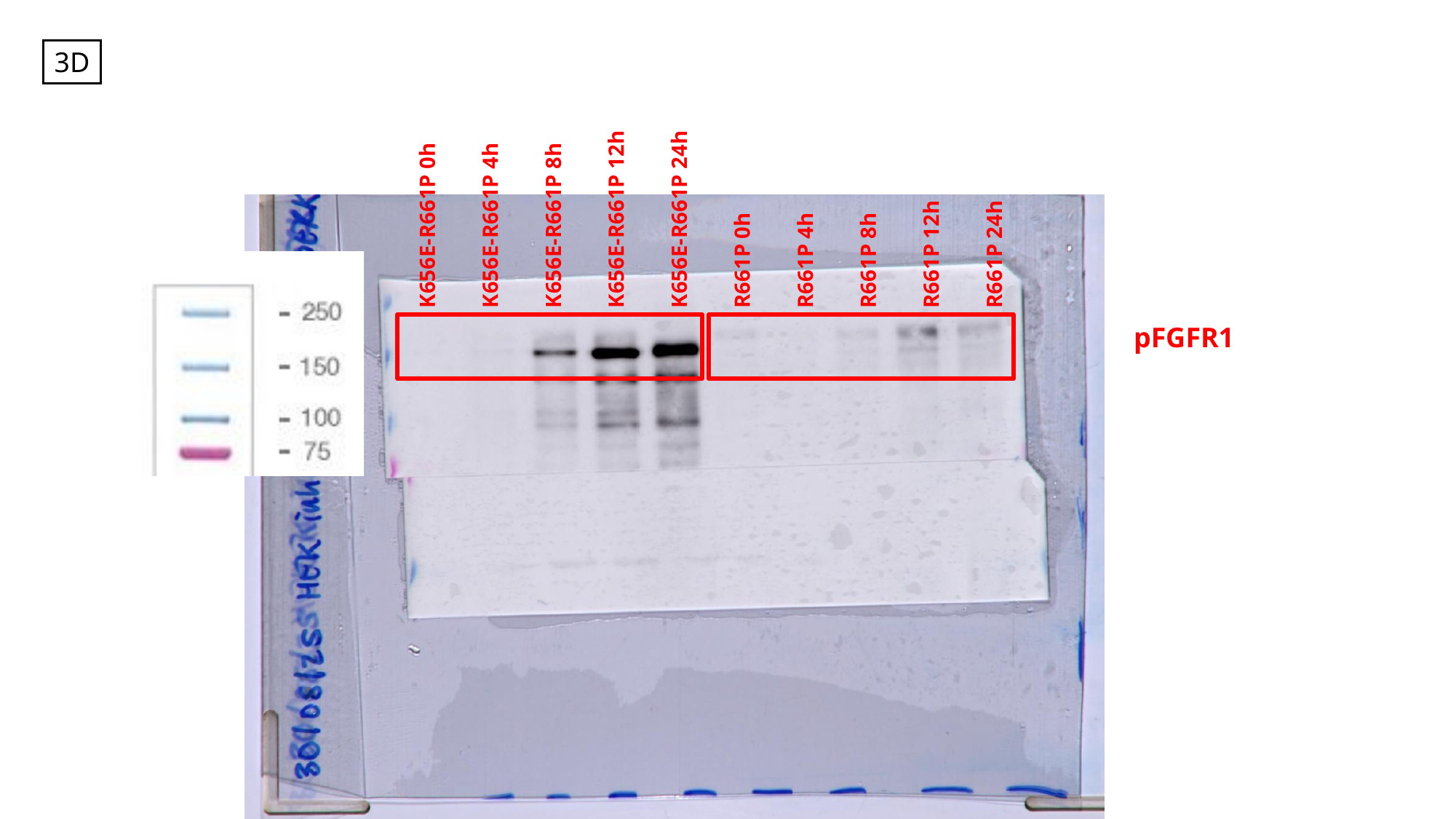

K656E-R661P 0h
K656E-R661P 4h
K656E-R661P 8h
K656E-R661P 12h
K656E-R661P 24h
R661P 0h
R661P 4h
R661P 8h
R661P 12h
R661P 24h
3D
pFGFR1

## Slide 9
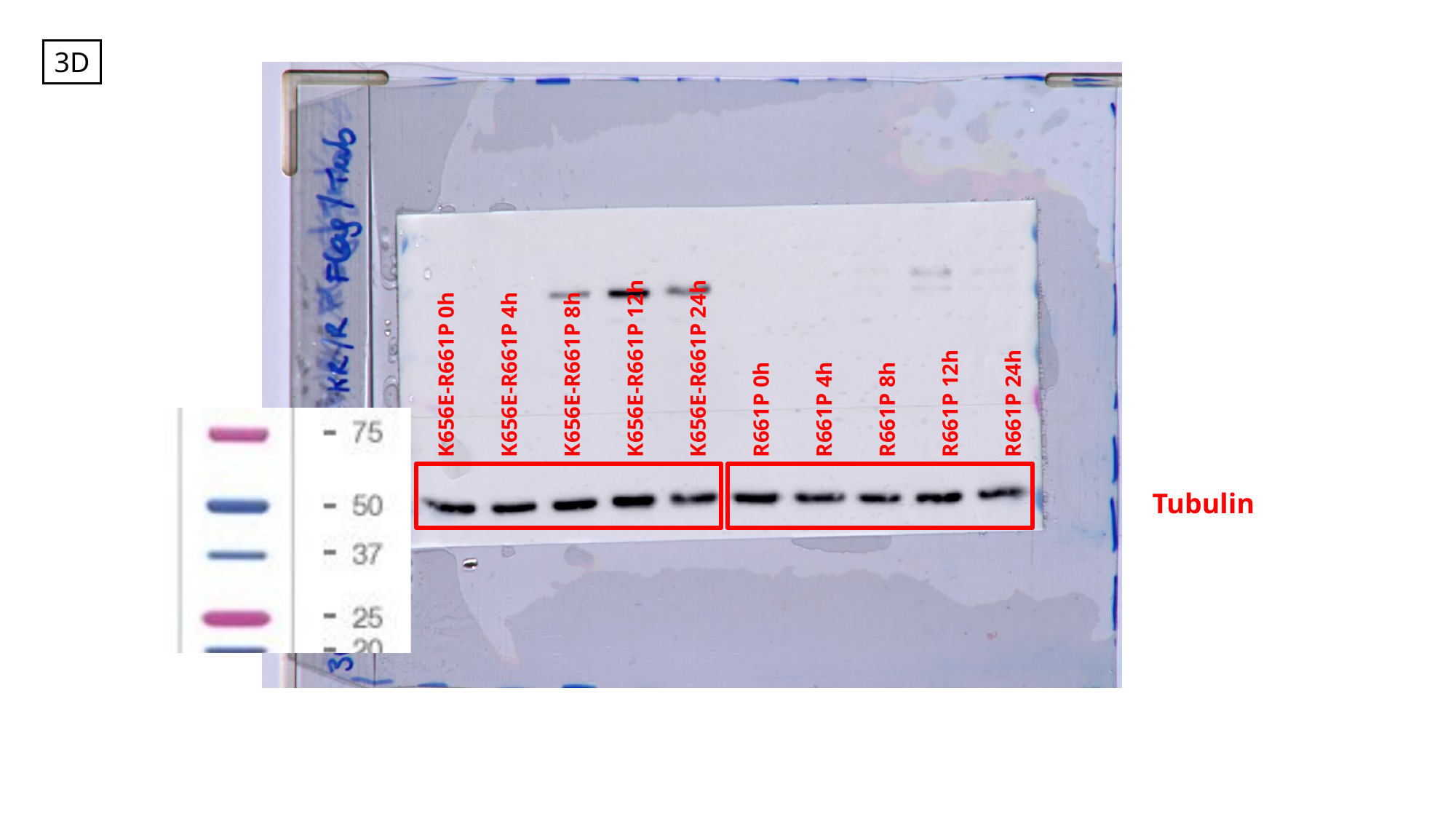

K656E-R661P 0h
K656E-R661P 4h
K656E-R661P 8h
K656E-R661P 12h
K656E-R661P 24h
R661P 0h
R661P 4h
R661P 8h
R661P 12h
R661P 24h
3D
Tubulin

## Slide 10
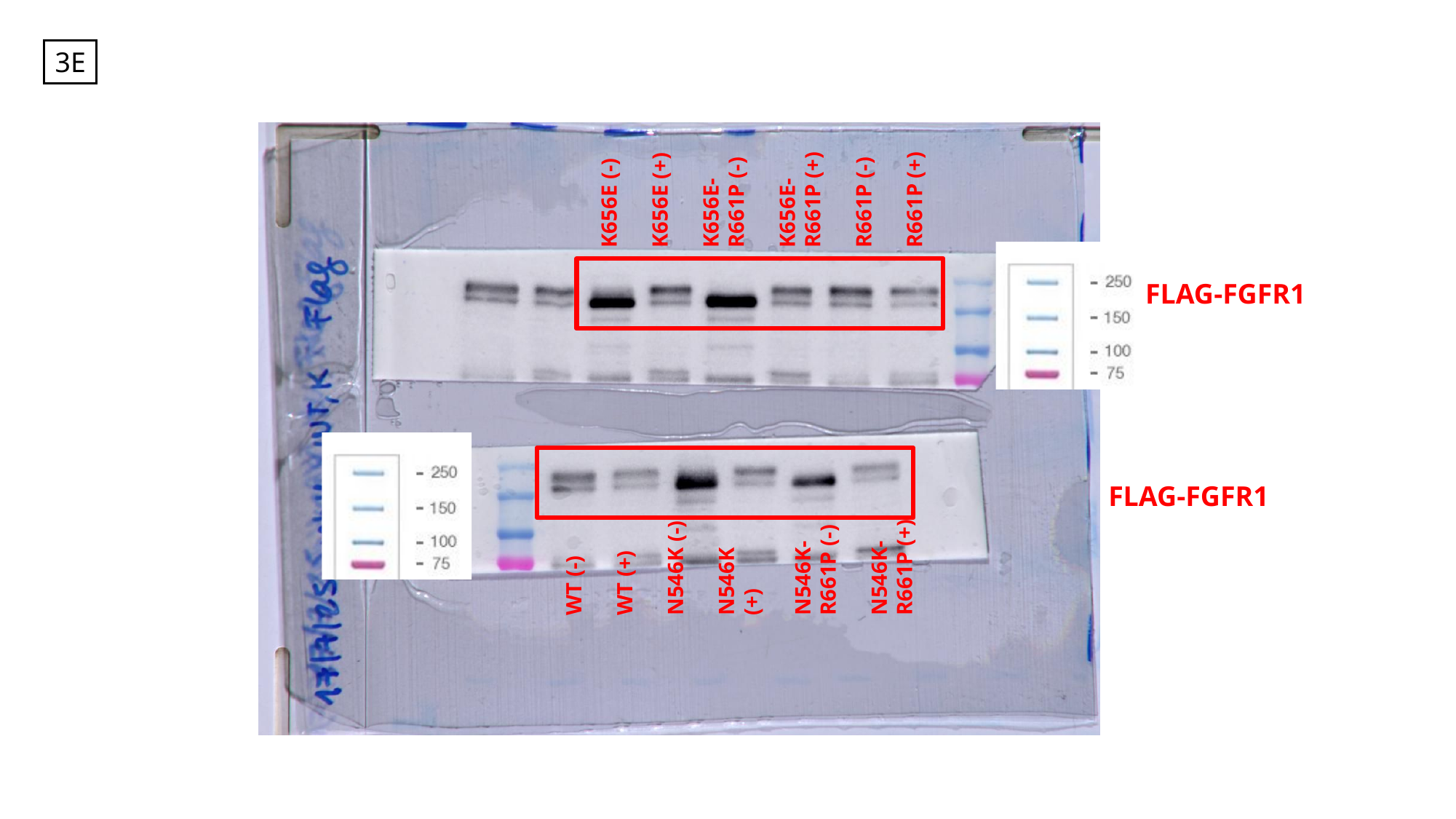

K656E (-)
K656E (+)
K656E-R661P (-)
K656E-R661P (+)
R661P (-)
R661P (+)
3E
FLAG-FGFR1
WT (-)
WT (+)
N546K (-)
N546K (+)
N546K-R661P (-)
N546K-R661P (+)
FLAG-FGFR1

## Slide 11
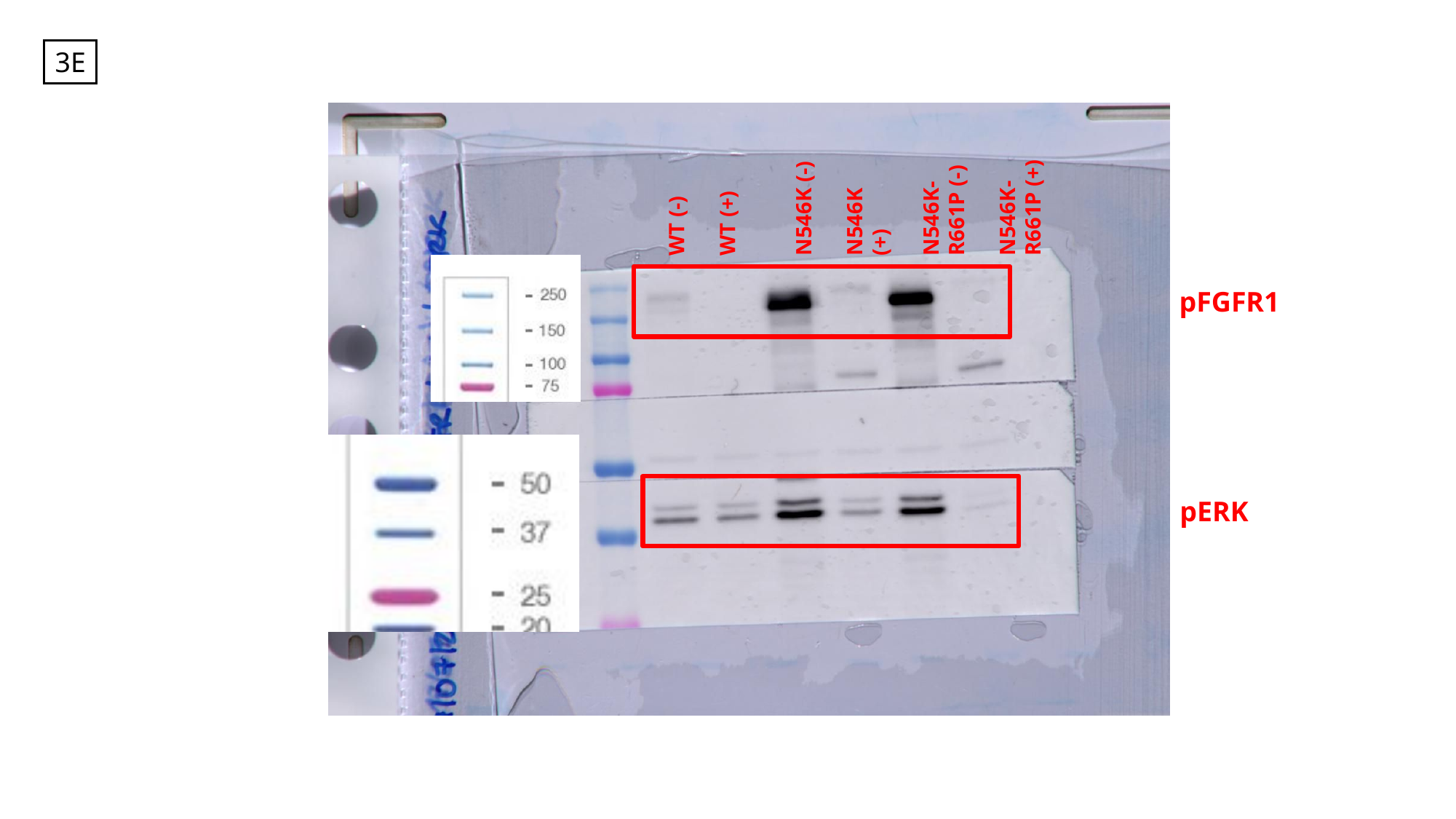

WT (-)
WT (+)
N546K (-)
N546K (+)
N546K-R661P (-)
N546K-R661P (+)
3E
pFGFR1
pERK

## Slide 12
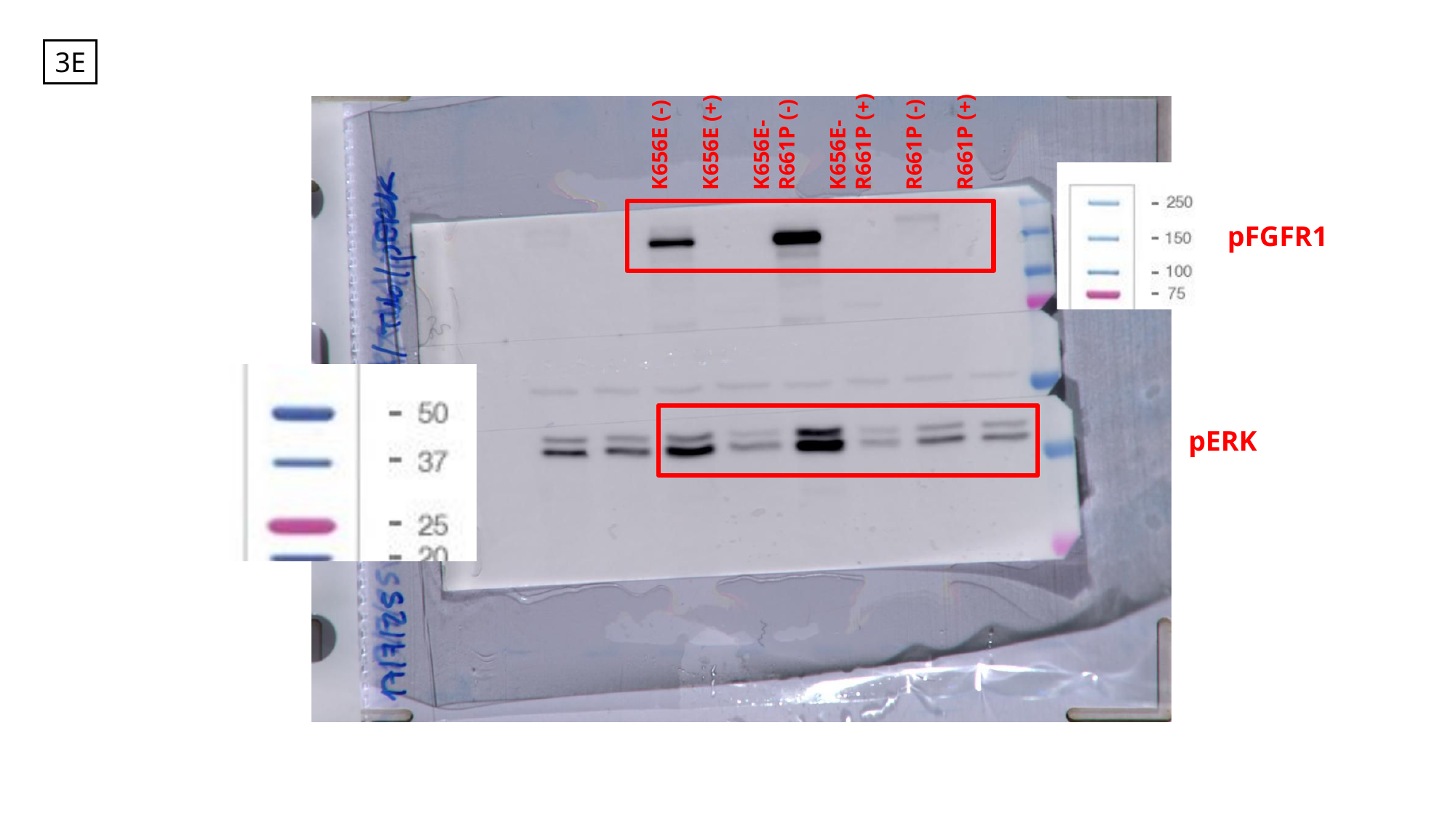

K656E (-)
K656E (+)
K656E-R661P (-)
K656E-R661P (+)
R661P (-)
R661P (+)
3E
pFGFR1
pERK

## Slide 13
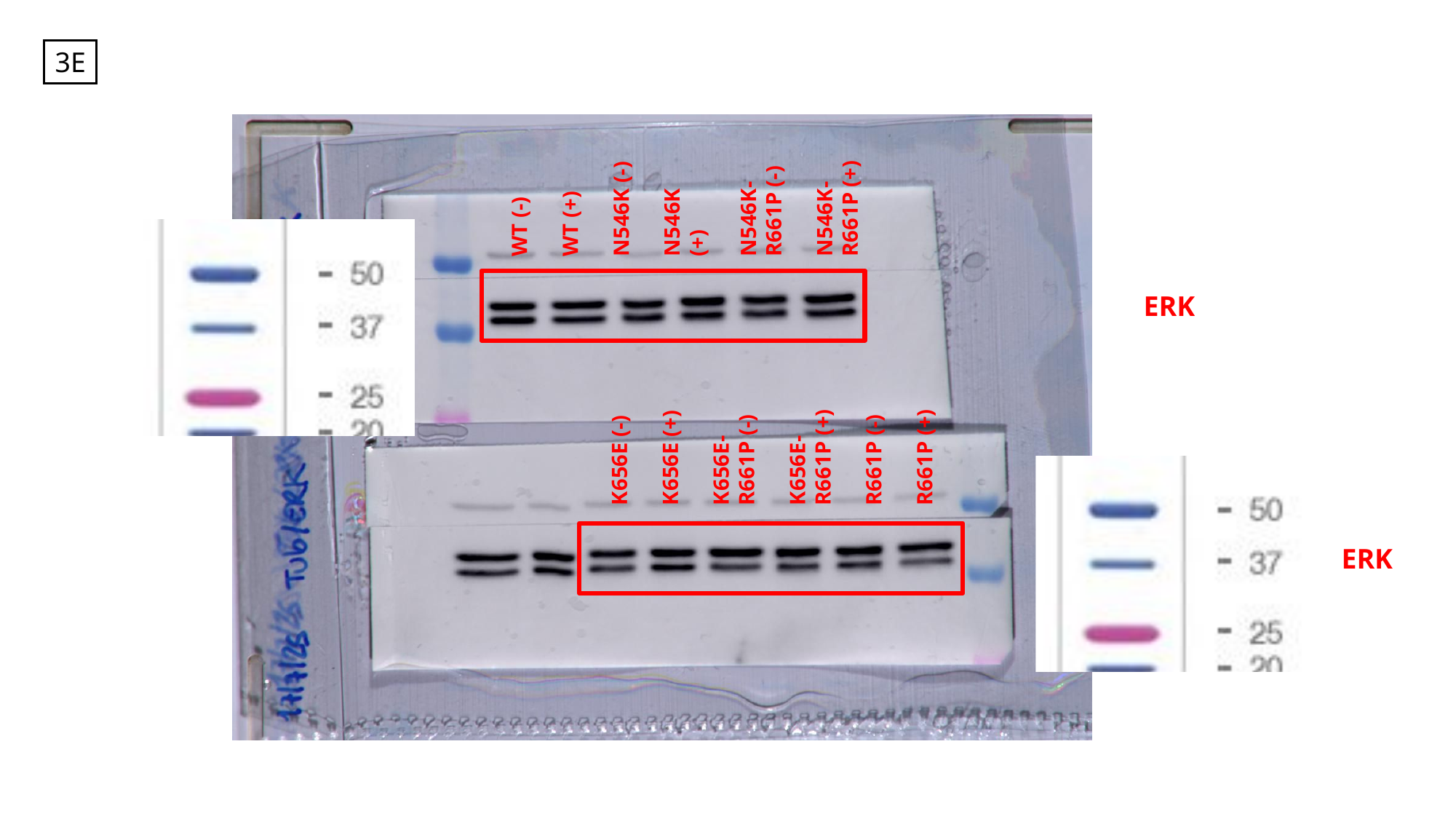

WT (-)
WT (+)
N546K (-)
N546K (+)
N546K-R661P (-)
N546K-R661P (+)
3E
K656E (-)
K656E (+)
K656E-R661P (-)
K656E-R661P (+)
R661P (-)
R661P (+)
ERK
ERK

## Slide 14
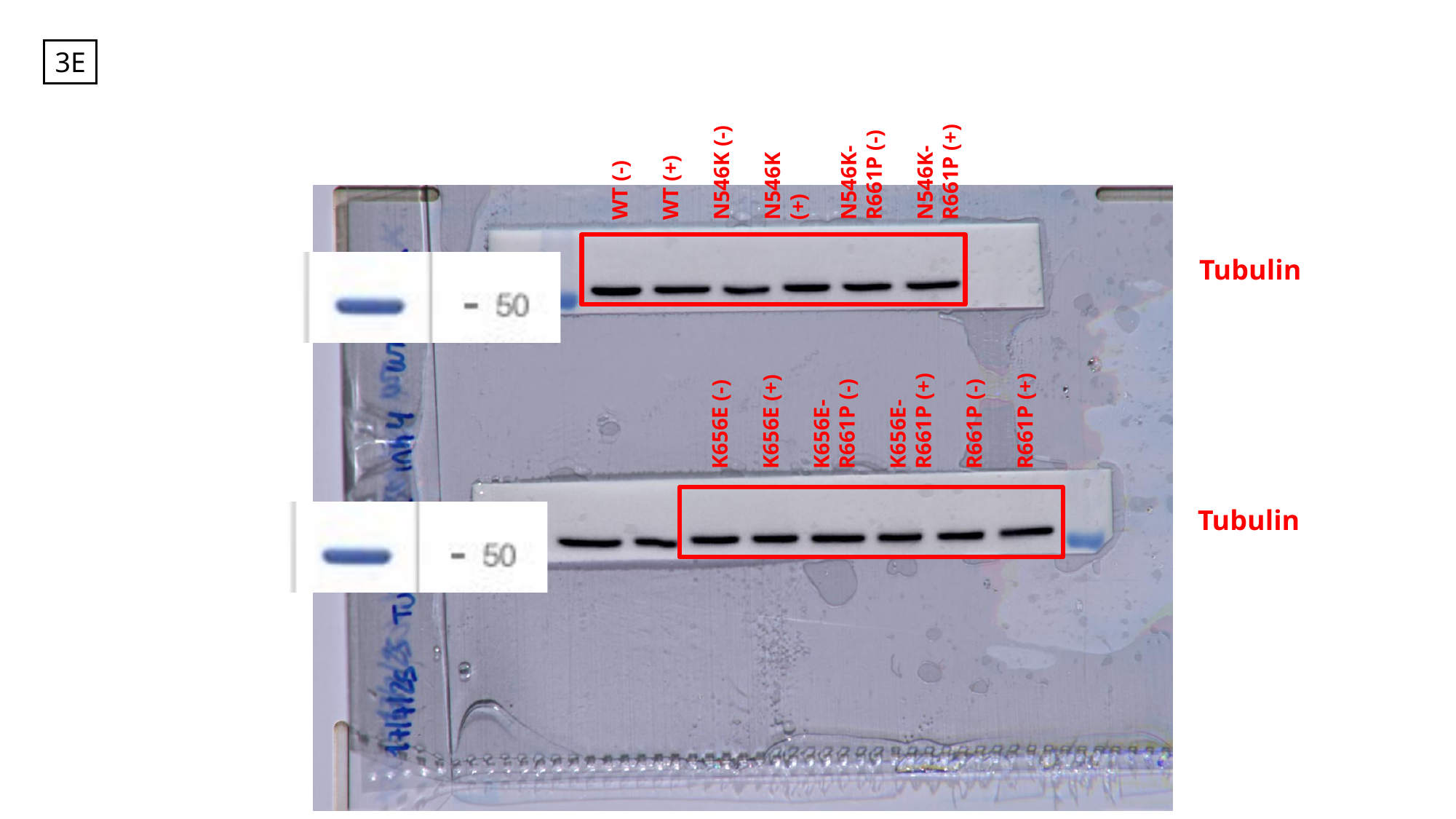

WT (-)
WT (+)
N546K (-)
N546K (+)
N546K-R661P (-)
N546K-R661P (+)
3E
K656E (-)
K656E (+)
K656E-R661P (-)
K656E-R661P (+)
R661P (-)
R661P (+)
Tubulin
Tubulin
