# Supplementary material for: Concurrence of FGFR1 mutations modulates oncogenesis in glioneuronal tumors
Source: EMBO J. 2025 Oct 31;44(24):7513–40. doi: 10.1038/s44318-025-00600-3 (PMC12705663; doi:10.1038/s44318-025-00600-3)
Supplement: Supplementary file 7 — Source data Fig. 4 [file 44318_2025_600_MOESM7_ESM.zip › Figure 4/4E/WB 4A-C-E.pptx]

## Slide 1
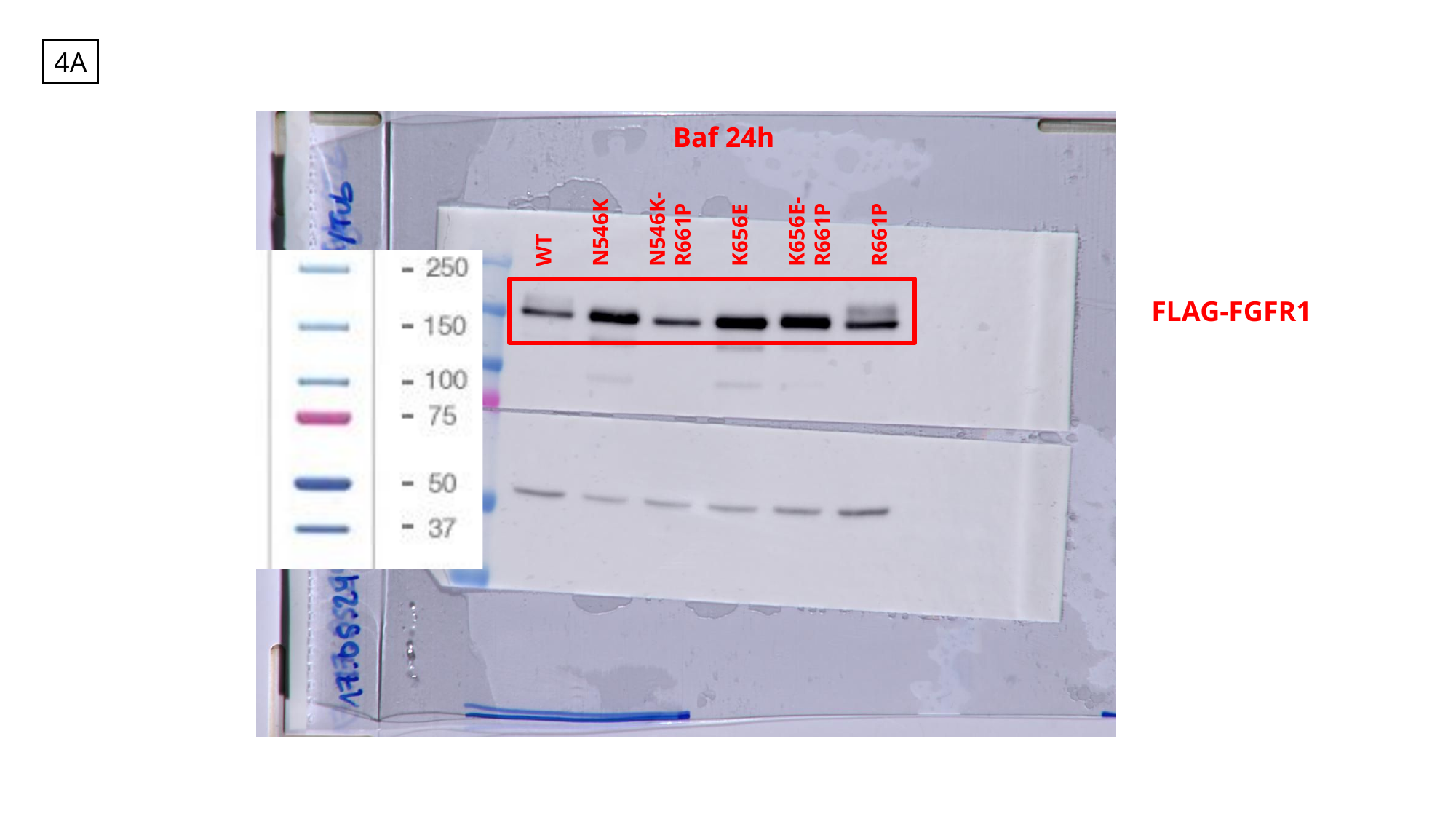

WT
N546K
N546K-R661P
K656E
K656E-R661P
R661P
4A
Baf 24h
FLAG-FGFR1

## Slide 2
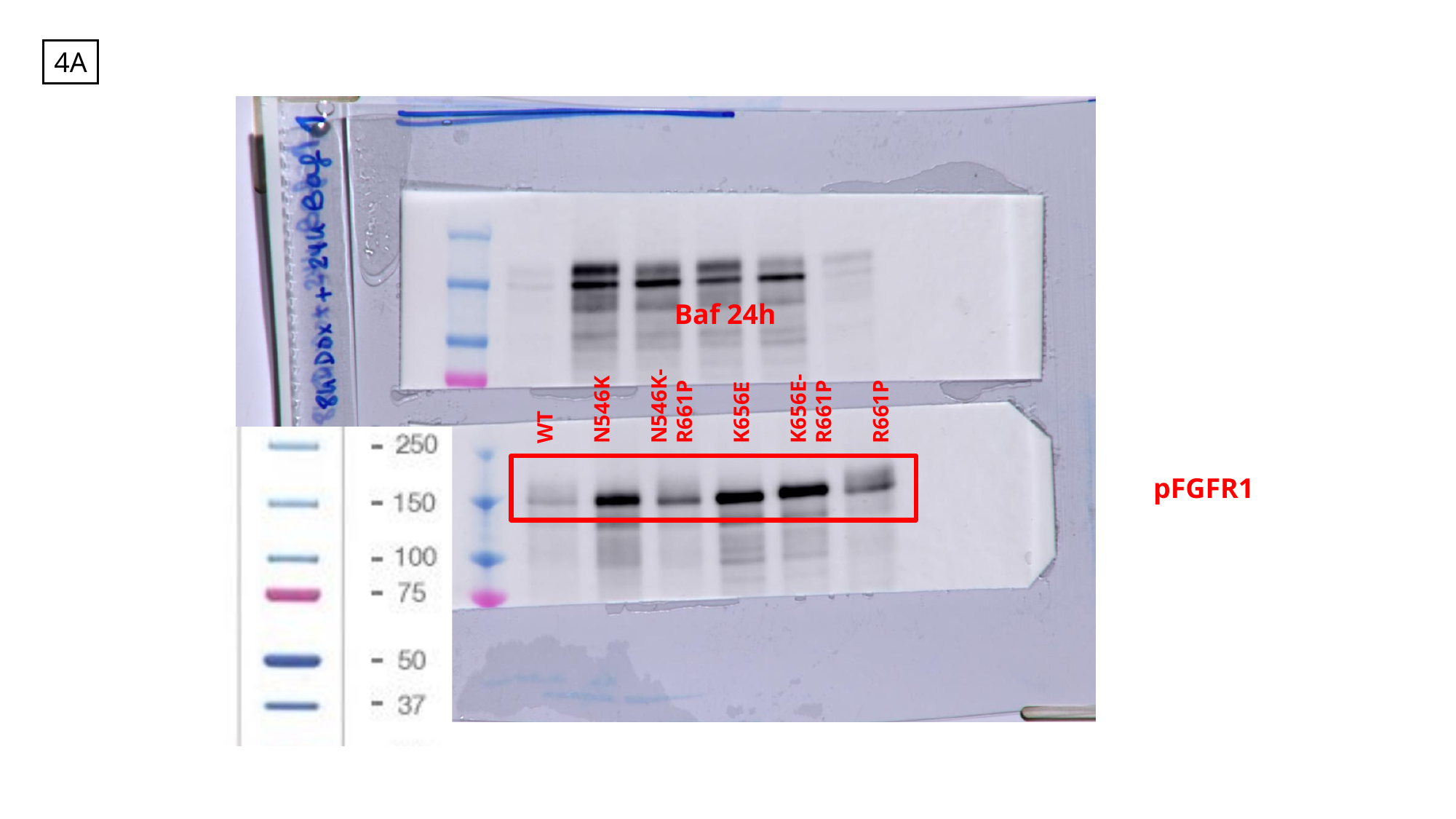

4A
WT
N546K
N546K-R661P
K656E
K656E-R661P
R661P
Baf 24h
pFGFR1

## Slide 3
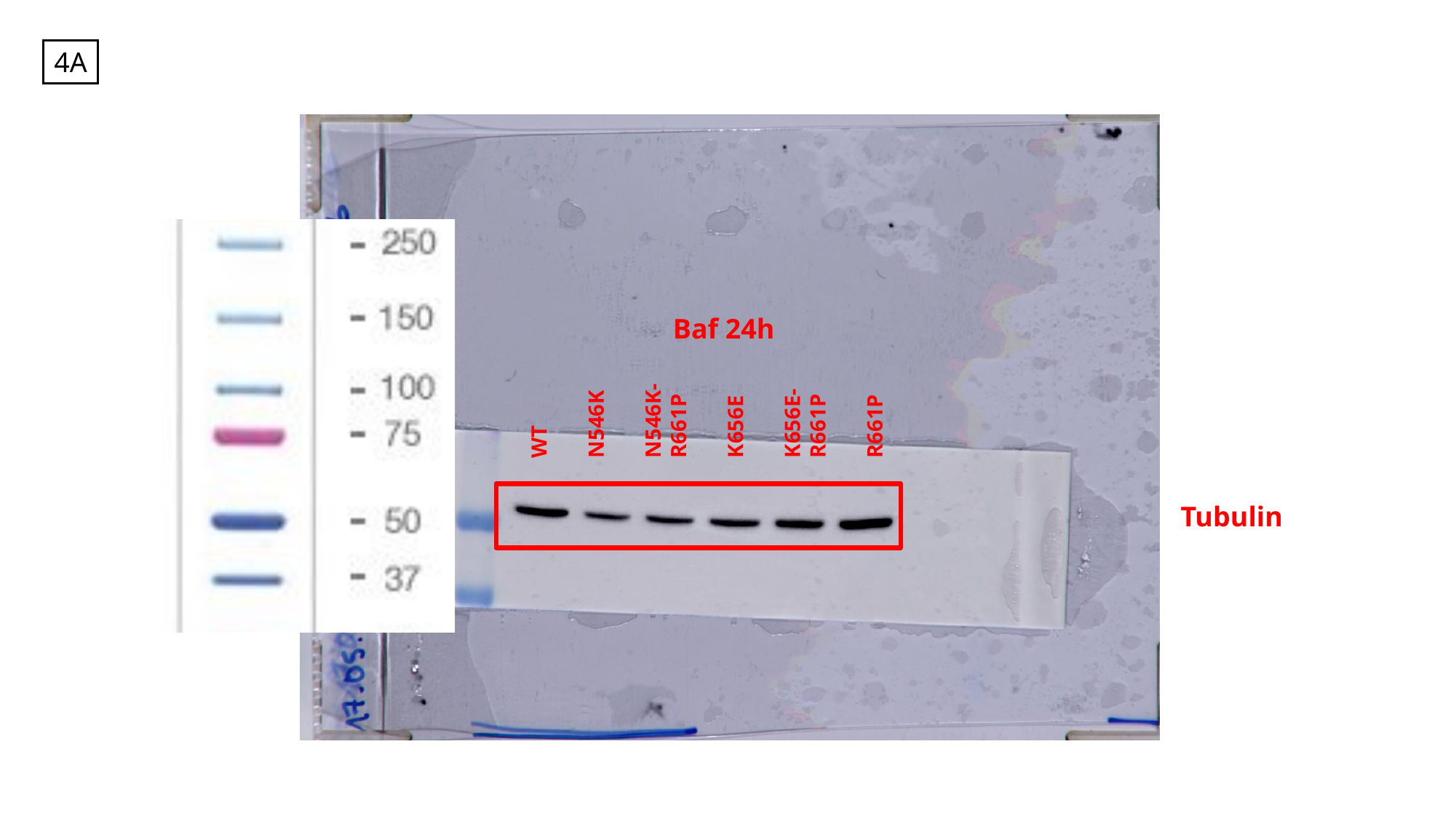

4A
WT
N546K
N546K-R661P
K656E
K656E-R661P
R661P
Baf 24h
Tubulin

## Slide 4
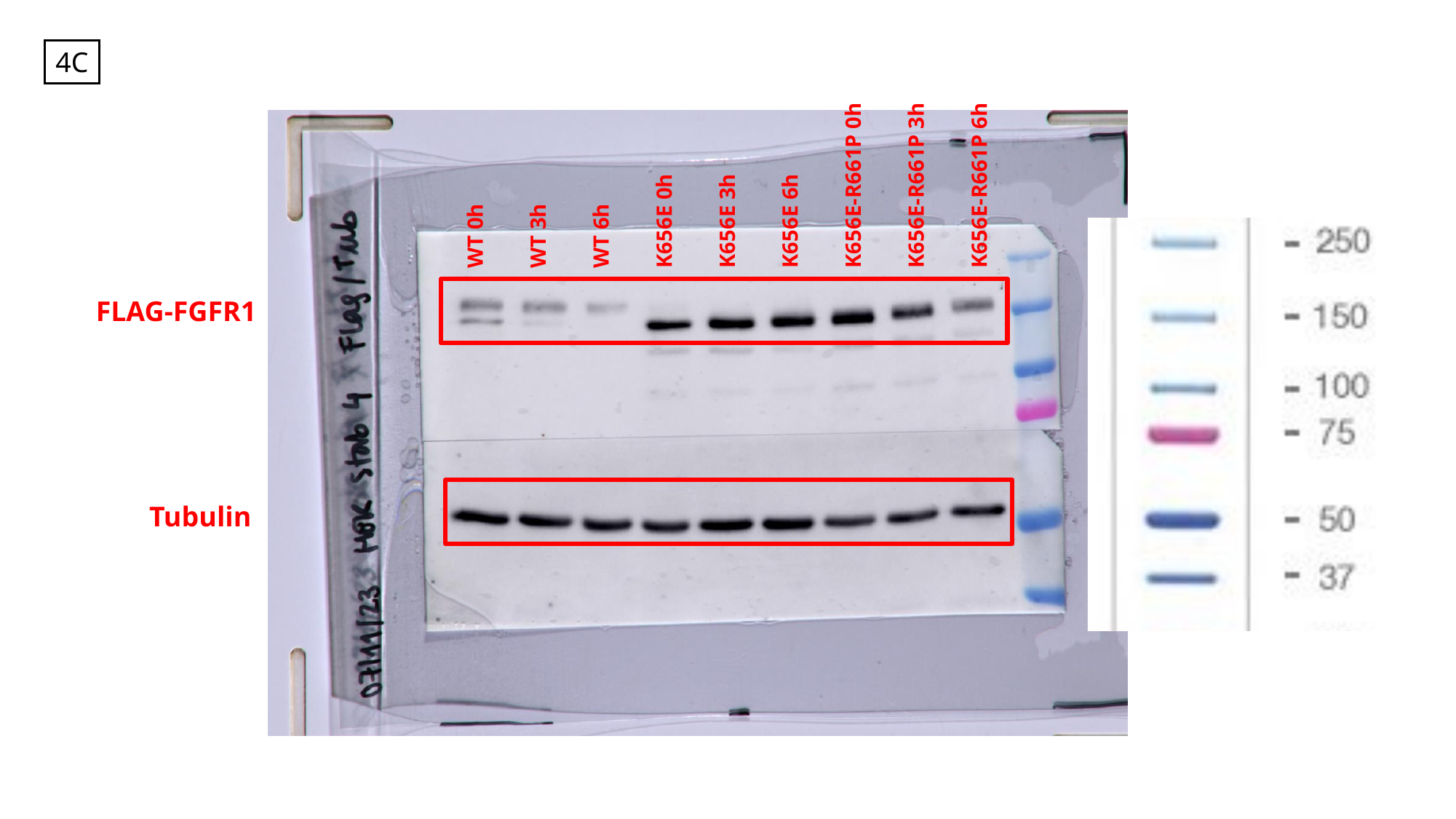

WT 0h
WT 3h
WT 6h
K656E 0h
K656E 3h
K656E 6h
K656E-R661P 0h
K656E-R661P 3h
K656E-R661P 6h
4C
FLAG-FGFR1
Tubulin

## Slide 5
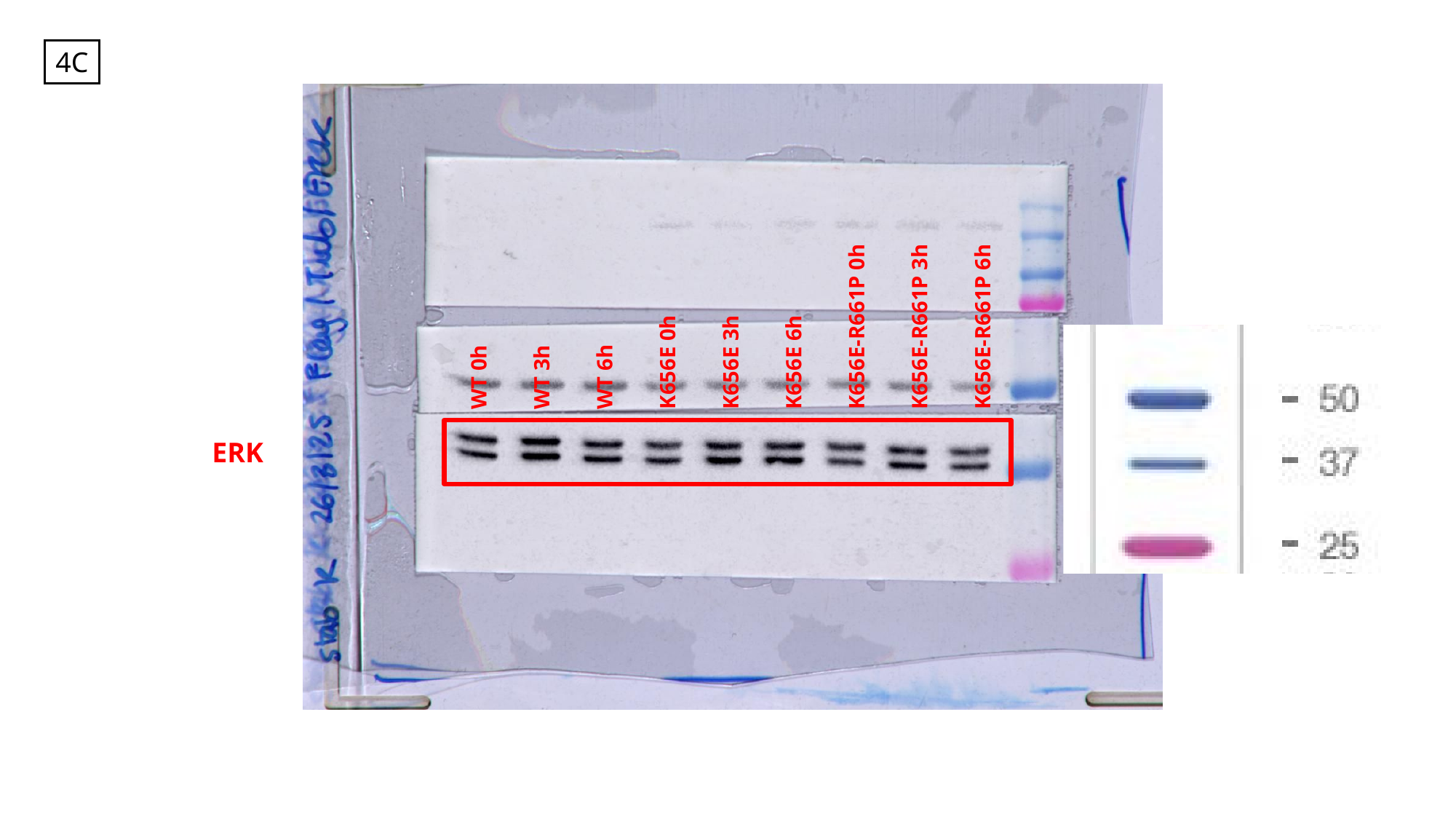

4C
WT 0h
WT 3h
WT 6h
K656E 0h
K656E 3h
K656E 6h
K656E-R661P 0h
K656E-R661P 3h
K656E-R661P 6h
ERK

## Slide 6
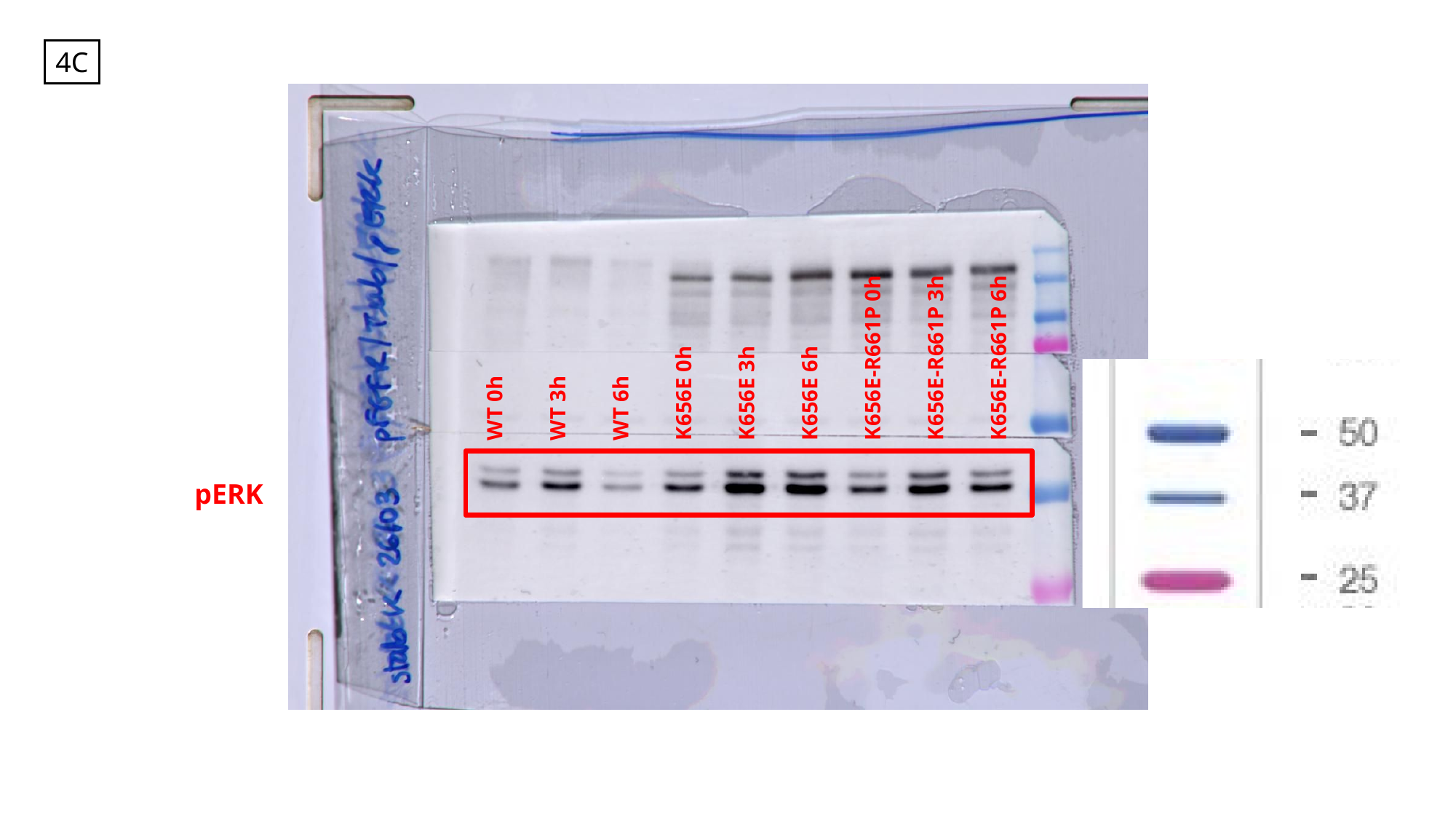

4C
WT 0h
WT 3h
WT 6h
K656E 0h
K656E 3h
K656E 6h
K656E-R661P 0h
K656E-R661P 3h
K656E-R661P 6h
pERK

## Slide 7
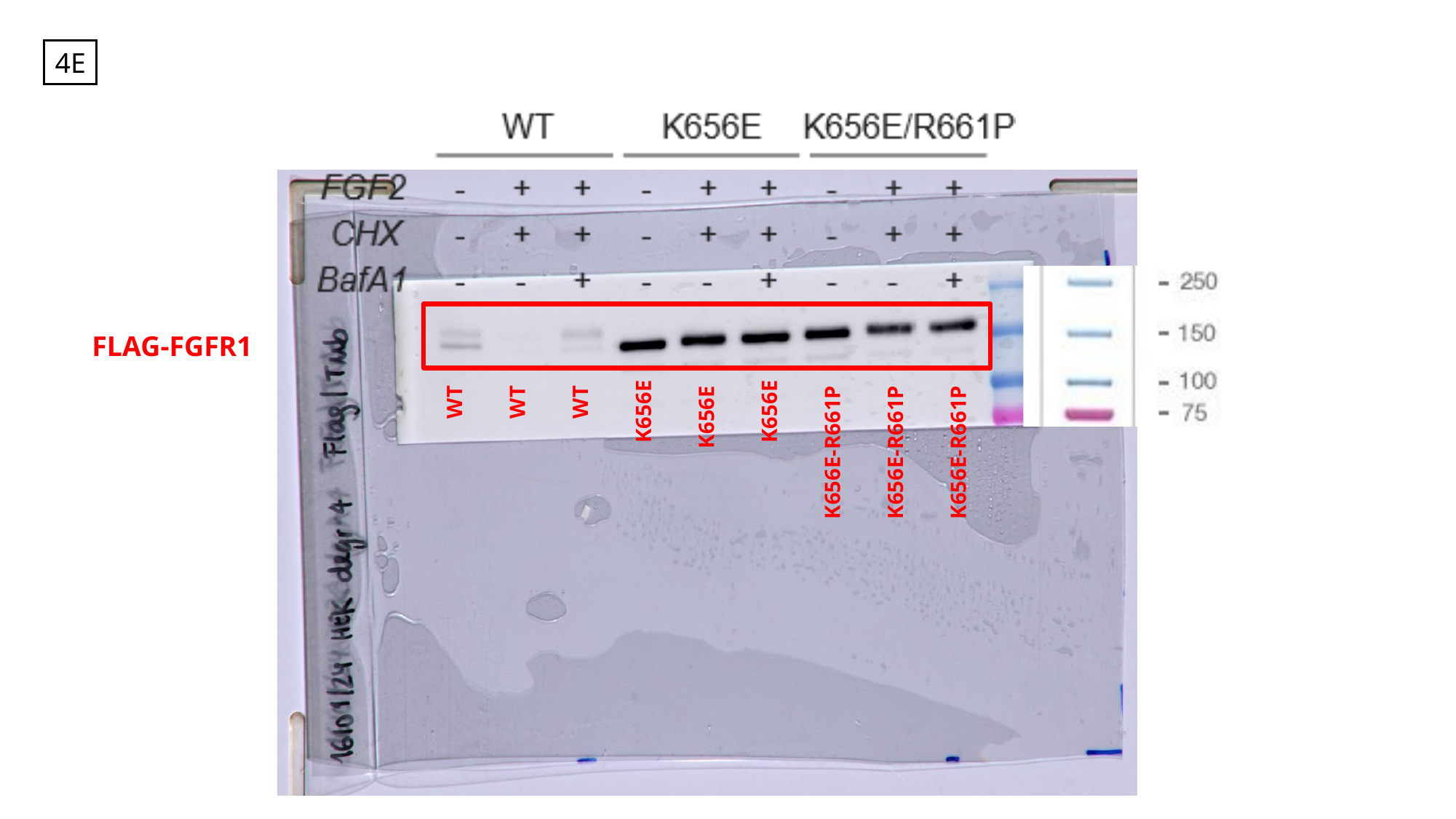

4E
WT
WT
WT
K656E
K656E
K656E
K656E-R661P
K656E-R661P
K656E-R661P
FLAG-FGFR1

## Slide 8
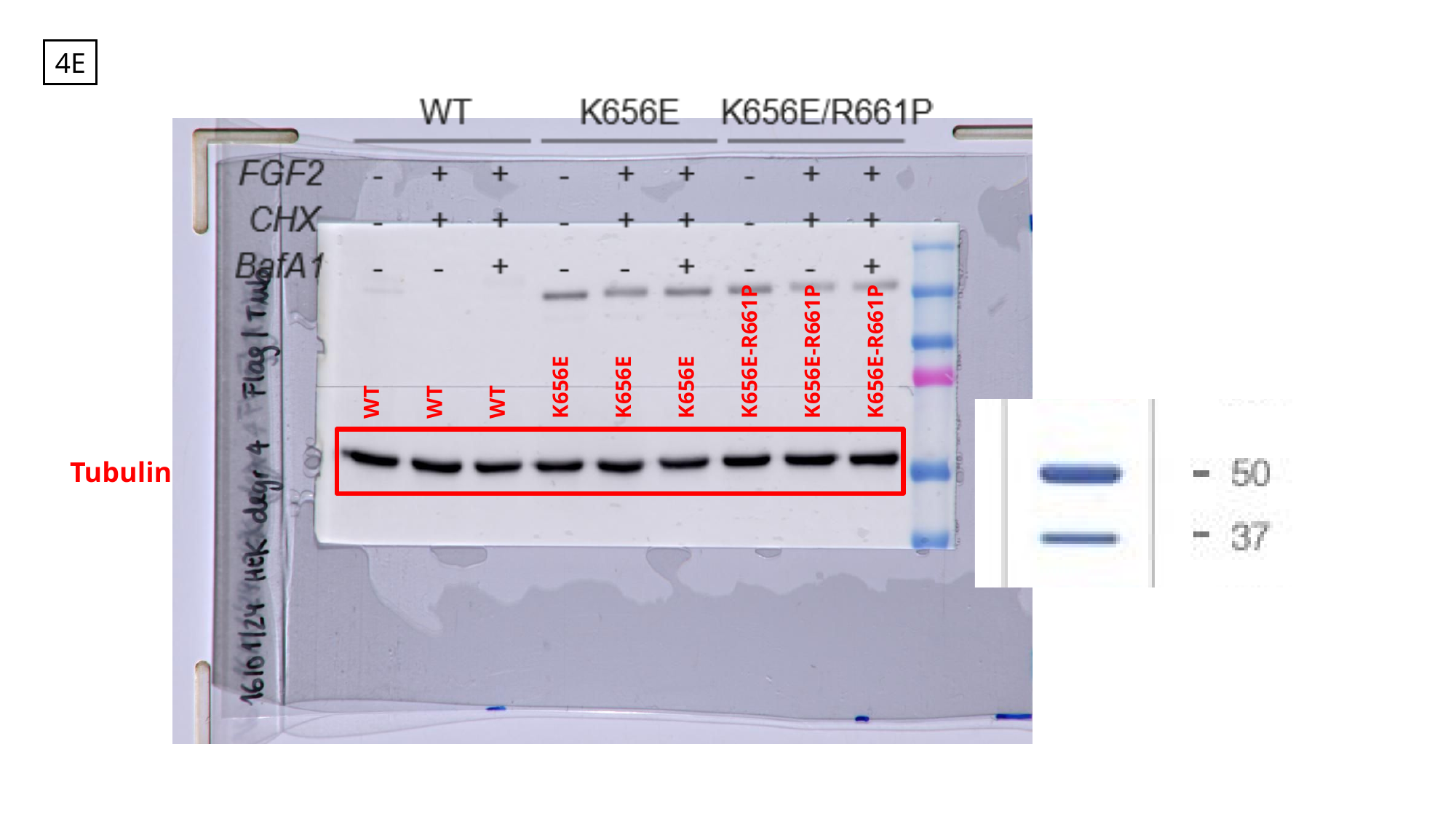

4E
WT
WT
WT
K656E
K656E
K656E
K656E-R661P
K656E-R661P
K656E-R661P
Tubulin
