# Supplementary figures and images for: Concurrence of FGFR1 mutations modulates oncogenesis in glioneuronal tumors (part 2 of 2)
Source: EMBO J. 2025 Oct 31;44(24):7513–40. doi: 10.1038/s44318-025-00600-3 (PMC12705663; doi:10.1038/s44318-025-00600-3)

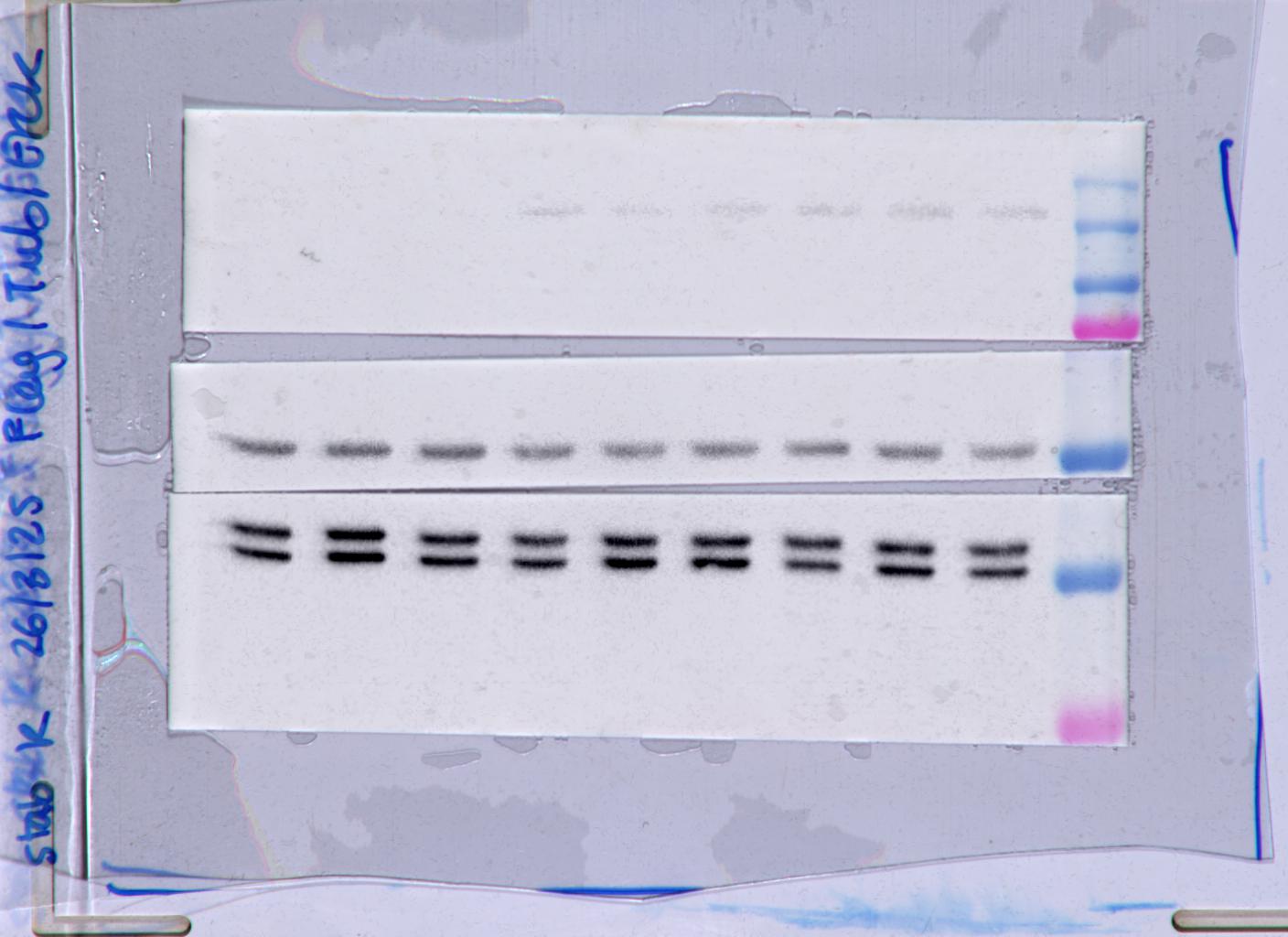

Supplement: Supplementary file 7 — Source data Fig. 4 [file 44318_2025_600_MOESM7_ESM.zip › Figure 4/4C/erk original.jpg]

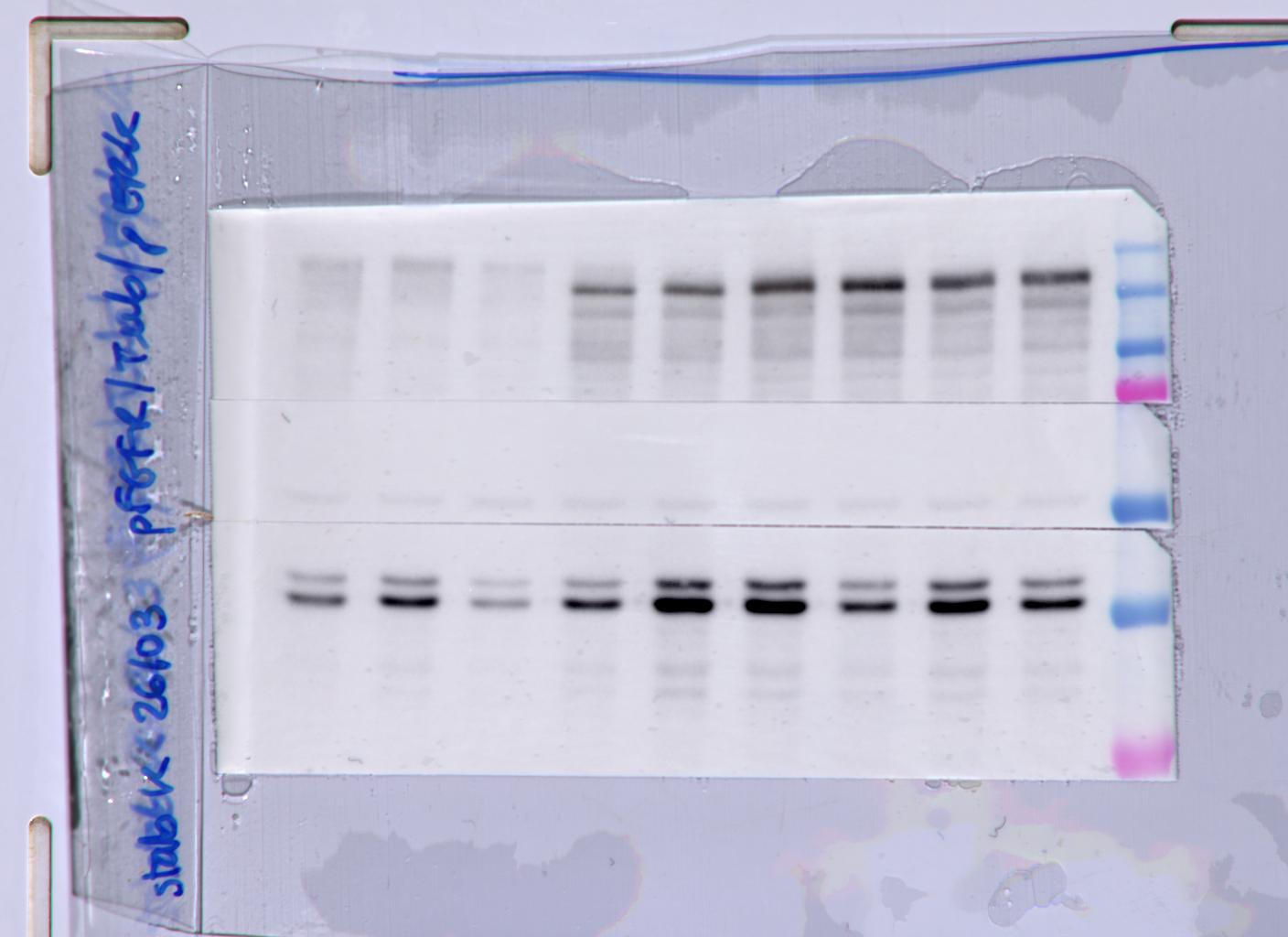

Supplement: Supplementary file 7 — Source data Fig. 4 [file 44318_2025_600_MOESM7_ESM.zip › Figure 4/4C/pERK original.jpg]

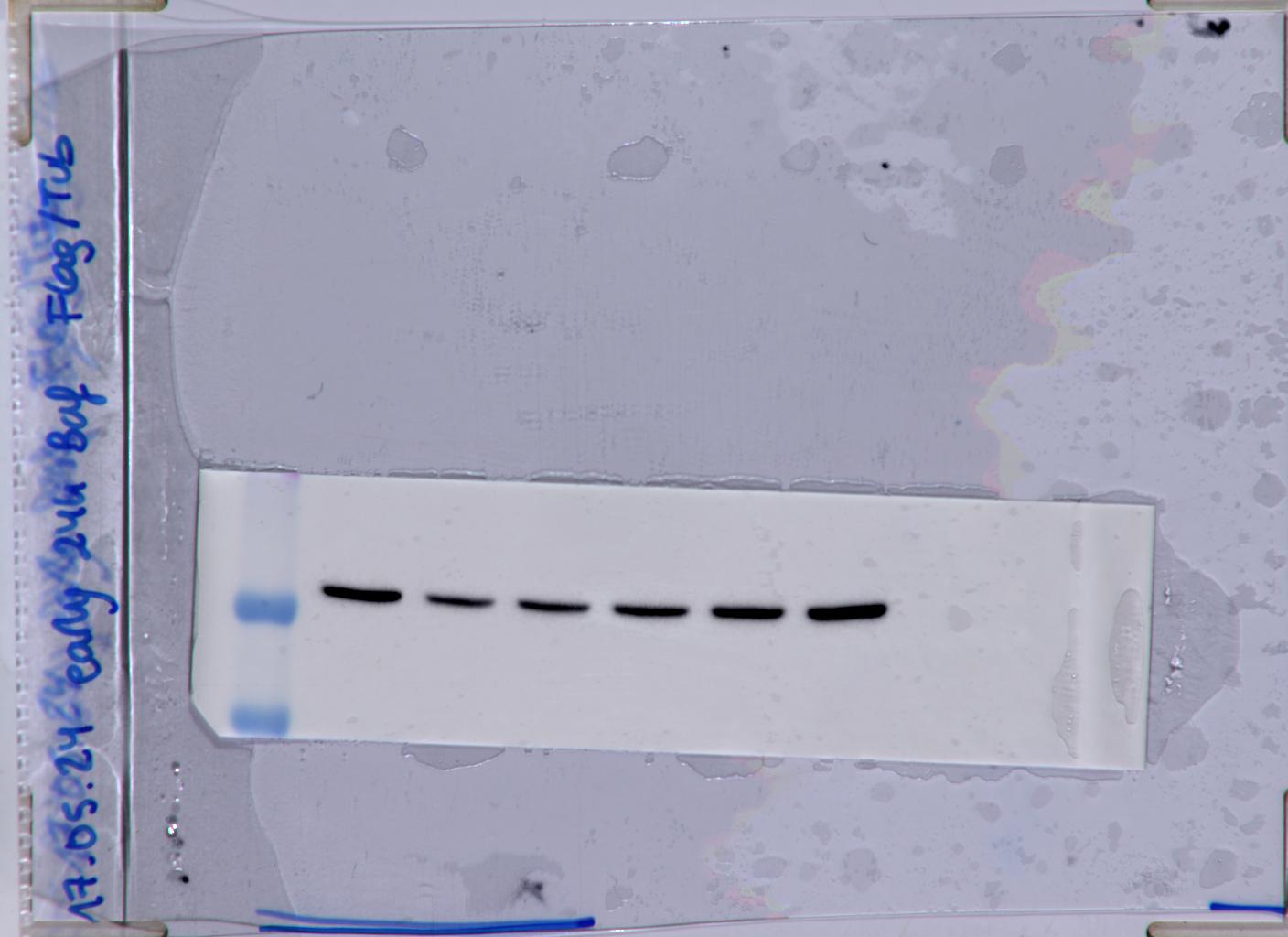

Supplement: Supplementary file 7 — Source data Fig. 4 [file 44318_2025_600_MOESM7_ESM.zip › Figure 4/4A/tub original.jpg]

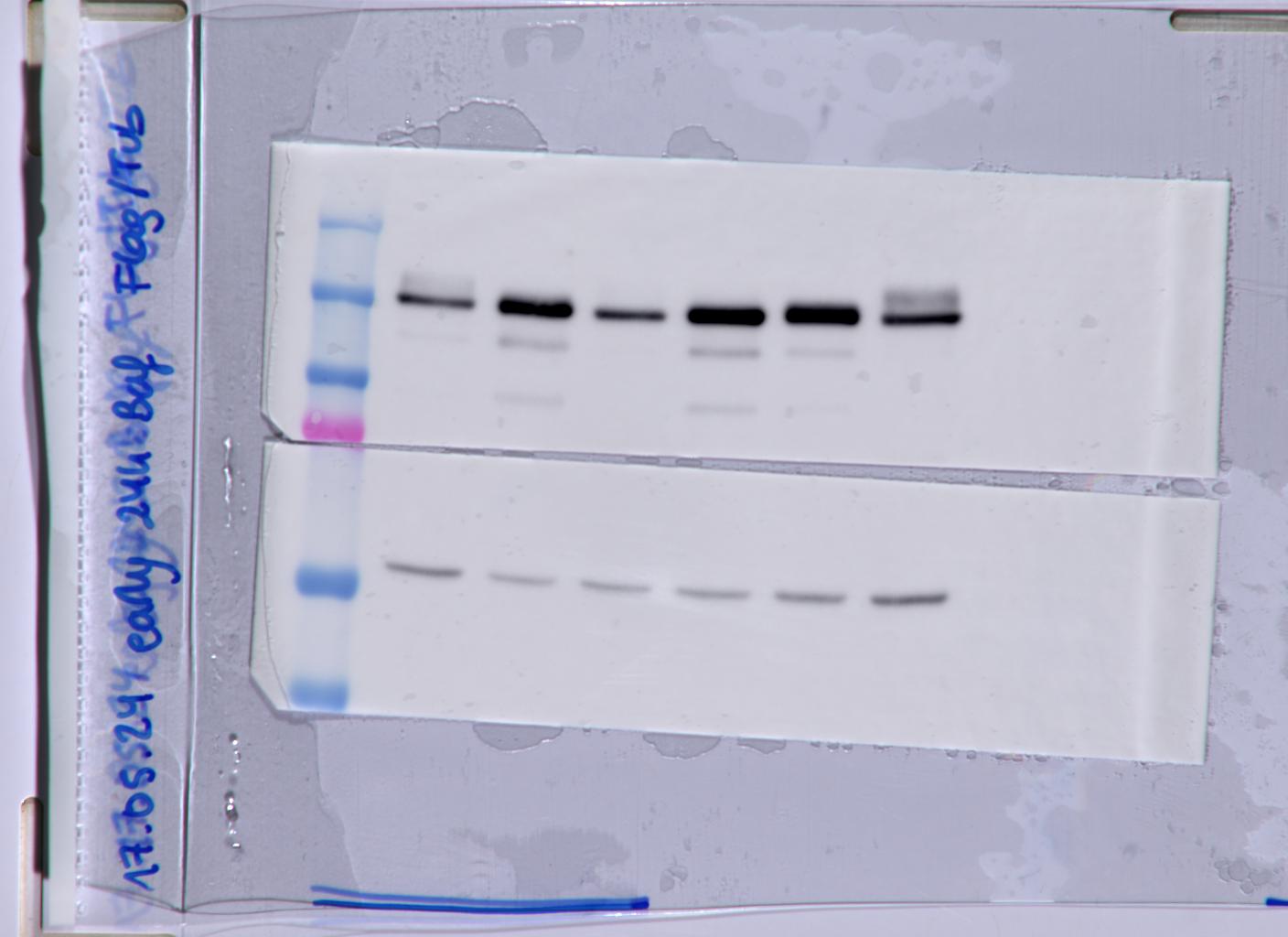

Supplement: Supplementary file 7 — Source data Fig. 4 [file 44318_2025_600_MOESM7_ESM.zip › Figure 4/4A/flag original.jpg]

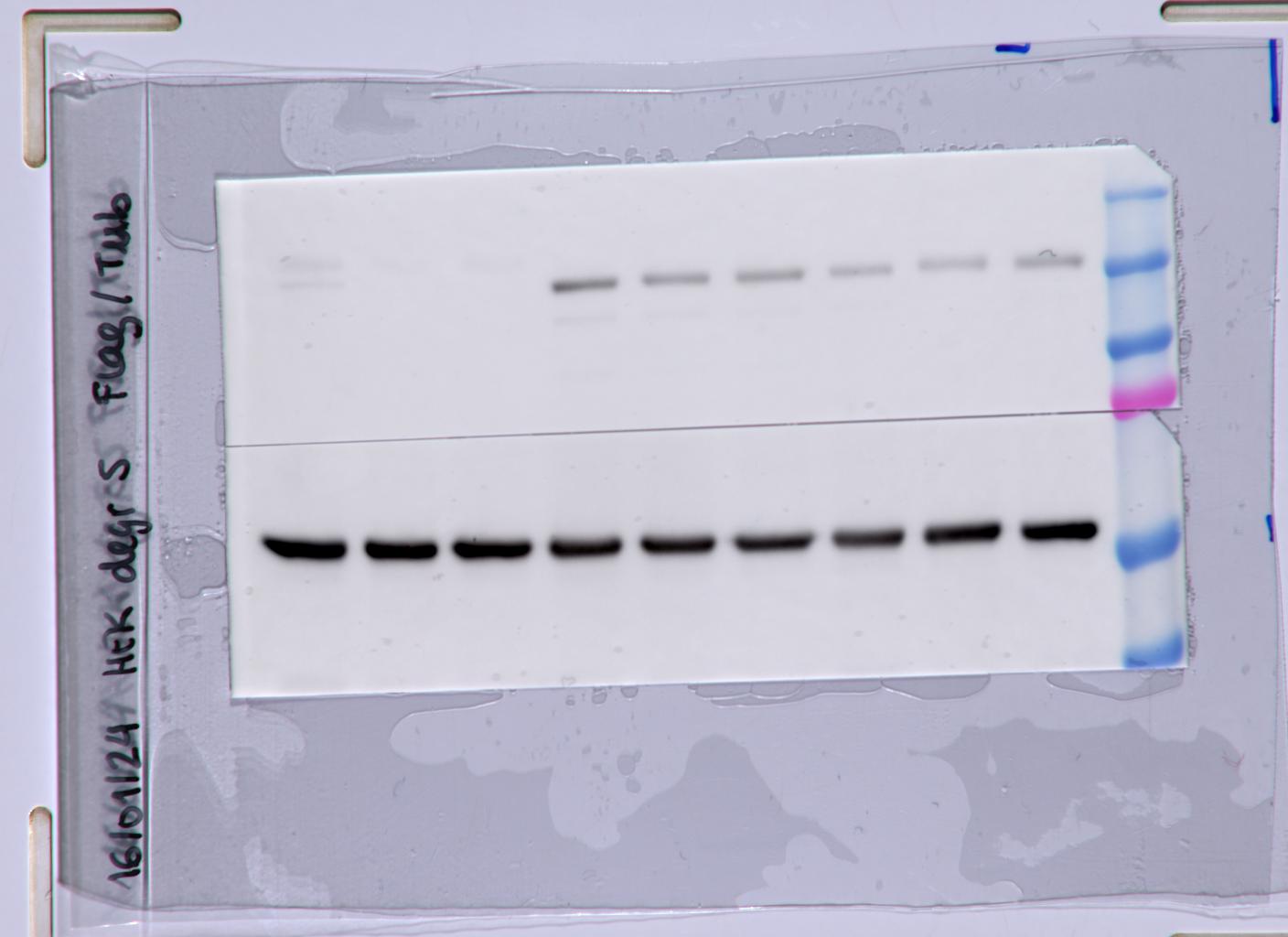

Supplement: Supplementary file 7 — Source data Fig. 4 [file 44318_2025_600_MOESM7_ESM.zip › Figure 4/4E/4E replicates/degr5.flagtub 1s 2024.01.16_11.48.06_Ch+Marker.jpg]

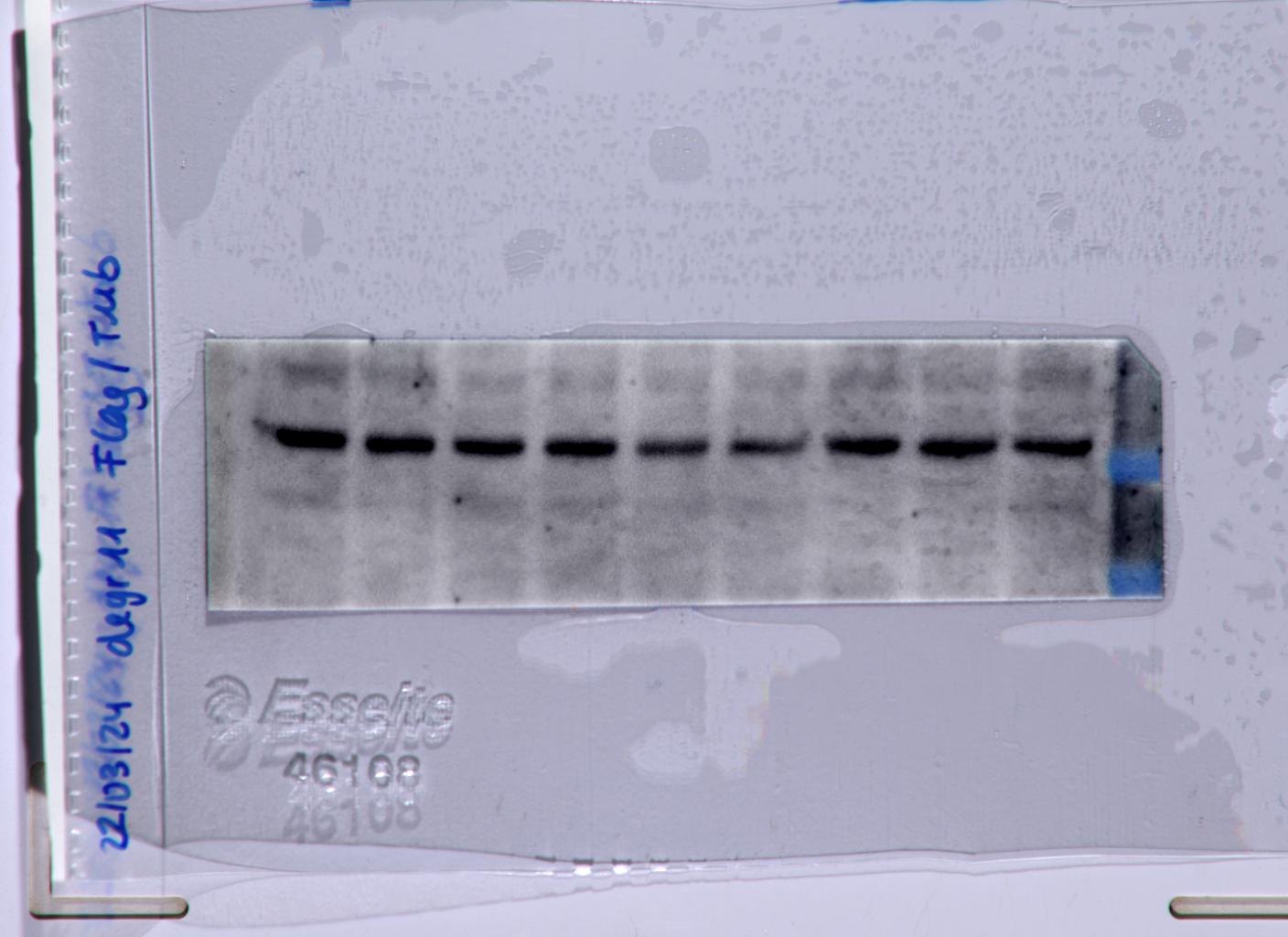

Supplement: Supplementary file 7 — Source data Fig. 4 [file 44318_2025_600_MOESM7_ESM.zip › Figure 4/4E/4E replicates/hek degr11 tub 8s 2024.03.22_11.22.57_Ch+Marker.jpg]

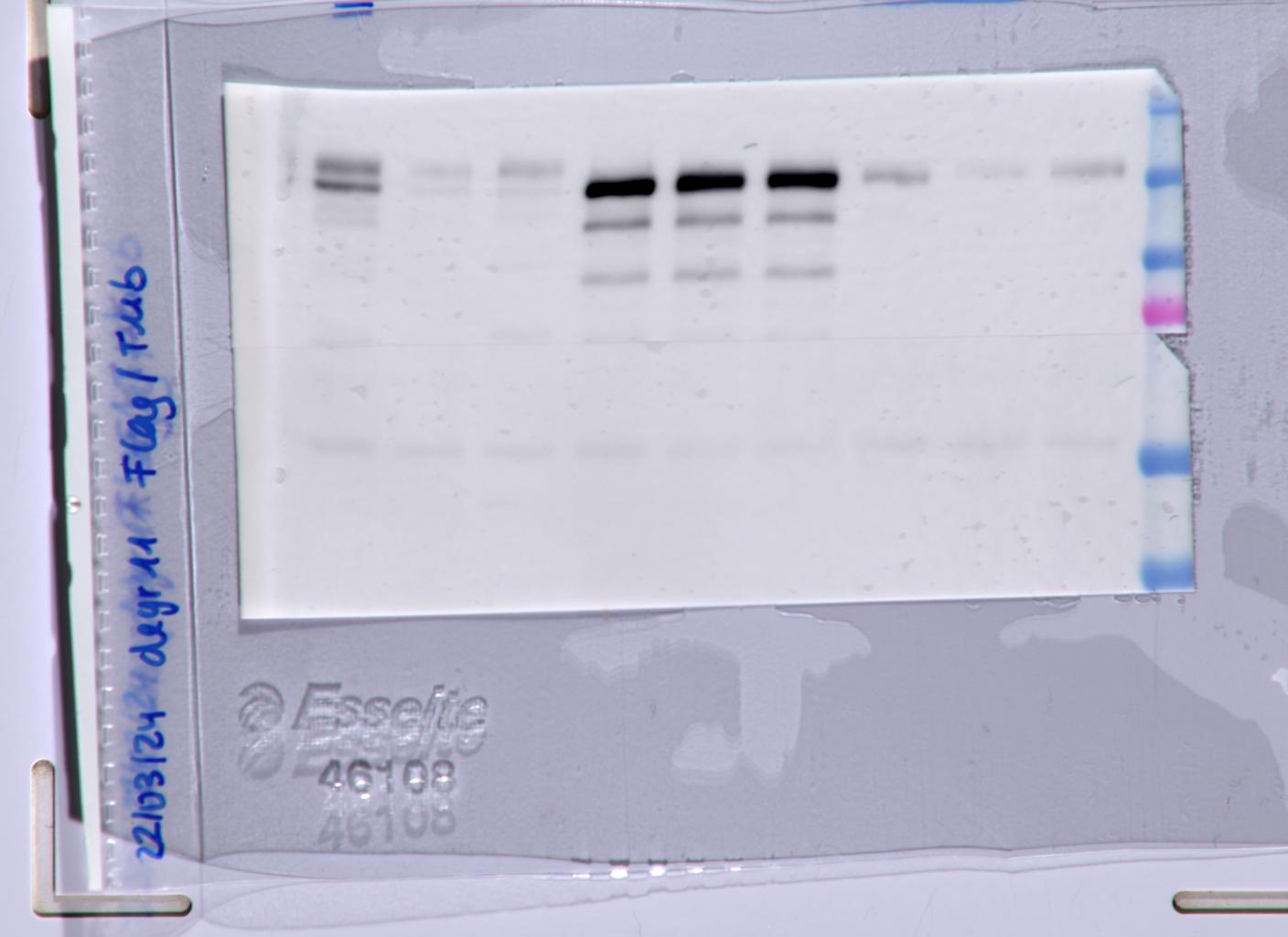

Supplement: Supplementary file 7 — Source data Fig. 4 [file 44318_2025_600_MOESM7_ESM.zip › Figure 4/4E/4E replicates/hek degr11 flgtub 6s 2024.03.22_11.16.12_Ch+Marker.jpg]

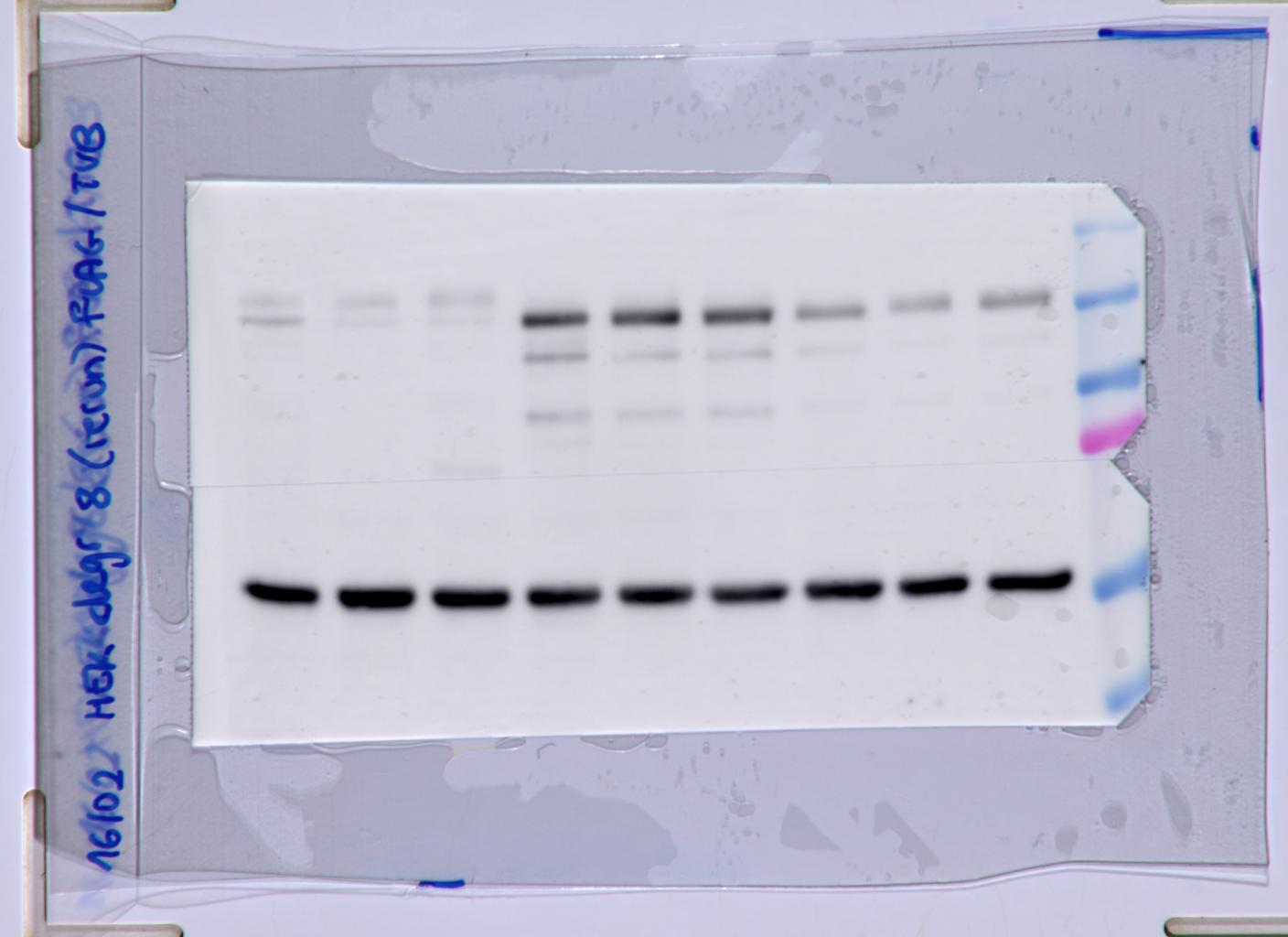

Supplement: Supplementary file 7 — Source data Fig. 4 [file 44318_2025_600_MOESM7_ESM.zip › Figure 4/4E/4E replicates/hek degr8 flagtub 2s 2024.02.16_12.34.41_Ch+Marker.jpg]

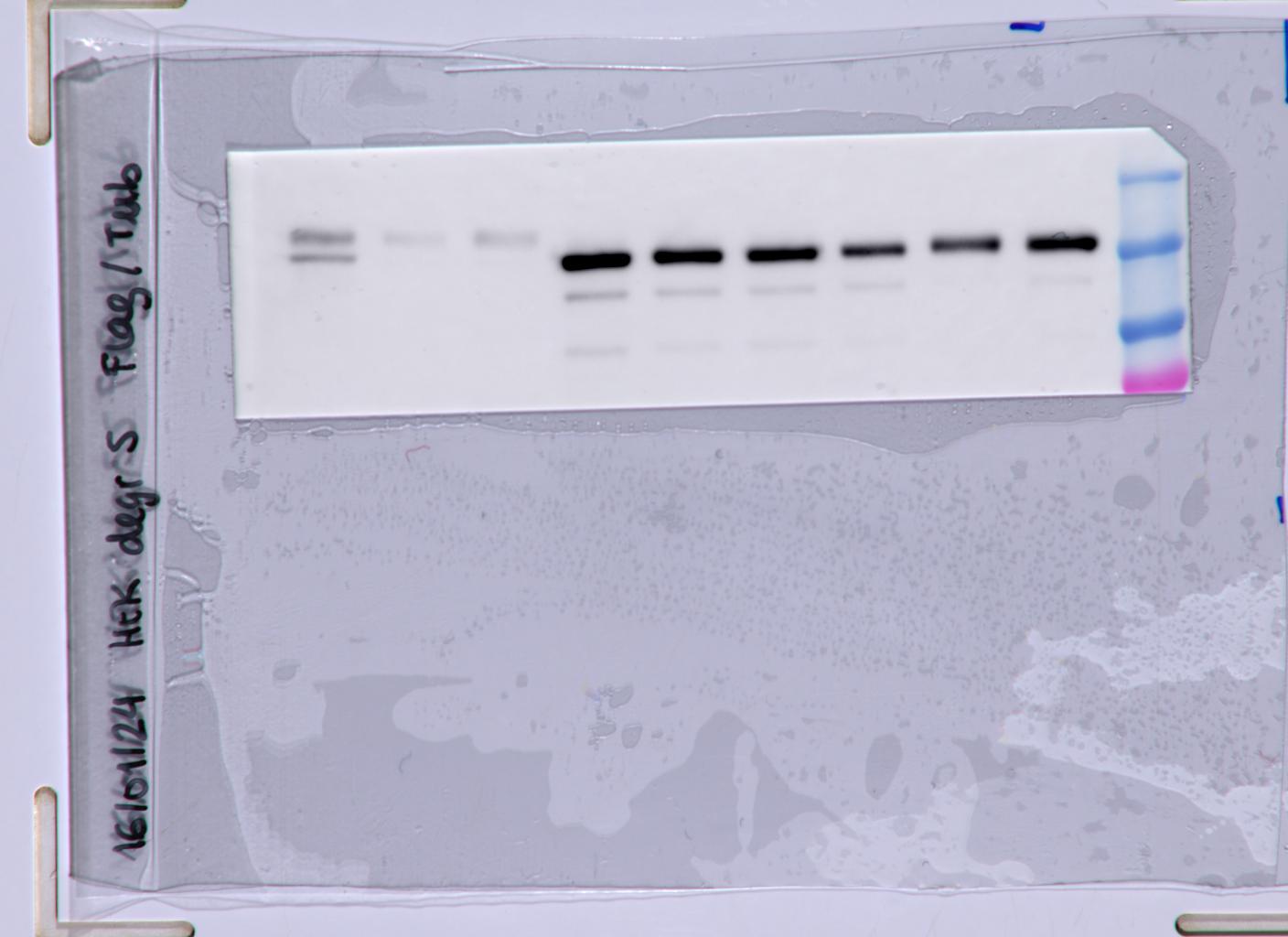

Supplement: Supplementary file 7 — Source data Fig. 4 [file 44318_2025_600_MOESM7_ESM.zip › Figure 4/4E/4E replicates/degr5.flag 5s 2024.01.16_11.50.39_Ch+Marker.jpg]

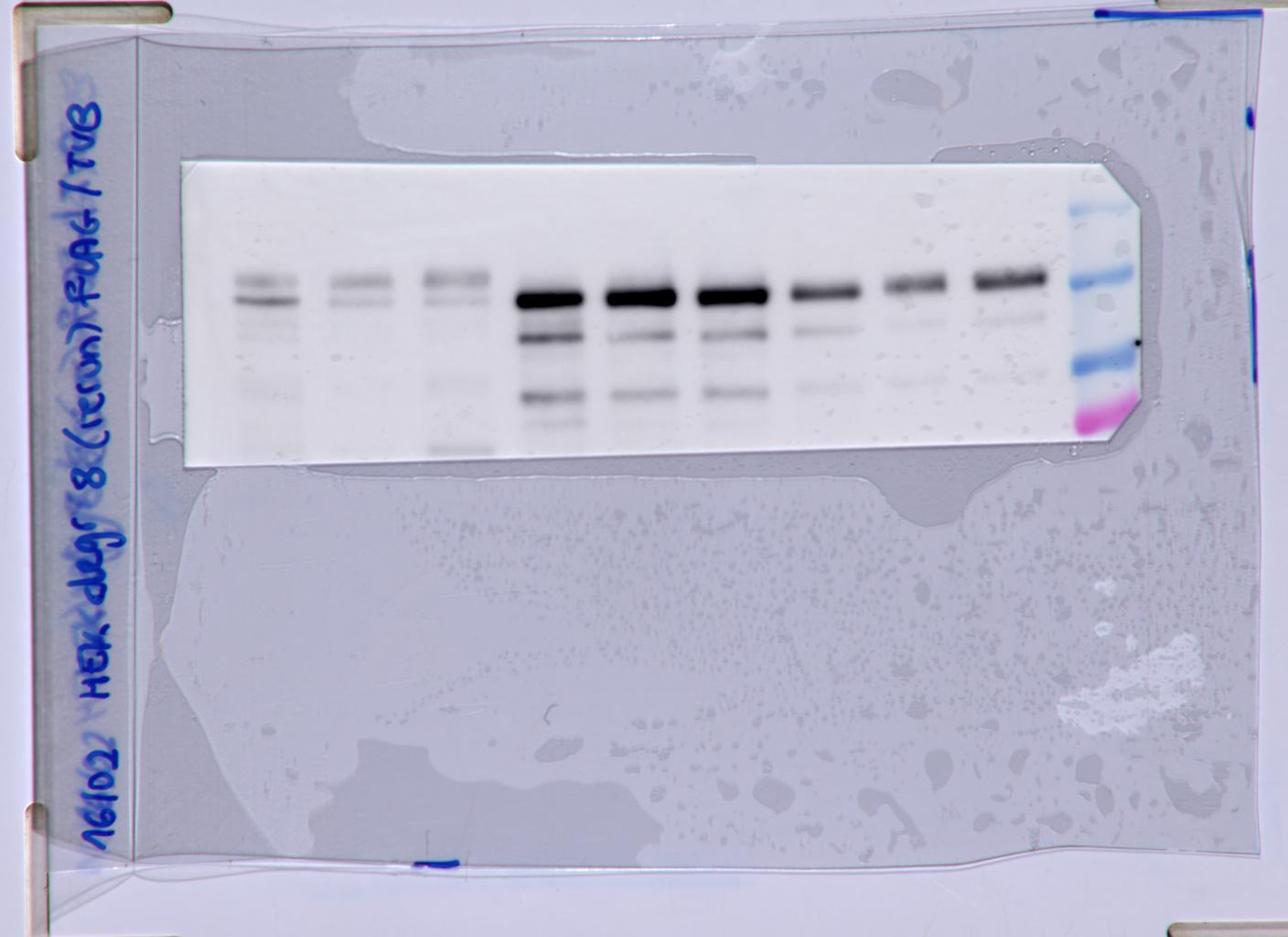

Supplement: Supplementary file 7 — Source data Fig. 4 [file 44318_2025_600_MOESM7_ESM.zip › Figure 4/4E/4E replicates/hek degr8 flag 3s 2024.02.16_12.37.18_Ch+Marker.jpg]

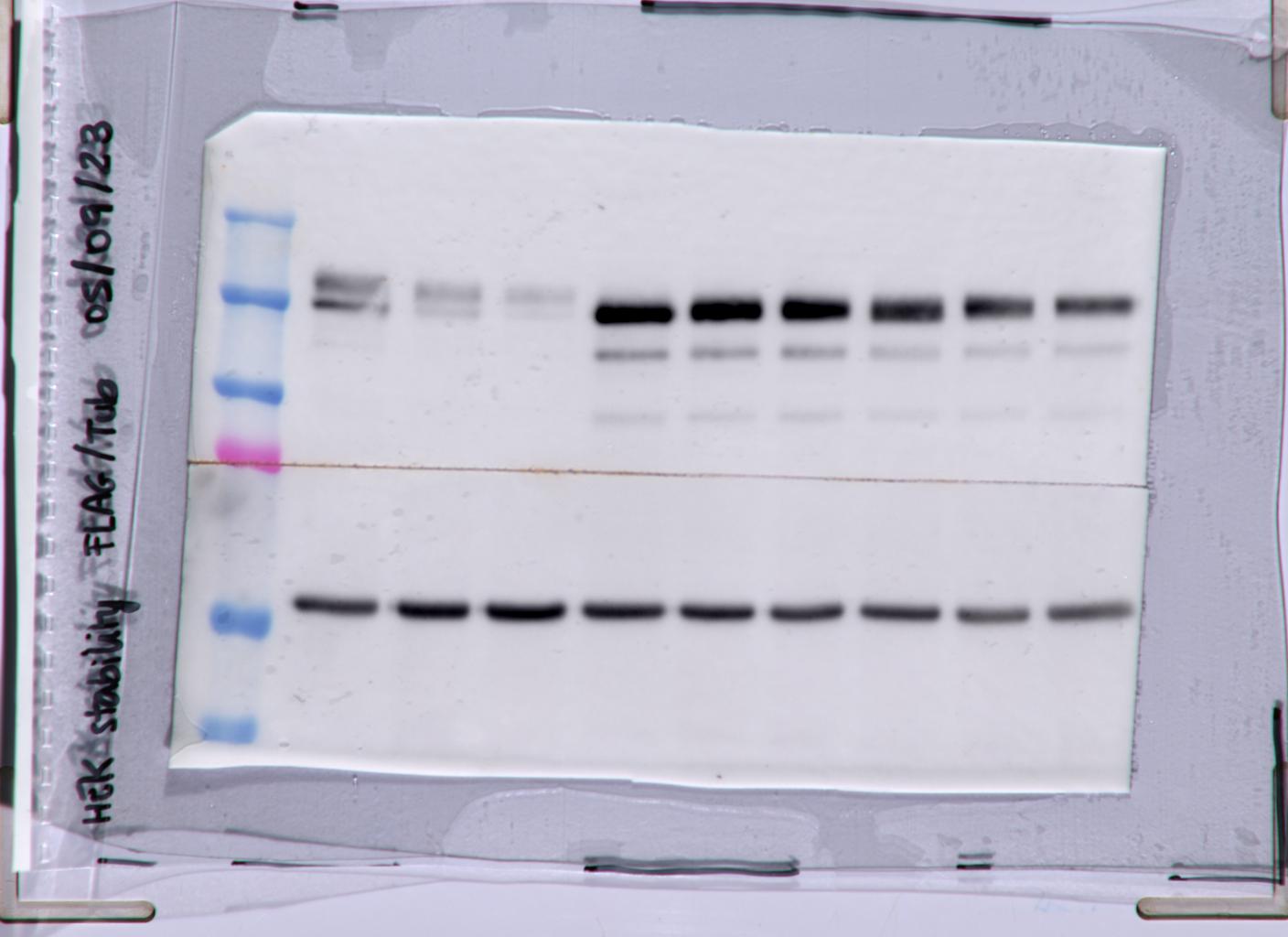

Supplement: Supplementary file 7 — Source data Fig. 4 [file 44318_2025_600_MOESM7_ESM.zip › Figure 4/4C/4C replicates/hek stab flagtub 1s 2023.09.05_11.38.27_Ch+Marker.jpg]

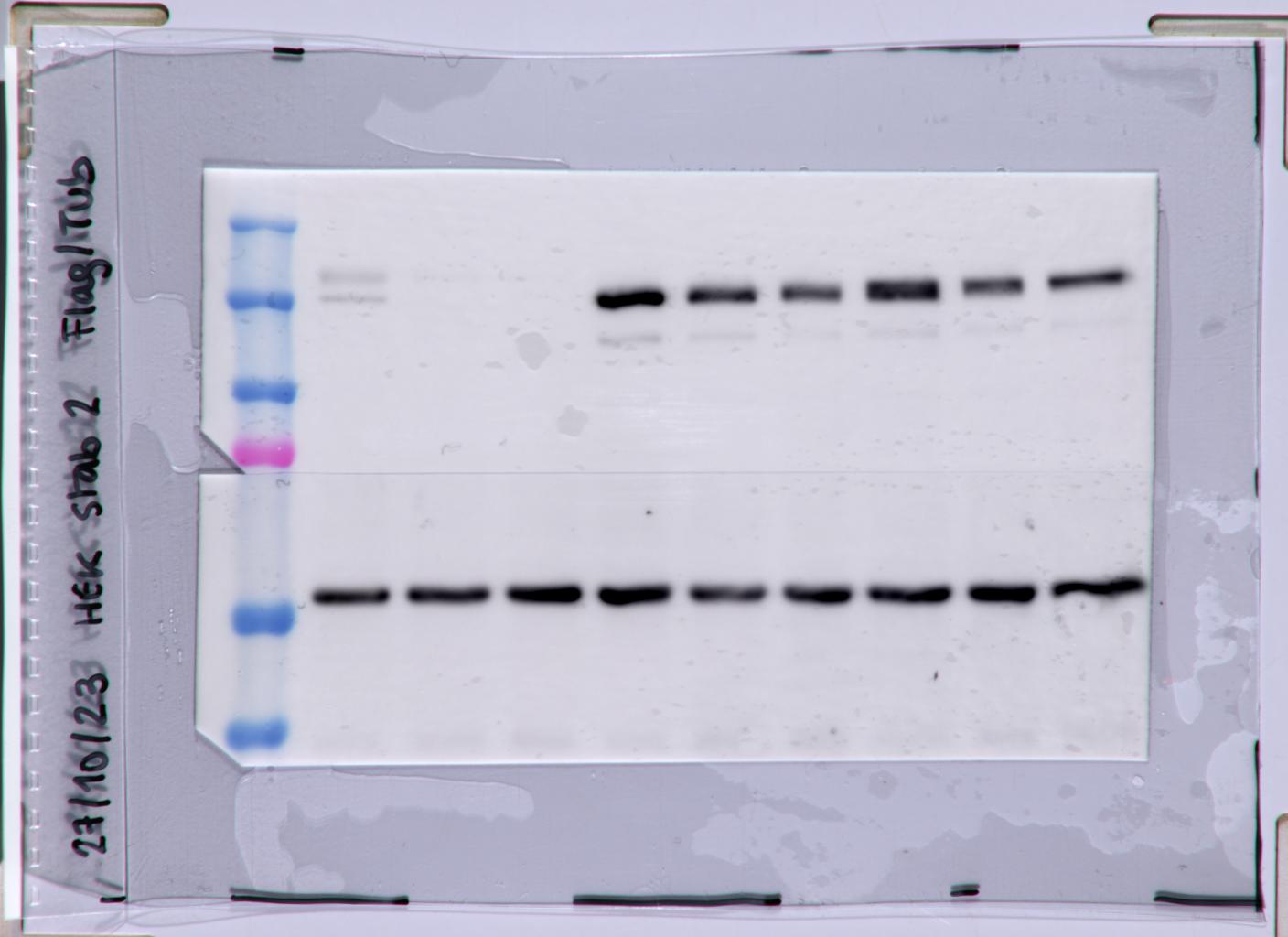

Supplement: Supplementary file 7 — Source data Fig. 4 [file 44318_2025_600_MOESM7_ESM.zip › Figure 4/4C/4C replicates/hek stab 2 flagtub10 2023.10.27_11.30.38_Ch+Marker.jpg]

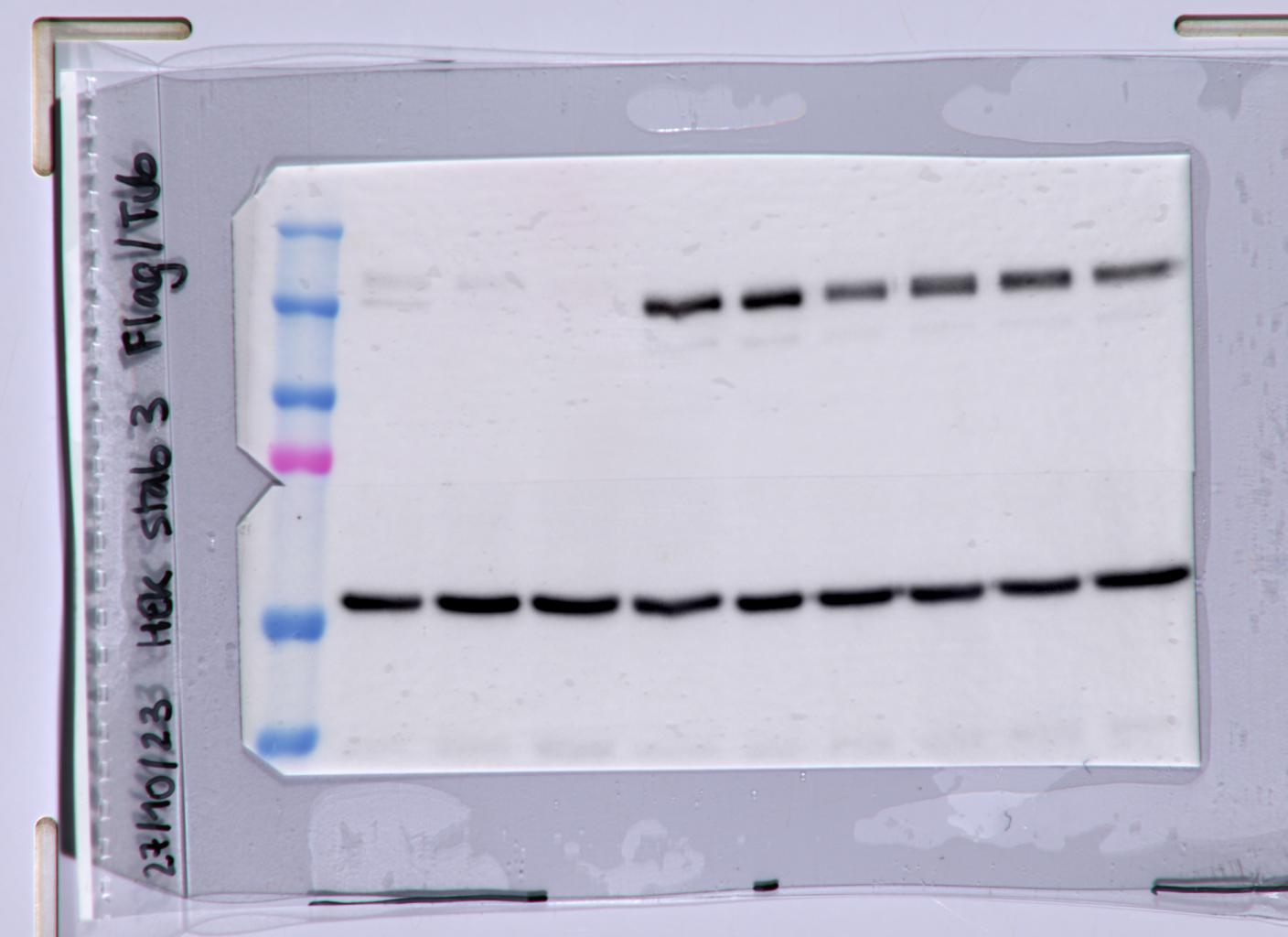

Supplement: Supplementary file 7 — Source data Fig. 4 [file 44318_2025_600_MOESM7_ESM.zip › Figure 4/4C/4C replicates/hek stab3 flagtub 1s 2023.10.27_11.32.25_Ch+Marker.jpg]

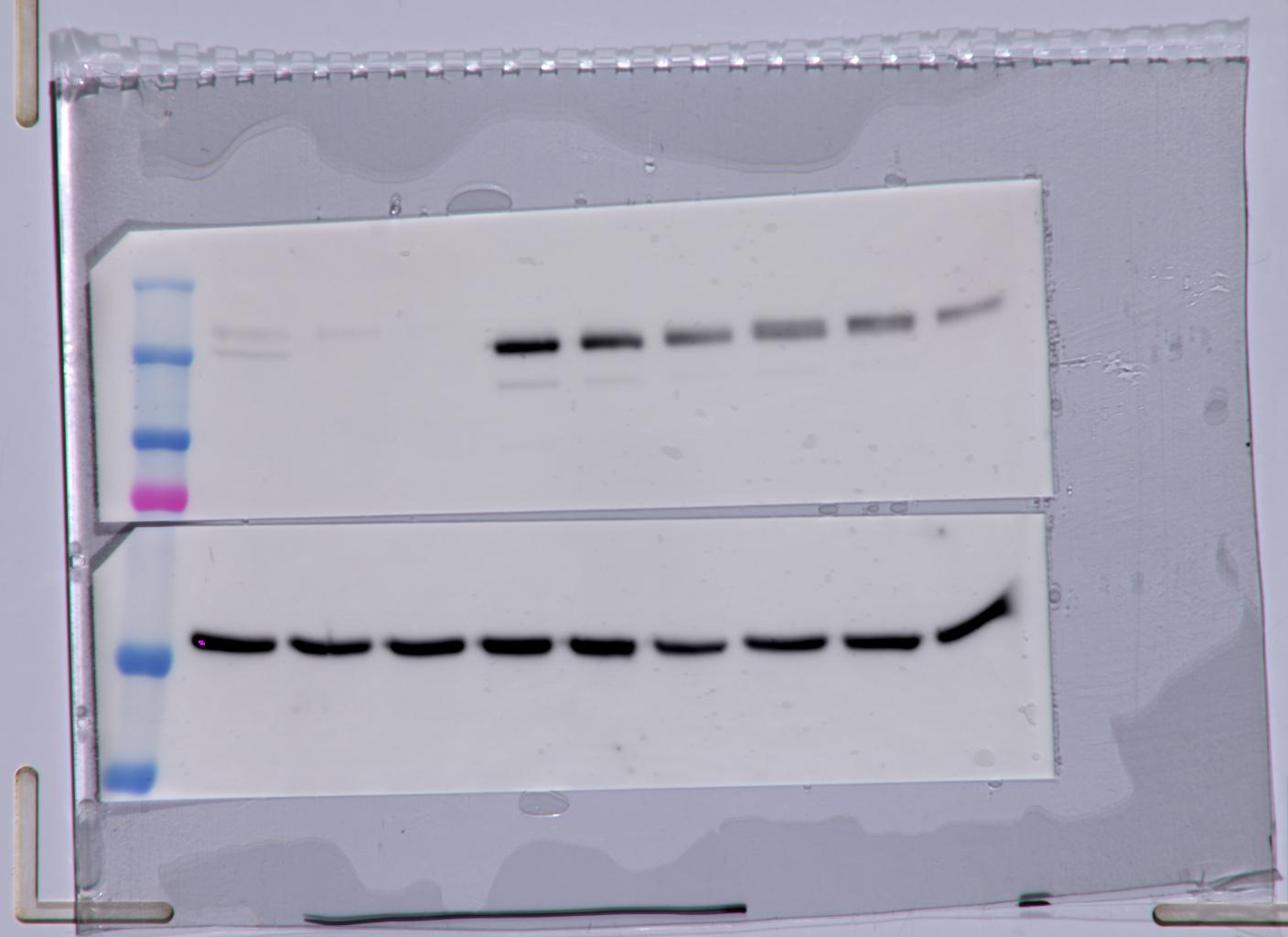

Supplement: Supplementary file 7 — Source data Fig. 4 [file 44318_2025_600_MOESM7_ESM.zip › Figure 4/4C/4C replicates/hek st6 flag.tub 5s 2023.11.21_13.50.49_Ch+Marker.jpg]

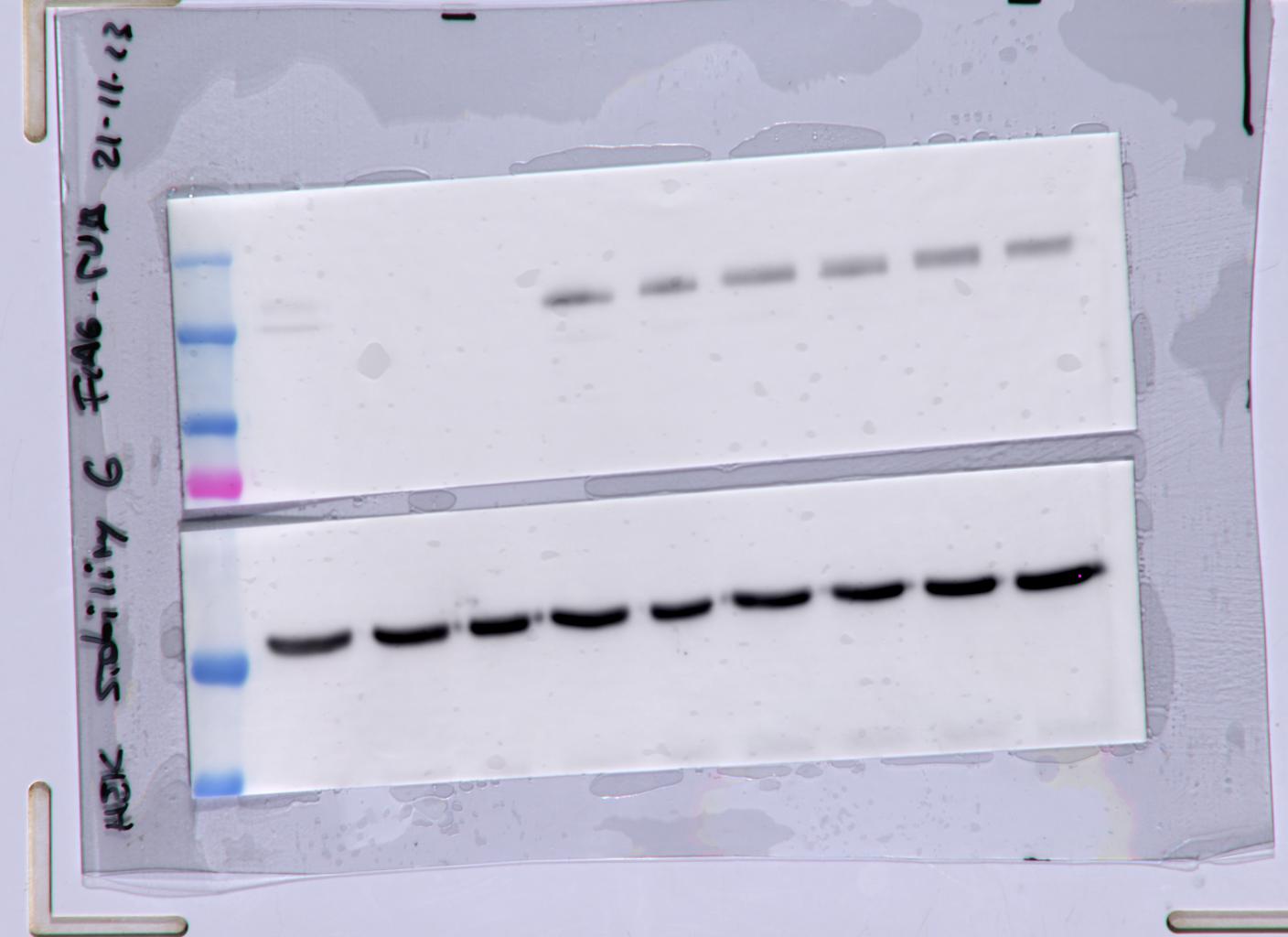

Supplement: Supplementary file 7 — Source data Fig. 4 [file 44318_2025_600_MOESM7_ESM.zip › Figure 4/4C/4C replicates/hek st5 flag.tub 5s 2023.11.21_13.54.30_Ch+Marker.jpg]

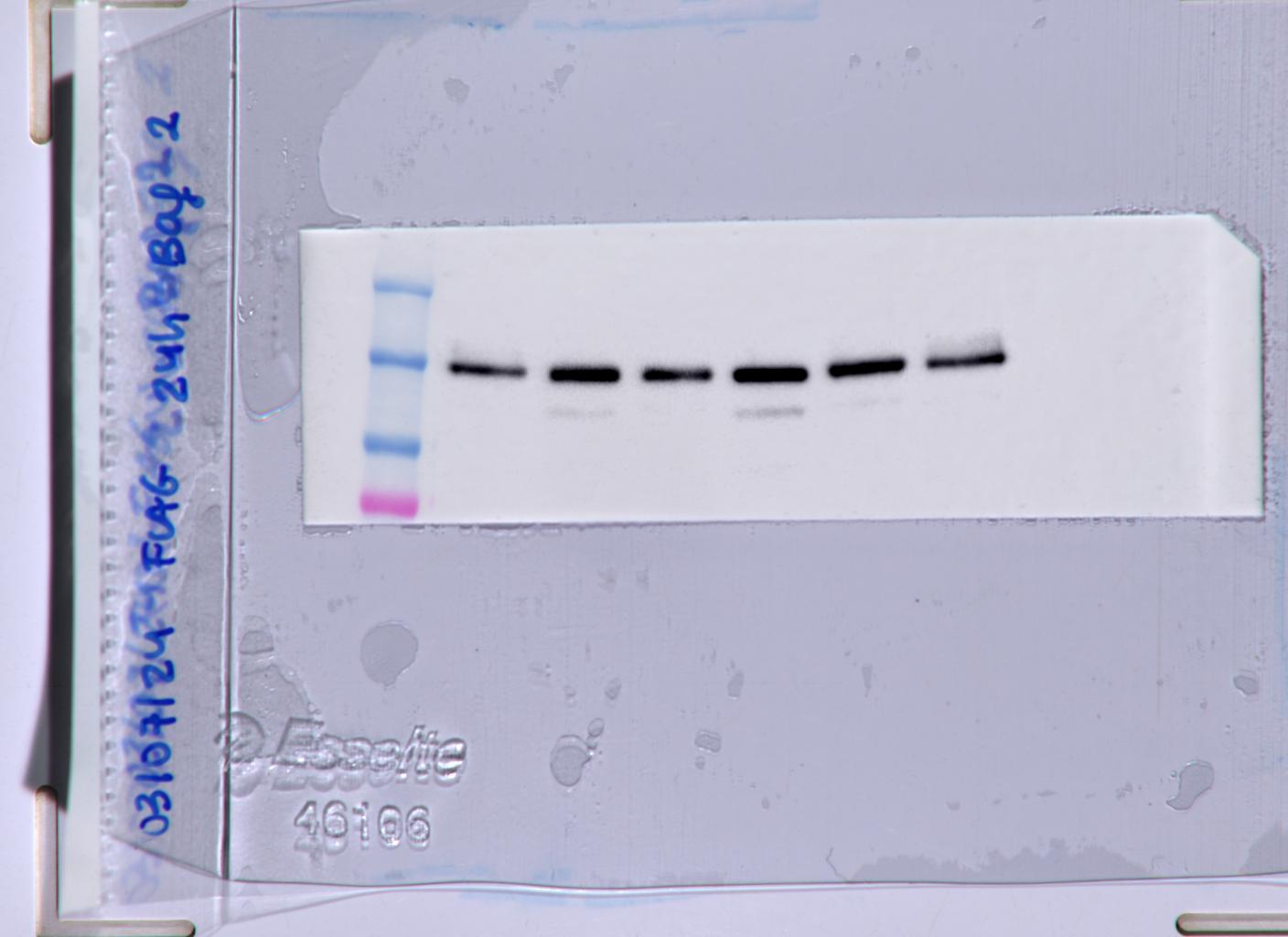

Supplement: Supplementary file 7 — Source data Fig. 4 [file 44318_2025_600_MOESM7_ESM.zip › Figure 4/4A/4A replicates/flag 24hbaf2 1s 2024.07.03_11.05.41_Ch+Marker.jpg]

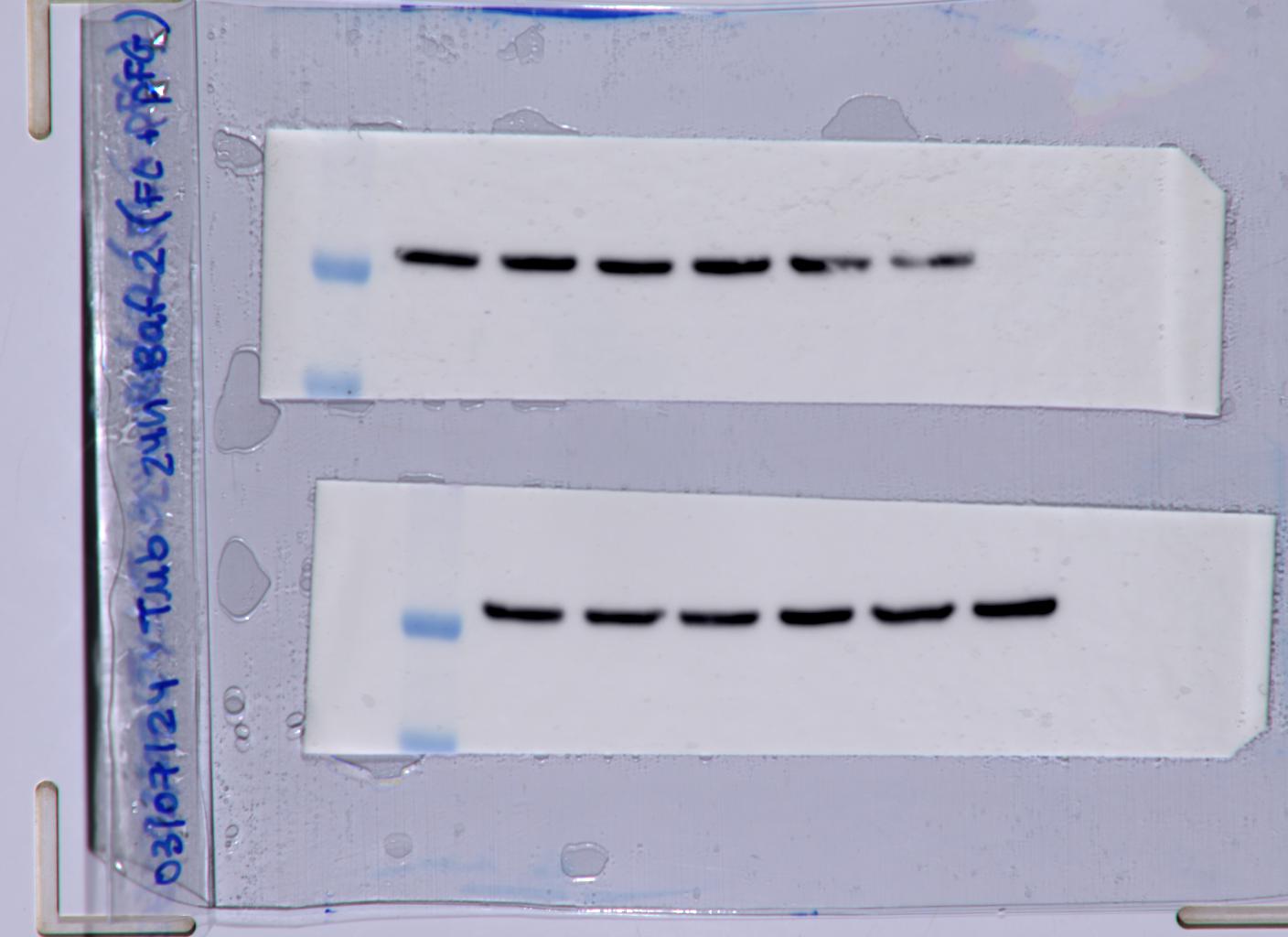

Supplement: Supplementary file 7 — Source data Fig. 4 [file 44318_2025_600_MOESM7_ESM.zip › Figure 4/4A/4A replicates/tub 24hbaf2 30s 2024.07.04_11.26.05_Ch+Marker.jpg]

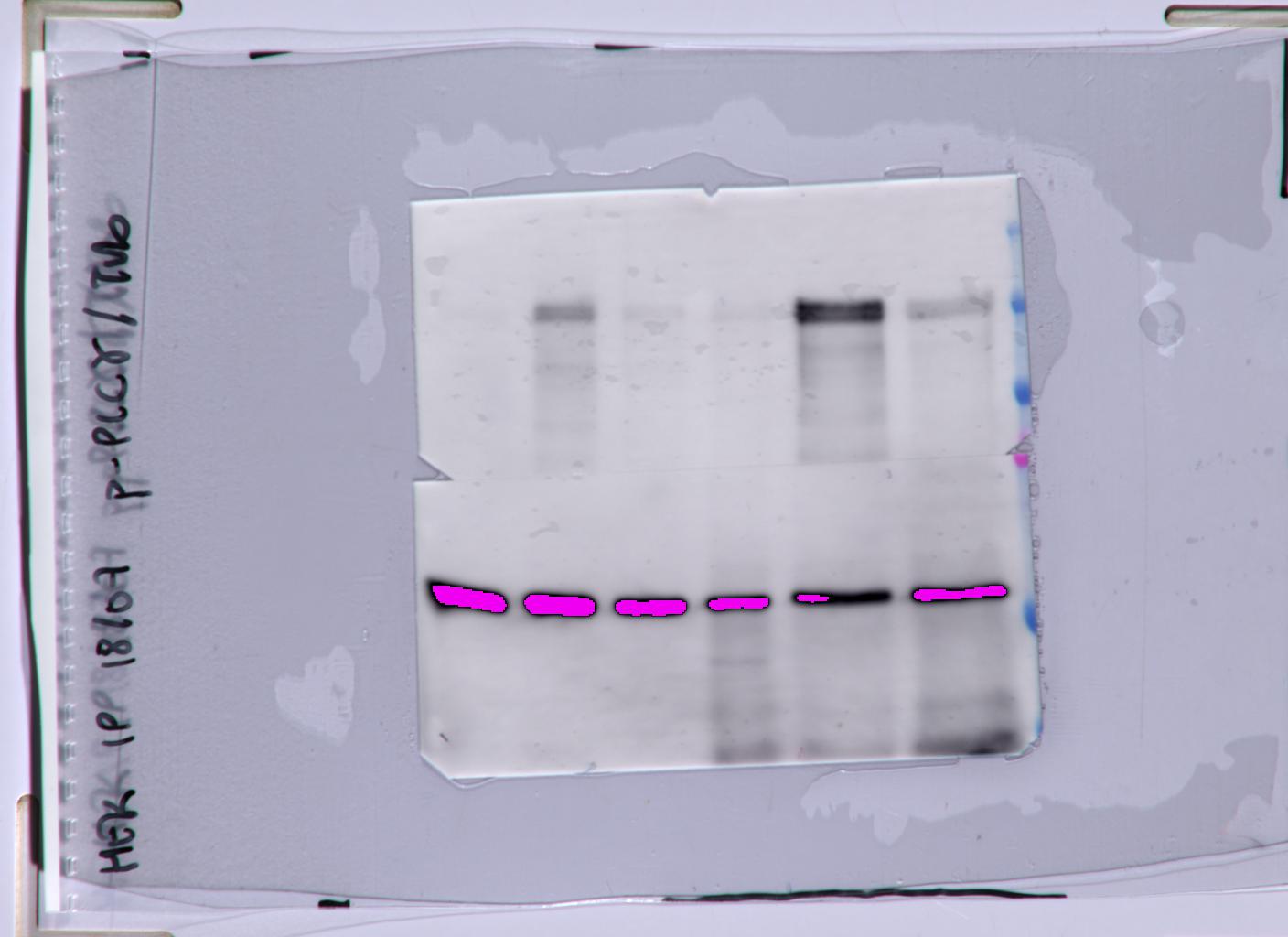

Supplement: Supplementary file 8 — Source data Fig. 5 [file 44318_2025_600_MOESM8_ESM.zip › Figure 5/5C/p-PLCgamma original.jpg]

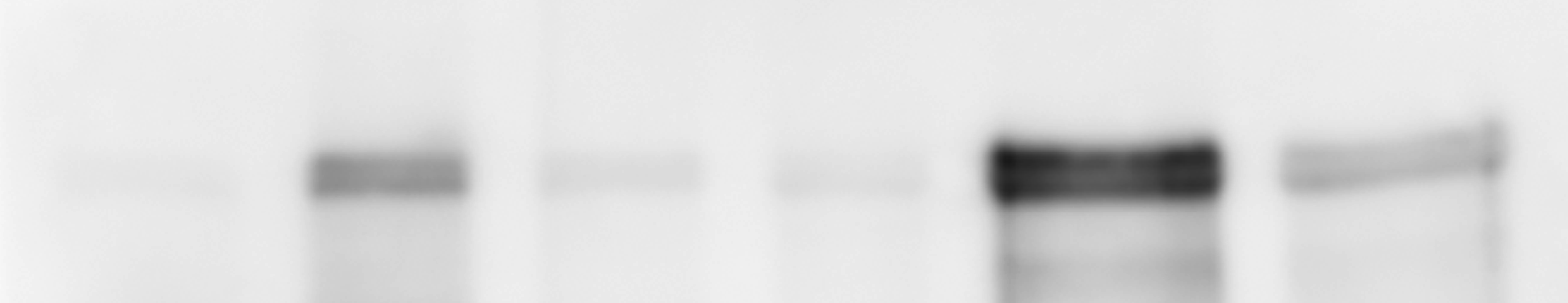

Supplement: Supplementary file 8 — Source data Fig. 5 [file 44318_2025_600_MOESM8_ESM.zip › Figure 5/5C/IP_pPLCg.tif]

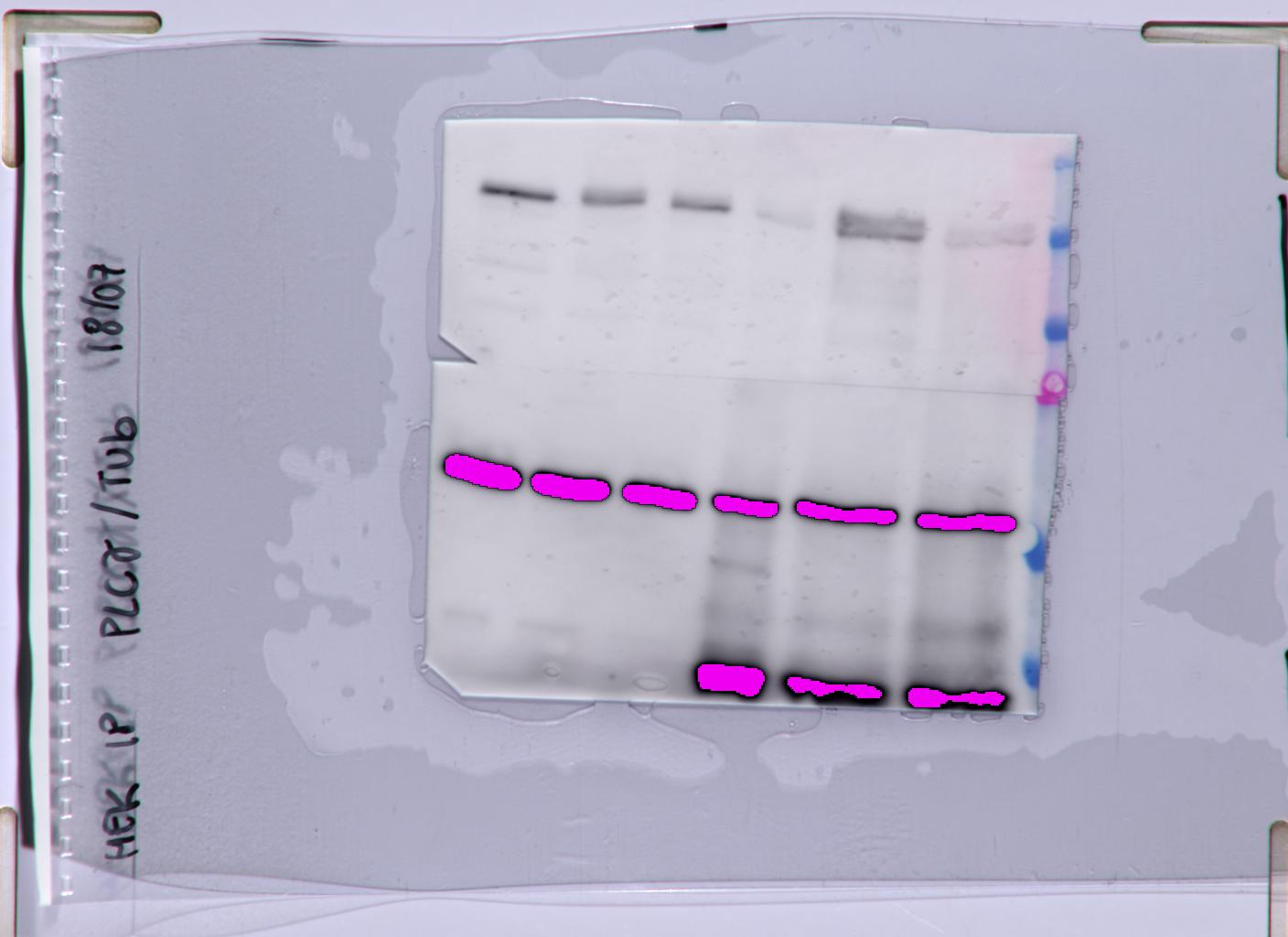

Supplement: Supplementary file 8 — Source data Fig. 5 [file 44318_2025_600_MOESM8_ESM.zip › Figure 5/5C/PLCgamma original.jpg]

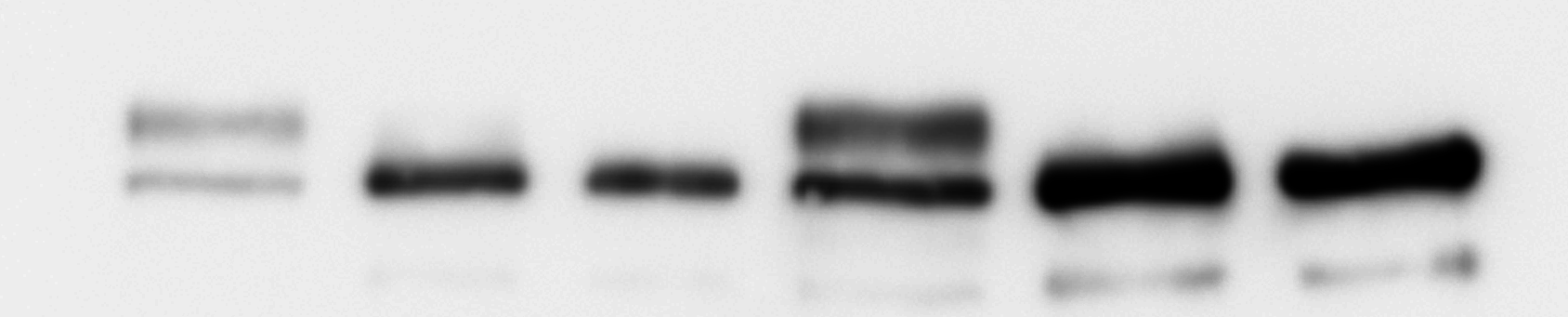

Supplement: Supplementary file 8 — Source data Fig. 5 [file 44318_2025_600_MOESM8_ESM.zip › Figure 5/5C/IP_FLAG.tif]

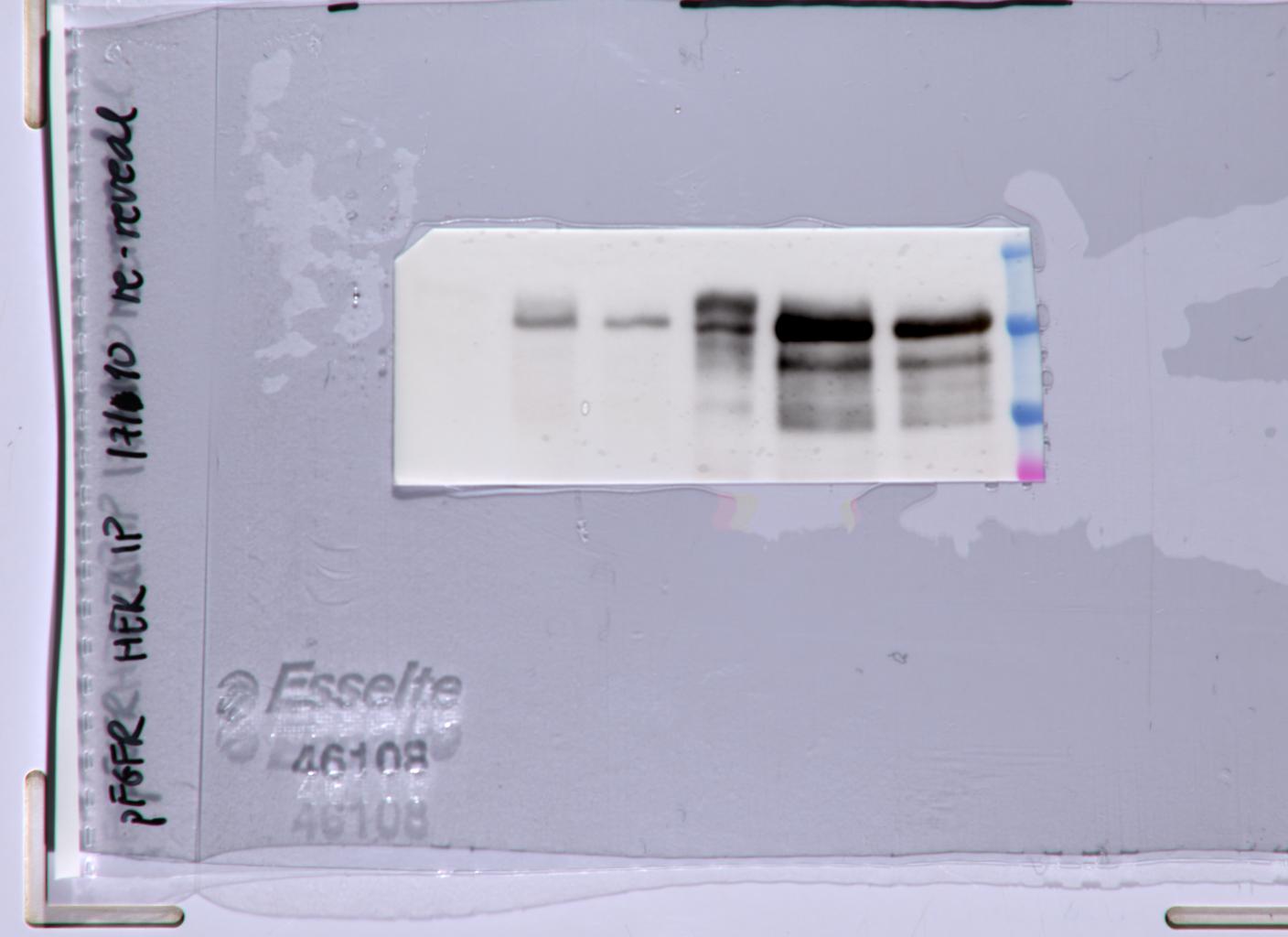

Supplement: Supplementary file 8 — Source data Fig. 5 [file 44318_2025_600_MOESM8_ESM.zip › Figure 5/5C/pFGFR1 original.jpg]

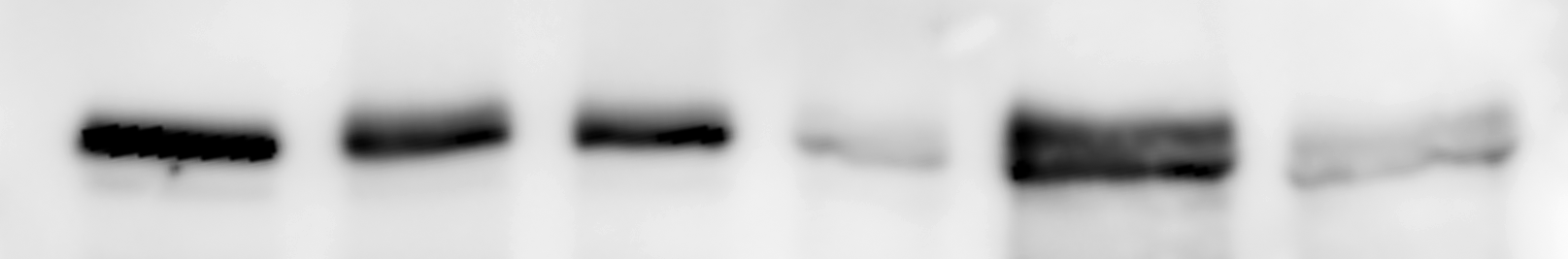

Supplement: Supplementary file 8 — Source data Fig. 5 [file 44318_2025_600_MOESM8_ESM.zip › Figure 5/5C/IP_PLCg.tif]

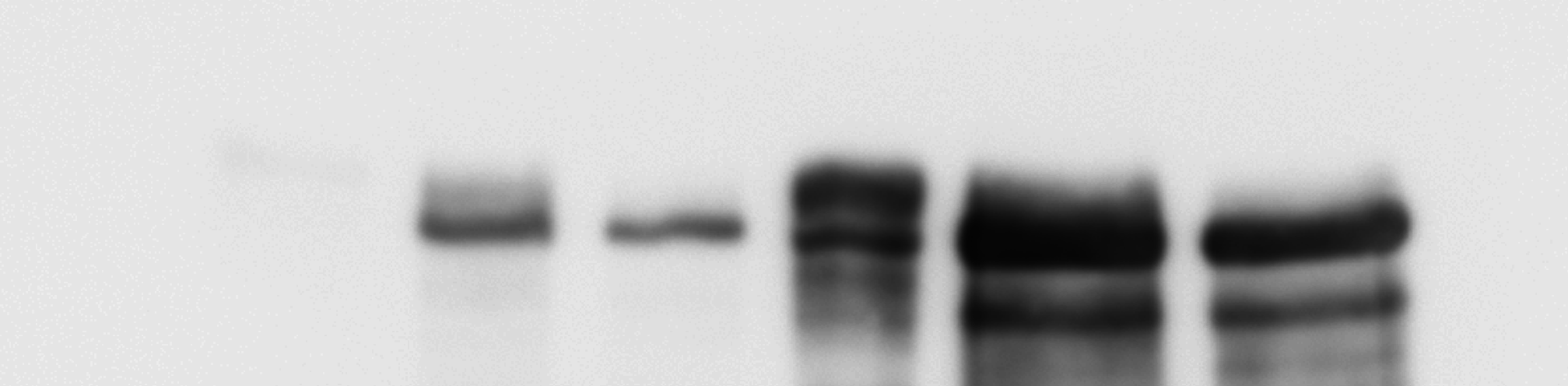

Supplement: Supplementary file 8 — Source data Fig. 5 [file 44318_2025_600_MOESM8_ESM.zip › Figure 5/5C/IP_pFGFR1.tif]

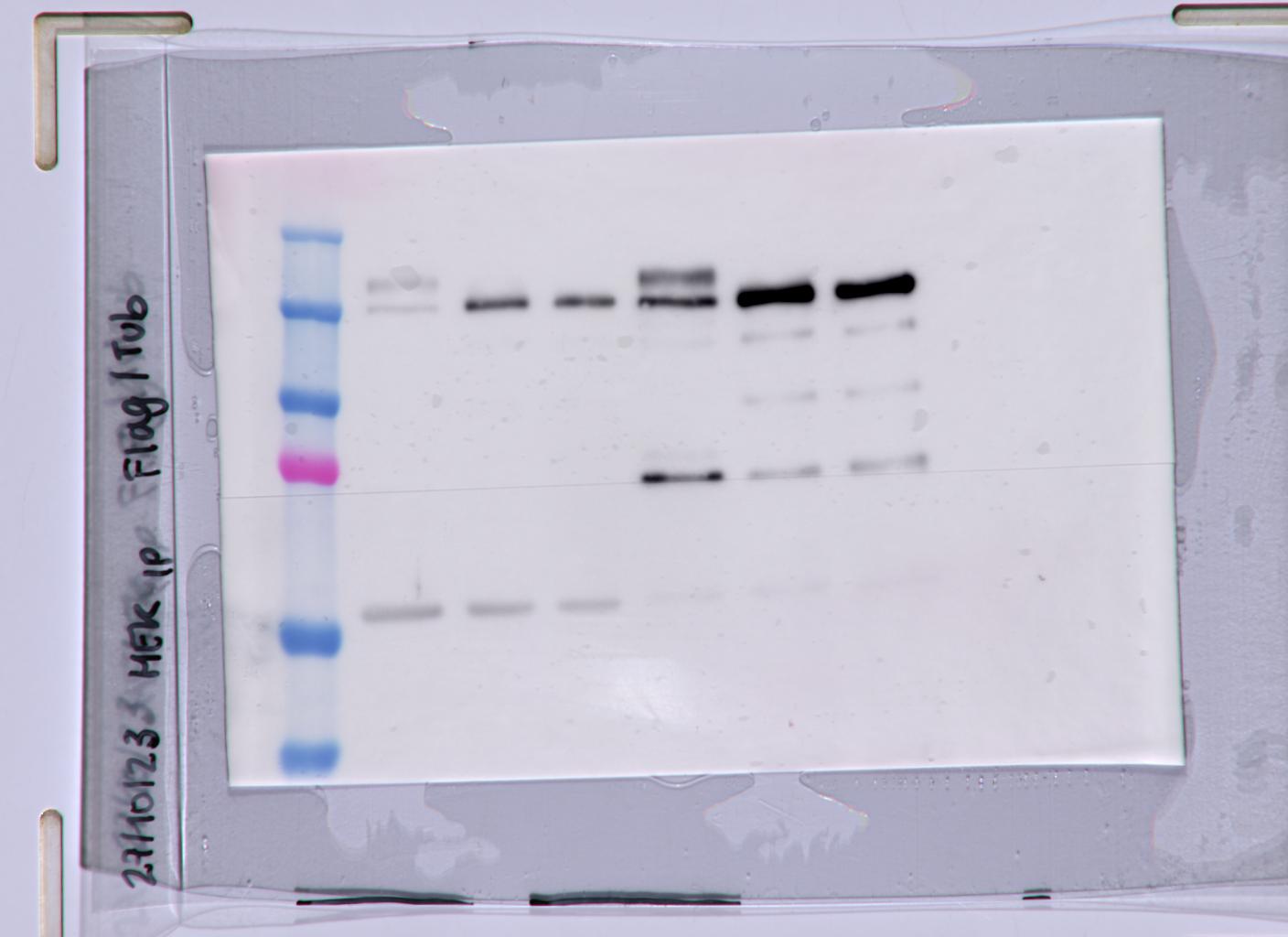

Supplement: Supplementary file 8 — Source data Fig. 5 [file 44318_2025_600_MOESM8_ESM.zip › Figure 5/5C/flag original.jpg]

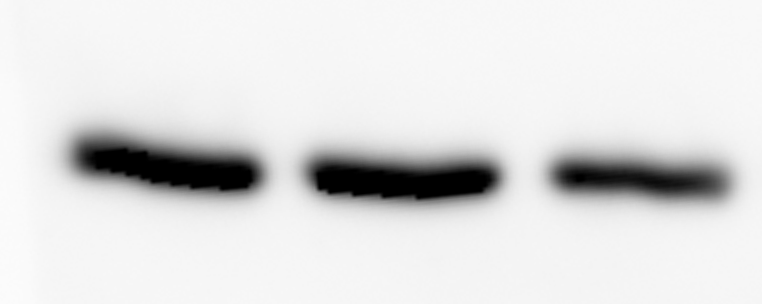

Supplement: Supplementary file 8 — Source data Fig. 5 [file 44318_2025_600_MOESM8_ESM.zip › Figure 5/5C/IP_Tub.tif]

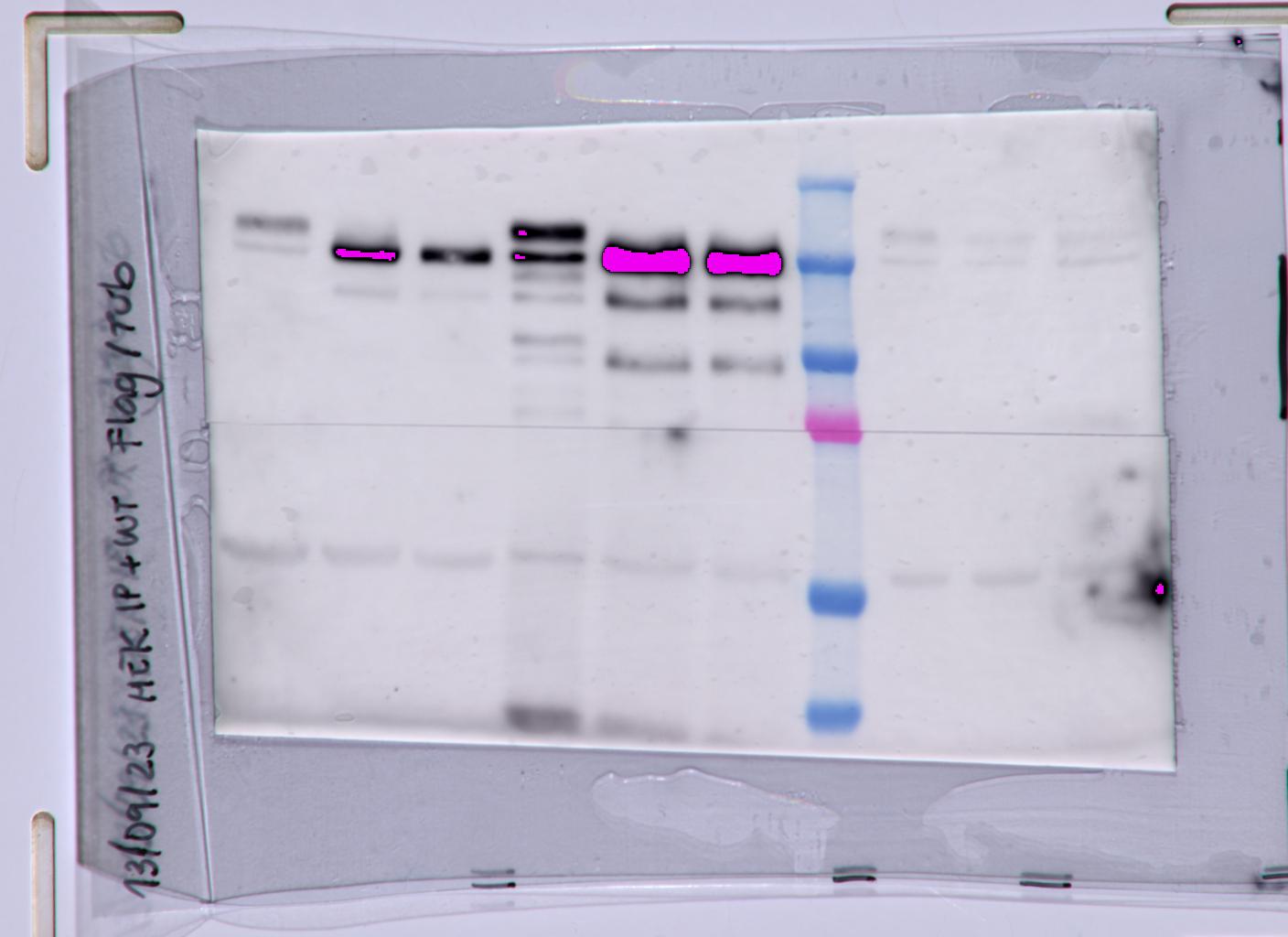

Supplement: Supplementary file 8 — Source data Fig. 5 [file 44318_2025_600_MOESM8_ESM.zip › Figure 5/5C/5C replicates/hek ip wt flagtub 5s 2023.09.13_11.19.59_Ch+Marker.jpg]

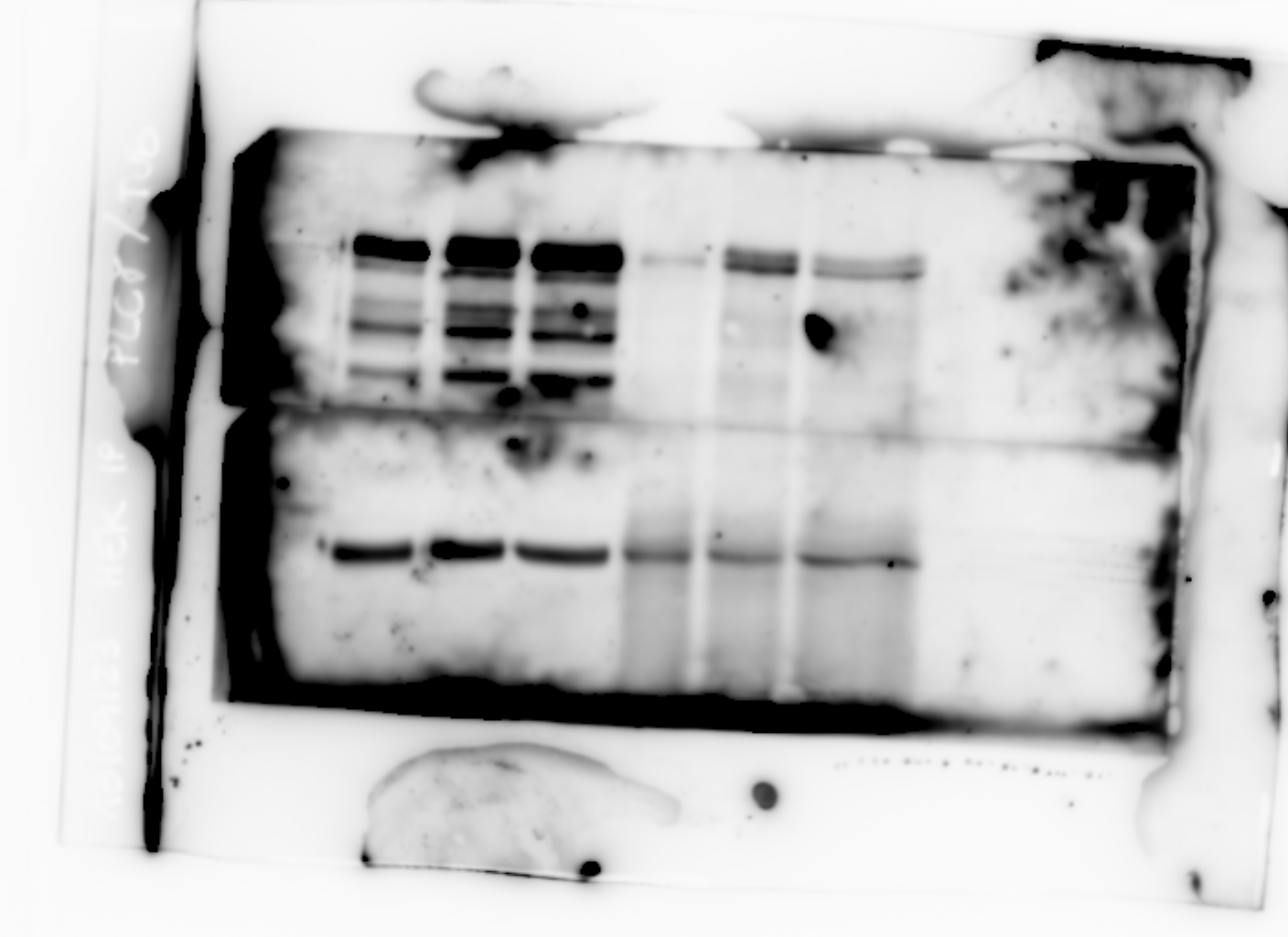

Supplement: Supplementary file 8 — Source data Fig. 5 [file 44318_2025_600_MOESM8_ESM.zip › Figure 5/5C/5C replicates/hek ip plctub 1m 2023.09.13_11.30.55_Ch.tif]

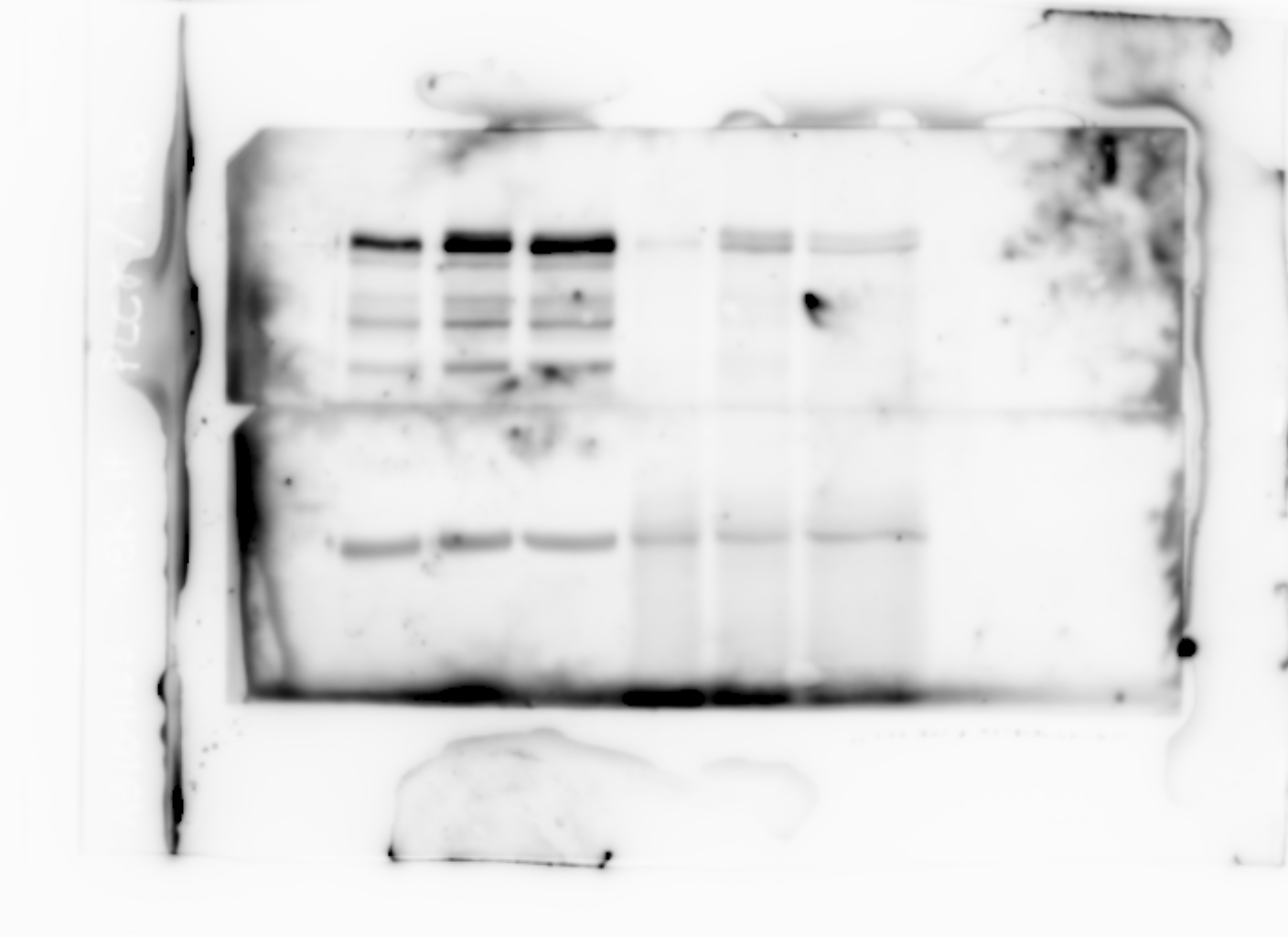

Supplement: Supplementary file 8 — Source data Fig. 5 [file 44318_2025_600_MOESM8_ESM.zip › Figure 5/5C/5C replicates/hek ip plctub 20s 2023.09.13_11.26.27_Ch.tif]

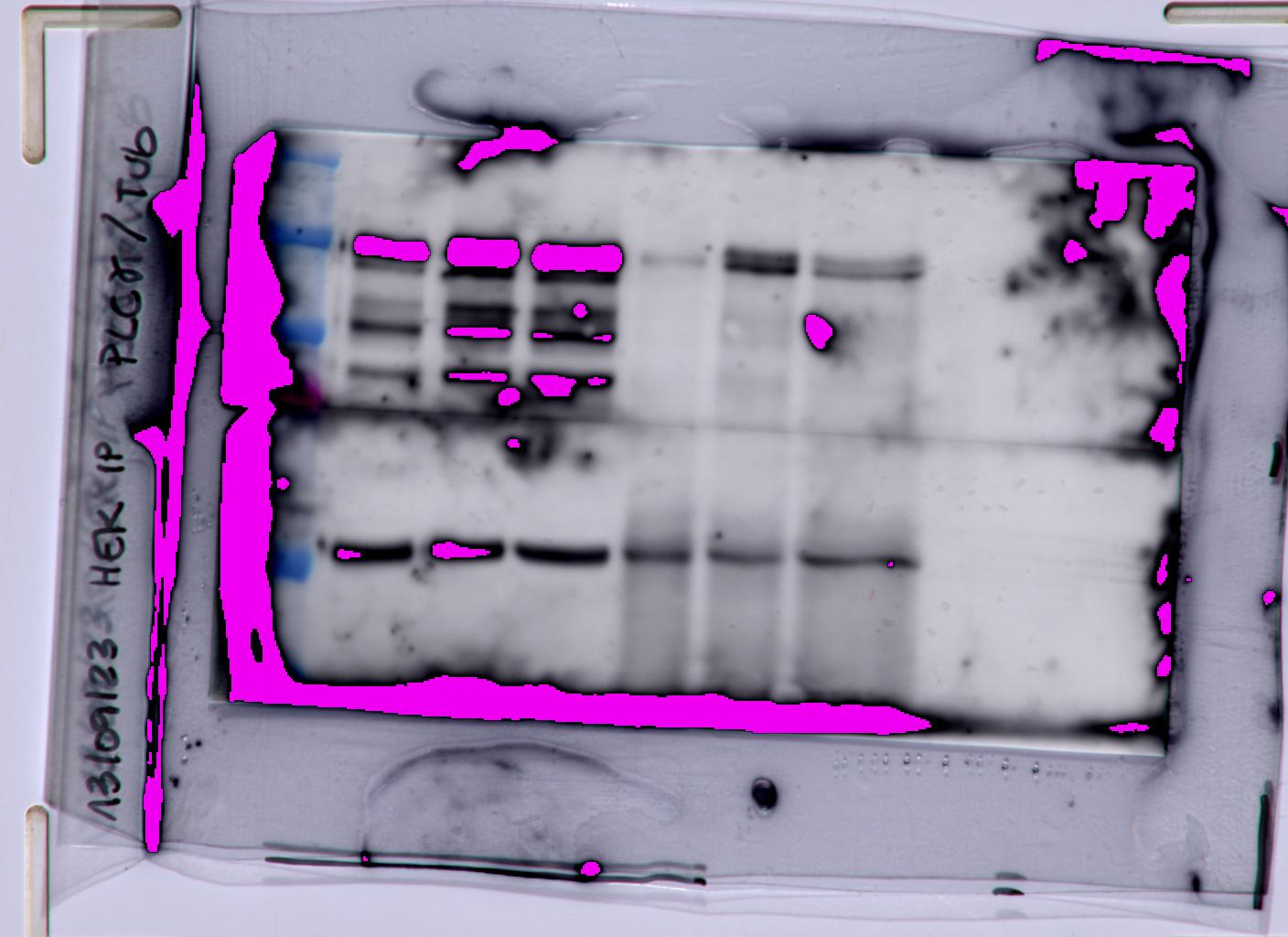

Supplement: Supplementary file 8 — Source data Fig. 5 [file 44318_2025_600_MOESM8_ESM.zip › Figure 5/5C/5C replicates/hek ip plctub 1m 2023.09.13_11.30.55_Ch+Marker.jpg]

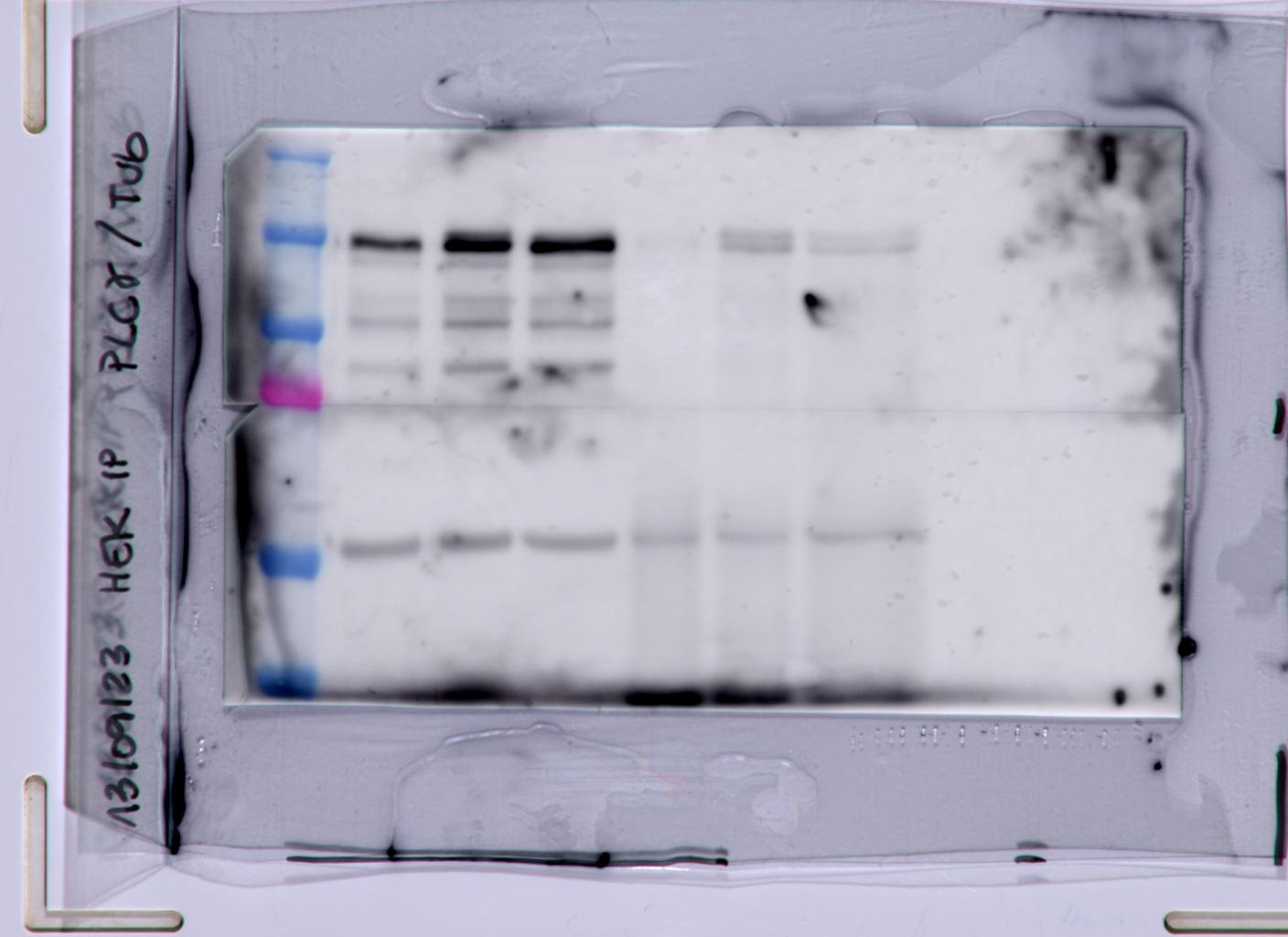

Supplement: Supplementary file 8 — Source data Fig. 5 [file 44318_2025_600_MOESM8_ESM.zip › Figure 5/5C/5C replicates/hek ip plctub 5s 2023.09.13_11.24.53_Ch+Marker.jpg]

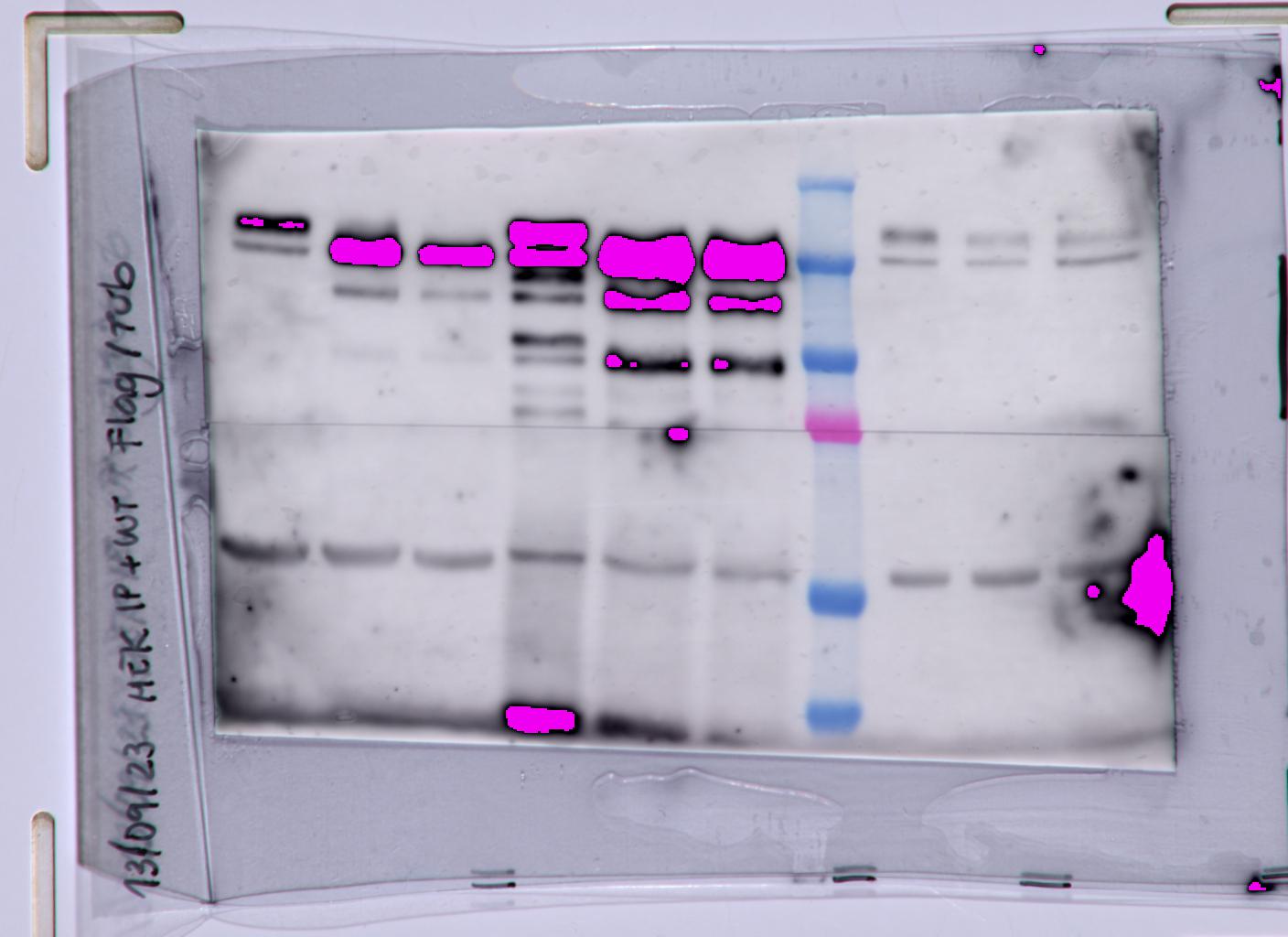

Supplement: Supplementary file 8 — Source data Fig. 5 [file 44318_2025_600_MOESM8_ESM.zip › Figure 5/5C/5C replicates/hek ipwt flagtub 20s 2023.09.13_11.22.14_Ch+Marker.jpg]

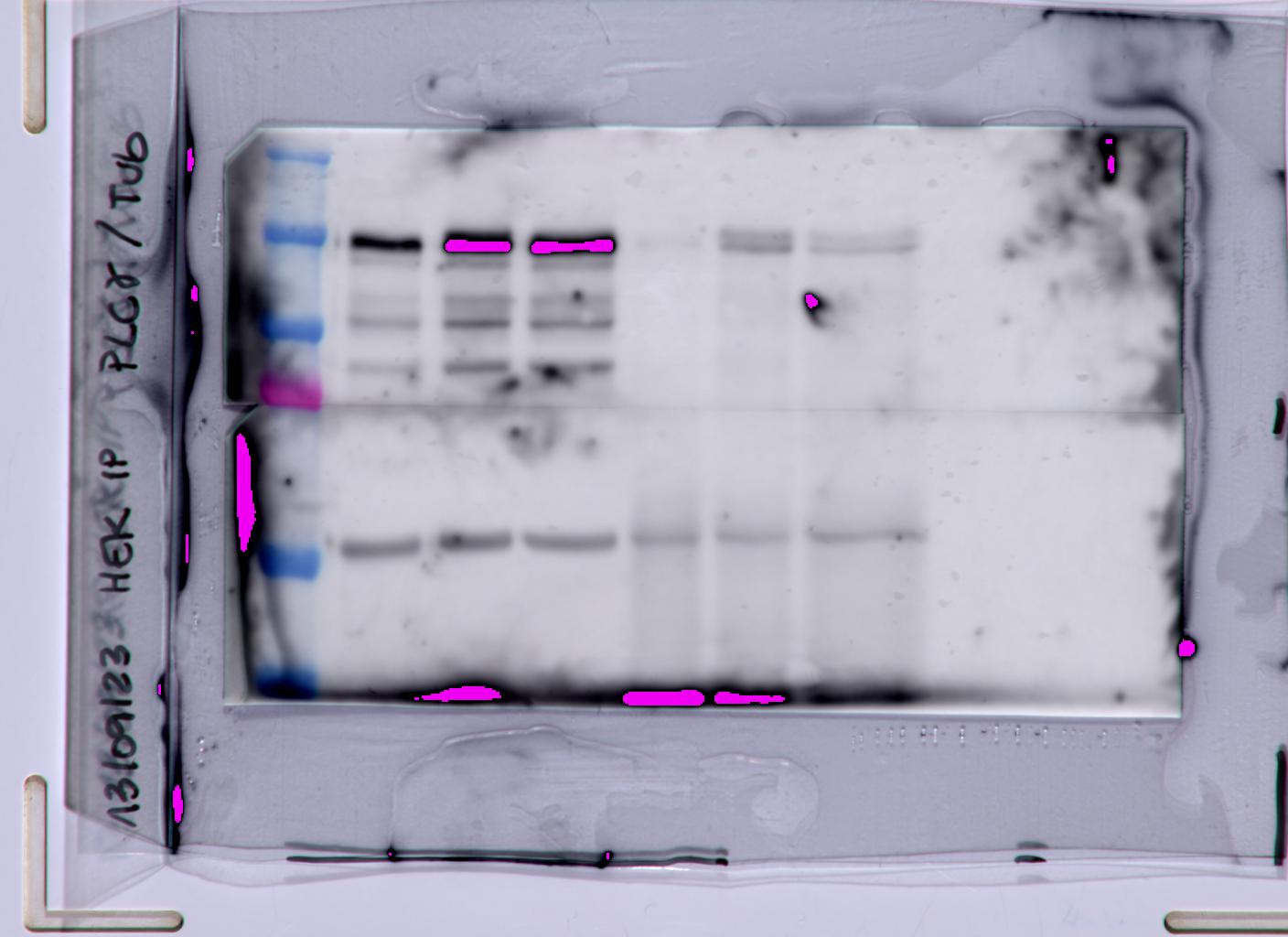

Supplement: Supplementary file 8 — Source data Fig. 5 [file 44318_2025_600_MOESM8_ESM.zip › Figure 5/5C/5C replicates/hek ip plctub 20s 2023.09.13_11.26.27_Ch+Marker.jpg]

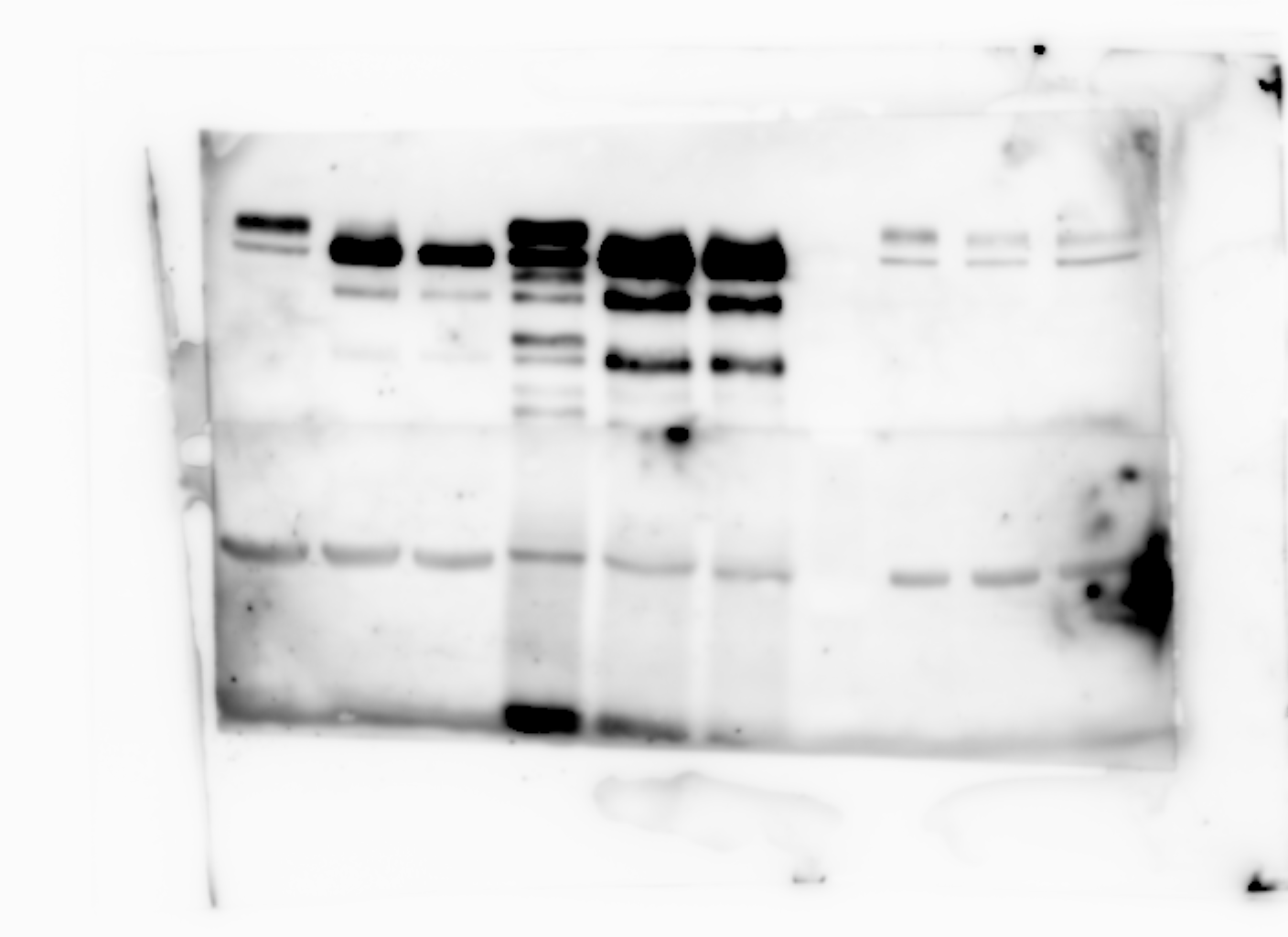

Supplement: Supplementary file 8 — Source data Fig. 5 [file 44318_2025_600_MOESM8_ESM.zip › Figure 5/5C/5C replicates/hek ipwt flagtub 20s 2023.09.13_11.22.14_Ch.tif]

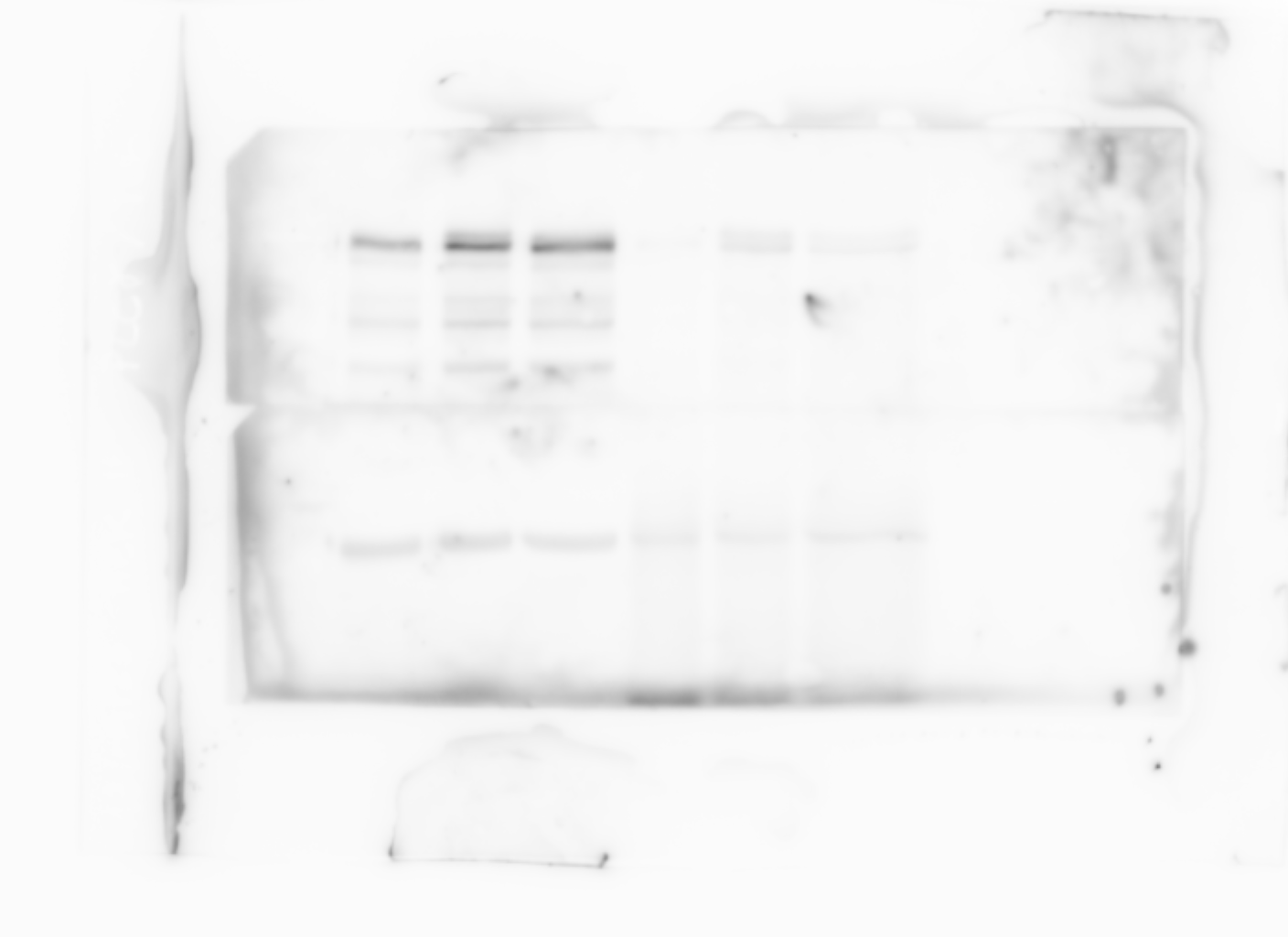

Supplement: Supplementary file 8 — Source data Fig. 5 [file 44318_2025_600_MOESM8_ESM.zip › Figure 5/5C/5C replicates/hek ip plctub 5s 2023.09.13_11.24.53_Ch.tif]

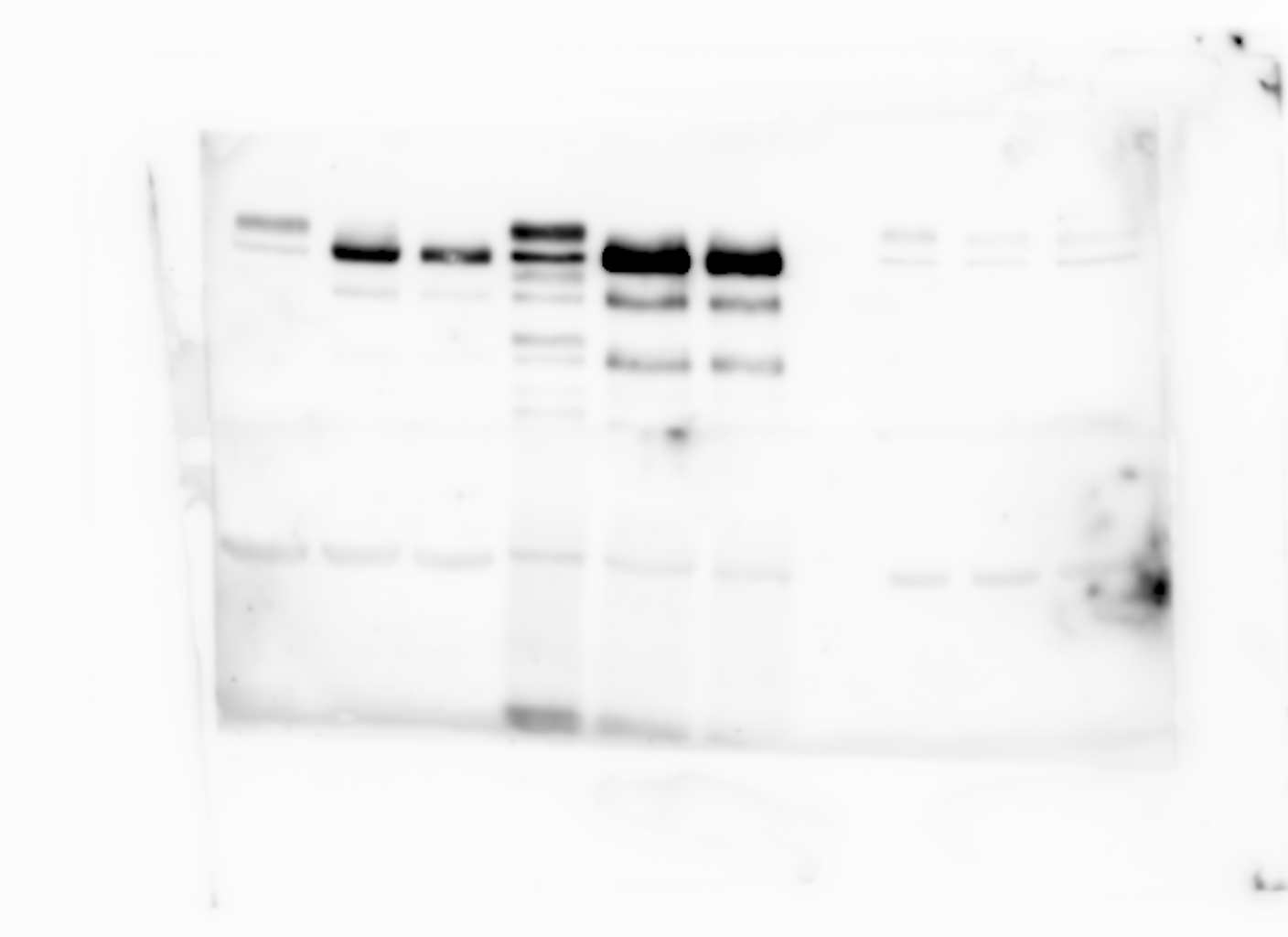

Supplement: Supplementary file 8 — Source data Fig. 5 [file 44318_2025_600_MOESM8_ESM.zip › Figure 5/5C/5C replicates/hek ip wt flagtub 5s 2023.09.13_11.19.59_Ch.tif]

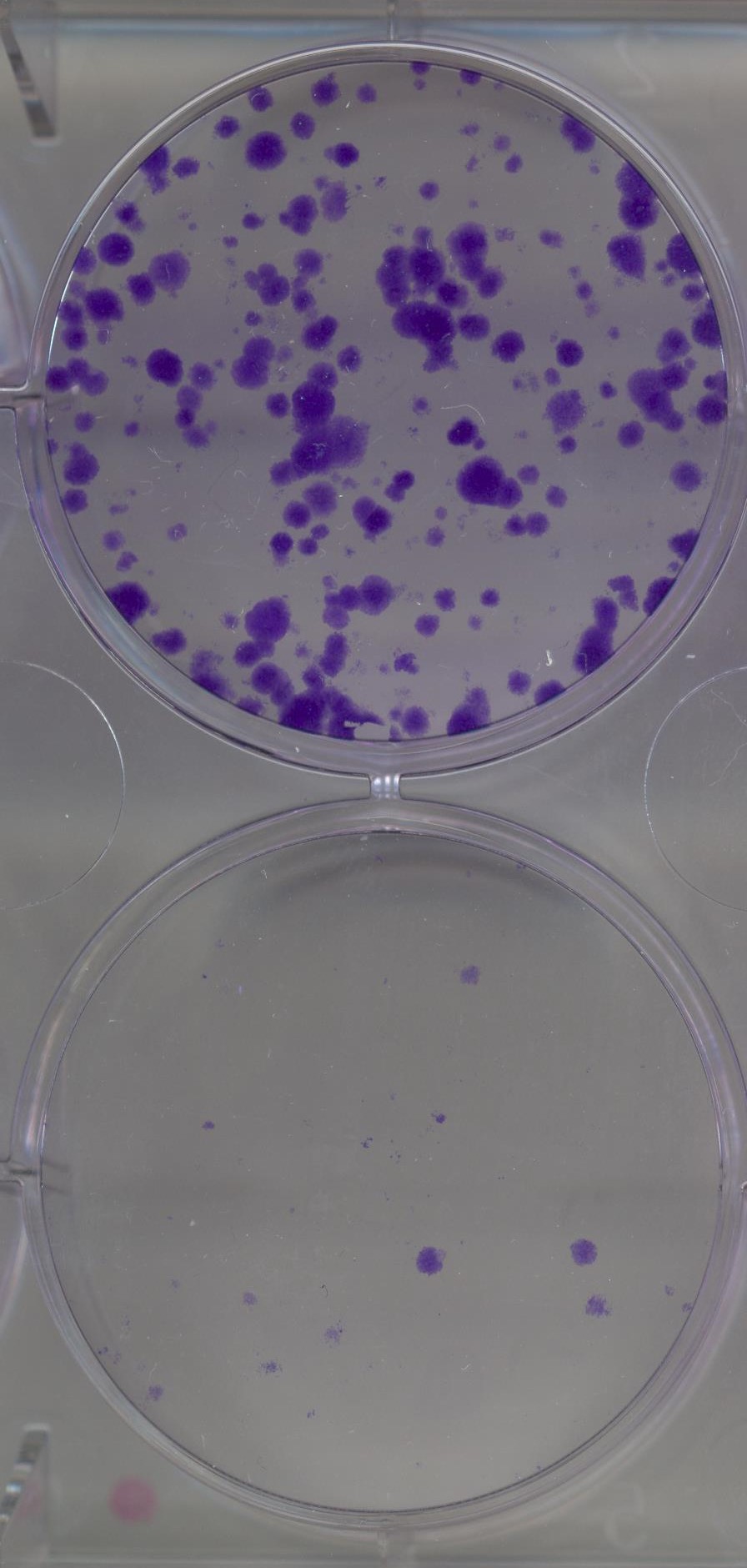

Supplement: Supplementary file 9 — Source data Fig. 6 [file 44318_2025_600_MOESM9_ESM.zip › Figure 6/6B/P-R2.jpg]

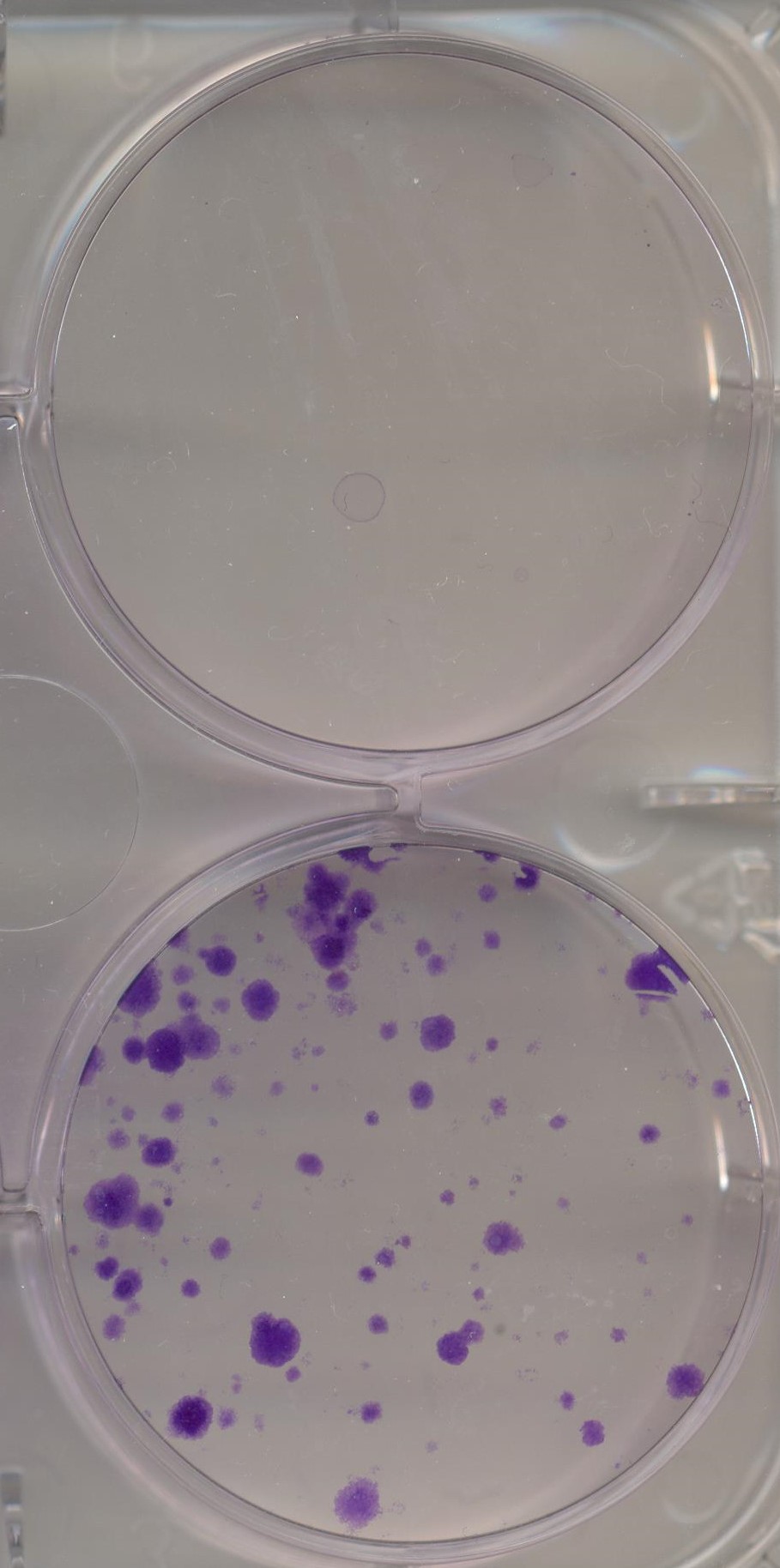

Supplement: Supplementary file 9 — Source data Fig. 6 [file 44318_2025_600_MOESM9_ESM.zip › Figure 6/6B/P-R1.jpg]

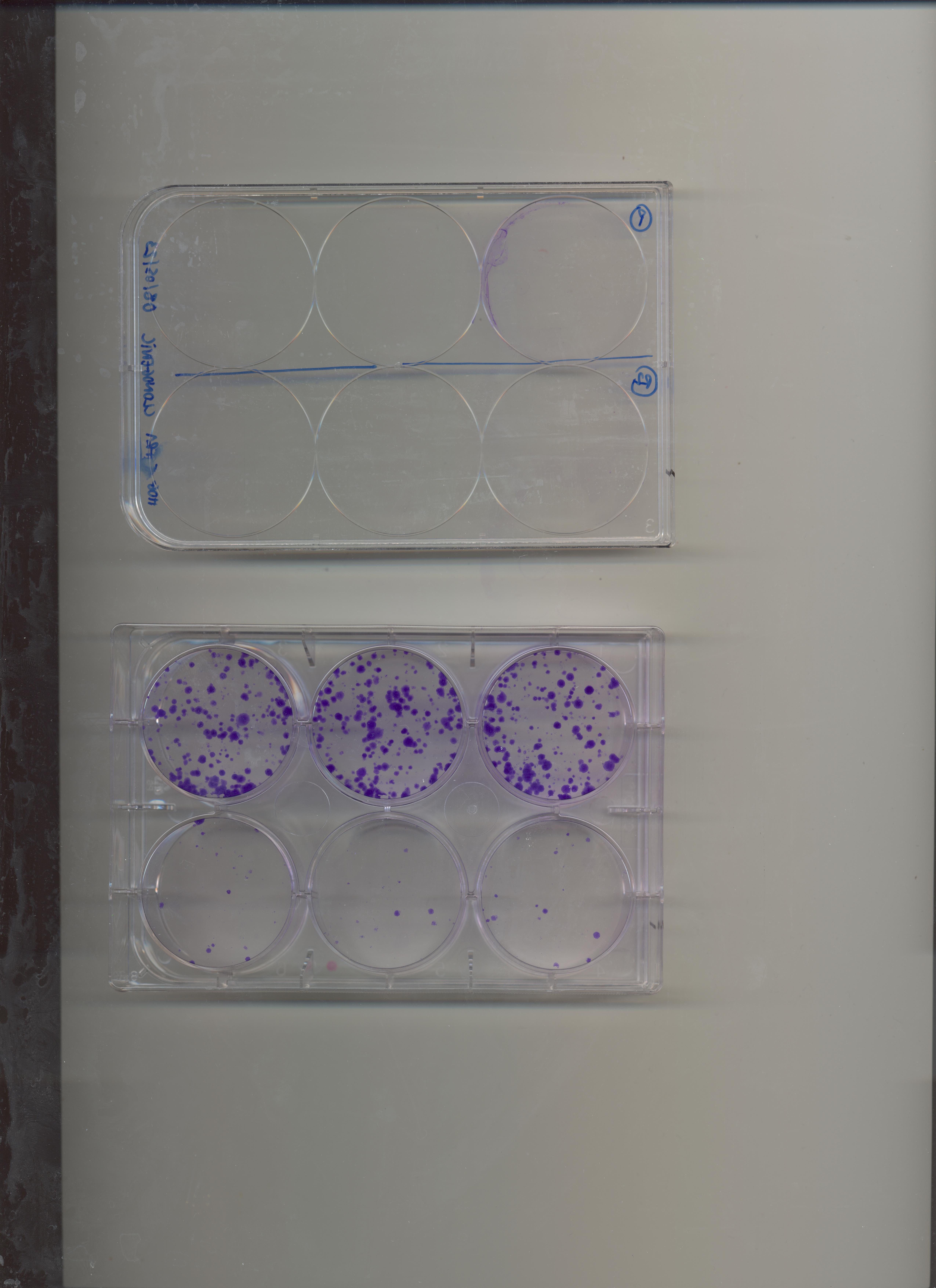

Supplement: Supplementary file 9 — Source data Fig. 6 [file 44318_2025_600_MOESM9_ESM.zip › Figure 6/6B/080523_P-R2 001.jpg]

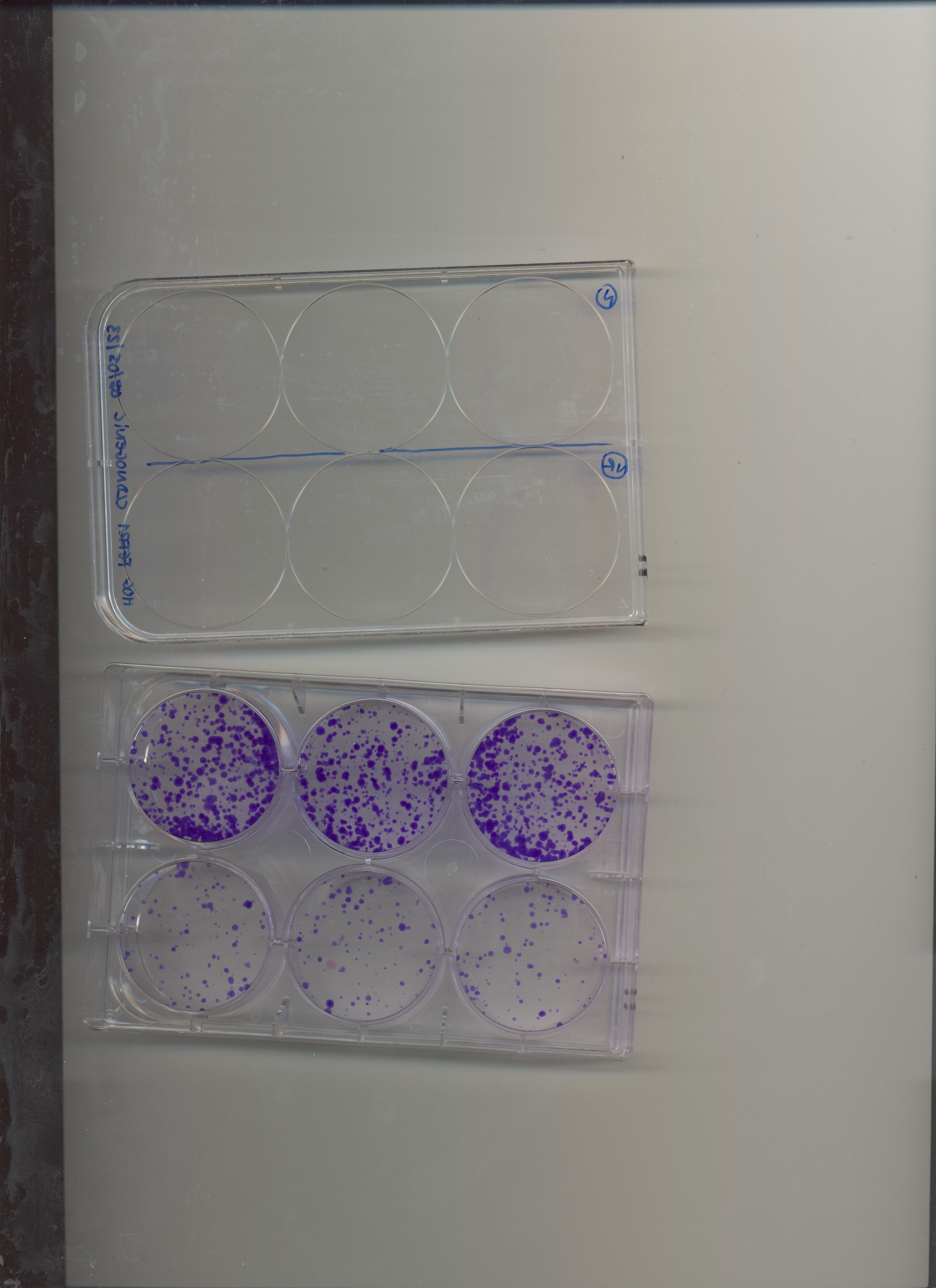

Supplement: Supplementary file 9 — Source data Fig. 6 [file 44318_2025_600_MOESM9_ESM.zip › Figure 6/6B/080523_N-NR 001.jpg]

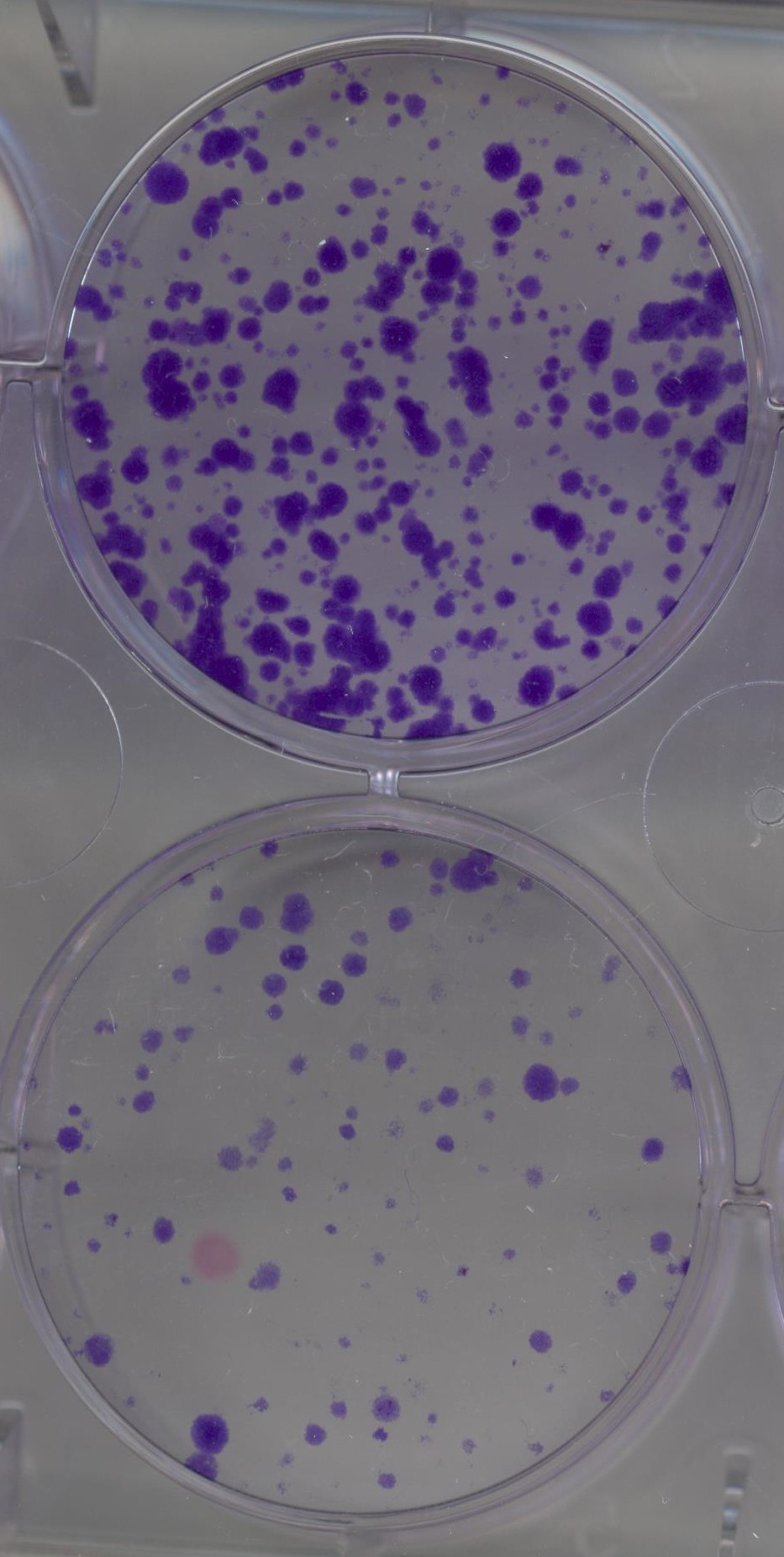

Supplement: Supplementary file 9 — Source data Fig. 6 [file 44318_2025_600_MOESM9_ESM.zip › Figure 6/6B/N1-NR1.jpg]

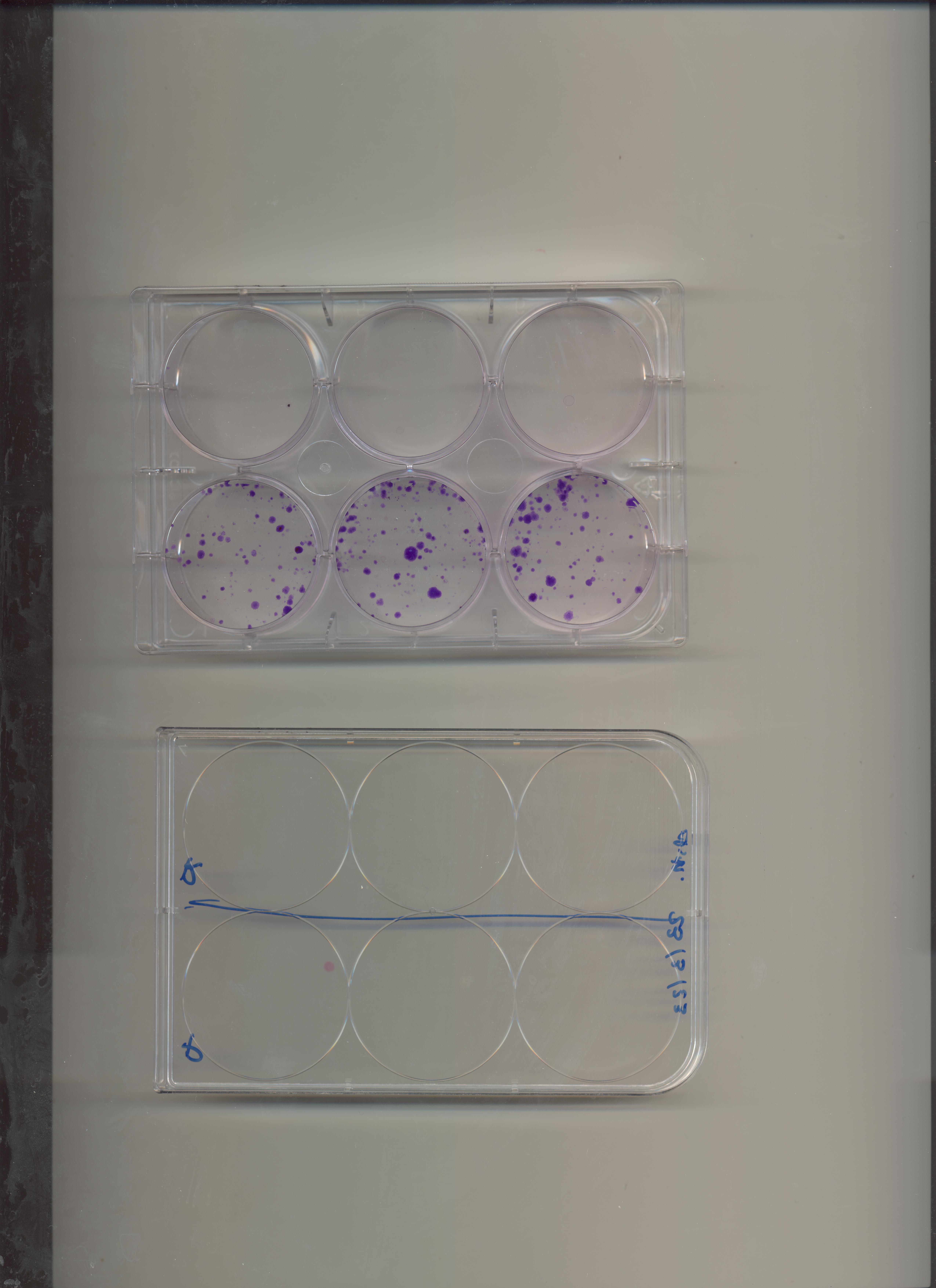

Supplement: Supplementary file 9 — Source data Fig. 6 [file 44318_2025_600_MOESM9_ESM.zip › Figure 6/6B/230323_P-R 001.jpg]

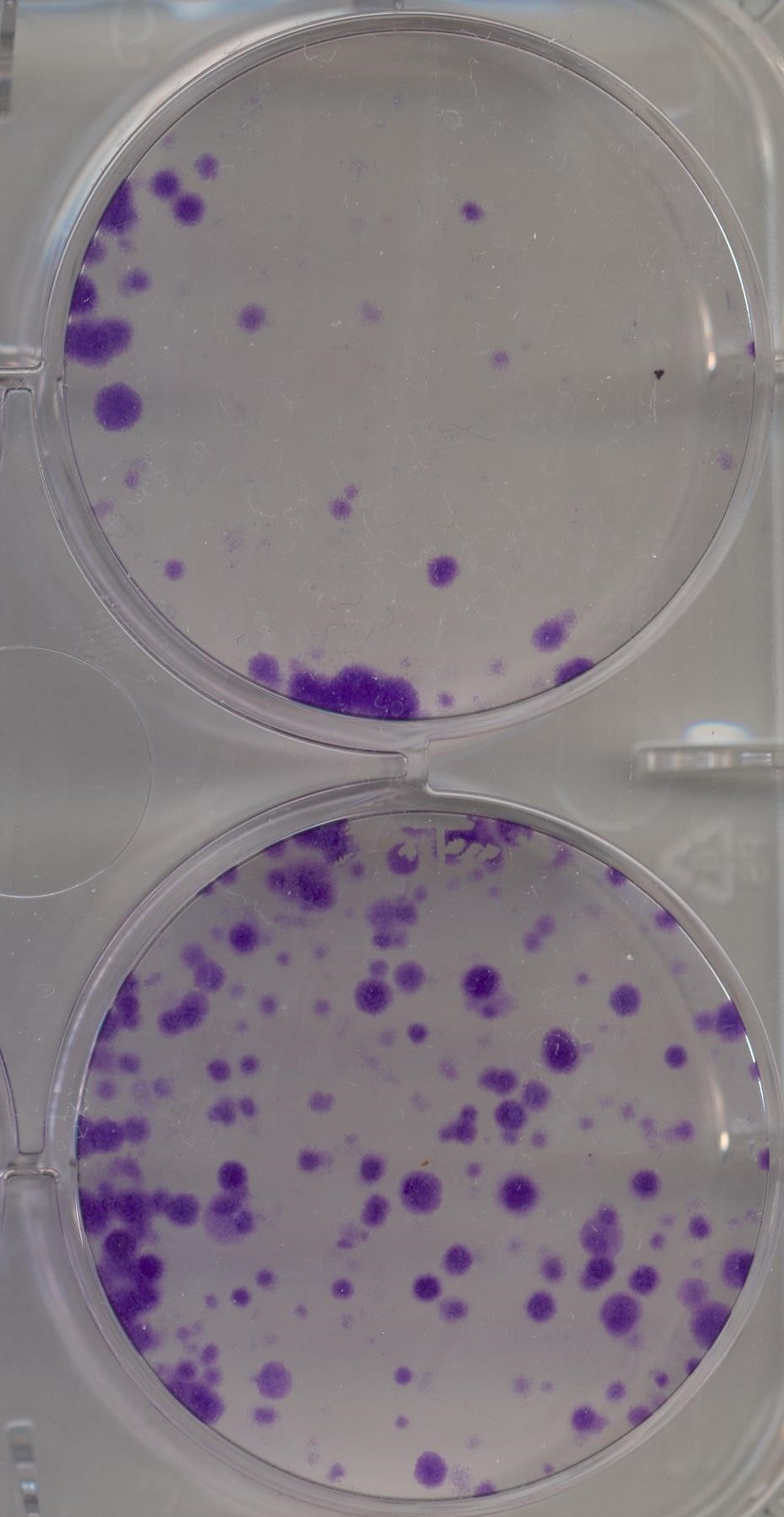

Supplement: Supplementary file 9 — Source data Fig. 6 [file 44318_2025_600_MOESM9_ESM.zip › Figure 6/6B/K1-KR1.jpg]

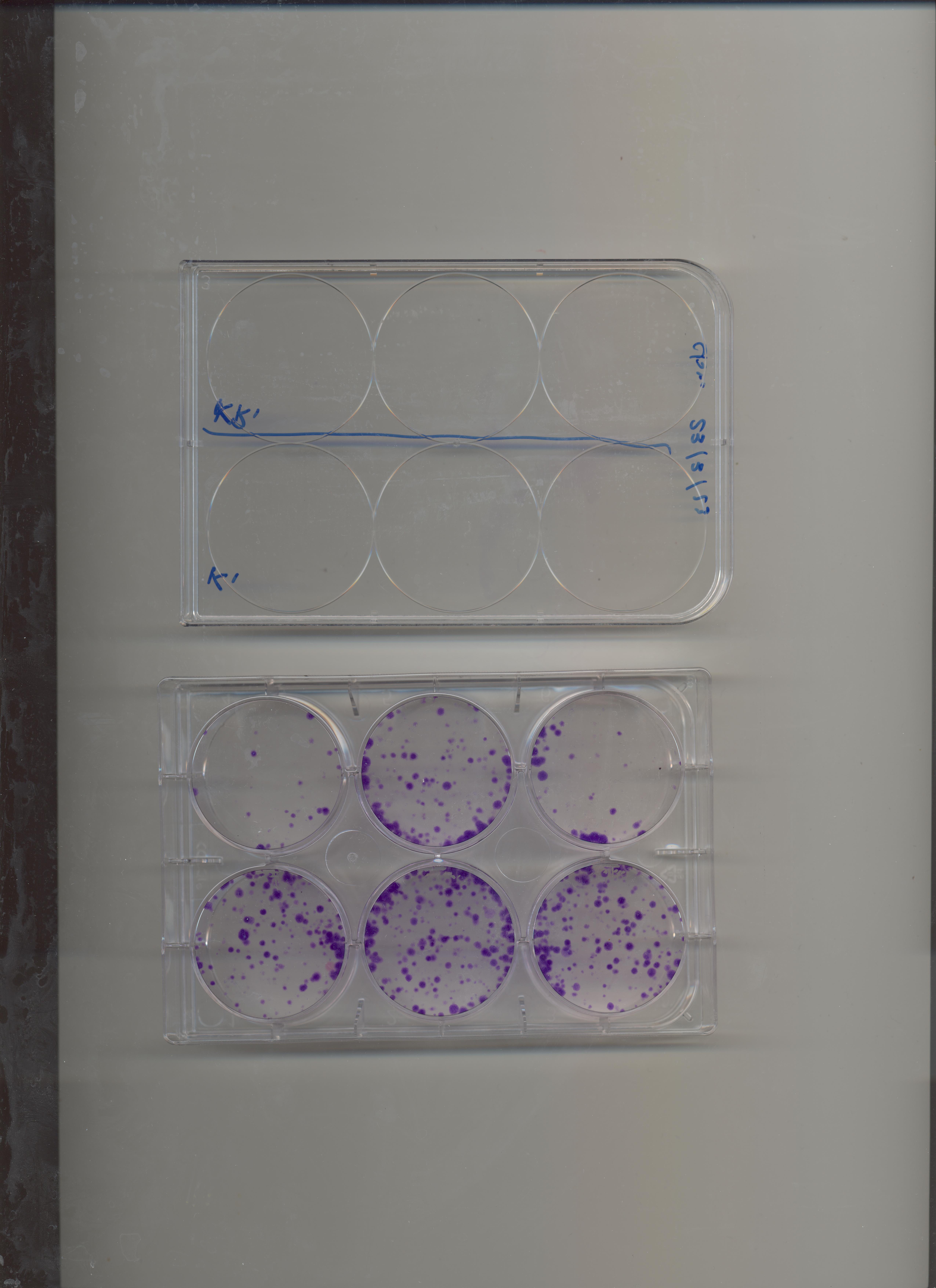

Supplement: Supplementary file 9 — Source data Fig. 6 [file 44318_2025_600_MOESM9_ESM.zip › Figure 6/6B/230323_K1-KR1 001 (1).jpg]

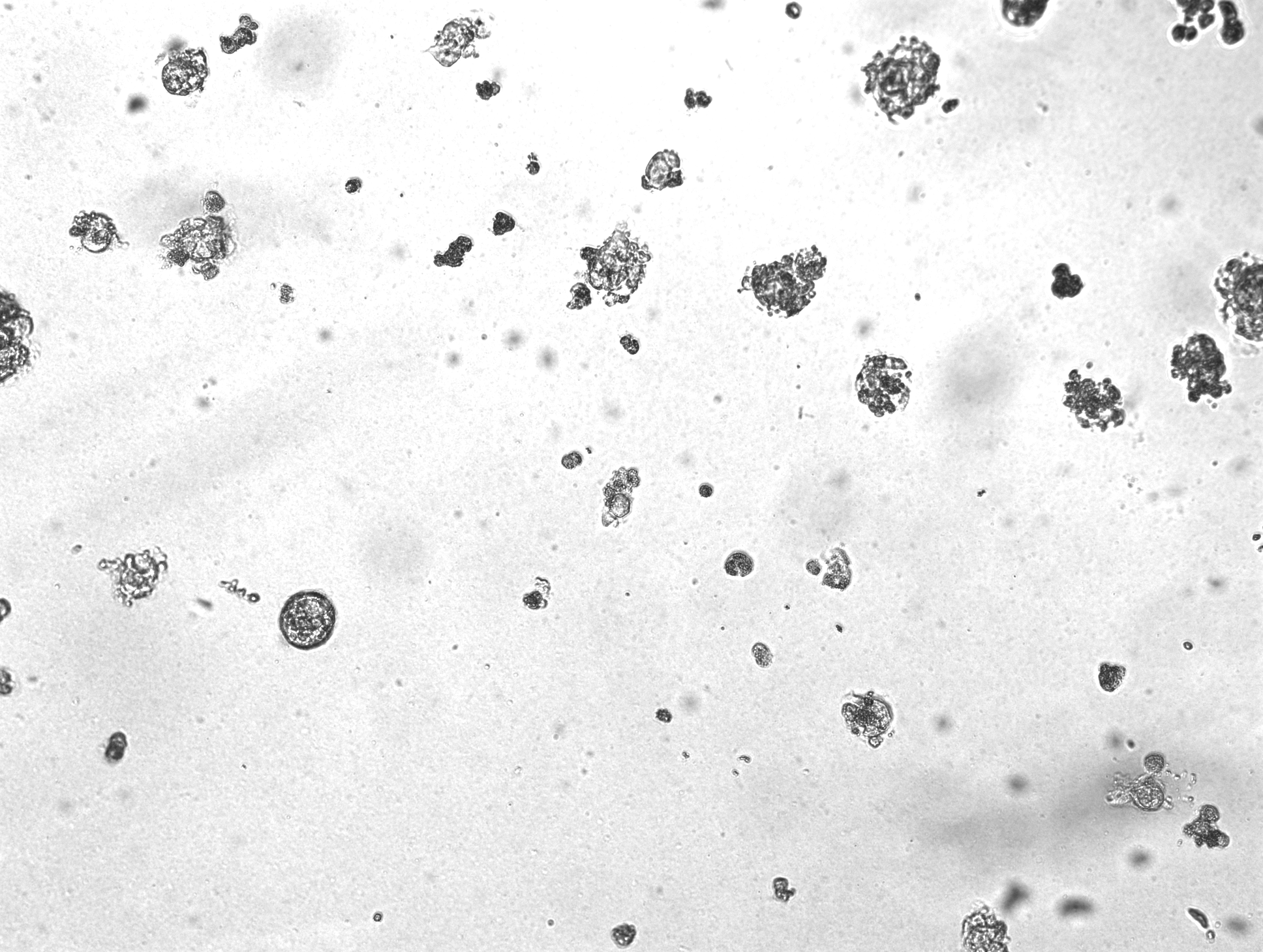

Supplement: Supplementary file 9 — Source data Fig. 6 [file 44318_2025_600_MOESM9_ESM.zip › Figure 6/6D/R2.1-1.tiff]

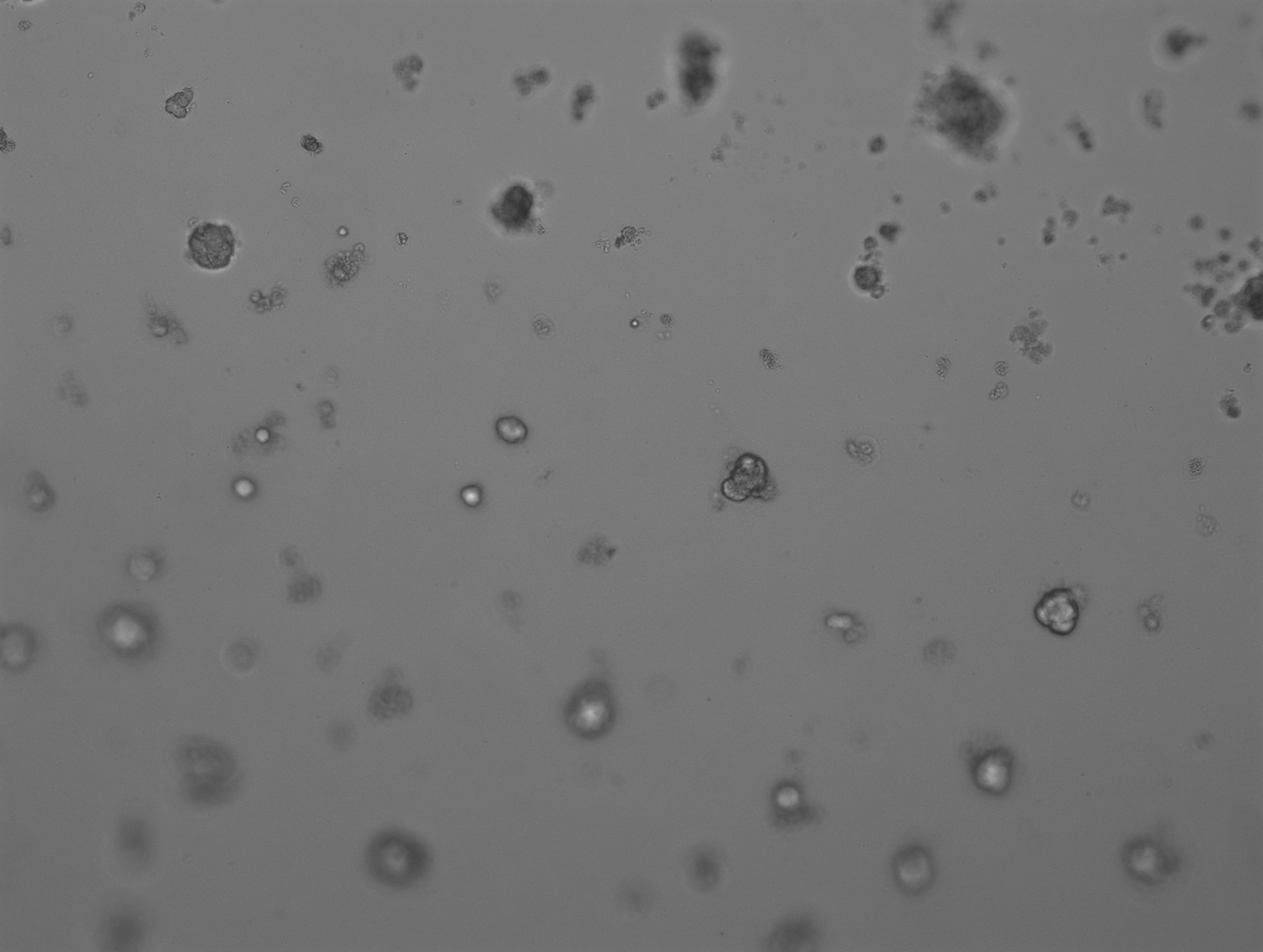

Supplement: Supplementary file 9 — Source data Fig. 6 [file 44318_2025_600_MOESM9_ESM.zip › Figure 6/6D/r1-1+.tif]

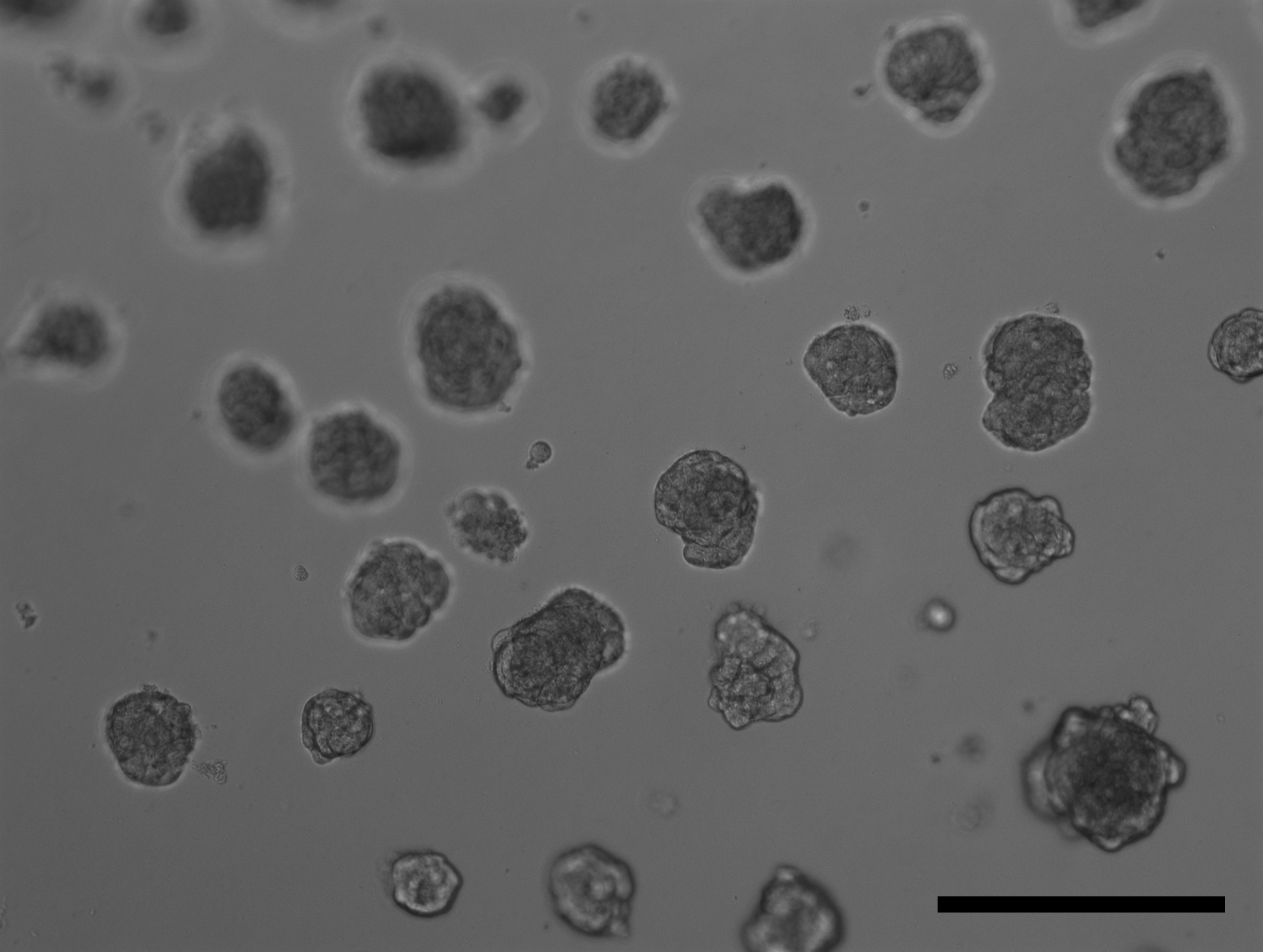

Supplement: Supplementary file 9 — Source data Fig. 6 [file 44318_2025_600_MOESM9_ESM.zip › Figure 6/6D/P1-3+.tif]

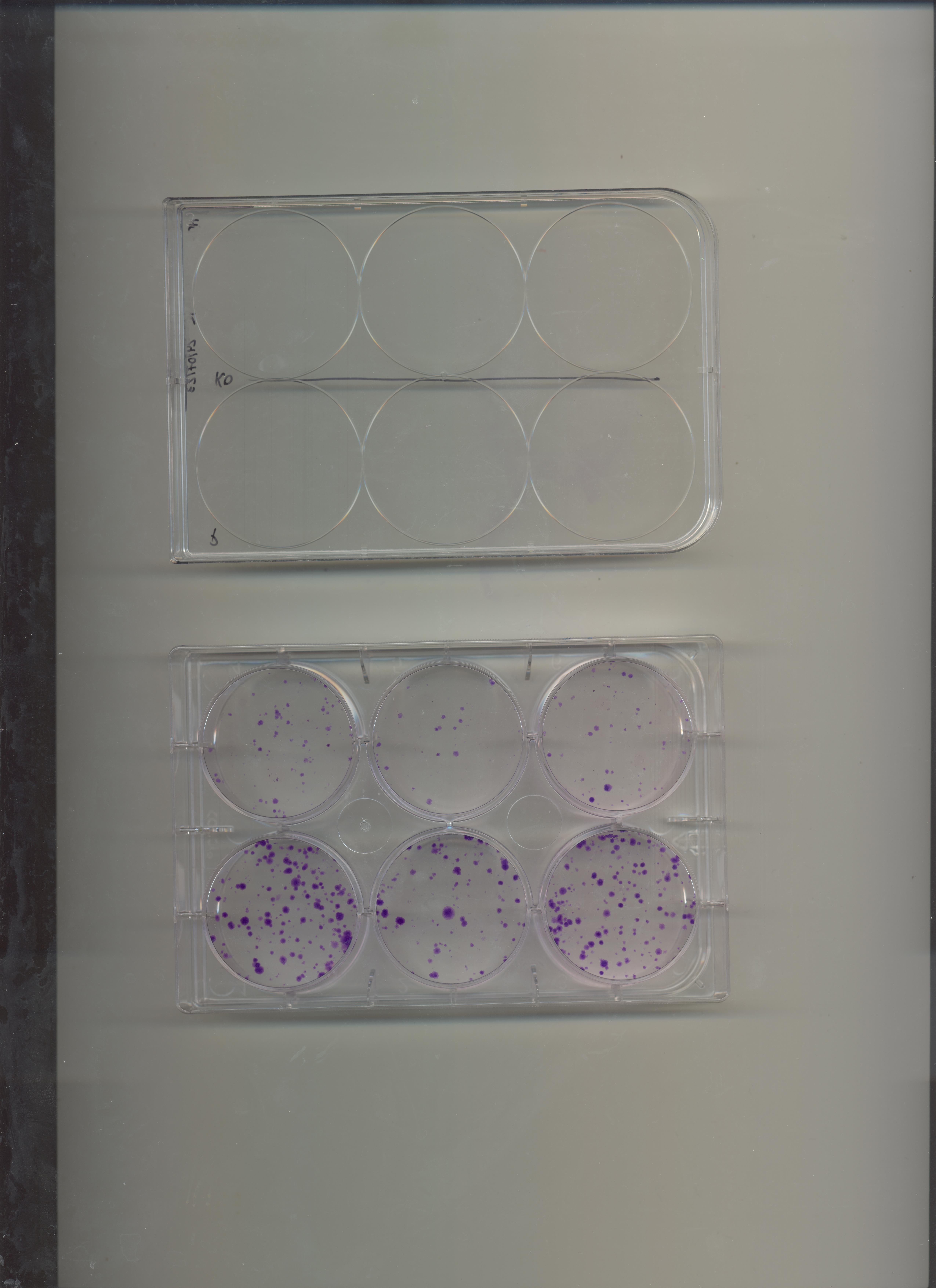

Supplement: Supplementary file 9 — Source data Fig. 6 [file 44318_2025_600_MOESM9_ESM.zip › Figure 6/6C/clonogenic image Parental and KO.jpg]

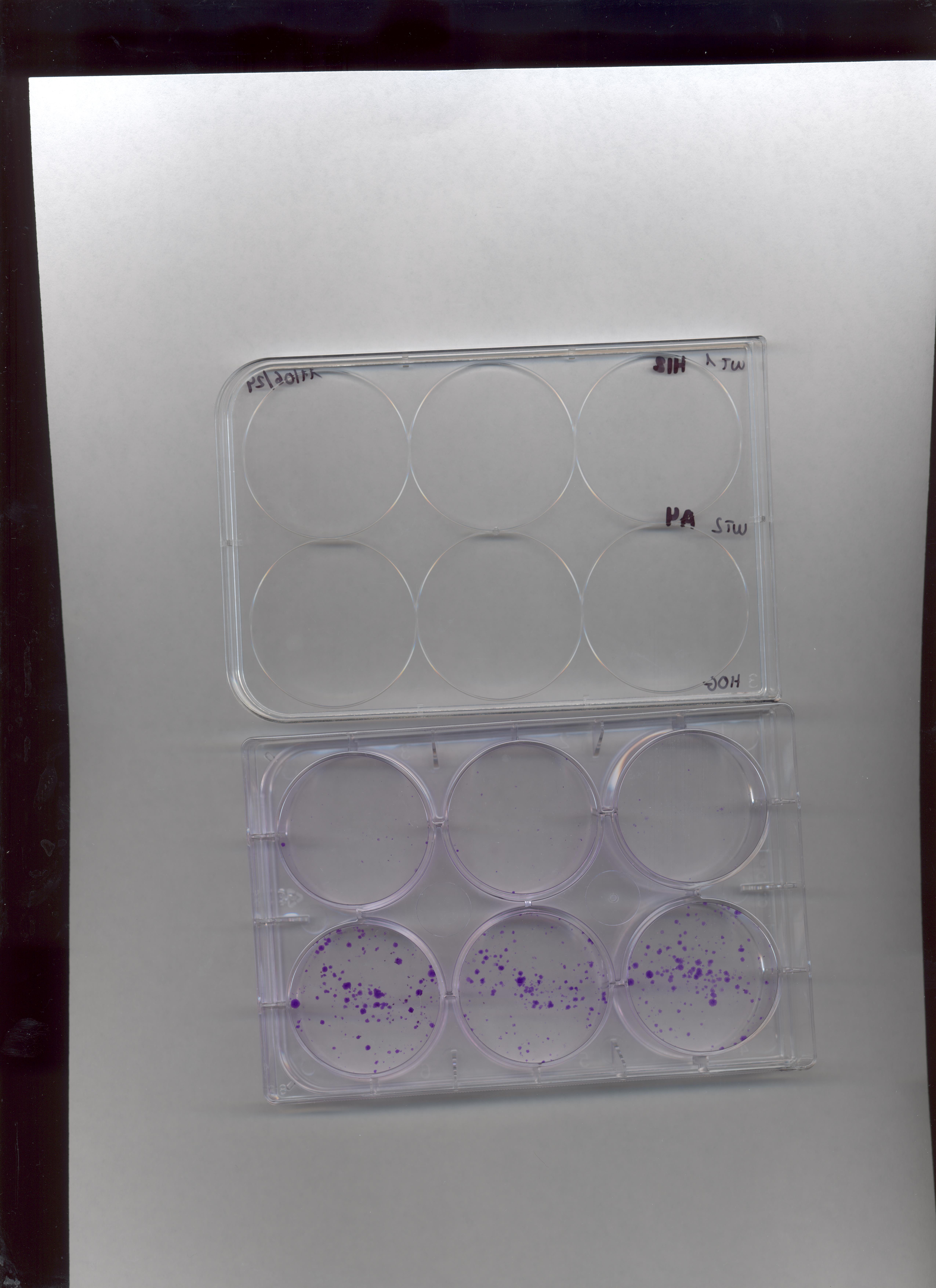

Supplement: Supplementary file 9 — Source data Fig. 6 [file 44318_2025_600_MOESM9_ESM.zip › Figure 6/6C/clonogenic image WT.jpeg]
