# Supplementary material for: Concurrence of FGFR1 mutations modulates oncogenesis in glioneuronal tumors
Source: EMBO J. 2025 Oct 31;44(24):7513–40. doi: 10.1038/s44318-025-00600-3 (PMC12705663; doi:10.1038/s44318-025-00600-3)
Supplement: Supplementary file 8 — Source data Fig. 5 [file 44318_2025_600_MOESM8_ESM.zip › Figure 5/5C/WB 5C.pptx]

## Slide 1
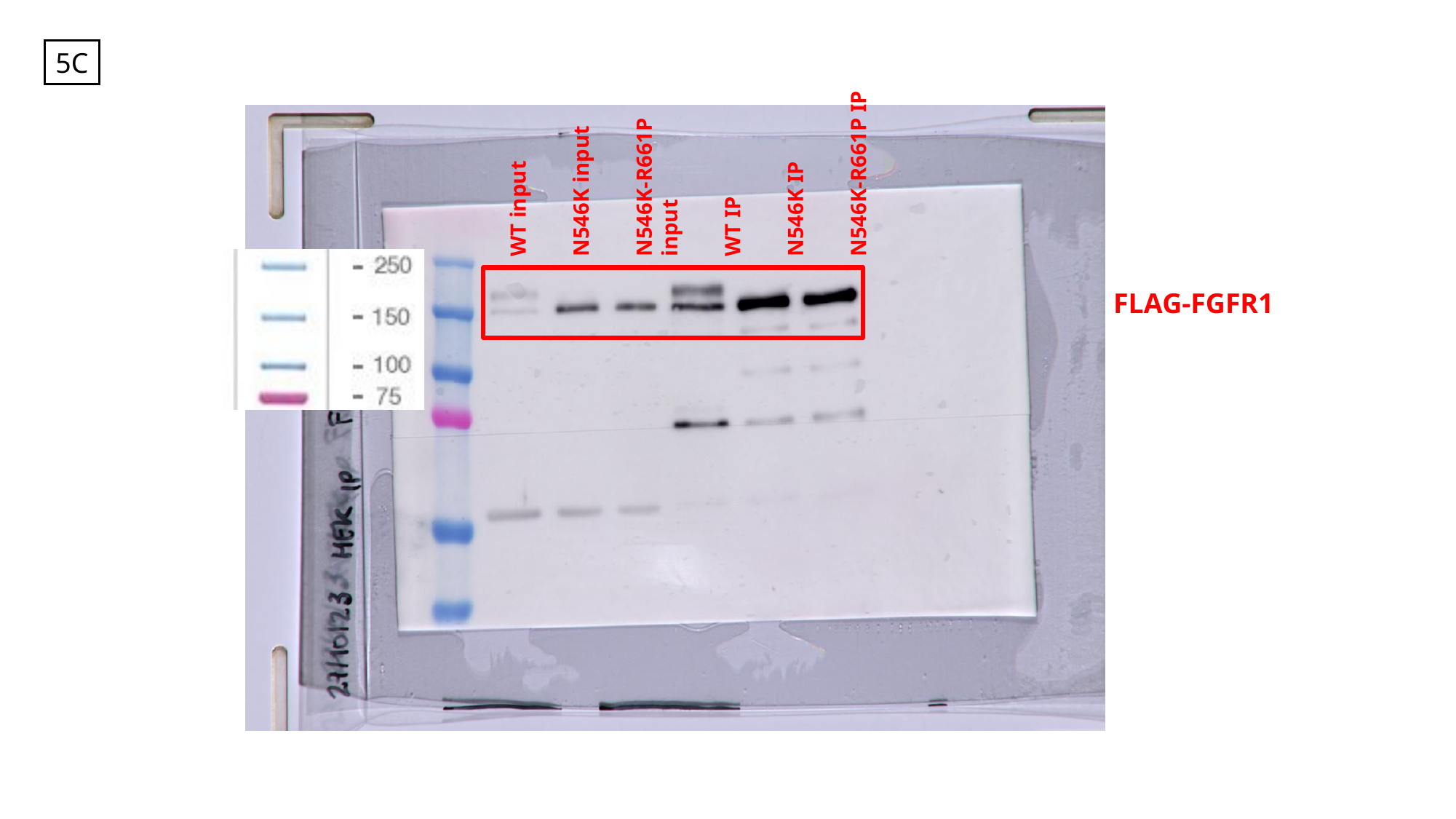

WT input
N546K input
N546K-R661P input
WT IP
N546K IP
N546K-R661P IP
5C
FLAG-FGFR1

## Slide 2
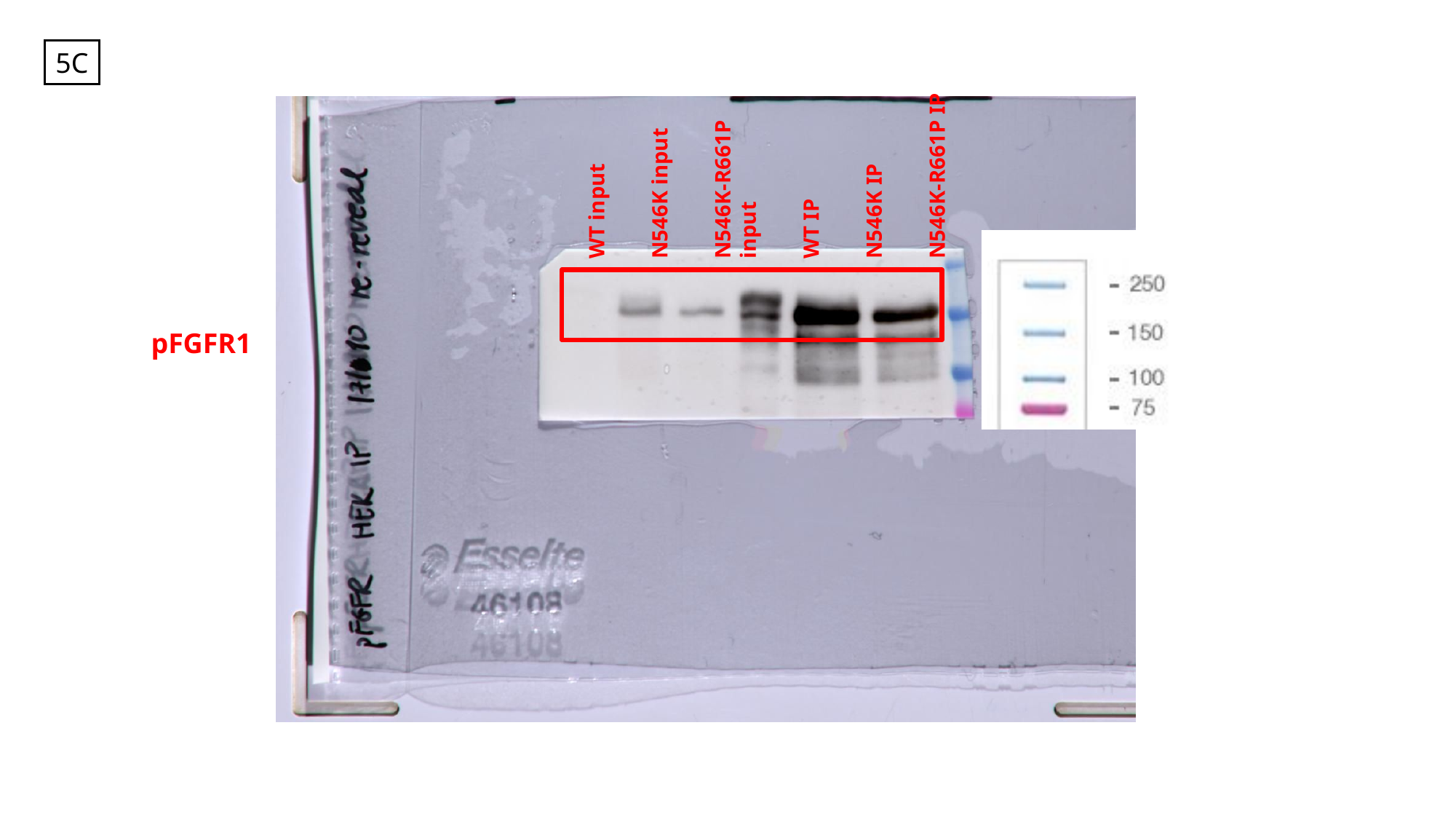

WT input
N546K input
N546K-R661P input
WT IP
N546K IP
N546K-R661P IP
5C
pFGFR1

## Slide 3
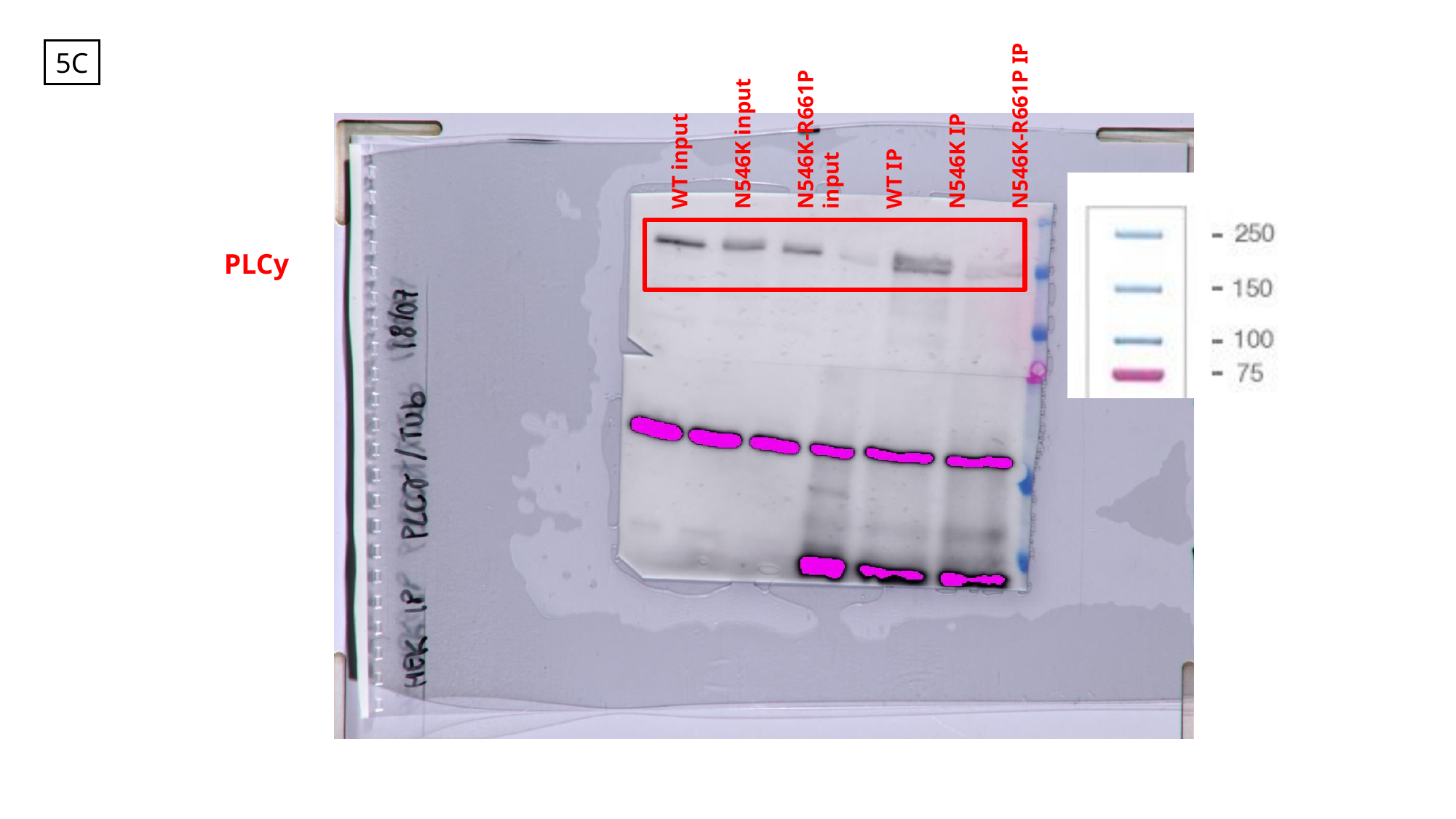

WT input
N546K input
N546K-R661P input
WT IP
N546K IP
N546K-R661P IP
5C
PLCy

## Slide 4
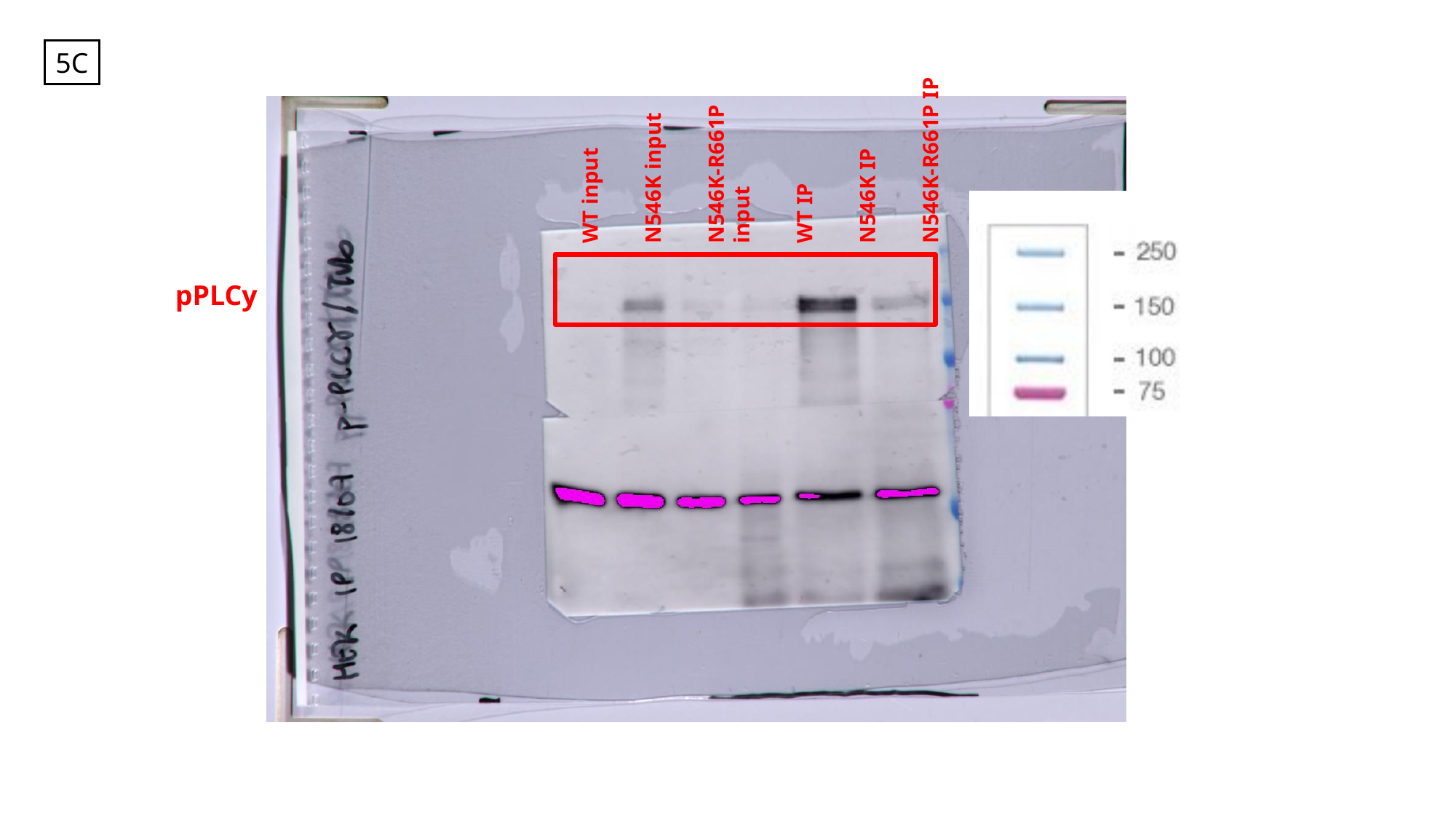

WT input
N546K input
N546K-R661P input
WT IP
N546K IP
N546K-R661P IP
5C
pPLCy
