# Supplementary material for: Concurrence of FGFR1 mutations modulates oncogenesis in glioneuronal tumors
Source: EMBO J. 2025 Oct 31;44(24):7513–40. doi: 10.1038/s44318-025-00600-3 (PMC12705663; doi:10.1038/s44318-025-00600-3)
Supplement: Supplementary file 11 — Expanded View Figures [file 44318_2025_600_MOESM11_ESM.pdf]

Expanded View Figures

Figure EV1. Changes in receptor activation, ERK signaling and phenotype in FGFR1-mutant HEK293 cell lines.

(**A**) Western blot of the parental (empty-Flag) and the six Flp-In T-REx HEK293 cell lines showing expression and activation of FGFR1 receptor and downstream MAPK/ERK signaling pathway (total and phosphorylated ERK1/2), after 24 h of Tet induction. (**B**) Bar plot of relative amounts (quantifications of western blots from $n = 6$ independent experiments) of autophosphorylated WT and mutant Flag-FGFR1 protein levels (as in Fig. 2C) obtained from the six T-REx HEK293 cell lines, induced with doxycycline (24 h). Intrinsic autophosphorylation rates shown in the plot have been evaluated correcting phospho-tyrosines (Y653/654) outputs against each respective total Flag-FGFR1 protein amount. Fold change (FC) values, obtained normalizing against WT levels, are indicated. Error bars represent standard errors of the mean ± SEM values. Significant comparisons against WT values are indicated (Kruskal–Wallis test); *P* value: $^*P = 0.0309$. (**C**) Western blot of the six cell lines used in the present study, showing expression and activation (phosphorylation of Y653/654 residues) of FGFR1, after 24 h of Tet induction and under serum starvation (0% FBS overnight) conditions. (**D**) Optical microscope captures of T-REx Flp-In HEK293 expressing WT and mutant FGFR1 proteins 24 h post Tet-induction. Scale bar: 200 µm. (**E**) Dot plot of GO enrichment analysis using the list of proteins found in Clusters 14-15 of the heatmap in Fig. 2H (R661P-specific + WT/R661P shared interactors) as input list. Significantly enriched (adj. *P* value ≤ 0.05, Benjamini–Hochberg method) have been represented. Size and color of dots represent numbers of matched genes and adjusted *P* value (*P*.adjust), respectively. GO Gene Ontology.

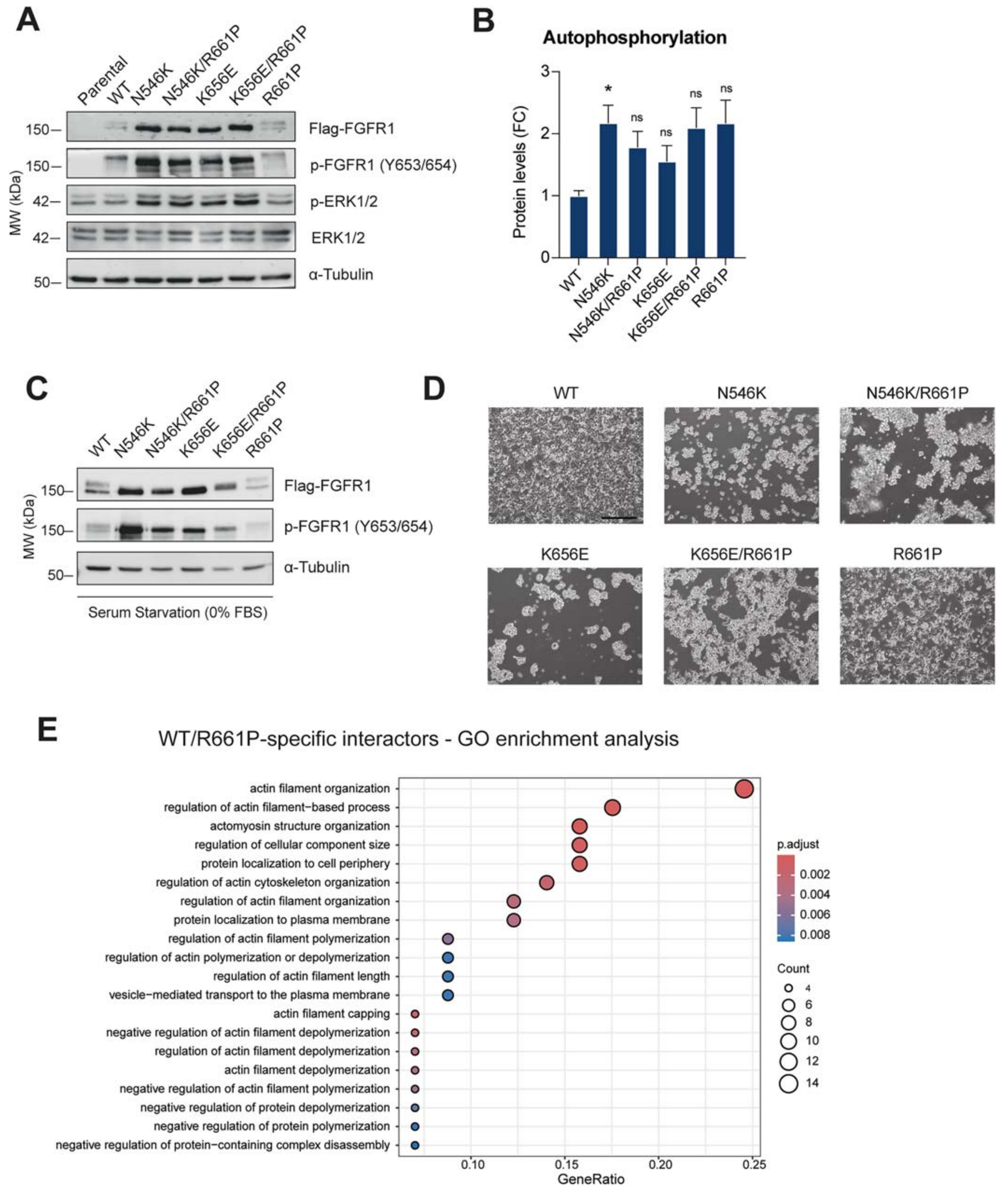
A
Parental
WT
N546K
N546K/R661P
K656E
K656E/R661P
R661P
150–
Flag-FGFR1
150–
p-FGFR1 (Y653/654)
42–
p-ERK1/2
42–
ERK1/2
50–
α-Tubulin
MW (kDa)
B
Autophosphorylation
Protein levels (FC)
3
2
1
0
*
ns
ns
ns
ns
WT
N546K
N546K/R661P
K656E
K656E/R661P
R661P
C
WT
N546K
N546K/R661P
K656E
K656E/R661P
R661P
150–
Flag-FGFR1
150–
p-FGFR1 (Y653/654)
50–
α-Tubulin
MW (kDa)
Serum Starvation (0% FBS)
D
WT
N546K
N546K/R661P
K656E
K656E/R661P
R661P
E
WT/R661P-specific interactors - GO enrichment analysis
actin filament organization
regulation of actin filament−based process
actomyosin structure organization
regulation of cellular component size
protein localization to cell periphery
regulation of actin cytoskeleton organization
regulation of actin filament organization
protein localization to plasma membrane
regulation of actin filament polymerization
regulation of actin polymerization or depolymerization
regulation of actin filament length
vesicle−mediated transport to the plasma membrane
actin filament capping
negative regulation of actin filament depolymerization
regulation of actin filament depolymerization
actin filament depolymerization
negative regulation of actin filament polymerization
negative regulation of protein depolymerization
negative regulation of protein polymerization
negative regulation of protein−containing complex disassembly
0.10
0.15
0.20
0.25
GeneRatio
p.adjust
0.002
0.004
0.006
0.008
Count
4
6
8
10
12
14

 © The Author(s)

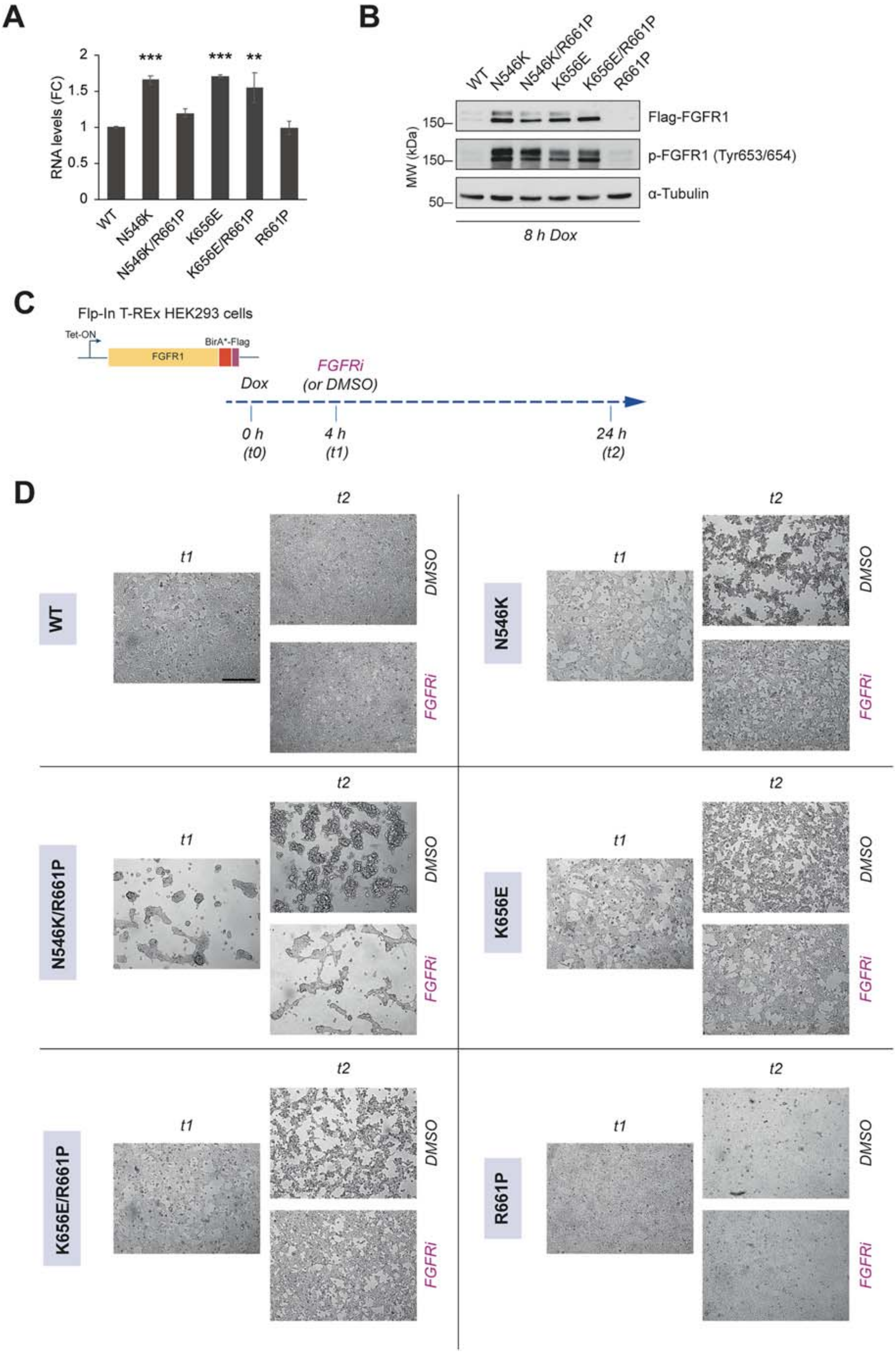
A
RNA levels (FC)
2
1.5
1
0.5
0
***
***
**
WT
N546K
N546K/R661P
K656E
K656E/R661P
R661P
B
WT
N546K
N546K/R661P
K656E
K656E/R661P
R661P
MW (kDa)
150–
150–
50–
Flag-FGFR1
p-FGFR1 (Tyr653/654)
α-Tubulin
8 h Dox
C
Flp-In T-REx HEK293 cells
Tet-ON
FGFR1
BirA*-Flag
Dox
FGFRi
(or DMSO)
0 h
(t0)
4 h
(t1)
24 h
(t2)
D
WT
N546K
N546K/R661P
K656E
K656E/R661P
R661P
t1
t2
DMSO
FGFRi

Figure EV2. N546K- and K656E-mediated protein accumulation and transformation are dependent on FGFR1 hyperphosphorylation.

(**A**) FGFR1 RNA levels in WT and the five mutant T-REx Flp-In HEK293 cell lines, obtained by RT-qPCR. Data is represented by mean ± SEM from $n = 2$ independent experiments and significant differences against WT condition have been indicated (ANOVA test); P values from left to right: ***$P = 0.00094$, ***$P = 0.00017$, **$P = 0.00731$. (**B**) Western blot assays revealing Flag-FGFR1 expression and autophosphorylation after 8 h of Tet-induction (Dox). (**C**) Experimental design for experiments with FGFR inhibitor. Cells were treated four hours after inducing Flag-FGFR1 expression either with DMSO or AZD4547 compound and lysates were collected at 24 h post-Tet induction. (**D**) Optical microscope captures of T-REx Flp-In HEK293 expressing WT and mutant FGFR1 proteins 4 h (treatment starting timepoint) and 24 h (end timepoint) post Tet-induction. For every condition, end-point pictures represent cells treated either with DMSO (upper panel) or FGFRi (bottom panel). Scale bar: 200 μm.

 © The Author(s)

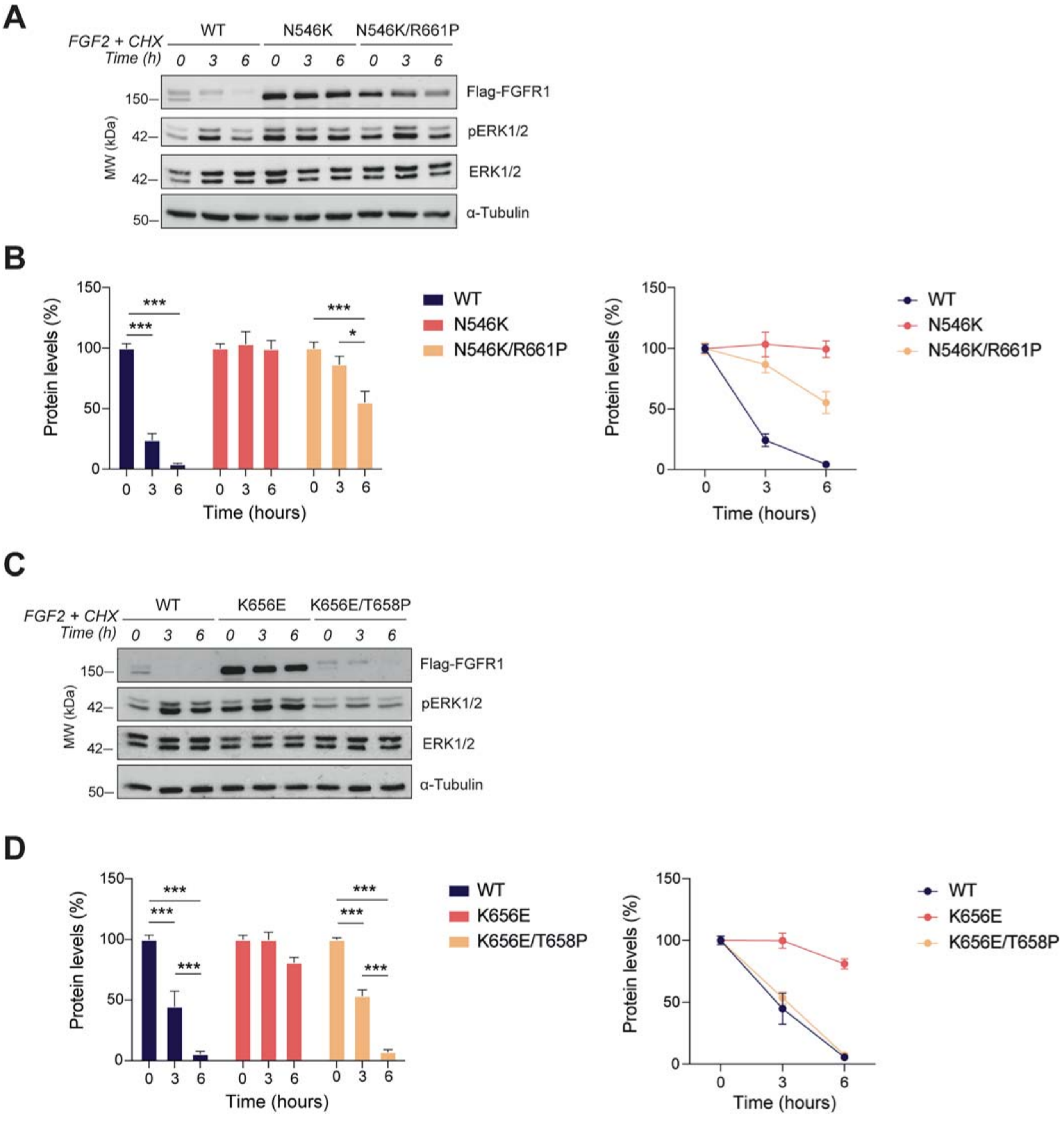

Figure EV3. Rescue of oncogenic FGFR1 proteins degradation mediated by secondary mutations.

(**A**) Western blot analysis of a representative time-course experiment comparing WT FGFR1, N546K single and double mutants showing total Flag-FGFR1 as well as total and phospho-ERK1 protein levels at 0, 3 and 6 h post-treatment with FGF2 and cycloheximide (CHX). (**B**) Relative amounts of WT, N546K single and double (N546K/R661P) mutant FGFR1 protein obtained by quantifying western blots from n = 3 independent experiments. Values have been normalized (FC) against each relative 0 h reference values. In each plot, data is represented by mean ± SEM and significant variations in protein amounts against each specific reference values have been indicated in the bar plots (ANOVA test); *P* values from left to right: ****P* < 1e-12, ****P* < 1e-12, ****P* = 0.00011, **P* = 0.0187. (**C**) Western blot analysis of a representative time-course experiment comparing WT FGFR1, K656E single mutant and K656E/T658P double mutant showing total Flag-FGFR1 as well as total and phospho-ERK protein levels at 0, 3 and 6 h post-treatment with FGF2 and cycloheximide (CHX). (**D**) Relative amounts of WT, K656E and K656E/T658P FGFR1 protein obtained by quantifying western blots from n = 3 independent experiments. Values have been normalized (FC) against each relative 0 h reference values. In each plot, data is represented by mean ± SEM and significant variations in protein amounts against each specific reference values have been indicated in the bar plots (ANOVA test); *P* values from left to right: ****P* = 2.1e-08, ****P* < 1e-12, ****P* = 9.8e-05, ****P* = 2.3e-06, ****P* < 1e-12, ****P* = 2.7e-06.

© The Author(s)

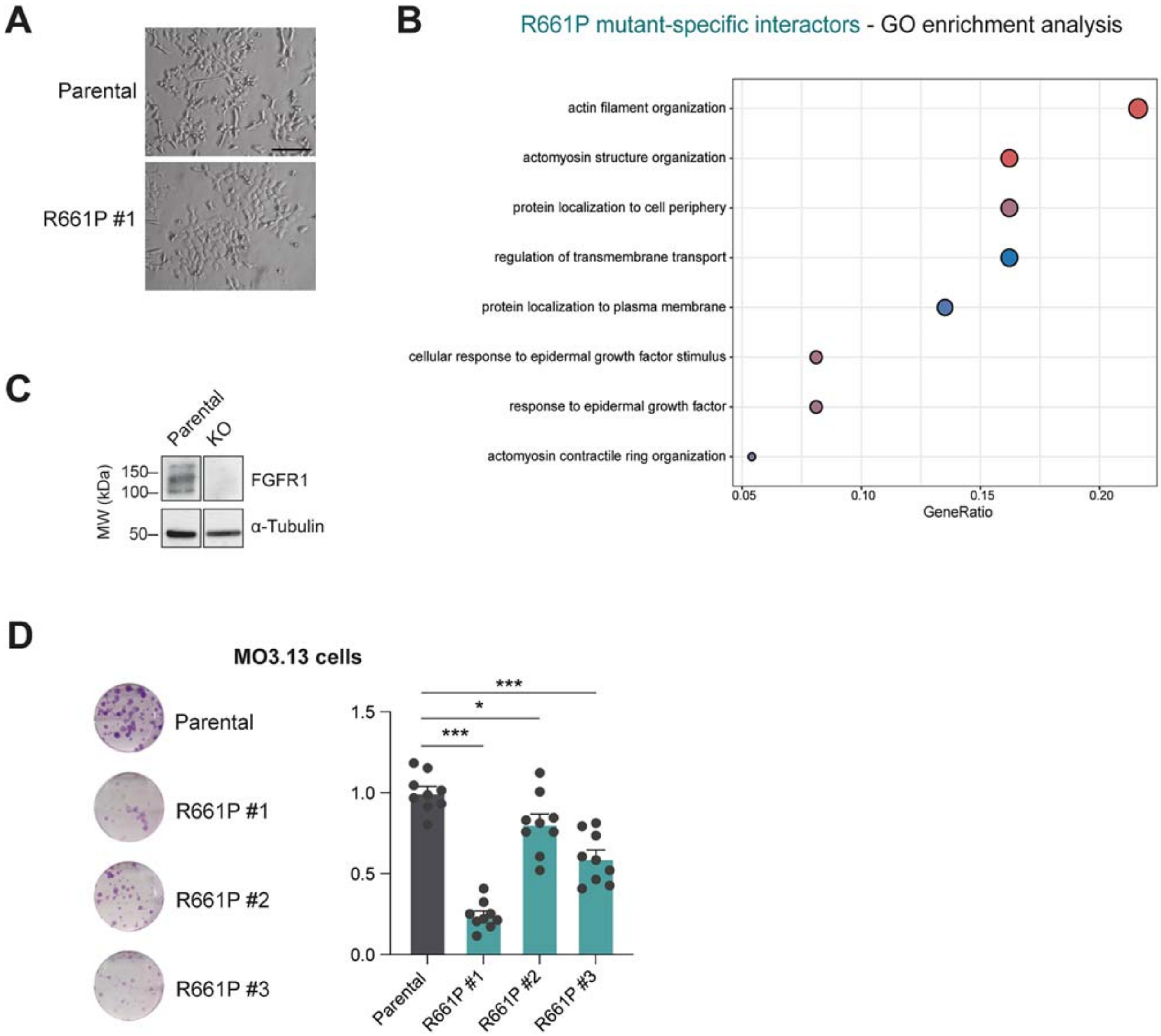

Figure EV4. FGFR1 R661P variant molecular and cellular associated phenotypes.

(**A**) Optical microscope captures of Parental (upper panel) and R661P #1 (lower panel). Scale bar: 200 µm. (**B**) Dot plot of GO enrichment analysis using the list of preys forming Cluster 14 of the heatmap in Fig. 2H (BioID R661P-specific interactors). Size and color of dots represent numbers of matched genes and adjusted *P* value (p.adjust), respectively. (**C**) Western blot of FGFR1 protein confirming absence of FGFR1 expression in KO HOG cells, compared to Parental control. GO Gene Ontology. (**D**) Colony forming assays comparing CRISPR-edited, FGFR1-R661P MO3.13 clones with the parental cell line. Three homozygous R661P/R661P clones have been assayed. Microscope captures of representative wells for each condition have been included on the left. Quantification of $n \geq 3$ experiments have been plotted, normalized against the parental cell line. Replicates are represented by black dots. Data is represented by mean ± SEM and significant variations in protein amounts against each specific reference values have been indicated (ANOVA test); *P* values from left to right: ****P* = 4.3e-12, **P* = 0.0306, ****P* = 4.4e-06.

 © The Author(s)
